# Automated High-Throughput RNAi Screening in Human Cells Combined with Reporter mRNA Transfection to Identify Novel Regulators of Translation

**DOI:** 10.1371/journal.pone.0045943

**Published:** 2012-09-27

**Authors:** Claudia M. Casanova, Peter Sehr, Kerstin Putzker, Matthias W. Hentze, Beate Neumann, Kent E. Duncan, Christian Thoma

**Affiliations:** 1 Department of Medicine II, University Hospital of Freiburg, Freiburg, Germany; 2 EMBL, Heidelberg, Germany; 3 Center for Molecular Neurobiology, University Medical Center Hamburg-Eppendorf, Hamburg, Germany; Pohang University of Science and Technology, Republic of Korea

## Abstract

Proteins that promote angiogenesis, such as vascular endothelial growth factor (VEGF), are major targets for cancer therapy. Accordingly, proteins that specifically activate expression of factors like VEGF are potential alternative therapeutic targets and may help to combat evasive resistance to angiogenesis inhibitors. VEGF mRNA contains two internal ribosome entry sites (IRESs) that enable selective activation of VEGF protein synthesis under hypoxic conditions that trigger angiogenesis. To identify novel regulators of VEGF IRES-driven translation in human cells, we have developed a high-throughput screening approach that combines siRNA treatment with transfection of a VEGF-IRES reporter mRNA. We identified the kinase MAPK3 as a novel positive regulator of VEGF IRES-driven translation and have validated its regulatory effect on endogenous VEGF. Our automated method is scalable and readily adapted for use with other mRNA regulatory elements. Consequently, it should be a generally useful approach for high-throughput identification of novel regulators of mRNA translation.

## Introduction

mRNA translation by the ribosome is the ultimate step in the expression of the ∼20,000 human genes that encode proteins. Regulation of this event- ‘translational control’- ensures that the right amount of each protein is synthesized in the right place within an organism or cell at the right time. Translational control of gene expression plays a crucial role in adaptive cellular responses to external stimuli [1] and failure to properly regulate protein synthesis is a common feature of many diseases, including cancer [2]. Under normal physiological conditions, translation initiates via a ‘cap-dependent’ mode, in which recruitment of the small ribosomal subunit to the mRNA involves the 7-methyl-guanosine (‘cap’) structure, located at the 5′ end of cellular mRNAs [3], [4]. This interaction is mediated by the cytoplasmic cap-binding complex eIF4F, which enables recruitment of other translation initiation factors, scanning to the start codon, and joining of the large ribosomal subunit for translational elongation and protein synthesis [3], [4], [5]. Cap-dependent initiation appears to be the dominant mode for most cellular mRNAs under most conditions, and is the target of a wide variety of regulatory mechanisms [1]. However, certain viral RNAs and some mammalian mRNAs can use alternatives to cap-dependent initiation and thus are translated efficiently under conditions in which cap-dependent translation is repressed, such as apoptosis, mitosis, hypoxia, and cellular stress [6], [7]. In these cases, translation initiation occurs efficiently independent of the cap structure and many of the associated translation initiation factors [6].

The subset of cellular mRNAs (∼3%) that apparently can be translated efficiently when cap-dependent translation is generally compromised [8] includes cell growth regulators that are critical in cancer [2], [9], [10]. One important example of major clinical relevance is the mRNA encoding vascular endothelial growth factor-A (VEGF-A, henceforth referred to as ‘VEGF’). As a key regulator of tumor angiogenesis, VEGF plays a crucial role in cancer progression for essentially all solid tumors [11], [12] and consequently is a major oncology drug target. VEGF is also important for development and maintenance of the nervous system and both VEGF and regulators of VEGF signaling are of great therapeutic interest in neurodegenerative disease and acute neurological disorders, including cerebral ischemia/stroke [13], [14]. Cap-independent translation of the VEGF mRNA is mediated by two internal ribosome entry sequences (VEGF IRES-A and –B, respectively) located in the 5′ untranslated region (UTR) [15]. Cancer-relevant cellular stress conditions, such as hypoxia, can activate cap-independent translation mediated by these and other IRESs, while simultaneously repressing cap-dependent translation [16], [17], [18]. Thus, in many cancers tumorigenesis involves a switch enabling more cap-independent translation, which appears to be important for tumor progression [2]. Accordingly, ‘druggable’ specific positive regulators of the translational activity of VEGF IRES (and perhaps other cellular IRESs) could potentially be attractive additional therapeutic targets in oncology. However, to our knowledge no cellular factors that specifically modulate VEGF IRES activity have yet been identified.

The discovery that RNAi works effectively in mammalian cells [19] paved the way for high-throughput RNAi screening to address such questions in a global manner. Here, we present a novel, automated, easily scalable high-throughput screening approach that enables the identification of regulatory proteins that modulate the translational activity of a specific mRNA element contained in a transfected reporter mRNA. We took advantage of the well established protocol for solid-phase reverse transfection of cells on small interfering siRNA transfection mixes in coated 96-well plate format [20]. This robust approach routinely achieves strong and specific knockdowns, but has so far been used predominantly for high-content microscopy applications. Here we significantly expand its range of use by optimizing its compatibility with automated transfection of a reporter mRNA of interest. Specifically, we present a robust method to: 1) quantitatively analyze IRES-dependent translation and 2) compare it with conventional cap-dependent translation activity using monocistronic reporter mRNAs. Our approach addresses the numerous caveats for studying IRES activity that are inherent to the commonly used DNA transfection method with bicistronic reporters, which has led to much confusion and contention in the IRES translation field [21]. For this reason, we expect the method presented here to be a more sensitive screening tool with a much lower false positive rate.

In a proof-of-concept screen we assessed the potential role of 702 human kinases and 298 human phosphatases in VEGF IRES-driven translation. 91 genes that qualified as hits in the primary screen were selected for secondary validation assays, in which specificity for IRES-driven translation was examined by transfecting the original VEGF IRES-driven reporter mRNA and a cap-driven reporter mRNA. Ultimately, we identified three kinases specifically involved in IRES-, but not in cap-dependent translational regulation. For one of these, MAPK3, we show here that it is a bona fide novel, positive-acting, post-transcriptional regulator of VEGF production. In our view this provides a striking demonstration of the power of this method for identifying novel regulators of mRNA translation. Importantly, the method can be easily scaled up for genome-wide screens and could also be readily adapted for use with other mRNA elements of (clinical) interest.

## Results

### Combining RNAi with mRNA Transfection Enables High-throughput Screening for Human Kinases and Phosphatases that Regulate mRNA Translation

**Figure 1:**
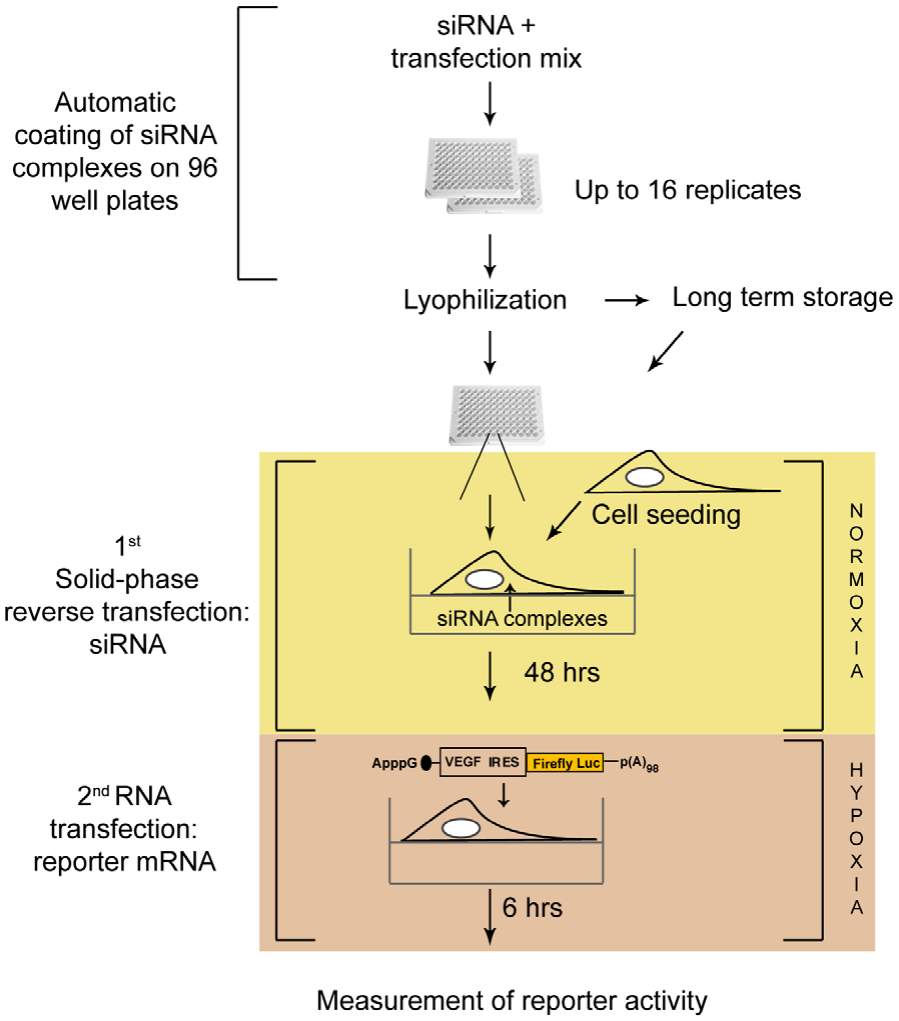
Workflow of the high-throughput siRNA screening approach to identify novel regulators of mRNA translation. The strategy features automated generation of lyophilized siRNA-coated 96 well plates, followed by two independent RNA transfections. Plates are first coated with siRNAs transfection mixes, cells are then seeded onto siRNA-coated plates (solid-phase reverse transfection) and incubated for two days to allow reduction of the protein levels of the targeted genes. Subsequently, a second RNA transfection is performed to introduce the reporter mRNA of interest. Finally, the effects of each siRNA knock-down are measured.

To identify potential regulators of VEGF IRES-dependent translation we developed a general RNAi screening approach integrated with mRNA transfections. In the first step siRNA transfection complexes are ‘solid-phase reverse transfected’ (i.e. cells are added to plates coated with lyophilized siRNAs transfection mixes) [20]. Subsequently, a second RNA transfection introduces a reporter mRNA whose structure can be modified according to the interest of the investigator. Transfection mixes containing siRNAs of interest are distributed to 96-well plates using automated liquid handling (Fig. 1). Lyophilization of the plates allows long-term storage and guarantees “ready to transfect” plates with similar transfection efficiency for up to 15 months [20]. We have chosen to perform the screen in the HeLa human cell line because: I) the established siRNA treatment procedure is optimized for these cells; II) the VEGF IRES is active (Fig. S1A) and III) and responds to low oxygen tension (hypoxia, 0.7% O_2_) with an approximately 3-fold activation (Fig. S1B). After cell seeding, siRNA solid-phase reverse transfection is performed for 48 hours under normoxic conditions (Fig. 1). Subsequently, cells are transfected with the reporter mRNA of interest, in this case a firefly luciferase (FLuc) reporter containing the VEGF IRES elements a and b and a non-physiological adenosine cap structure (‘A-cap’). The A-cap maintains stability of the mRNA, but is not recognized by the cytoplasmic cap binding complex [22], [23] and thereby ensures that translation of this reporter mRNA is driven in a cap-independent mode via the VEGF IRES contained in the mRNA 5′ UTR (Fig. 1). For this screen cells are incubated under hypoxic conditions (0.7% oxygen tension) after reporter mRNA transfection for 6 hours prior to lysis and measurement of Fluc activity (Fig. 1), since this mimics the physiological condition under which the VEGF IRES is maximally active. Importantly, direct introduction of a reporter mRNA in the second step is designed to significantly limit the number of hits to those that affect translation, as opposed to other steps in gene expression.

### Identification of 91 Potential Novel Regulators of VEGF IRES-dependent Translation

**Figure 2:**
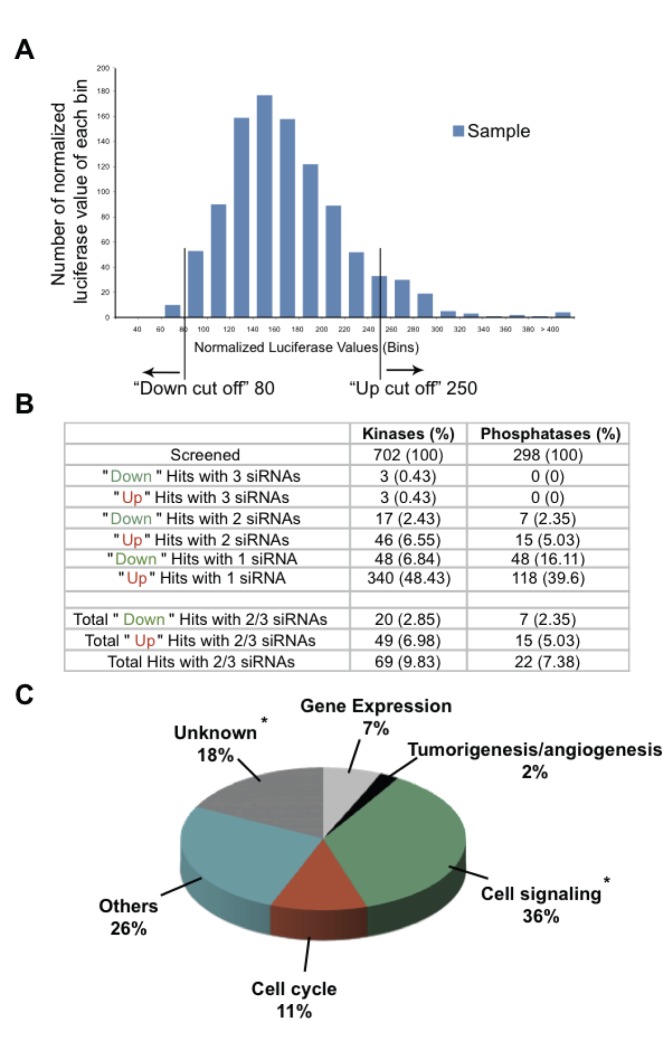
Identification of kinases and phosphatases modulating VEGF IRES activity. (a) Frequency distribution of the normalized median values of all samples (kinases and phosphatases). Numbers of normalized median values in each bin are shown on the y-axis, bins are shown on the x-axis. The cut-off values for up and down hits are indicated. (b) 702 kinases and 298 phosphatases were screened with three different siRNA sequences in three independent reactions, each performed in triplicates. Among them 69 kinases and 22 phosphatases had a similar effect on VEGF IRES activity when silenced with at least 2 different siRNA sequences in independent reactions. These 91 genes have been considered for further validation in secondary screening experiments. (c) Schematic classification of the 91 genes selected for validation studies according to their described function in GO annotation. * Two of the specific hits belong to the “Unknown” group and one to the “Cell signaling” group.

We screened 702 human kinases (Table S1) and 298 human phosphatases (Table S2). Each gene was targeted with three different siRNA sequences (Tables S1 and S2), each in an independent reaction. Pilot experiments revealed low well-to-well variability in different wells on different plates (Fig. S2). Nevertheless, to ensure assay quality, each reaction was performed in triplicate on separate plates. In other words, each gene was analyzed in 9 independent reactions in total. Each value was normalized to the median value of four scrambled siRNA negative control reactions (100) on the same plate. The median value of the triplicate was obtained from the normalized individual values. siRNAs targeting firefly luciferase (FLuc) were used as positive controls. In principle, silencing of a defined kinase or phosphatase can result in either a higher (‘Up Hit’) or a lower (‘Down Hit’) production of luciferase, due to the corresponding modulation of VEGF IRES activity. We used the frequency distribution of the normalized median value of all samples to define cut-off values of 250 and 80 for Up and Down hits, respectively in order to give a hit rate <10% (Fig. 2A). From the total number of siRNAs tested, siRNAs targeting 457 kinases and 188 phosphatases affected VEGF IRES activity. Among them only 69 kinases and 22 phosphatases had a similar effect on VEGF IRES activity when silenced with at least 2 different siRNA sequences in independent reactions: 64 genes behaved as negative regulators and 27 as positive regulators of VEGF IRES function (Fig. 2B). Genes identified with two or more siRNAs in individual reactions are most likely true hits rather than false positives due to off target effects. The use of silencer select siRNAs also reduced the likelihood of off target effects [24]. We re-tested the 91 genes (69 kinases and 22 phosphatases) that were identified with at least two siRNAs (Fig 2B). These genes have been grouped on the basis of their relevance in particular biological processes, according to Gene Ontology (GO) annotation (Fig. 2C). This classification revealed that: (1) 36% of the genes participate in cell signaling pathways, (2) 18% are genes with unknown function, and (3) 2% are previously linked to tumorigenesis/angiogenesis.

### Secondary Screen for Hit Verification and Determination of Specificity for VEGF IRES Translation

**Figure 3:**
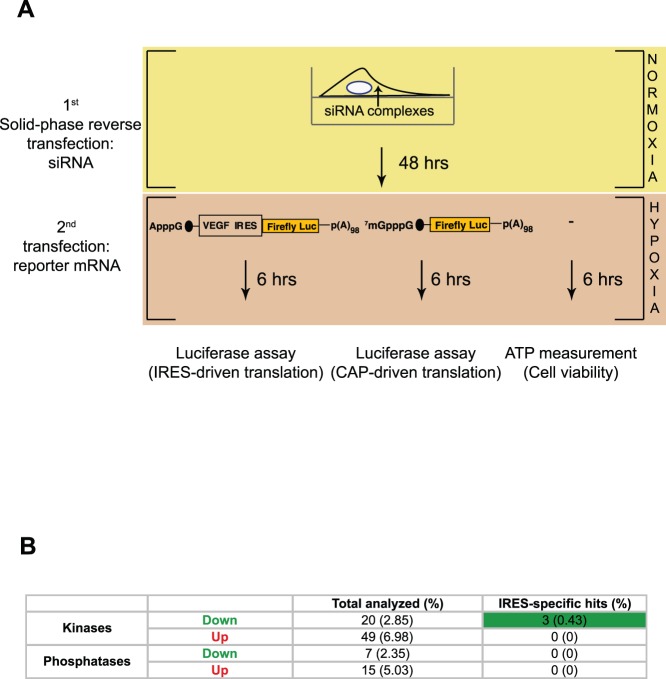
Secondary screen of 91 candidate regulators of VEGF IRES-dependent translation. (a) Workflow of the secondary screen. Cells were reverse-transfected with individual siRNAs designated as hits from the primary screen (Table S1 and S2). This was followed 48 hours later by three assays which were designed, respectively, to assess: 1) *specificity* of the effects for IRES-driven translation and 2) to determine effects on cell *viability* that might confound the other analyses. (b) Summary of secondary screening data.

In secondary validation experiments we aimed to: 1) identify genes specifically showing an effect on IRES, but not on cap-dependent translation, and 2) exclude genes affecting cell viability that might be indirectly affecting protein synthesis. Validation experiments were performed according to the workflow depicted in Fig. 3A. To test specificity, the original VEGF IRES-driven reporter mRNA and a reporter mRNA containing a physiological cap structure, but lacking VEGF IRES sequences were transfected. Confirmed hits that showed no effect on the cap-driven reporter mRNA were considered IRES-specific. Hits that specifically affected IRES-, but not cap-dependent translation were then assessed for their effect on cell viability through ATP measurement. Following this workflow, we identified three kinases that specifically affected IRES-, but not cap-dependent translation, and that had no effect on cell viability when targeted by siRNAs (Fig. 3B and Table S3).

### Novel Role for MAPK3 as a Positive Regulator of VEGF IRES Activity and VEGF Protein Levels

**Figure 4:**
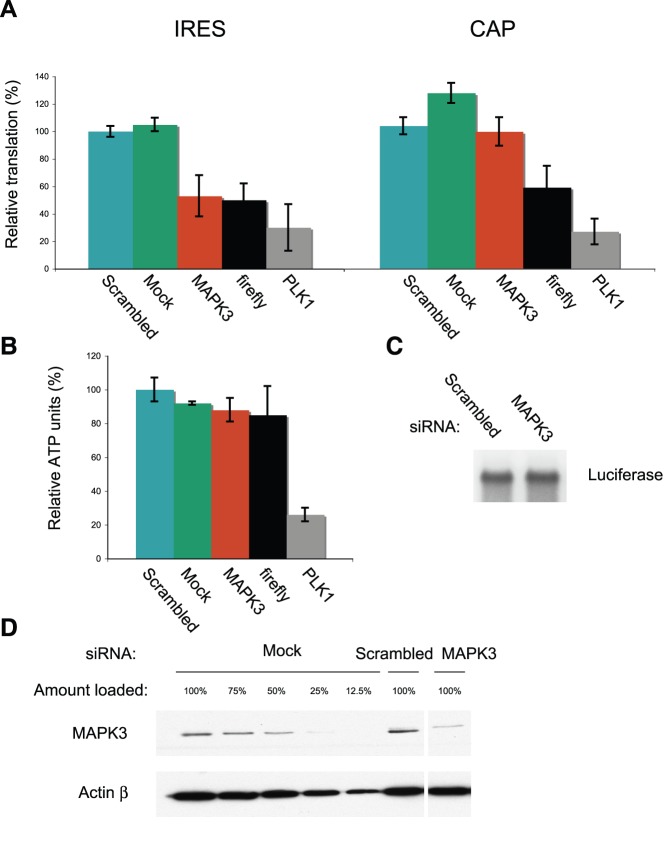
MAPK3 specifically regulates VEGF IRES-dependent translation. (a) HeLa cells were reverse transfected with the indicated siRNAs. This was followed 48 h later by reporter mRNA transfection for IRES-driven translation (left panel) and cap-driven translation (right panel). Luciferase activity was analyzed and the corresponding relative translation rate is indicated. (b) Quantification of ATP content as an indicator of cell viability. (c) Physical stability of the reporter VEGF IRES luciferase mRNA analyzed by Northern blotting. (d) Western blot of MAPK3 in siRNA-treated cells or control cells treated with scrambled siRNA. Beta-actin serves as a control for loading/transfer efficiency.

One of the three confirmed IRES-specific regulators with no effect on cell viability was mitogen-activated protein kinase 3 (MAPK3), also known as ERK1, which we selected for further validation experiments. siRNA-mediated reduction of MAPK3 levels by approximately 70% (Figure 4D) led to a 50% decrease in VEGF IRES Fluc reporter mRNA activity (Fig. 4A, left panel), but had no effect on cap-dependent Fluc reporter mRNA activity (Fig. 4A, right panel). Mock and scrambled siRNA reverse transfection (negative controls) did not affect translation of either reporter. siRNAs targeting the Fluc coding sequence or Polo-Like Kinase 1“ (PLK1) served as positive controls for RNAi efficacy. Both led to a reduction of Fluc levels for both IRES- and cap-dependent reporter mRNAs, the former due to RNAi and the latter as an indirect consequence of impaired growth (silencing of PLK1 leads to a prometaphase arrest followed by apoptosis [25]. Importantly, the reduction in reporter expression in MAPK3 siRNA-treated cells is not due to changes in mRNA abundance, as shown in Fig. 4C. To rule out that the reduction in VEGF IRES translation due to MAPK3 siRNA action might result from an indirect effect on cell growth, we assessed cell viability through ATP measurement. As a positive control we silenced PLK1, which led to a commensurate reduction of ATP counts to 25%, as expected (Fig. 4B). In contrast, silencing of MAPK3 had almost no effect on cell viability (Fig. 4B). Taken together, these observations strongly suggest that MAPK3 is a novel specific activator of VEGF IRES-driven translation.

**Figure 5:**
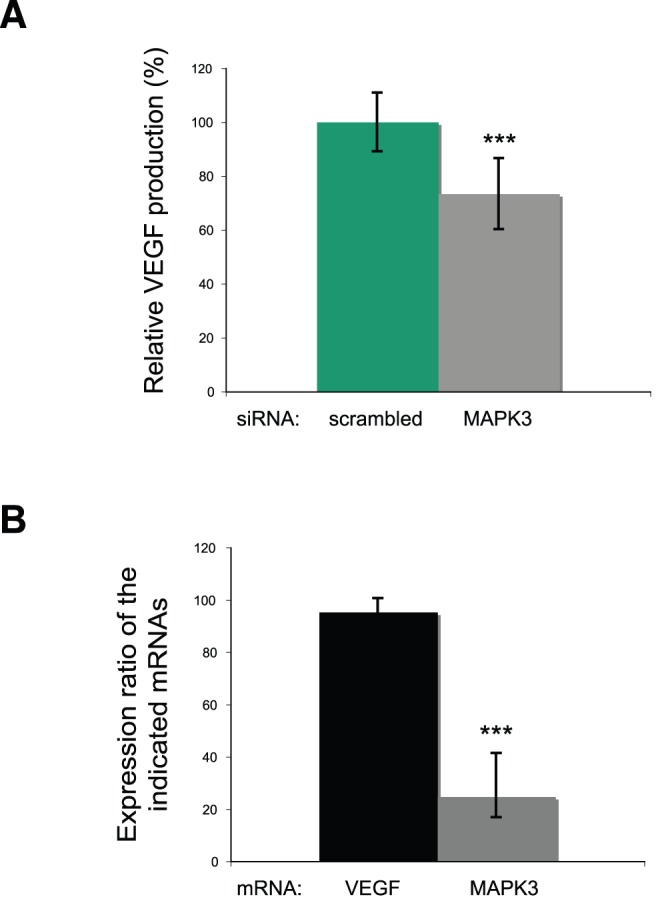
MAPK3 specifically regulates endogenous VEGF expression without affecting mRNA stability. (a) Determination of endogenous VEGF by ELISA**.** Error bars represent standard deviations calculated from 3 independent experiments, each performed at least in duplicates. Statistical analysis of differences between controls and experimental samples were performed with the Microsoft Excel unpaired, type 2, Student’s t test. (b) Endogenous VEGF and MAPK3 mRNA levels measured by qRT-PCR. The expression ratio of the indicated endogenous mRNAs (VEGF and MAPK3) in MAPK3 siRNA-treated cells relative to scramble siRNA treated cells (%) is shown. Statistical analysis of differences between controls and experimental samples were performed with the Microsoft Excel unpaired, type 2, Student’s t test. A p-value of >0.5 and <0.05 was determined for VEGF and MAPK3, respectively. Relative VEGF and MAPK3 mRNA levels, standard deviations and statistical significance were calculated with REST 2009 software as described in “Material and Methods”.

If translation driven by the VEGF IRES is contributing significantly to VEGF protein levels under hypoxia, then our results predict that MAPK3 knockdown would lead to a reduction in endogenous VEGF protein levels. To test this prediction we wanted to use as reproducible and quantitative an assay as possible, since we expected on the basis of our mRNA reporter assays to see a relatively modest reduction in VEGF levels due to MAPK3 knockdown. We therefore analyzed secreted VEGF levels by ELISA, which is typically superior to immunoblotting for accurate quantification of mild changes and is a standard method for monitoring changes in endogenous VEGF levels [18]. HeLa cells were seeded on siRNA-coated 96 well plates and grown for 24 hours under normoxic conditions, followed by incubation under hypoxic conditions for 24 hours. Thereafter, ELISA analysis of VEGF secretion was performed, which revealed that MAPK3 depletion causes a 25% reduction of VEGF (Fig. 5A). Statistical analysis was performed as described in “Methods” and achieved p = 0.00063. To determine whether these effects are due to an effect on endogenous VEGF mRNA stability, RT-qPCR experiments were performed: down-regulation of MAPK3 by RNAi led to a significant reduction of endogenous MAPK3 mRNA levels to 25% (Fig. 5B), demonstrating that RNAi was effective. However, endogenous VEGF mRNA levels were not affected (Fig. 5B). Thus, the reduction of VEGF protein levels in MAPK3 siRNA-treated cells is not due to changes in transcription or mRNA abundance. These data demonstrate a novel functional role for MAPK3 as a bona fide positive regulator of endogenous VEGF protein levels in human cells. Taken together with our reporter mRNA results described above, these data strongly suggest that the mechanism by which MAPK3 affects VEGF protein levels is through specific modulation of VEGF IRES-dependent translation. To our knowledge this is the first demonstration of a signaling pathway kinase that stimulates VEGF IRES translational activity without affecting cap-dependent translation. We interpret our ability to identify such a factor through the relatively small screen described here as strong evidence of the power of our screening methodology.

## Discussion

Regulation of ‘gene expression’ has been viewed historically as a primarily nuclear event controlled by variations in transcription factor activity. More recent developments indicate that translational regulation of gene expression is not only common, but also a major contributor to regulation of protein expression levels. This paradigm shift has been driven in part by the discovery of microRNAs as major cytoplasmic regulators of gene expression [26], [27], [28], as well as the development of functional genomic and proteomic approaches that enable large-scale quantitative analysis- and therefore direct comparison- of the correspondence between mRNA and protein levels under different cellular conditions [29]. At the same time, there has been increasing appreciation of the major role of altered translational control in numerous human diseases, particularly cancer, and a corresponding major interest in targeting translational regulatory factors for drug discovery [2], [30], [31], [32], [33], [34], [35]. In principle, cell-based RNAi screening should be a powerful approach for identification of regulators of mRNA translation. However, we are not aware of any publication describing a screen for regulators of cellular translation in a high-throughput format in mammalian cells. A major reason for this is undoubtedly technical: translation is a ‘downstream step’ in the pathway from gene to protein, and therefore the usual genome-wide RNAi screening methodologies do not enable direct and specific assessment of translation. For example, screening approaches that rely on traditional DNA-based reporter genes are sensitive to identification of proteins affecting other steps of gene expression. This is likely to be particularly problematic for cellular IRES element-mediated translation, where using common DNA-based transfection approaches such as bicistronic reporters bear the risk that few of the molecules identified will specifically affect translation. The lower level of activity that is typical for cellular IRESs in comparison to cap- or viral IRES-driven translation makes it difficult to exclude that changes in activity observed with DNA-based strategies result from effects on other steps in gene expression, such as cryptic promoter or splicing activity [21]. While it may sometimes be possible through rigorous controls to demonstrate IRES activity using DNA reporters, this is not easily amenable to a high-throughput format.

Here we have presented a novel high-throughput RNAi screening strategy that avoids by design all of the problems associated with DNA-encoded reporters. Our approach combines highly efficient siRNA solid-phase reverse transfection with subsequent direct reporter mRNA transfection. We reasoned that this combination would be more likely to identify bona fide regulators of VEGF levels that act by specific translational mechanisms. This idea is borne out by our successful identification of three IRES-specific translational regulatory proteins in what was primarily intended to be a proof-of-concept screen. In this study we focused on the further analysis of MAPK3. The additional kinases will be the subject of future studies.

Importantly, we have also demonstrated here that MAPK3 is a bona fide regulator of endogenous VEGF expression under hypoxia in human cells (Figure 5A). This effect is independent of changes in VEGF mRNA levels (Fig. 5B), as expected from an effect of MAPK3 on the VEGF IRES. We note that reducing MAPK3 levels by 75% leads to a 25% reduction of endogenous VEGF levels. While this might seem to be a modest effect, we think it is unlikely that the effect on endogenous VEGF would be much stronger for two reasons. First, the knockdown is not complete and kinases can act catalytically. Second, the effect on the VEGF IRES reporter upon MAPK3 was only ∼50% reduction and presumably this result sets a lower bound for the magnitude of any effect on endogenous VEGF levels. In fact, the magnitude of endogenous VEGF reduction in response to MAPK3 depletion is less than that observed with our reporter (25% vs. 50% reduced, respectively). Although we do not know the exact reason for this difference, one possibility is that it relates to the large number of regulatory controls that act to tune VEGF levels within a relatively narrow range [13]. Indeed, ample evidence suggests that induction of tumor angiogenesis, a hallmark of cancer, is governed by a complex biological rheostat that senses numerous positive and negative inputs that converge through multiple mechanisms on regulation of VEGF protein levels [11]. Thus, there might be a positive compensatory mechanism that would act homeostatically to raise levels of endogenous VEGF when IRES activity is reduced by MAPK3 knockdown (e.g. increased endogenous VEGF-A transcription or VEGF protein stability). In contrast, the VEGF IRES luciferase reporter would be immune to these compensatory mechanisms. Regardless of why MAPK3 depletion decreases VEGF levels by only 25%, this reduction would be expected to be functionally relevant, since it has previously been demonstrated in mice that a 25–30% reduction in VEGF levels can produce dramatic biological consequences in the nervous system [13]. We conclude that control of VEGF IRES-dependent translation via MAPK3 contributes an important additional regulatory layer for proper maintenance of VEGF protein levels within a biologically optimal range. Accordingly, the IRES-mediated translational control mechanism that we have described here might be useful as a novel target for therapeutic strategies to modulate VEGF levels.

How does MAPK3 promote VEGF IRES translation? Previous work has highlighted a hypoxia-controlled switch from cap-dependent to cap-independent translation in breast cancer that results from an increase in eIF4E-Binding Protein 1 (4E-BP1) activity [18]. Thus, MAPK3 might conceivably regulate VEGF IRES translation via effects on 4E-BP1 activity. We consider this unlikely for several reasons. First, in contrast to MAPK1, which has been shown to phosphorylate 4EBP1 in vitro [36], we are not aware of any evidence that MAPK3 can directly phosphorylate 4E-BP1. MAPK3 could conceivably indirectly promote 4E-BP1 phosphorylation, since it can act through Tsc1/2 as an upstream activator of mTOR, the main kinase known to phosphorylate 4E-BP1 in vivo [37]. However, phosphorylation of 4E-BP1 leads to *reduced* eIF4E binding and therefore reduced inhibition of eIF4E activity. Thus, regardless of whether MAPK3 would act directly or indirectly to promote phosphorylation of 4E-BP1, the prediction in either case would be that silencing of MAPK3 would lead to less phosphorylation of 4E-BP1, with resultant inhibition of cap-dependent translation and stimulation of IRES translation. However, this is not what we observe: we found no effect on cap-dependent translation and a decrease in VEGF IRES function in MAPK3 siRNA-treated cells. We therefore conclude that our screening strategy has uncovered a novel IRES-specific translational stimulatory signaling mechanism mediated by MAPK3. Future work will explore the nature of the underlying signaling cascade in this pathway and how it impinges on the translational machinery to specifically promote VEGF production.

In summary, we report a powerful method to identify novel regulators of mRNA translation. We have applied our screening method here to the VEGF IRES and have thereby identified a novel function for MAPK3 as a specific, positive regulator of VEGF-IRES and VEGF expression. Our approach is readily applicable to other mRNA regulatory elements and should also be straightforward to adapt to other cell types. Indeed, we think the applications of this approach are broad. Areas of clear future interest would be to screen with additional IRESs or other mRNA regulatory motifs implicated in disease, such as upstream open reading frames, microRNA or regulatory protein binding sites [3], as well as alternative polyadenylation sites [38], [39]. More challenging, but equally interesting applications would be to use this method to identify specific translational regulators in other cell types, for example other cancer cell lines, primary cell cultures, stem cells or differentiated cell types obtained from patient-derived induced pluripotent stem cells [40], [41], [42], [43].

## Materials and Methods

### Cell Culture, Cell Extracts, Antibodies, Plasmids and *in vitro* Transcription

HeLa Kyoto cells used in this study [44] were grown, unless elsewhere indicated in 4.5 g/L glucose DMEM, 10% FCS, 1% penicillin/streptomycin, 1% glutamine. Hypoxic studies were performed at 0.7% O_2_ and 5% CO_2_. All experiments were performed with cells in the exponential growth phase at sub-confluent (∼70%) density. For Western Blot analysis, HeLa total cell extracts were prepared in RIPA buffer (10 mM Tris-HCl pH 8.0, 150 mM NaCl, 1 mM EDTA, 1% NP40, 0.1% SDS) containing freshly added Complete™ EDTA-free Protease Inhibitor Cocktail (Roche). Mouse monoclonal antibodies against beta-actin and MAPK3 were purchased from Sigma and Abcam, respectively, and anti-mouse secondary antibody from Amersham. The plasmid encoding firefly luciferase pT3luc(pA) has been described previously [45]. The VEGF lucA construct with an A_98_ tail containing the VEGF 5′UTR (1048 nt) upstream of the firefly luciferase coding sequence cloned in a pBluescript II KS vector was kindly provided by Antje Ostareck-Lederer. The “no-IRES” control was described previously [46]. VEGF lucA templates linearized with Not1 for VEGF IRES mRNAs, no IRES control templates linearized with ECL136II for no IRES control mRNAs or pT3luc(pA) templates linearized with BamH1 for cap mRNAs were used with the T3 MEGAscript kit (Applied Biosystems/Ambion; Austin, TX) and either ApppG cap analogs (NEB; Ipswich, MA) (IRES and no IRES control mRNAs) or 3-O-Me-m^7^G(5)ppp(5)G (cap-mRNAs) to give 80% capping efficiency. RNAs were purified via RNeasy (QIAGEN). mRNA concentration and integrity were assessed by OD measurement and agarose gel electrophoresis.

### Preparation of 96 Well Plates for siRNA Solid-phase Reverse Transfection

21 nt RNA duplexes were obtained from Ambion Europe, Ltd, all with the silencer select modification [24]. The full list of siRNA sequences used in this study is available in the Table S1 and Table S2. Control silencer select siRNA sequences (scrambled, firefly and PLK1) are available in Table S4.

White 96 well plates (Nunclon™ Delta Surface, Nunc) were coated with siRNA transfection solutions using a Microlab STAR pipetting robot (Hamilton), similar as previously described [20]. In detail: siRNA stock solution were prepared by dissolving siRNAs with milliQ water to a final concentration of 3 µM. 3 µl OptiMEM, containing 0.4 M sucrose was transferred to each well of a 384 well low volume plate. 1.75 µl water and 1.75 µl Lipofectamine 2000 was added to each well followed by a 8 times mixing step. 5 µl of the respective siRNA stock solution (3 µM) was added to each well followed again by a 8 times mixing step. After incubation of 30 min at RT 7.25 µl of a 0.2% (w/v) gelatin solution was added and the final solution was mixed 8 times using the slower mixing mode possible on the MICROLAB STAR from Hamilton. Plates were lyophilized in Concentrator System from Genevac called Mivac Quattro (purchased via Fisher Scientific) and stored in plastic boxes containing drying orange heavy metal free (Fluka, catalog number 94098).

### Solid-phase siRNA Reverse Transfection, Reporter mRNA Transfection and Luciferase Assays

HeLa cells resuspended in low glucose medium (1 g/L) were seeded onto siRNA-coated plates (3000 cells/well of a 96 well plate in a volume of 100 µl/well), using a FlexDrop™ IV EXi bulk dispenser (PerkinElmer). After 48 hours of culture under normoxic conditions the medium was removed and the second transfection with reporter mRNA performed using TransMessenger transfection reagent (Qiagen), according to manufacturer’s instructions in serum-free medium. Same molar amounts of reporter mRNA, corresponding to 150 ng of VEGF IRES reporter mRNA and 80 ng of no-IRES reporter mRNA, were transfected per well, with a RNA:enhancer 1∶2 ratio and a RNA:Transmessenger 1∶4 ratio. The transfection-mix (42 µl/well) was transferred immediately after removal of the old medium onto 96 well plates with a Evolution™ P3 pipetting robot (PerkinElmer). After 6 hours incubation under hypoxic conditions firefly luciferase was quantified with the Britelite™ plus Reporter Gene Assay System (PerkinElmer). 40 µl/well BriteLite reagent was added with the FlexDrop™ bulk dispenser and luminescence measured with an EnVision™ HTS plate reader (PerkinElmer).

### Effect of siRNA Transfection on Cell Proliferation

Similarly to the reporter assay, cells were solid-phase reverse transfected and incubated for 48 hours in siRNA-coated plates. Next, medium was replaced with serum-free medium and plates were incubated for an additional 6 hours under low oxygen conditions to induce hypoxia. Cell viability was then monitored using the ATPlite 1step Luminescence Assay System (PerkinElmer). Briefly, 40 µl/well ATPlite reagent was added with the FlexDrop™ IV EXi (PerkinElmer) and the ATP-dependent chemilumiscence was measured as indicator of cell viability read with an EnVision™ HTS plate reader.

### Northern Blotting

Total RNA was extracted from siRNA treated HeLa cells in 96 well plate format using 50 µl/well TRIzol reagent (Invitrogen), according to manufacturer’s instructions. Equivalent amounts of total RNA were loaded on 1% agarose gels containing 1% formaldehyde and this was verified by ethidium bromide staining. RNA was transferred onto positively charged nylon membranes (Roth). Hybridisation was done with a ^32^P-labeled fragment obtained upon digestion of the firefly luciferase coding plasmid previously reported [45] with restriction enzymes NcoI and EclII.

### Gene Annotation

Genes selected in the first screening round were classified with Gene Ontology term annotation. GO terms were assigned to the first suitable of the following function groups: gene expression, tumorigenesis/angiogenesis, cell signaling, cell cycle, other and unknown.

### VEGF ELISA

Studies on endogenous VEGF production and secretion into medium were performed with siRNA reverse transfected cells in a 96 well plate format. Cells were seeded as described above and cultured for 24 hours under normoxic conditions. After a medium exchange cells were further incubated for 24 hours under hypoxic conditions before VEGF concentration in the medium was determined with VEGF Quantikine Colorimetric Sandwich ELISA (R&D Systems) according to the manufacturer’s instructions.

### Reverse Transcription-quantitative PCR (RT-qPCR)

Cell lysis, RNA extraction, DNAse treatment and reverse transcription were performed using the Power SYBR Green Cells-to-C_T_ Kit (Applied Biosystems), according to the manufacturer’s instructions. RT-qPCR analysis of endogenous VEGF mRNAs was performed using the Power SYBR Green Cells-to-C_T_ Kit in a 7500 Real-Time PCR System (Applied Biosystems). Forward and reverse primer sequences used to detect VEGF, MAPK3 and actin β mRNAs are available in Table S5. Transcripts levels in MAPK3 siRNA-treated cells were normalized to the expression levels of beta-actin mRNA and measured relative to those in scrambled siRNA-treated cells as previously described [47]. Standard deviations and statistic analysis was performed with REST© 2009 Software [47].

## Supporting Information

Figure S1 **VEGF IRES activity in HeLa cells and its stimulation by hypoxia**. (a) VEGF IRES is functional in HeLa cells. HeLa cells were transfected with A-capped reporter mRNAs with or without the VEGF IRES element. 6 hours later FLuc protein levels were measured. (b) HeLa cells transfected with VEGF IRES reporter mRNA were incubated either under normoxic or hypoxic conditions for 6 hours before assaying Fluc expression.
(PDF)

Figure S2 **Robustness of Pilot screen**. Pilot screen in 96 well plate format displays very low well-to-well variability. HeLa cells were seeded in four different random positions on three different 96 well plates and reverse transfected with scrambled siRNAs (negative control). 48 hours later they were transfected with the VEGF IRES Fluc reporter mRNA and 6 hours later FLuc reporter expression was measured. Very similar VEGF IRES activity was observed in different wells (indicated on the x axis) and on different plates (standard deviation).
(PDF)

Table S1 **Kinase library.** Gene symbol, RefSeq, sense- and antisense siRNA sequences and normalized luciferase values for the screened 702 kinases.
(PDF)

Table S2 **Phosphatase library.** Gene symbol, RefSeq, sense- and antisense siRNA sequences and normalized luciferase values for the screened 298 phosphatases.
(PDF)

Table S3 **Confirmed IRES specific hits.** Gene symbol, RefSeq, sense- and antisense siRNA sequences for the 3 IRES specific hits.
(PDF)

Table S4 **siRNA control sequences.**
(PDF)

Table S5 **Primer sequences used for qRT-PCR experiments.**
(PDF)
