# Supplementary figures and images for: Automated High-Throughput RNAi Screening in Human Cells Combined with Reporter mRNA Transfection to Identify Novel Regulators of Translation
Source: PLoS One. 2012 Sep 27;7(9):e45943. doi: 10.1371/journal.pone.0045943 (PMC3459937; doi:10.1371/journal.pone.0045943)

# Supplementary Figure 1

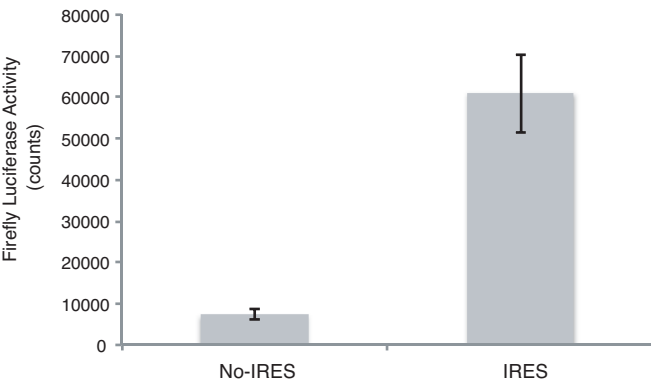

**b**

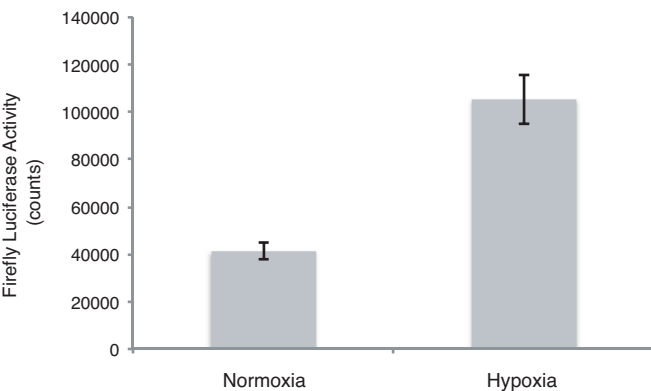

Supplement: Figure S1 — VEGF IRES activity in HeLa cells and its stimulation by hypoxia. (a) VEGF IRES is functional in HeLa cells. HeLa cells were transfected with A-capped reporter mRNAs with or without the VEGF IRES element. 6 hours later FLuc protein levels were measured. (b) HeLa cells transfected with VEGF IRES reporter mRNA were incubated either under normoxic or hypoxic conditions for 6 hours before assaying Fluc expression. (PDF) [file pone.0045943.s001.pdf]

# Supplementary Figure 2

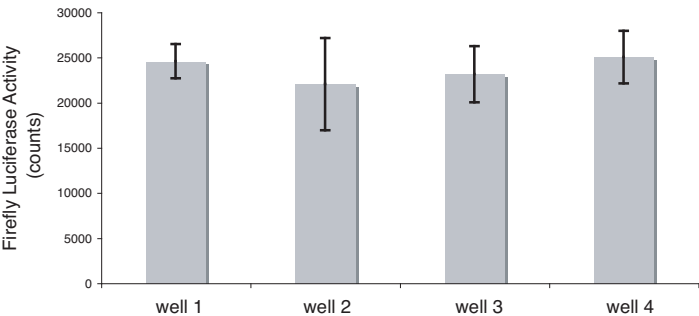

Supplement: Figure S2 — Robustness of Pilot screen. Pilot screen in 96 well plate format displays very low well-to-well variability. HeLa cells were seeded in four different random positions on three different 96 well plates and reverse transfected with scrambled siRNAs (negative control). 48 hours later they were transfected with the VEGF IRES Fluc reporter mRNA and 6 hours later FLuc reporter expression was measured. Very similar VEGF IRES activity was observed in different wells (indicated on the x axis) and on different plates (standard deviation). (PDF) [file pone.0045943.s002.pdf]
