# Supplementary material for: Automated High-Throughput RNAi Screening in Human Cells Combined with Reporter mRNA Transfection to Identify Novel Regulators of Translation
Source: PLoS One. 2012 Sep 27;7(9):e45943. doi: 10.1371/journal.pone.0045943 (PMC3459937; doi:10.1371/journal.pone.0045943)
Supplement: Table S1 — Kinase library. Gene symbol, RefSeq, sense- and antisense siRNA sequences and normalized luciferase values for the screened 702 kinases. (PDF) [file pone.0045943.s003.pdf]

Supplementary table 1

Gene Symbol	RefSeq	Sense siRNA Sequence 5'-3'	Antisense siRNA Sequence 5'-3'	Normalized luciferase values
AAK1	NM_014911	CGUGAGUAGCGGUGAUGUAtt	UACAUCACCGCUACUCACGtt	102
AAK1	NM_014911	CCUCGGACCUCUCAACAAAtt	UUUGUUGAGAGGUCCGAGGag	251
AAK1	NM_014911	GACAAGCAAUGGGAUGAAAtt	UUUCAUCCCAUUGCUUGUCct	104
AATK	NM_001080395	GGUGAAGAUUGGUGACUAUtt	AUAGUCACCAAUCUUCACCgt	106
AATK	NM_001080395	CGGUUCCGCUGAGAUCAGAtt	UCUGAUCUCAGCGGAACCGgg	128
AATK	NM_001080395	GCACCUUCAUCGCAACAAUtt	AUUGUUGCGAUGAAGGUGCag	107
ABL1	NM_007313	GAAGGGAGGGUGUACCAUUtt	AAUGGUACACCCUCCCUUCgt	181
ABL1	NM_007313	CGACAAGUGGGAGAUGGAAtt	UUCCAUCUCCCACUUGUCGta	1024
ABL1	NM_007313	CACUCUAAGCAUAACUAAAtt	UUUAGUUAUGCUUAGAGUGtt	133
ABL2	NM_007314	CCCUGUCCUUAAUAACUUAtt	UAAGUUAUUAAGGACAGGGtt	217
ABL2	NM_007314	CAAGCAUCCUAAUCUGGUAtt	UACCAGAUUAGGAUGCUUGat	109
ABL2	NM_007314	CCCUCAAACUCGCAACAAAtt	UUUGUUGCGAGUUUGAGGGat	109
ACVR1	NM_001105	GAGGCAUGAAAAUAUCUUAtt	UAAGAUAUUUUCAUGCCUCag	322
ACVR1	NM_001105	GGAUCAUUCGUGUACAUCAtt	UGAUGUACACGAAUGAUCCaa	180
ACVR1	NM_001105	GUUGCUCUCCGAAAAUUUAtt	UAAAUUUUCGGAGAGCAACtc	162
ACVR1B	NM_020327	AGUACUUGAUGAAACCAUUtt	AAUGGUUUCAUCAAGUACUtc	426
ACVR1B	NM_020327	GAACCAUCGUUUUACAAGAtt	UCUUGUAAAACGAUGGUUCgg	285
ACVR1B	NM_020327	GAAGCAGAGAUAUACCAGAtt	UCUGGUAUAUCUCUGCUUCcc	275
ACVR1C	NM_145259	GCCUAACUGCUCUUCGUAUtt	AUACGAAGAGCAGUUAGGCgg	146
ACVR1C	NM_145259	CAAUGUUACCAAAACCGAAtt	UUCGGUUUUGGUAACAUUGtt	163
ACVR1C	NM_145259	GGCUCCUUAUAUGACUAUUtt	AAUAGUCAUAUAAGGAGCCct	125
ACVR2A	NM_001616	GCCCAGUUGCUUAACGAAUtt	AUUCGUUAAGCAACUGGGCtt	286
ACVR2A	NM_001616	GGAUGAUAUCAACUGCUAUtt	AUAGCAGUUGAUAUCAUCCag	906
ACVR2A	NM_001616	CAGACUUUCUUAAGGCUAAtt	UUAGCCUUAAGAAAGUCUGat	180
ACVR2B	NM_001106	CAUCAUCACAUGGAACGAAtt	UUCGUUCCAUGUGAUGAUGtt	286
ACVR2B	NM_001106	CCAUCGAGCUCGUGAAGAAtt	UUCUUCACGAGCUCGAUGGtg	126

ACVR2B	NM_001106	GCUCCAACCUCGAAGUAGAtt	UCUACUUCGAGGUUGGAGCct	141
ACVRL1	NM_001077401	CCGAGUUCGUCAACCACUAtt	UAGUGGUUGACGAACUCGGtg	101
ACVRL1	NM_001077401	GGAUCAAGAAGACACUACAtt	UGUAGUGUCUUCUUGAUCCgc	115
ACVRL1	NM_001077401	GAUCCGCACGGACUGCUUUtt	AAAGCAGUCCGUGCGGAUCtg	308
ADCK2	NM_052853	GAAUUCCCGUGGACUUGAAtt	UUCAAGUCCACGGGAAUUCct	98
ADCK2	NM_052853	GACCAGUCGUUUCUAGAAAtt	UUUCUAGAAACGACUGGUCtg	105
ADCK2	NM_052853	GAUUGACCUGCGUUACGAAtt	UUCGUAACGCAGGUCAAUCtg	129
ADCK4	NM_024876	GCUUGUGCCCAGAAUUUCAtt	UGAAAUUCUGGGCACAAGCcg	98
ADCK4	NM_024876	UCACAGGCUUUGAAACCAAtt	UUGGUUUCAAAGCCUGUGAgg	241
ADCK4	NM_024876	GGACCUGCGGAACCAGAUUtt	AAUCUGGUUCCGCAGGUCCtg	304
ADCK5	NM_174922	GGCCUUUGCUGAGCAGAUAtt	UAUCUGCUCAGCAAAGGCCtt	112
ADCK5	NM_174922	GUGCAUGACAUAGCAGAAAtt	UUUCUGCUAUGUCAUGCACtg	136
ADCK5	NM_174922	GCAACGUUCUGGUGCGGAAtt	UUCCGCACCAGAACGUUGCca	170
ADK	NM_006721	GCAAGGCUUUGAGACUAAAtt	UUUAGUCUCAAAGCCUUGCtc	67
ADK	NM_006721	GCAUUGGGAUAGAUAAAUUtt	AAUUUAUCUAUCCCAAUGCat	147
ADK	NM_006721	GCCUUAUGUUGAUAUACUUtt	AAGUAUAUCAACAUAAGGCat	158
ADRBK1	NM_001619	GGAAGAAUGUGGAGCUCAAtt	UUGAGCUCCACAUUCUUCCac	223
ADRBK1	NM_001619	GGGAGAUCUUCGACUCAUAtt	UAUGAGUCGAAGAUCUCCCgg	136
ADRBK1	NM_001619	ACAUCGAAGAGAUUUGUCAtt	UGACAAAUCUCUUCGAUGUat	148
ADRBK2	NM_005160	GCAGCAAGAAGUAACGGAAtt	UUCCGUUACUUCUUGCUGCca	121
ADRBK2	NM_005160	GCAAGCUGUAGAACACGUAtt	UACGUGUUCUACAGCUUGCtt	136
ADRBK2	NM_005160	GCUACUUGAUUGCGACCAAtt	UUGGUCGCAAUCAAGUAGCtt	117
AGK	NM_018238	GCAUUGAACCUGACACCAUtt	AUGGUGUCAGGUUCAAUGCag	194
AGK	NM_018238	CUAUUGUUAAGACAGAUUAtt	UAAUCUGUCUUAACAAUAGtc	134
AGK	NM_018238	GCACUUUGCUUAUCCCGGAtt	UCCGGGAUAAGCAAAGUGCac	232
AK1	NM_000476	GAAUGAAAUCCGAACAGUUtt	AACUGUUCGGAUUUCAUUCgg	199
AK1	NM_000476	CAUUUCCAUUGGUUAUUUAtt	UAAAUAACCAAUGGAAAUGaa	275
AK1	NM_000476	GGUGCUCACGUGUCCUUAAtt	UUAAGGACACGUGAGCACCaa	142
AK2	NM_013411	GCUUGAUUCUGUGAUUGAAtt	UUCAAUCACAGAAUCAAGCtt	112
AK2	NM_013411	GACUCUCUGCUGAUCCGAAtt	UUCGGAUCAGCAGAGAGUCtg	159
AK2	NM_013411	GUCGAUCAGAUGAUAAUGAtt	UCAUUAUCAUCUGAUCGACgg	59

AK3	NM_016282	GGCUUAUGAAGACCAAACAtt	UGUUUGGUCUUCAUAAGCCtt	159
AK3	NM_016282	GCGUGAGGAUGAUAAACCAtt	UGGUUUAUCAUCCUCACGCtg	125
AK3	NM_016282	GGCCCUAUGUAUAUGCUUUtt	AAAGCAUAUACAUAGGGCCaa	429
AK3L1	NM_001005353	GUUGUAAGAUCAUAUCUUAtt	UAAGAUAUGAUCUUACAACat	402
AK3L1	NM_001005353	GAACUGAUAGGAAAACAAAtt	UUUGUUUUCCUAUCAGUUCac	89
AK3L1	NM_001005353	CAUCUUUUCUAGUUGAAAUtt	AUUUCAACUAGAAAAGAUGct	198
AK3L2	NM_001002921	GUCAUUGAAUUAUACAAGAtt	UCUUGUAUAAUUCAAUGACtg	139
AK3L2	NM_001002921	GGAACGGAGACGAACAAAAtt	UUUUGUUCGUCUCCGUUCCgg	112
AK3L2	NM_001002921	GGAGGAUGAUAAACCCGAAtt	UUCGGGUUUAUCAUCCUCCtg	308
AK5	NM_174858	GGAGACUAAUGAACUUCAAtt	UUGAAGUUCAUUAGUCUCCtt	114
AK5	NM_174858	GGCUUGUGCUAAUCAGAGAtt	UCUCUGAUUAGCACAAGCCag	193
AK5	NM_174858	CGAUAUGGAUUCCAAUACAtt	UGUAUUGGAAUCCAUAUCGtt	383
AK7	NM_152327	GGACAUCAAUAUCGACGAUtt	AUCGUCGAUAUUGAUGUCCcg	103
AK7	NM_152327	GAAUAGACUUGCUAUCAAAtt	UUUGAUAGCAAGUCUAUUCtg	91
AK7	NM_152327	GGAUGCUUCGGAUGAGUUUtt	AAACUCAUCCGAAGCAUCCag	76
AKAP12	NM_005100	CCAGGCUAAUGAUAUUGGAtt	UCCAAUAUCAUUAGCCUGGga	114
AKAP12	NM_005100	CCAUGACUGUUGAGGUAGAtt	UCUACCUCAACAGUCAUGGtc	154
AKAP12	NM_005100	CGAAACAGCUGUUACCGUAtt	UACGGUAACAGCUGUUUCGtg	440
AKAP14	NM_001008534	GGAUGAAUUGACUCAAGUAtt	UACUUGAGUCAAUUCAUCCtc	139
AKAP14	NM_001008534	ACAUCAAGUGGAUGACUCAtt	UGAGUCAUCCACUUGAUGUtt	98
AKAP14	NM_001008534	CUGUUAAGAUUGUGGAAGAtt	UCUUCCACAAUCUUAACAGca	148
AKAP7	NM_016377	CGAAGCAGCUGAUCAGAAUtt	AUUCUGAUCAGCUGCUUCGgt	119
AKAP7	NM_016377	AACUAGUAAGGCUCAGUAAtt	UUACUGAGCCUUACUAGUUca	208
AKAP7	NM_016377	GCUGAACUAGUAAGGCUCAtt	UGAGCCUUACUAGUUCAGCgt	426
AKAP8	NM_005858	CGGAAGCAGUUCCAACUUUtt	AAAGUUGGAACUGCUUCCGtt	72
AKAP8	NM_005858	CCGUAGCUUUGAUGACGAAtt	UUCGUCAUCAAAGCUACGGaa	99
AKAP8	NM_005858	GCAGUACAGUGAAUGCCGAtt	UCGGCAUUCACUGUACUGCcc	151
AKAP8L	NM_014371	GGAACACUUUAAGUACGUAtt	UACGUACUUAAAGUGUUCCtt	78
AKAP8L	NM_014371	CCAUGGAUCACAACCGGAAtt	UUCCGGUUGUGAUCCAUGGtc	191
AKAP8L	NM_014371	CAGUCGACAUACUCGGAUAtt	UAUCCGAGUAUGUCGACUGca	373
AKT1	NM_001014431	GCGUGACCAUGAACGAGUUtt	AACUCGUUCAUGGUCACGCgg	72

AKT1	NM_001014431	GAACAAUCCGAUUCACGUAtt	UACGUGAAUCGGAUUGUUCtg	287
AKT1	NM_001014431	CGGUAGCACUUGACCUUUUtt	AAAAGGUCAAGUGCUACCGtg	84
AKT2	NM_001626	CAACUUCUCCGUAGCAGAAtt	UUCUGCUACGGAGAAGUUGtt	108
AKT2	NM_001626	CGGGCUAAAGUGACCAUGAtt	UCAUGGUCACUUUAGCCCGtg	343
AKT2	NM_001626	UGACUUCGACUAUCUCAAAtt	UUUGAGAUAGUCGAAGUCAtt	98
AKT3	NM_181690	GUAACAUCUGAGACAGAUAtt	UAUCUGUCUCAGAUGUUACtt	50
AKT3	NM_181690	GGACUAUCUACAUUCCGGAtt	UCCGGAAUGUAGAUAGUCCaa	102
AKT3	NM_181690	GCUCAUUCAUAGGAUAUAAtt	UUAUAUCCUAUGAAUGAGCca	220
ALDH18A1	NM_002860	GCGUGAUGAGAUCCUGUUAtt	UAACAGGAUCUCAUCACGCtg	239
ALDH18A1	NM_002860	GGAAUUAUGCAUUGAAGUAtt	UACUUCAAUGCAUAAUUCCag	111
ALDH18A1	NM_002860	CGGAUGUCAUCGUCACAGAtt	UCUGUGACGAUGACAUCCGtg	64
ALK	NM_004304	CAUGCUCUAUUGCUCAGUAtt	UACUGAGCAAUAGAGCAUGgt	108
ALK	NM_004304	CUGUUUGAGAGAAACCCAAtt	UUGGGUUUCUCUCAAACAGgt	119
ALK	NM_004304	CAAACCAGUUAAUCCAGAAtt	UUCUGGAUUAACUGGUUUGta	128
ALPK1	NM_025144	GGAAGUGAAUUAUCACGUUtt	AACGUGAUAAUUCACUUCCtc	127
ALPK1	NM_025144	GAGAUGUUGUGGUCGAUUUtt	AAAUCGACCACAACAUCUCta	150
ALPK1	NM_025144	CCAUGAGCAAGAACGAUUAtt	UAAUCGUUCUUGCUCAUGGaa	128
ALPK2	NM_052947	CAUCGGCCUGAGAACAAUAtt	UAUUGUUCUCAGGCCGAUGga	77
ALPK2	NM_052947	CCAUGACCUUCAUUGAUCAtt	UGAUCAAUGAAGGUCAUGGaa	72
ALPK2	NM_052947	GGGCUGUACCUGAUAGUCUtt	AGACUAUCAGGUACAGCCCag	71
ALPK3	NM_020778	GACUAGGCCUUUCAACAGAtt	UCUGUUGAAAGGCCUAGUCgg	138
ALPK3	NM_020778	GGUACAAGGAUGAUACGGAtt	UCCGUAUCAUCCUUGUACCag	261
ALPK3	NM_020778	CCAUGGAUAUGGAAACCCAtt	UGGGUUUCCAUAUCCAUGGgt	114
ALS2CR2	NM_018571	GCUUUACAGAAAGCCGUGAtt	UCACGGCUUUCUGUAAAGCtt	263
ALS2CR2	NM_018571	CCUCAAUCAGAAUCCAGAAtt	UUCUGGAUUCUGAUUGAGGga	216
ALS2CR2	NM_018571	GCCCAUUGGAUAUCAGUAUtt	AUACUGAUAUCCAAUGGGCta	279
ALS2CR7	NM_139158	GAGUCCCAUUUACAGCUAUtt	AUAGCUGUAAAUGGGACUCct	183
ALS2CR7	NM_139158	GCUCUUAUGCGACAGUUUAtt	UAAACUGUCGCAUAAGAGCct	283
ALS2CR7	NM_139158	CCUGAAACCUCAGAACUUAtt	UAAGUUCUGAGGUUUCAGGtc	45
AMHR2	NM_020547	CGACCACAUUGUCCGAUUUtt	AAAUCGGACAAUGUGGUCGtg	214
AMHR2	NM_020547	GAAUGUGCUCAUUCGGGAAtt	UUCCCGAAUGAGCACAUUCtg	294

AMHR2	NM_020547	CUGGGAGAGCUGCUAGAUAtt	UAUCUAGCAGCUCUCCCAGtg	70
ANKK1	NM_178510	AGCACAUCGUGUCUAUCUAtt	UAGAUAGACACGAUGUGCUga	160
ANKK1	NM_178510	GCAAAUACCUGAUCUGCAAtt	UUGCAGAUCAGGUAUUUGCcc	124
ANKK1	NM_178510	GGUUCCGCAUCAUCCAUGAtt	UCAUGGAUGAUGCGGAACCtg	145
ARAF	NM_001654	CGAGAUCUCAAGUCUAACAtt	UGUUAGACUUGAGAUCUCGgt	1122
ARAF	NM_001654	GUGUUGACAUGAGUACCAAtt	UUGGUACUCAUGUCAACACag	149
ARAF	NM_001654	UGCACAAUUUUGUACGGAAtt	UUCCGUACAAAAUUGUGCAtg	159
ASB10	NM_080871	CAACAUCCGUGCUCUGAGAtt	UCUCAGAGCACGGAUGUUGaa	92
ASB10	NM_080871	GGUUUGGAGCGAGAGUGGAtt	UCCACUCUCGCUCCAAACCtg	67
ASB10	NM_080871	CAACAUCGCUGACCAGGAUtt	AUCCUGGUCAGCGAUGUUGgg	104
ASCIZ	NM_015251	CGGACUUCUUACUCGCAGAtt	UCUGCGAGUAAGAAGUCCGtt	95
ASCIZ	NM_015251	GAAUCGAUUUUGAUAUCGAtt	UCGAUAUCAAAAUCGAUUCcg	165
ASCIZ	NM_015251	GGAUAGAAAGUCCAACGGAtt	UCCGUUGGACUUUCUAUCCca	344
ATM	NM_138292	AUAUAUCACCUGUUUGUUAtt	UAACAAACAGGUGAUAUAUtt	239
ATM	NM_138292	GCAAUUGUCAUAAAACCAAtt	UUGGUUUUAUGACAAUUGCtg	294
ATM	NM_138292	GCUGUUACCUGUUUGAAAAtt	UUUUCAAACAGGUAACAGCtg	149
ATR	NM_001184	GAGCCGAUUUUUAAGUCAAtt	UUGACUUAAAAAUCGGCUCat	182
ATR	NM_001184	GAUGAGUAUGCAAAAUUUAtt	UAAAUUUUGCAUACUCAUCaa	218
ATR	NM_001184	UUGUAGAAAUGGAUACUGAtt	UCAGUAUCCAUUUCUACAAgg	145
AURKA	NM_003600	GCGCAUUCCUUUGCAAGCAtt	UGCUUGCAAAGGAAUGCGCtg	155
AURKA	NM_003600	GAGUCUACCUAAUUCUGGAtt	UCCAGAAUUAGGUAGACUCtg	167
AURKA	NM_003600	GGAUCAGCUGGAGAGCUUAtt	UAAGCUCUCCAGCUGAUCCaa	57
AURKAIP1	NM_017900	GCAGAUCAAGUUCGAGAAAtt	UUUCUCGAACUUGAUCUGCtt	191
AURKAIP1	NM_017900	CCACCGCAAUCCUACCAGUtt	ACUGGUAGGAUUGCGGUGGag	82
AURKAIP1	NM_017900	AGAUCAAGUUCGAGAAAGAtt	UCUUUCUCGAACUUGAUCUgc	37
AURKB	NM_004217	CCUGCGUCUCUACAACUAUtt	AUAGUUGUAGAGACGCAGGat	78
AURKB	NM_004217	UCGUCAAGGUGGACCUAAAtt	UUUAGGUCCACCUUGACGAtg	201
AURKB	NM_004217	GCAAGUUUGGAAACGUGUAtt	UACACGUUUCCAAACUUGCct	460
AURKC	NM_003160	CCUGCGCCUGUAUAACUAUtt	AUAGUUAUACAGGCGCAGGat	105
AURKC	NM_003160	GGGUGUACCUGAUUCUGGAtt	UCCAGAAUCAGGUACACCCgg	105
AURKC	NM_003160	GUGAUUCACAGAGAUAUUAtt	UAAUAUCUCUGUGAAUCACtt	285

AXL	NM_021913	GGAACUGCAUGCUGAAUGAtt	UCAUUCAGCAUGCAGUUCCtg	166
AXL	NM_021913	GGGUGGAGGUUAUCCUGAAtt	UUCAGGAUAACCUCCACCCtc	304
AXL	NM_021913	CAGCGAGAUUUAUGACUAUtt	AUAGUCAUAAAUCUCGCUGtt	151
BCKDK	NM_005881	ACAAUGAUGUCGAUCUGAUtt	AUCAGAUCGACAUCAUUGUtg	60
BCKDK	NM_005881	AGACCGUCACCUCCUUUUAtt	UAAAAGGAGGUGACGGUCUtg	114
BCKDK	NM_005881	CGCUACUUCUUGGACAAGAtt	UCUUGUCCAAGAAGUAGCGga	202
BLK	NM_001715	GCUCCUUUCUUAUCAGAGAtt	UCUCUGAUAAGAAAGGAGCcg	149
BLK	NM_001715	UCUACGCAGUGGUCACCAAtt	UUGGUGACCACUGCGUAGAgt	197
BLK	NM_001715	UGAUGGAAGUUGUCACUUAtt	UAAGUGACAACUUCCAUCAgg	93
BMP2K	NM_198892	GAAUGAUGGUGGGAACUAUtt	AUAGUUCCCACCAUCAUUCaa	153
BMP2K	NM_198892	CGAUGUGCAUUGAAGCGAAtt	UUCGCUUCAAUGCACAUCGga	213
BMP2K	NM_198892	GCUUGAACCAGAUCCGGAAtt	UUCCGGAUCUGGUUCAAGCat	141
BMP2KL	XM_936694	UAAUGAUAAUGAUACCGAAtt	UUCGGUAUCAUUAUCAUUAaa	205
BMP2KL	XM_936694	GCAUAGAUAUAUUUGACUUtt	AAGUCAAAUAUAUCUAUGCtt	137
BMP2KL	XM_936694	AACCCAAGAUAUUAAUACAtt	UGUAUUAAUAUCUUGGGUUgg	209
BMPR1A	NM_004329	GGCUCGUCGUUGUAUCACAtt	UGUGAUACAACGACGAGCCat	161
BMPR1A	NM_004329	GGCCGAUAUGGAGAAGUAUtt	AUACUUCUCCAUAUCGGCCtt	285
BMPR1A	NM_004329	GAAUCUGGAUAGUAUGCUUtt	AAGCAUACUAUCCAGAUUCtg	158
BMPR1B	NM_001203	GGACGAGAGCUUGAACAGAtt	UCUGUUCAAGCUCUCGUCCaa	148
BMPR1B	NM_001203	GAAGUUGACAUACCACCUAtt	UAGGUGGUAUGUCAACUUCat	127
BMPR1B	NM_001203	CACUUCCAGUCUUACAUCAtt	UGAUGUAAGACUGGAAGUGat	101
BMPR2	NM_001204	GAACGGCUAUGUGCGUUUAtt	UAAACGCACAUAGCCGUUCtt	145
BMPR2	NM_001204	CCGAAAUUCAAUUAACUAUtt	AUAGUUAAUUGAAUUUCGGtt	261
BMPR2	NM_001204	GGACAAUAUUAUGCUCGAAtt	UUCGAGCAUAAUAUUGUCCca	245
BMX	NM_001721	GUACCACUCUAGCCCAAUAtt	UAUUGGGCUAGAGUGGUACtt	150
BMX	NM_001721	GUACCAGUCUAGCGCAAUAtt	UAUUGCGCUAGACUGGUACtt	59
BMX	NM_001721	CAGACUAUGAUGAAACUCAtt	UGAGUUUCAUCAUAGUCUGgg	101
BRAF	NM_004333	CAGAGGAUUUUAGUCUAUAtt	UAUAGACUAAAAUCCUCUGtt	164
BRAF	NM_004333	GCAUAAUCCACCAUCAAUAtt	UAUUGAUGGUGGAUUAUGCtc	77
BRAF	NM_004333	CAGUUGUCUGGAUCCAUUUtt	AAAUGGAUCCAGACAACUGtt	210
BRDG1	NM_012108	GGCAAGUAAUUAAACUGCAtt	UGCAGUUUAAUUACUUGCCca	167

BRDG1	NM_012108	GCCUGGUAGUGACAGUAGAtt	UCUACUGUCACUACCAGGCct	289
BRDG1	NM_012108	CACCCUUGUUUUGCCGAAAtt	UUUCGGCAAAACAAGGGUGaa	275
BRSK1	NM_032430	AAAUAUUCCUCGUGCUAAAtt	UUUAGCACGAGGAAUAUUUgt	132
BRSK1	NM_032430	AGACAGGGCUGGUUAAACUtt	AGUUUAACCAGCCCUGUCUgt	79
BRSK1	NM_032430	GAAACAUCCUUGGUACCUAtt	UAGGUACCAAGGAUGUUUCtg	93
BRSK2	NM_003957	CGGAAAGAAAGGUACCCGAtt	UCGGGUACCUUUCUUUCCGgt	202
BRSK2	NM_003957	GCACUUGUCAGACACCACUtt	AGUGGUGUCUGACAAGUGCtg	203
BRSK2	NM_003957	AGAAUGAGCCCGAACCAGAtt	UCUGGUUCGGGCUCAUUCUtg	305
BTK	NM_000061	GAAACUGUUUGGUAAACGAtt	UCGUUUACCAAACAGUUUCga	158
BTK	NM_000061	CACCAGCUCAAAAACGUAAtt	UUACGUUUUUGAGCUGGUGaa	109
BTK	NM_000061	CAGUAAGAAGGGUUCAAUAtt	UAUUGAACCCUUCUUACUGcc	0
BUB1	NM_004336	GAGUCAAAUAUGGAACGAAtt	UUCGUUCCAUAUUUGACUCtt	251
BUB1	NM_004336	GGCCCUACGUAAUAGGCUAtt	UAGCCUAUUACGUAGGGCCct	81
BUB1	NM_004336	GGAUUACCACAGCCUAAAAtt	UUUUAGGCUGUGGUAAUCCat	111
BUB1B	NM_001211	GGAUUACUGCAUUAAACGAtt	UCGUUUAAUGCAGUAAUCCtc	204
BUB1B	NM_001211	CCCUAUGAUUGUAACAAGAtt	UCUUGUUACAAUCAUAGGGat	186
BUB1B	NM_001211	GCACACUAGCUGAACUAAAtt	UUUAGUUCAGCUAGUGUGCtt	155
C19orf35	NM_198532	CGCUGAUCAAUGUCUCUCUtt	AGAGAGACAUUGAUCAGCGgg	109
C19orf35	NM_198532	CUGCCCAAGAAGAUCCUAAtt	UUAGGAUCUUCUUGGGCAGgg	102
C19orf35	NM_198532	GCCCACGUAUAGCAACCUUtt	AAGGUUGCUAUACGUGGGCtg	156
C1orf57	NM_032324	AAAACAACAUUGAUCCAUAtt	UAUGGAUCAAUGUUGUUUUtc	110
C1orf57	NM_032324	GAUUCGAUGUCGUCACGUUtt	AACGUGACGACAUCGAAUCct	77
C1orf57	NM_032324	UGAUGGAUUUUAUACCGAAtt	UUCGGUAUAAAAUCCAUCAac	107
C21orf7	NM_020152	CCAUGGAGGUGUUCAAACAtt	UGUUUGAACACCUCCAUGGat	110
C21orf7	NM_020152	GGAUUACAGUAUUUCAGUUtt	AACUGAAAUACUGUAAUCCaa	121
C21orf7	NM_020152	AGUUAGAUCAGGCAGAAAAtt	UUUUCUGCCUGAUCUAACUtg	64
C9orf95	NM_017881	GACUAUUCCAUAUGAAGAAtt	UUCUUCAUAUGGAAUAGUCag	54
C9orf95	NM_017881	AGAUAGAGACAGAUAAAAAtt	UUUUUAUCUGUCUCUAUCUca	102
C9orf95	NM_017881	GACACUAUAUGGAAUAGAAtt	UUCUAUUCCAUAUAGUGUCaa	110
C9orf96	NM_153710	CGACCAUGGAGCUACAUGAtt	UCAUGUAGCUCCAUGGUCGtg	98
C9orf96	NM_153710	AGAAAAUCAUUGACUCUGAtt	UCAGAGUCAAUGAUUUUCUtt	75

C9orf96	NM_153710	GCAUUUUAGAGGUCAUGCAtt	UGCAUGACCUCUAAAAUGCtg	226
C9orf98	NM_152572	CGGUCCUGAUCGAGAGAAAtt	UUUCUCUCGAUCAGGACCGtg	122
C9orf98	NM_152572	UGACCUAUGUCCAAAGCAAtt	UUGCUUUGGACAUAGGUCAga	71
C9orf98	NM_152572	UGGAGAACCUGAUCUUAAAtt	UUUAAGAUCAGGUUCUCCAgg	106
CALM1	NM_006888	AGGCAUUCCGAGUCUUUGAtt	UCAAAGACUCGGAAUGCCUca	137
CALM1	NM_006888	UGACAAACUUAGGAGAAAAtt	UUUUCUCCUAAGUUUGUCAtg	57
CALM1	NM_006888	GCCUUCUCCCUAUUUGAUAtt	UAUCAAAUAGGGAGAAGGCtt	194
CALM2	NM_001743	AAAGGAAUUGGGAACUGUAtt	UACAGUUCCCAAUUCCUUUgt	72
CALM2	NM_001743	GCACAAUUGACUUCCCUGAtt	UCAGGGAAGUCAAUUGUGCca	122
CALM2	NM_001743	CAAAGAAGCUUUUUCACUAtt	UAGUGAAAAAGCUUCUUUGaa	122
CALM3	NM_005184	AGAUGAUCAGGGAGGCUGAtt	UCAGCCUCCCUGAUCAUCUca	203
CALM3	NM_005184	GACUUCCCGGAGUUCCUGAtt	UCAGGAACUCCGGGAAGUCaa	141
CALM3	NM_005184	CGGAGUUCCUGACCAUGAUtt	AUCAUGGUCAGGAACUCCGgg	154
CAMK1	NM_003656	AUACAGCUCUAGAUAAGAAtt	UUCUUAUCUAGAGCUGUAUct	112
CAMK1	NM_003656	AGAUUUUGAAGGCCGAGUAtt	UACUCGGCCUUCAAAAUCUgt	242
CAMK1	NM_003656	CCAUAGGUGUCAUCGCCUAtt	UAGGCGAUGACACCUAUGGac	375
CAMK1D	NM_020397	GCUGUGAAGUGUAUCCCUAtt	UAGGGAUACACUUCACAGCaa	183
CAMK1D	NM_020397	CCGAAGUGGUUUUAGCUGAtt	UCAGCUAAAACCACUUCGGaa	327
CAMK1D	NM_020397	CCAUCGGAGUGAUUGCCUAtt	UAGGCAAUCACUCCGAUGGac	210
CAMK1G	NM_020439	GAGUCUAAGCUUUUCGAGAtt	UCUCGAAAAGCUUAGACUCcg	171
CAMK1G	NM_020439	GGAGGGCUACUAUGAGUUUtt	AAACUCAUAGUAGCCCUCCtt	261
CAMK1G	NM_020439	GAAACGGAGUCUAAGCUUUtt	AAAGCUUAGACUCCGUUUCtt	221
CAMK2A	NM_015981	ACAUCGUCCGACUACAUGAtt	UCAUGUAGUCGGACGAUGUtg	142
CAMK2A	NM_015981	GGACUUCCAUCGAUUCUAUtt	AUAGAAUCGAUGGAAGUCCag	131
CAMK2A	NM_015981	GGAGAUUUUGAGUCCUACAtt	UGUAGGACUCAAAAUCUCCat	142
CAMK2B	NM_001220	GAGUGUCUGAAAAAGUUCAtt	UGAACUUUUUCAGACACUCca	137
CAMK2B	NM_001220	CGCAGUACAUUGACGGGCAtt	UGCCCGUCAAUGUACUGCGtg	130
CAMK2B	NM_001220	CGUUCUCCAUUGUCACCAAtt	UUGGUGACAAUGGAGAACGgc	126
CAMK2D	NM_172115	GCAUAGCAUAUAUUAGGCUtt	AGCCUAAUAUAUGCUAUGCag	172
CAMK2D	NM_172115	GCGACUUCAUGAUAGCAUAtt	UAUGCUAUCAUGAAGUCGCac	92
CAMK2D	NM_172115	GGGAUGGAUUUUCACCGAUtt	AUCGGUGAAAAUCCAUCCCtt	276

CAMK2G	NM_172170	CGAAAACAGGAGAUCAUUAtt	UAAUGAUCUCCUGUUUUCGca	131
CAMK2G	NM_172170	ACCAGAAACUAGAACGUGAtt	UCACGUUCUAGUUUCUGGUga	111
CAMK2G	NM_172170	GAUCAUUAAGAUUACAGAAtt	UUCUGUAAUCUUAAUGAUCtc	94
CAMK2N1	NM_018584	GAAUGAUACAUAGAAAAGAtt	UCUUUUCUAUGUAUCAUUCct	171
CAMK2N1	NM_018584	GGAGCAAGCGGGUUGUUAUtt	AUAACAACCCGCUUGCUCCgg	384
CAMK2N1	NM_018584	CUUUAGAAGUUAUCAGGAAtt	UUCCUGAUAACUUCUAAAGat	305
CAMK4	NM_001744	CUCUCAAAGUGUUAAAGAAtt	UUCUUUAACACUUUGAGAGca	169
CAMK4	NM_001744	CGUAAGAACUGAGAUAGGAtt	UCCUAUCUCAGUUCUUACGat	163
CAMK4	NM_001744	GCAGAUGCCGUUAAACAAAtt	UUUGUUUAACGGCAUCUGCag	97
CAMKK1	NM_172206	GGAGGAGGUUAAGAACUCAtt	UGAGUUCUUAACCUCCUCCtc	135
CAMKK1	NM_172206	GGCCUACAACGAAAGUGAAtt	UUCACUUUCGUUGUAGGCCag	68
CAMKK1	NM_172206	CUGAAGAUGUUAGACAAGAtt	UCUUGUCUAACAUCUUCAGga	99
CAMKK2	NM_172215	GGCACAUCAAGAUCGCUGAtt	UCAGCGAUCUUGAUGUGCCca	241
CAMKK2	NM_172215	GCAUCGAGUACUUACACUAtt	UAGUGUAAGUACUCGAUGCct	144
CAMKK2	NM_172215	CAAAGGCAUCGAGUACUUAtt	UAAGUACUCGAUGCCUUUGat	405
CAMKV	NM_024046	AGAUGAUUAUGAGAACCAUtt	AUGGUUCUCAUAAUCAUCUtc	144
CAMKV	NM_024046	CGGAGGUGACUGACAGAUAtt	UAUCUGUCAGUCACCUCCGat	196
CAMKV	NM_024046	CCAUAUUGGGAUGAUAUUUtt	AAAUAUCAUCCCAAUAUGGag	351
CARKL	NM_013276	GCACAGACUCCAGACCCUAtt	UAGGGUCUGGAGUCUGUGCag	34
CARKL	NM_013276	GGAGCAGGAUGUGAGUAGAtt	UCUACUCACAUCCUGCUCCcg	287
CARKL	NM_013276	GGAGUCGUGUUUUGGAAAAtt	UUUUCCAAAACACGACUCCat	257
CASK	NM_003688	GAGUUCGGCUGGUACAGUUtt	AACUGUACCAGCCGAACUCtg	148
CASK	NM_003688	GCUUUACAUGGUUUUCGAAtt	UUCGAAAACCAUGUAAAGCat	233
CASK	NM_003688	GGACGACAGAUCUAUGUAAtt	UUACAUAGAUCUGUCGUCCtt	450
CCRK	NM_012119	AGUAUGUGGUACAACUGAAtt	UUCAGUUGUACCACAUACUga	125
CCRK	NM_012119	GAACGAUAUUGAACAGCUUtt	AAGCUGUUCAAUAUCGUUCtt	217
CCRK	NM_012119	GCCUUUGAGUUCAUGCUGUtt	ACAGCAUGAACUCAAAGGCca	819
CD2	NM_001767	CCCACAGAGUAGCUACUGAtt	UCAGUAGCUACUCUGUGGGct	188
CD2	NM_001767	GGUCUGGACAUCUAUCUCAtt	UGAGAUAGAUGUCCAGACCtt	187
CD2	NM_001767	GGACAUCUAUCUCAUCAUUtt	AAUGAUGAGAUAGAUGUCCag	0
CDC2	NM_001786	GAACUUCGUCAUCCAAAUAtt	UAUUUGGAUGACGAAGUUCct	255

CDC2	NM_001786	GGUUAUAUCUCAUCUUUGAtt	UCAAAGAUGAGAUAUAACCtg	104
CDC2	NM_001786	GAAUCUUUACAGGACUAUAtt	UAUAGUCCUGUAAAGAUUCca	155
CDC2L1	NM_033492	CAGUCUGCCGAAGAAGUAAtt	UUACUUCUUCGGCAGACUGct	201
CDC2L1	NM_033492	AAAUCGAUCAGAUCAACAAtt	UUGUUGAUCUGAUCGAUUUct	88
CDC2L1	NM_033492	AGAUCUACAUCGUGAUGAAtt	UUCAUCACGAUGUAGAUCUtg	42
CDC2L5	NM_031267	GGAACUUGCACAACUAGAAtt	UUCUAGUUGUGCAAGUUCCtg	80
CDC2L5	NM_031267	GCUGAUAGCUUACGAGGAAtt	UUCCUCGUAAGCUAUCAGCtt	127
CDC2L5	NM_031267	CAGAUUGUCUAGAUCCAGAtt	UCUGGAUCUAGACAAUCUGtg	129
CDC2L6	NM_015076	GGAUUUGUUUGAGUACGAAtt	UUCGUACUCAAACAAAUCCtc	203
CDC2L6	NM_015076	GGUCAAGCCUGACAGCAAAtt	UUUGCUGUCAGGCUUGACCtt	160
CDC2L6	NM_015076	GAUUCUUGAUGGUAUCCAUtt	AUGGAUACCAUCAAGAAUCtg	265
CDC42BPA	NM_003607	CCGCAAUCAUAGAUCAUGAtt	UCAUGAUCUAUGAUUGCGGct	123
CDC42BPA	NM_003607	CCAUAUCUCUCGGUGUACAtt	UGUACACCGAGAGAUAUGGtg	188
CDC42BPA	NM_003607	GAUGGAAGAUGGAACGGUUtt	AACCGUUCCAUCUUCCAUCag	141
CDC42BPB	NM_006035	CGAGAACGGCAUAACGAGAtt	UCUCGUUAUGCCGUUCUCGct	122
CDC42BPB	NM_006035	CACUCAACUCCAUCGAAUAtt	UAUUCGAUGGAGUUGAGUGtt	185
CDC42BPB	NM_006035	GGCUGAUCCUUUGCUAUGAtt	UCAUAGCAAAGGAUCAGCCgt	172
CDC42BPG	NM_017525	GCAAGAUCAUGAACCACGAtt	UCGUGGUUCAUGAUCUUGCcg	199
CDC42BPG	NM_017525	GGAUGUGAACGGGCACAUUtt	AAUGUGCCCGUUCACAUCCag	452
CDC42BPG	NM_017525	CAAACUCCCUGAUUCCCUUtt	AAGGGAAUCAGGGAGUUUGag	416
CDC42SE2	NM_001038702	CAGUGGAAUGAAUUCAGUUtt	AACUGAAUUCAUUCCACUGaa	225
CDC42SE2	NM_001038702	CCCACAAACUUUGUGCAUAtt	UAUGCACAAAGUUUGUGGGct	339
CDC42SE2	NM_001038702	GGCGGAUUGACAGAAGUAUtt	AUACUUCUGUCAAUCCGCCgt	226
CDC7	NM_003503	GGCUAUUUCUACGAAAGUUtt	AACUUUCGUAGAAAUAGCCtt	146
CDC7	NM_003503	GUACGGGAAUAUAUGCUUAtt	UAAGCAUAUAUUCCCGUACtt	183
CDC7	NM_003503	GCAUUGUUUUGAUGAGUAUtt	AUACUCAUCAAAACAAUGCtc	188
CDK10	NM_003674	GUUUCCAACUUGCUCAUGAtt	UCAUGAGCAAGUUGGAAACct	655
CDK10	NM_003674	GGCCUAUGGUGUCCCAGUAtt	UACUGGGACACCAUAGGCCcg	283
CDK10	NM_003674	AGAUCGACUUGAUCGUGCAtt	UGCACGAUCAAGUCGAUCUgg	157
CDK2	NM_001798	CGGAGCUUGUUAUCGCAAAtt	UUUGCGAUAACAAGCUCCGtc	153
CDK2	NM_001798	GAGUCCCUGUUCGUACUUAtt	UAAGUACGAACAGGGACUCca	282

CDK2	NM_001798	CAAGAUCUCAAGAAAUUCAtt	UGAAUUUCUUGAGAUCUUGgt	74
CDK3	NM_001258	CCAGCUCUUUCGUAUCUUUtt	AAAGAUACGAAAGAGCUGGtc	289
CDK3	NM_001258	GAGCAUUGGUUGCAUCUUUtt	AAAGAUGCAACCAAUGCUCca	97
CDK3	NM_001258	AGCUCUAUCUGGUGUUUGAtt	UCAAACACCAGAUAGAGCUtc	151
CDK4	NM_000075	UGCUGACUUUUAACCCACAtt	UGUGGGUUAAAAGUCAGCAtt	169
CDK4	NM_000075	GGCUUUUGAGCAUCCCAAUtt	AUUGGGAUGCUCAAAAGCCtc	120
CDK4	NM_000075	CACCCGUGGUUGUUACACUtt	AGUGUAACAACCACGGGUGta	345
CDK5	NM_004935	GCAAUGAUGUCGAUGACCAtt	UGGUCAUCGACAUCAUUGCcg	76
CDK5	NM_004935	CCUCGAUCCUGAGAUUGUAtt	UACAAUCUCAGGAUCGAGGtc	254
CDK5	NM_004935	CGGGAGAUCUGCCUACUCAtt	UGAGUAGGCAGAUCUCCCGga	258
CDK6	NM_001259	GUUUGUAACAGAUAUCGAUtt	AUCGAUAUCUGUUACAAACtt	400
CDK6	NM_001259	GGAUAUGAUGUUUCAGCUUtt	AAGCUGAAACAUCAUAUCCtt	137
CDK6	NM_001259	GCAGAAAUGUUUCGUAGAAtt	UUCUACGAAACAUUUCUGCaa	145
CDK7	NM_001799	CAACAUUGGAUCCUACAUAtt	UAUGUAGGAUCCAAUGUUGat	202
CDK7	NM_001799	CCUUAAAGGAGCAAUCAAAtt	UUUGAUUGCUCCUUUAAGGtt	158
CDK7	NM_001799	GGACAUAGAUCAGAAGCUAtt	UAGCUUCUGAUCUAUGUCCaa	76
CDK8	NM_001260	CAAAACUAGUAAUCCUUAUtt	AUAAGGAUUACUAGUUUUGat	277
CDK8	NM_001260	CCCUUACCCAAAACGAGAAtt	UUCUCGUUUUGGGUAAGGGat	173
CDK8	NM_001260	GGCUAUAGGGUGUAUAUUUtt	AAAUAUACACCCUAUAGCCca	84
CDK9	NM_001261	UGAGAUUUGUCGAACCAAAtt	UUUGGUUCGACAAAUCUCAat	157
CDK9	NM_001261	CCGCUGCAAGGGUAGUAUAtt	UAUACUACCCUUGCAGCGGtt	161
CDK9	NM_001261	GAAGUUUCCAAAUACGAGAtt	UCUCGUAUUUGGAAACUUCat	243
CDKL1	NM_004196	GUACUUCAGUGGAGUGAAAtt	UUUCACUCCACUGAAGUACtg	116
CDKL1	NM_004196	GGACCGAGUGACUACUAUAtt	UAUAGUAGUCACUCGGUCCag	209
CDKL1	NM_004196	CGAAUGCUCAAGCAACUCAtt	UGAGUUGCUUGAGCAUUCGga	171
CDKL2	NM_003948	GGUUGGUGAUGUCAAGUAUtt	AUACUUGACAUCACCAACCaa	144
CDKL2	NM_003948	GUGUUUAGGUAAUCUAAUUtt	AAUUAGAUUACCUAAACACat	218
CDKL2	NM_003948	GAUCAGCUAUAUCAUAUUAtt	UAAUAUGAUAUAGCUGAUCaa	447
CDKL3	NM_016508	CUAUCAAUCCCAGCACUAAtt	UUAGUGCUGGGAUUGAUAGgg	198
CDKL3	NM_016508	GGAUAUCAUCUAGUGAUCUtt	AGAUCACUAGAUGAUAUCCtg	116
CDKL3	NM_016508	CUAUCUUCCUAGUAGUUCUtt	AGAACUACUAGGAAGAUAGgg	171

CDKL4	NM_001009565	GUAUGUUGAAGCAAUUAAAtt	UUUAAUUGCUUCAACAUACgt	100
CDKL4	NM_001009565	GAAUCCAGAUGACAGAUUAtt	UAAUCUGUCAUCUGGAUUCat	58
CDKL4	NM_001009565	CGAUUAUGUAGCUACGAGAtt	UCUCGUAGCUACAUAAUCGgt	127
CDKL5	NM_001037343	GAGUCGGCAUAGCUAUAUUtt	AAUAUAGCUAUGCCGACUCtg	109
CDKL5	NM_001037343	CUAUGUAUGUGACCCGUGAtt	UCACGGGUCACAUACAUAGaa	133
CDKL5	NM_001037343	GAAUGAUAUUGUCCAUCGAtt	UCGAUGGACAAUAUCAUUCtt	149
CERK	NM_022766	GUCAAAUACCAAUGCUUUAtt	UAAAGCAUUGGUAUUUGACat	120
CERK	NM_022766	UGAACAUCUUGUCCUUUUAtt	UAAAAGGACAAGAUGUUCAat	137
CERK	NM_022766	AGUUUGUUACUGUUAAAAUtt	AUUUUAACAGUAACAAACUtg	132
CERKL	NM_001030313	GGUUUAGACAGUUCAAGAAtt	UUCUUGAACUGUCUAAACCat	108
CERKL	NM_001030313	GCAUCAGAGGUCCAUAUUAtt	UAAUAUGGACCUCUGAUGCaa	308
CERKL	NM_001030313	GCAGAGAAGUGGUACUUUAtt	UAAAGUACCACUUCUCUGCtg	250
CHEK1	NM_001274	GACACGAUUCUUUACCAAAtt	UUUGGUAAAGAAUCGUGUCat	218
CHEK1	NM_001274	GCAUGGUAUUGGAAUAACUtt	AGUUAUUCCAAUACCAUGCag	306
CHEK1	NM_001274	GCAACAGUAUUUCGGUAUAtt	UAUACCGAAAUACUGUUGCca	225
CHEK2	NM_145862	GAUCAGUCAGUUUAUCCUAtt	UAGGAUAAACUGACUGAUCat	85
CHEK2	NM_145862	GGCACGUUUUACGACAGAAtt	UUCUGUCGUAAAACGUGCCtt	130
CHEK2	NM_145862	GCACUGUCACUAAGCAGAAtt	UUCUGCUUAGUGACAGUGCaa	114
CHKA	NM_212469	GGAAAAGUAUCUAAAGGAAtt	UUCCUUUAGAUACUUUUCCat	263
CHKA	NM_212469	CAAUGGAAAAGUAUCUAAAtt	UUUAGAUACUUUUCCAUUGtg	388
CHKA	NM_212469	GCUUGAAGUUAAUAGGUUUtt	AAACCUAUUAACUUCAAGCaa	80
CHUK	NM_001278	GGACUAAAAGAAGACUAUAtt	UAUAGUCUUCUUUUAGUCCag	156
CHUK	NM_001278	GAAGGAUCCAAAGUGUAUAtt	UAUACACUUUGGAUCCUUCtt	395
CHUK	NM_001278	GCCUAGAGCUAAGUACCAAtt	UUGGUACUUAGCUCUAGGCga	102
CIB1	NM_006384	AGACAUCAAGUCCCAUUAUtt	AUAAUGGGACUUGAUGUCUgg	242
CIB1	NM_006384	CGUCAUCUCCCGUUCUCCAtt	UGGAGAACGGGAGAUGACGtg	152
CIB1	NM_006384	ACGGAACCUUGAACAGAGAtt	UCUCUGUUCAAGGUUCCGUca	415
CIB4	NM_001029881	GAAGAUUGAGUAUGCCUUUtt	AAAGGCAUACUCAAUCUUCag	69
CIB4	NM_001029881	GAACAGUGAUGACAUGUCUtt	AGACAUGUCAUCACUGUUCag	141
CIB4	NM_001029881	AGUCGGAUCUGGACAAUGAtt	UCAUUGUCCAGAUCCGACUca	100
CIT	NM_007174	GGAAGGUGAUGACCGUCUAtt	UAGACGGUCAUCACCUUCCag	140

CIT	NM_007174	CGUGGAUUCUUACGGAAGAtt	UCUUCCGUAAGAAUCCACGaa	168
CIT	NM_007174	GAUUCUUACGGAAGACGUAtt	UACGUCUUCCGUAAGAAUCca	277
CKB	NM_001823	AGUUCUCGGAGGUGCUUAAtt	UUAAGCACCUCCGAGAACUtc	271
CKB	NM_001823	UCACCCAGAUUGAAACUCUtt	AGAGUUUCAAUCUGGGUGAgg	240
CKB	NM_001823	GAAACUCUCUUCAAGUCUAtt	UAGACUUGAAGAGAGUUUCaa	121
CKM	NM_001824	GAAGAUUGAGGAGAUCUUUtt	AAAGAUCUCCUCAAUCUUCtg	360
CKM	NM_001824	CCCUUGAACUCUACAAGAAtt	UUCUUGUAGAGUUCAAGGGtc	300
CKM	NM_001824	AGACUGACCUCAACCAUGAtt	UCAUGGUUGAGGUCAGUCUtg	190
CKMT1B	NM_020990	GCACACCACGGAUCUAGAUtt	AUCUAGAUCCGUGGUGUGCtt	238
CKMT1B	NM_020990	GUAUUGUCCUCUAGAGUCAtt	UGACUCUAGAGGACAAUACat	323
CKMT1B	NM_020990	AGGUAUGUAUUGUCCUCUAtt	UAGAGGACAAUACAUACCUct	108
CKMT2	NM_001825	GACCCACGCUUUUCUAAGAtt	UCUUAGAAAAGCGUGGGUCct	400
CKMT2	NM_001825	GGAUAAAUGAGGAGGAUCAtt	UGAUCCUCCUCAUUUAUCCag	329
CKMT2	NM_001825	GAGGCAAUAUGAAACGAGUtt	ACUCGUUUCAUAUUGCCUCct	117
CLK1	NM_004071	CAUUUCGACUGGAUCAUAUtt	AUAUGAUCCAGUCGAAAUGgt	68
CLK1	NM_004071	CUAUCUUGGGUUUACCGUAtt	UACGGUAAACCCAAGAUAGta	143
CLK1	NM_004071	CUGUCAGAGUGGAGACGUAtt	UACGUCUCCACUCUGACAGat	147
CLK2	NM_003993	GCUACAGACGCAACGAUUAtt	UAAUCGUUGCGUCUGUAGCtg	276
CLK2	NM_003993	GCUCUUCGAUCUGAUUGAAtt	UUCAAUCAGAUCGAAGAGCtg	141
CLK2	NM_003993	GAGCGAUAUGAAAUCGUUAtt	UAACGAUUUCAUAUCGCUCtt	166
CLK3	NM_001292	GCCGGUAUGUGAAGGAGAAtt	UUCUCCUUCACAUACCGGCcg	115
CLK3	NM_001292	CUACAGUCGGGAACAUGAAtt	UUCAUGUUCCCGACUGUAGga	96
CLK3	NM_001292	AGCUACCGAUGGAAGAGGAtt	UCCUCUUCCAUCGGUAGCUca	233
CLK4	NM_020666	GGAGAUACGUUGACGAAUAtt	UAUUCGUCAACGUAUCUCCgg	195
CLK4	NM_020666	GCACUUAAAUAGUACUGAUtt	AUCAGUACUAUUUAAGUGCtc	186
CLK4	NM_020666	CCAGCUAGAUUGGGAUGAAtt	UUCAUCCCAAUCUAGCUGGtt	60
CMPK	NM_016308	GGAAGGCAGAUGUAUCUUUtt	AAAGAUACAUCUGCCUUCCca	263
CMPK	NM_016308	GAUUGAUGGGUUUCCAAGAtt	UCUUGGAAACCCAUCAAUCaa	273
CMPK	NM_016308	GAAAGAUUGUACCAGUUGAtt	UCAACUGGUACAAUCUUUCct	263
CNKSR1	NM_006314	CUGCUACAGUGAGACCGAAtt	UUCGGUCUCACUGUAGCAGtc	109
CNKSR1	NM_006314	CAUUGGGCCUAGAAAUUCAtt	UGAAUUUCUAGGCCCAAUGga	121

CNKSR1	NM_006314	CCAUGACUUCCAGAGCAUAtt	UAUGCUCUGGAAGUCAUGGgt	153
CNKSR3	NM_173515	GAAGUUCACUUACCAAACAtt	UGUUUGGUAAGUGAACUUCct	132
CNKSR3	NM_173515	GGUGCUACAUCAACUCAGAtt	UCUGAGUUGAUGUAGCACCtg	106
CNKSR3	NM_173515	GGUACUCGGUGGAAACCAAtt	UUGGUUUCCACCGAGUACCca	147
COASY	NM_001042529	GGGUGUUUGGGAAUAAGAAtt	UUCUUAUUCCCAAACACCCgg	210
COASY	NM_001042529	GACGCAUUGUGGAGAGGGAtt	UCCCUCUCCACAAUGCGUCtt	440
COASY	NM_001042529	AGAUACUCACGGACAUUAUtt	AUAAUGUCCGUGAGUAUCUtc	207
COL4A3BP	NM_031361	GGCAGUUGGUUGUAGAAGAtt	UCUUCUACAACCAACUGCCaa	72
COL4A3BP	NM_031361	CGAGACGUAUUAUAUCUUUtt	AAAGAUAUAAUACGUCUCGct	201
COL4A3BP	NM_031361	GGUGACUUCUUGCAUAGUAtt	UACUAUGCAAGAAGUCACCat	318
CRKRS	NM_016507	CCGAAGAAGCAAUAUCGAAtt	UUCGAUAUUGCUUCUUCGGtt	257
CRKRS	NM_016507	GGACUUACUAAAAGCUAAAtt	UUUAGCUUUUAGUAAGUCCcg	122
CRKRS	NM_016507	GGACUUGCUCGGCUCUAUAtt	UAUAGAGCCGAGCAAGUCCaa	224
CSF1R	NM_005211	GUUGAGACCUUAGAGCACAtt	UGUGCUCUAAGGUCUCAACag	269
CSF1R	NM_005211	CCUCAACCUCGAUCAAGUAtt	UACUUGAUCGAGGUUGAGGgt	350
CSF1R	NM_005211	CAUAAUAACCGUUACCAAAtt	UUUGGUAACGGUUAUUAUGaa	140
CSK	NM_004383	GGAACAAAGUCGCCGUCAAtt	UUGACGGCGACUUUGUUCCct	512
CSK	NM_004383	CGAUUACCGAGGGAACAAAtt	UUUGUUCCCUCGGUAAUCGcc	378
CSK	NM_004383	CGCGCCUCAUUAAACCAAAtt	UUUGGUUUAAUGAGGCGCGta	182
CSNK1A1	NM_001892	GAUGAUCAGUAGAAUUGAAtt	UUCAAUUCUACUGAUCAUCtg	66
CSNK1A1	NM_001892	CAGUGAAGCUAGAAUCUCAtt	UGAGAUUCUAGCUUCACUGcc	388
CSNK1A1	NM_001892	GAAUUUGCGAUGUACUUAAtt	UUAAGUACAUCGCAAAUUCtg	87
CSNK1A1L	NM_145203	AGAAUUUUCUACACCGAGAtt	UCUCGGUGUAGAAAAUUCUtt	141
CSNK1A1L	NM_145203	AGAUGAUCAGCAGAAUUGAtt	UCAAUUCUGCUGAUCAUCUgg	86
CSNK1A1L	NM_145203	AACUCUACACGAUUCUUCAtt	UGAAGAAUCGUGUAGAGUUtg	222
CSNK1D	NM_139062	GGAUUAGCGAGAAGAAAAUtt	AUUUUCUUCUCGCUAAUCCtt	205
CSNK1D	NM_139062	UGAUCAGUCGCAUCGAAUAtt	UAUUCGAUGCGACUGAUCAtt	435
CSNK1D	NM_139062	CACGCACCUUGGAAUUGAAtt	UUCAAUUCCAAGGUGCGUGtt	44
CSNK1E	NM_001894	CCUCCGAAUUCUCAACAUAtt	UAUGUUGAGAAUUCGGAGGga	284
CSNK1E	NM_001894	GCUUUAUCGUGGUUGUUAAtt	UUAACAACCACGAUAAAGCtc	218
CSNK1E	NM_001894	UCGUGGUUGUUAAUUUGAAtt	UUCAAAUUAACAACCACGAta	149

CSNK1G1	NM_022048	CAGCUUCAUUUAGAGUACAtt	UGUACUCUAAAUGAAGCUGtg	139
CSNK1G1	NM_022048	CCUUCGAUAUGUCAGGCGAtt	UCGCCUGACAUAUCGAAGGta	240
CSNK1G1	NM_022048	GGACUUCUUUGAAAAACCUtt	AGGUUUUUCAAAGAAGUCCag	181
CSNK1G2	NM_001319	GCCUGGACUUCUUCGAGAAtt	UUCUCGAAGAAGUCCAGGCgc	218
CSNK1G2	NM_001319	GAAUCUCUAUACAAAUGAAtt	UUCAUUUGUAUAGAGAUUCtt	101
CSNK1G2	NM_001319	GCUUCGUGUUCGACUAUGAtt	UCAUAGUCGAACACGAAGCca	130
CSNK1G3	NM_004384	GGAGAAUUACGAUUAGGGAtt	UCCCUAAUCGUAAUUCUCCaa	394
CSNK1G3	NM_004384	GAGACGAUUUAGAAGCUUUtt	AAAGCUUCUAAAUCGUCUCtt	264
CSNK1G3	NM_004384	CUGACUUGUUUGAUCGAAAtt	UUUCGAUCAAACAAGUCAGta	186
CSNK2A1	NM_001895	GGCUCGAAUGGGUUCAUCUtt	AGAUGAACCCAUUCGAGCCtg	295
CSNK2A1	NM_001895	AGAUGUACGAUUAUAGUUUtt	AAACUAUAAUCGUACAUCUga	274
CSNK2A1	NM_001895	GAUCCACGUUUCAAUGAUAtt	UAUCAUUGAAACGUGGAUCta	283
CSNK2A2	NM_001896	GGAGUACAAUGUUCGUGUAtt	UACACGAACAUUGUACUCCtg	367
CSNK2A2	NM_001896	GAUCCACACUUCAACGAUAtt	UAUCGUUGAAGUGUGGAUCta	359
CSNK2A2	NM_001896	GCAUGAUCUUUCGAAGGGAtt	UCCCUUCGAAAGAUCAUGCtt	142
DAK	NM_015533	GAGCUGAUCUGUUACAAGUtt	ACUUGUAACAGAUCAGCUCct	186
DAK	NM_015533	CCGCCGAUGAGAUUGUGAAtt	UUCACAAUCUCAUCGGCGGtt	479
DAK	NM_015533	UGCCCAUGCUGGUUUCAUAtt	UAUGAAACCAGCAUGGGCAgg	263
DAPK1	NM_004938	GAUCAAGCCUAAAGAUACAtt	UGUAUCUUUAGGCUUGAUCca	252
DAPK1	NM_004938	GGCUGUAACGUGAACAUCAtt	UGAUGUUCACGUUACAGCCgg	111
DAPK1	NM_004938	GGGACACCUCCAUUACUCAtt	UGAGUAAUGGAGGUGUCCCgt	289
DAPK2	NM_014326	GAAAAUUGCUCACUUUGAUtt	AUCAAAGUGAGCAAUUUUCtt	115
DAPK2	NM_014326	CGGAAUUUGUUGCUCCAGAtt	UCUGGAGCAACAAAUUCCGgc	153
DAPK2	NM_014326	GGACUUUAUUCGGAAGCUUtt	AAGCUUCCGAAUAAAGUCCtt	219
DAPK3	NM_001348	AGUUUGCGAUCGUGCGGAAtt	UUCCGCACGAUCGCAAACUgg	193
DAPK3	NM_001348	CCAACAUCUCAGCCGUGAAtt	UUCACGGCUGAGAUGUUGGtg	340
DAPK3	NM_001348	GAGGAGUACUUCAGCAACAtt	UGUUGCUGAAGUACUCCUCgt	114
DCAKD	NM_024819	AGCACACCGUGGUAGUAUAtt	UAUACUACCACGGUGUGCUtc	144
DCAKD	NM_024819	GUACAGUAAUUAGCCGAAAtt	UUUCGGCUAAUUACUGUACtg	357
DCAKD	NM_024819	ACCGCUACGUGAUUCUGGAtt	UCCAGAAUCACGUAGCGGUat	223
DCK	NM_000788	CAACUUCGAUUAUCUUCAAtt	UUGAAGAUAAUCGAAGUUGgt	161

DCK	NM_000788	CAAUGUUCAAAGUACUCAAtt	UUGAGUACUUUGAACAUUGca	136
DCK	NM_000788	GCAUCUAAUUUGUAUGAAUtt	AUUCAUACAAAUUAGAUGCaa	129
DCLK1	NM_004734	CGAUAUAAAGUCGGAAGAAtt	UUCUUCCGACUUUAUAUCGtt	98
DCLK1	NM_004734	GAUCGACUGCUAGAGAGUAtt	UACUCUCUAGCAGUCGAUCtt	254
DCLK1	NM_004734	GAAAGUUCGUUUCUAUCGAtt	UCGAUAGAAACGAACUUUCtt	405
DCLK2	NM_001040261	CAGAUGCUCUGAAUCAUCAtt	UGAUGAUUCAGAGCAUCUGtt	140
DCLK2	NM_001040261	GAUCCUCAGCUGUUAAGUAtt	UACUUAACAGCUGAGGAUCga	112
DCLK2	NM_001040261	GCAAUUUUGCAGUAGUCAAtt	UUGACUACUGCAAAAUUGCca	128
DCLK3	XM_940612	GAAUGACCGUGUGAGGAAAtt	UUUCCUCACACGGUCAUUCtt	55
DCLK3	XM_940612	CCACUGACACUGAAGAGCAtt	UGCUCUUCAGUGUCAGUGGtt	75
DCLK3	XM_940612	GACGAGCUCUUUAACAUCAtt	UGAUGUUAAAGAGCUCGUCct	78
DDR1	NM_001954	CUCAAGAUCUGGUUAGUCUtt	AGACUAACCAGAUCUUGAGgg	156
DDR1	NM_001954	CCAUCAAAAUCGCAGACUUtt	AAGUCUGCGAUUUUGAUGGtg	38
DDR1	NM_001954	GGCUAUGCAGGUCCACUGUtt	ACAGUGGACCUGCAUAGCCtg	138
DDR2	NM_006182	CCUAUGAUCCAAUGCUUAAtt	UUAAGCAUUGGAUCAUAGGtt	163
DDR2	NM_006182	GCACUGUCAGUUACACCAAtt	UUGGUGUAACUGACAGUGCgt	191
DDR2	NM_006182	GGUUAUUGAGAAUACUGGAtt	UCCAGUAUUCUCAAUAACCtg	156
DGKA	NM_201554	GGAUCGUAAAAAUAGCAAAtt	UUUGCUAUUUUUACGAUCCgg	156
DGKA	NM_201554	GCAUCGCAGUGCUAAACAUtt	AUGUUUAGCACUGCGAUGCct	125
DGKA	NM_201554	CCGGAGAAGUUCAACAGCAtt	UGCUGUUGAACUUCUCCGGat	113
DGKB	NM_145695	GGCUUAGAAAAUAACGUGAtt	UCACGUUAUUUUCUAAGCCca	168
DGKB	NM_145695	GACAUUGUCUGUUACCUGUtt	ACAGGUAACAGACAAUGUCct	107
DGKB	NM_145695	GGUUUUGGAUUGCAUAGAAtt	UUCUAUGCAAUCCAAAACCca	72
DGKD	NM_152879	CCCUAGAGUAUUACACGGAtt	UCCGUGUAAUACUCUAGGGtt	92
DGKD	NM_003648	GGCUUACGGUUAUUCCAGAtt	UCUGGAAUAACCGUAAGCCga	282
DGKD	NM_003648	CAGAAACCCUAGAGUAUUAtt	UAAUACUCUAGGGUUUCUGgt	232
DGKE	NM_003647	GGAAUUAAACUAGAUCGAUtt	AUCGAUCUAGUUUAAUUCCat	100
DGKE	NM_003647	GGCUAAUCCUUUUCGAAUAtt	UAUUCGAAAAGGAUUAGCCag	255
DGKE	NM_003647	CUCUCGUAGUGGAACUAAUtt	AUUAGUUCCACUACGAGAGtt	521
DGKG	NM_001346	GUGUCAAAACGUACUCAAAtt	UUUGAGUACGUUUUGACACaa	134
DGKG	NM_001346	CAGAUACUAAUAUACAGAAtt	UUCUGUAUAUUAGUAUCUGcg	125

DGKG	NM_001346	CAGCGCAGAUACUAAUAUAtt	UAUAUUAGUAUCUGCGCUGtt	164
DGKH	NM_152910	GGAGUAUAAUGACAUAUGAtt	UCAUAUGUCAUUAUACUCCac	108
DGKH	NM_152910	GGAUUGGAUUAGAUGCAAAtt	UUUGCAUCUAAUCCAAUCCca	103
DGKH	NM_152910	GGAGUUCGAUUAUCAACAAtt	UUGUUGAUAAUCGAACUCCca	129
DGKI	NM_004717	GGGUGAACAAAAUCAGUUUtt	AAACUGAUUUUGUUCACCCgg	170
DGKI	NM_004717	CCGAGAAGCUUCUAUUUCAtt	UGAAAUAGAAGCUUCUCGGag	188
DGKI	NM_004717	CCUUCCGAGUUAUUGGAUAtt	UAUCCAAUAACUCGGAAGGtc	277
DGKK	NM_001013742	CACCUGAAUCUAUACGCUUtt	AAGCGUAUAGAUUCAGGUGta	117
DGKK	NM_001013742	GGAGUGUGAUGAUUCGUGAtt	UCACGAAUCAUCACACUCCat	101
DGKK	NM_001013742	GAAUUGGACUGGAUGCUAAtt	UUAGCAUCCAGUCCAAUUCcg	82
DGKQ	NM_001347	GGAUUGCCCAGGGUUCCUAtt	UAGGAACCCUGGGCAAUCCgg	164
DGKQ	NM_001347	GGAAGCUACUGAACCCUCAtt	UGAGGGUUCAGUAGCUUCCgg	105
DGKQ	NM_001347	AGAUCGUGCAGAUGAGUAAtt	UUACUCAUCUGCACGAUCUtg	97
DGKZ	NM_201532	GCACAGGAUGAGAUUUAUAtt	UAUAAAUCUCAUCCUGUGCga	82
DGKZ	NM_201532	GCCGCUUUCGGAAUAAGAUtt	AUCUUAUUCCGAAAGCGGCtg	184
DGKZ	NM_201532	CGACAAGUCUUCGACCUGAtt	UCAGGUCGAAGACUUGUCGgg	174
DGUOK	NM_080916	CACGAAAACUUACCCAGAAtt	UUCUGGGUAAGUUUUCGUGag	357
DGUOK	NM_080916	GAAACUUGCUGGAUAUGAUtt	AUCAUAUCCAGCAAGUUUCca	112
DGUOK	NM_080916	GGAUGUCAAUGAUGAUUUUtt	AAAAUCAUCAUUGACAUCCaa	176
DKFZp434B1231	NM_178275	GAAGUGUGACUGUCACUAAtt	UUAGUGACAGUCACACUUCtt	94
DKFZp434B1231	NM_178275	GAGUCAUCUUUAAGCAAGAtt	UCUUGCUUAAAGAUGACUCca	75
DKFZp434B1231	NM_178275	GGAAGCCGGUGAUAGUGAAtt	UUCACUAUCACCGGCUUCCca	132
DKFZp761P0423	XM_937796	CACUUUAGCUAUUCGUUGAtt	UCAACGAAUAGCUAAAGUGag	116
DKFZp761P0423	XM_937796	GAGAAUAACUGGUCGCUCUtt	AGAGCGACCAGUUAUUCUCat	164
DKFZp761P0423	XM_937796	GGCACCUUCUAAAAAUGAAtt	UUCAUUUUUAGAAGGUGCCgc	57
DMPK	NM_001081560	GCAAGAUCGUCCACUACAAtt	UUGUAGUGGACGAUCUUGCca	161
DMPK	NM_001081560	GCACUUCGCCUUCCAGGAUtt	AUCCUGGAAGGCGAAGUGCag	80
DMPK	NM_001081560	AGAUCGUCCACUACAAGGAtt	UCCUUGUAGUGGACGAUCUtg	93
DNAJC6	NM_014787	CACUGGAUGUAGAACUACAtt	UGUAGUUCUACAUCCAGUGtc	62
DNAJC6	NM_014787	CCCUAUGAACAAUACGCAAtt	UUGCGUAUUGUUCAUAGGGtt	135
DNAJC6	NM_014787	CGACCCAACUACAACGUGAtt	UCACGUUGUAGUUGGGUCGgt	395

DTYMK	NM_012145	UGCCGUUAAUUAAGGAAAAtt	UUUUCCUUAAUUAACGGCAct	213
DTYMK	NM_012145	GAUCAACUGAAAUCGGCAAtt	UUGCCGAUUUCAGUUGAUCtt	137
DTYMK	NM_012145	GAUGGUGGAUGCUUCCAAAtt	UUUGGAAGCAUCCACCAUCtt	221
DYRK1A	NM_130438	CAAGCAUAUUAAUGAGGUUtt	AACCUCAUUAAUAUGCUUGta	136
DYRK1A	NM_130438	CCGUAAACUUCAUAACAUUtt	AAUGUUAUGAAGUUUACGGgt	163
DYRK1A	NM_130438	GCUGACUACUUGAAGUUCAtt	UGAACUUCAAGUAGUCAGCga	188
DYRK1B	NM_004714	CAUCAAUGAGGUAUACUAUtt	AUAGUAUACCUCAUUGAUGtg	78
DYRK1B	NM_004714	GGACGAAAGAACUCAGGAAtt	UUCCUGAGUUCUUUCGUCCtt	213
DYRK1B	NM_004714	GGCACUUCAUGUUCCGGAAtt	UUCCGGAACAUGAAGUGCCgc	306
DYRK2	NM_003583	GCCUUUGGUUCGCAAGUUUtt	AAACUUGCGAACCAAAGGCag	182
DYRK2	NM_003583	CCCUAAAGAUGGUGCGGAAtt	UUCCGCACCAUCUUUAGGGcc	150
DYRK2	NM_003583	CCGGUGCUAUCACAUCUAUtt	AUAGAUGUGAUAGCACCGGtg	145
DYRK3	NM_001004023	CGGAUUUUGGAGCAUCUUAtt	UAAGAUGCUCCAAAAUCCGga	150
DYRK3	NM_001004023	GAAAAGACAUGGAGUUAUUtt	AAUAACUCCAUGUCUUUUCtt	165
DYRK3	NM_001004023	CCAUCUAGCUUAUCGAUAUtt	AUAUCGAUAAGCUAGAUGGtc	231
DYRK4	NM_003845	GUAUACACGUACAUCCAAAtt	UUUGGAUGUACGUGUAUACtt	119
DYRK4	NM_003845	GGACAGAGCAAAAACUUCUtt	AGAAGUUUUUGCUCUGUCCtg	214
DYRK4	NM_003845	CCUACAAUGUGGUGCAUAUtt	AUAUGCACCACAUUGUAGGtg	191
EEF2K	NM_013302	GCUCGAACCAGAAUGUCAAtt	UUGACAUUCUGGUUCGAGCtt	100
EEF2K	NM_013302	GCGACGAUGAGGAAGGUUAtt	UAACCUUCCUCAUCGUCGCtg	140
EEF2K	NM_013302	GGAUUUGAUUACUUACUAAtt	UUAGUAAGUAAUCAAAUCCtt	182
EGFR	NM_005228	GAAUAGGUAUUGGUGAAUUtt	AAUUCACCAAUACCUAUUCcg	234
EGFR	NM_005228	CCAUAAAUGCUACGAAUAUtt	AUAUUCGUAGCAUUUAUGGag	220
EGFR	NM_005228	GAUCUUUCCUUCUUAAAGAtt	UCUUUAAGAAGGAAAGAUCat	121
EIF2AK1	NM_014413	GAUUAAGGGUGCAACUAAAtt	UUUAGUUGCACCCUUAAUCag	93
EIF2AK1	NM_014413	GCUCAUUGAGACUACAUCAtt	UGAUGUAGUCUCAAUGAGCta	74
EIF2AK1	NM_014413	CGAAGAAUCUUCCGAAGAAtt	UUCUUCGGAAGAUUCUUCGgt	83
EIF2AK3	NM_004836	CGCGGCAGGUCAUUAGUAAtt	UUACUAAUGACCUGCCGCGcg	68
EIF2AK3	NM_004836	GUGACGAAAUGGAACAAGAtt	UCUUGUUCCAUUUCGUCACta	193
EIF2AK3	NM_004836	CAACAAGAAUAUCCGCAAAtt	UUUGCGGAUAUUCUUGUUGta	311
EIF2AK4	NM_001013703	GCACCGUCAAGAUUACGGAtt	UCCGUAAUCUUGACGGUGCct	151

EIF2AK4	NM_001013703	GGAUCCCUUUUGCAAGAUAtt	UAUCUUGCAAAAGGGAUCCgc	107
EIF2AK4	NM_001013703	GGUCCAAGGAAGCACCAAAtt	UUUGGUGCUUCCUUGGACCtc	56
EPHA1	NM_005232	GGUACCAGAUGGUUCUAGAtt	UCUAGAACCAUCUGGUACCgt	171
EPHA1	NM_005232	CUACCUCAGUAAUCACAAUtt	AUUGUGAUUACUGAGGUAGtt	113
EPHA1	NM_005232	CCUCAGUAAUCACAAUUAUtt	AUAAUUGUGAUUACUGAGGta	85
EPHA10	NM_001004338	GCAGGAUAAUAAAAACUUGtt	CAAGUUUUUAUUAUCCUGCac	61
EPHA10	NM_001004338	GCAUCUUCGUGGAACUGCAtt	UGCAGUUCCACGAAGAUGCgc	60
EPHA10	NM_001004338	CCAAGGAACUGGAUGCGAAtt	UUCGCAUCCAGUUCCUUGGcg	133
EPHA2	NM_004431	UGAUGAUCAUCACUGAGUAtt	UACUCAGUGAUGAUCAUCAtg	147
EPHA2	NM_004431	GGAAGUACGAGGUCACUUAtt	UAAGUGACCUCGUACUUCCac	135
EPHA2	NM_004431	GUAUCUUCAUUGAGCUCAAtt	UUGAGCUCAAUGAAGAUACgc	264
EPHA3	NM_182644	GGAUGUACUGCAGUACAGAtt	UCUGUACUGCAGUACAUCCtt	214
EPHA3	NM_182644	CACCUGUCCUGACGAUUAAtt	UUAAUCGUCAGGACAGGUGat	92
EPHA3	NM_182644	CGAGGUCAAAUACUAUGAAtt	UUCAUAGUAUUUGACCUCGta	95
EPHA4	NM_004438	GAACUUGGGUGGAUAGCAAtt	UUGCUAUCCACCCAAGUUCtc	197
EPHA4	NM_004438	CGAAGAUCCCAACCAAGCAtt	UGCUUGGUUGGGAUCUUCGta	131
EPHA4	NM_004438	GUACGAAGAUCCCAACCAAtt	UUGGUUGGGAUCUUCGUACgt	111
EPHA5	NM_004439	CAUCCUGCAGAGUAUCUAAtt	UUAGAUACUCUGCAGGAUGca	235
EPHA5	NM_004439	CAAUCAAGCUGUCCACGAAtt	UUCGUGGACAGCUUGAUUGgg	122
EPHA5	NM_004439	CAUUGAUCCACAUACCUAUtt	AUAGGUAUGUGGAUCAAUGta	80
EPHA6	NM_173655	GGUCAAUAGCAACUUAGUAtt	UACUAAGUUGCUAUUGACCag	128
EPHA6	NM_173655	GACCAGUAAUGAUUGUGGUtt	ACCACAAUCAUUACUGGUCtg	142
EPHA6	NM_173655	UCCCGGGAAUUAAAACUUAtt	UAAGUUUUAAUUCCCGGGAag	96
EPHA7	NM_004440	GAUGGGCAAUUUACAGUCAtt	UGACUGUAAAUUGCCCAUCat	176
EPHA7	NM_004440	GGAACGGACCUACUCAACAtt	UGUUGAGUAGGUCCGUUCCct	88
EPHA7	NM_004440	CAGCUAAUGUUGGAUUGUUtt	AACAAUCCAACAUUAGCUGgt	223
EPHA8	NM_001006943	CAGUGUGAAUGGGACAUCAtt	UGAUGUCCCAUUCACACUGga	147
EPHA8	NM_001006943	GGACAUCAGUGACUCUGGAtt	UCCAGAGUCACUGAUGUCCca	92
EPHA8	NM_001006943	CCUCAAAAUCGACACCAUUtt	AAUGGUGUCGAUUUUGAGGaa	156
EPHB1	NM_004441	GUCCGUGUCUUCUUCAAAAtt	UUUUGAAGAAGACACGGACag	223
EPHB1	NM_004441	CGACCAUCCUAACAUCAUUtt	AAUGAUGUUAGGAUGGUCGaa	152

EPHB1	NM_004441	GAAACGGGCUUAUAGCAAAtt	UUUGCUAUAAGCCCGUUUCct	157
EPHB2	NM_017449	GCGUGAUCCUGGACUAUGAtt	UCAUAGUCCAGGAUCACGCca	116
EPHB2	NM_017449	ACAUCGAUCCUUUCACCUAtt	UAGGUGAAAGGAUCGAUGUag	127
EPHB2	NM_017449	AGAUGAUCCGCAAUCCCAAtt	UUGGGAUUGCGGAUCAUCUtg	157
EPHB3	NM_004443	GGGUCUACGUGGAGCUCAAtt	UUGAGCUCCACGUAGACCCgc	253
EPHB3	NM_004443	GAAUGAAGGUUUAUAUUGAtt	UCAAUAUAAACCUUCAUUCca	157
EPHB3	NM_004443	CGAUCCUACCUACACCAGUtt	ACUGGUGUAGGUAGGAUCGga	176
EPHB4	NM_004444	GGACAAACACGGACAGUAUtt	AUACUGUCCGUGUUUGUCCga	168
EPHB4	NM_004444	GCAGAGCAAUGGGAGAGAAtt	UUCUCUCCCAUUGCUCUGCtt	88
EPHB4	NM_004444	GCUGCUGCCUUCAUAUUGAtt	UCAAUAUGAAGGCAGCAGCtg	189
EPHB6	NM_004445	CCUGGAUUACAUCUACUUAtt	UAAGUAGAUGUAAUCCAGGag	190
EPHB6	NM_004445	ACACGUACCCUACAUCUUAtt	UAAGAUGUAGGGUACGUGUgc	127
EPHB6	NM_004445	GGACAAUUGGUUGCAGACAtt	UGUCUGCAACCAAUUGUCCtg	172
ERBB2	NM_001005862	GCUCAUCGCUCACAACCAAtt	UUGGUUGUGAGCGAUGAGCac	219
ERBB2	NM_001005862	GGAGACCCGCUGAACAAUAtt	UAUUGUUCAGCGGGUCUCCat	102
ERBB2	NM_001005862	GUUGGAUGAUUGACUCUGAtt	UCAGAGUCAAUCAUCCAACat	53
ERBB3	NM_001005915	CAGUGGAUUCGAGAAGUGAtt	UCACUUCUCGAAUCCACUGca	93
ERBB3	NM_001005915	UCGUCAUGUUGAACUAUAAtt	UUAUAGUUCAACAUGACGAag	111
ERBB3	NM_001005915	GAAUGAAUUCUCUACUCUAtt	UAGAGUAGAGAAUUCAUUCat	184
ERBB4	NM_005235	CCCUUUUGUUUCUCGGAGAtt	UCUCCGAGAAACAAAAGGGtt	161
ERBB4	NM_005235	CCCUUACAAUGCAAUUGAAtt	UUCAAUUGCAUUGUAAGGGtc	84
ERBB4	NM_005235	CCCGUAAUGUCUUAGUGAAtt	UUCACUAAGACAUUACGGGct	105
ERN1	NM_001433	GAAACUUCCUUUUACCAUCtt	GAUGGUAAAAGGAAGUUUCgt	118
ERN1	NM_001433	CCUGCGCUAUCUGACCUUCtt	GAAGGUCAGAUAGCGCAGGgt	142
ERN1	NM_001433	CAGGACAUCUGGUAUGUUAtt	UAACAUACCAGAUGUCCUGct	160
ERN2	NM_033266	GAAGGACCAAUGUACGUCAtt	UGACGUACAUUGGUCCUUCga	190
ERN2	NM_033266	GGAUGAUCCCGUCAUCGAAtt	UUCGAUGACGGGAUCAUCCct	248
ERN2	NM_033266	CAAUGUACGUCACAGAAAUtt	AUUUCUGUGACGUACAUUGgt	324
ETNK1	NM_018638	GGAAUCACAAAUAAACUUAtt	UAAGUUUAUUUGUGAUUCCat	162
ETNK1	NM_018638	CAAUAAGACUGAGUUAUUAtt	UAAUAACUCAGUCUUAUUGcc	308
ETNK1	NM_018638	GCACCACAACUCUACUGUAtt	UACAGUAGAGUUGUGGUGCac	115

ETNK2	NM_018208	GCGUGAAUGAGGUGGAUUAtt	UAAUCCACCUCAUUCACGCct	179
ETNK2	NM_018208	CCAUUUCAAUGAGUUUGCAtt	UGCAAACUCAUUGAAAUGGtt	590
ETNK2	NM_018208	GGCUCUACGUGCAAGUCAAtt	UUGACUUGCACGUAGAGCCtt	92
FASTK	NM_033015	GUACAGUCACAAGGACAUAtt	UAUGUCCUUGUGACUGUACtt	61
FASTK	NM_033015	ACAAGGACAUAGUAGCUGAtt	UCAGCUACUAUGUCCUUGUga	198
FASTK	NM_033015	GCAGCAAGGUGGUACAGAAtt	UUCUGUACCACCUUGCUGCtg	184
FER	NM_005246	CACGAUCAGUUACAUCUAUtt	AUAGAUGUAACUGAUCGUGca	141
FER	NM_005246	GAAUUAUGUCAGCAACGUAtt	UACGUUGCUGACAUAAUUCat	108
FER	NM_005246	GAACAACGGCUGCUAAAGAtt	UCUUUAGCAGCCGUUGUUCtg	155
FES	NM_002005	CCACGCUGGAGAUCCUUAAtt	UUAAGGAUCUCCAGCGUGGgt	262
FES	NM_002005	CCUCAGCAAUCAGCAGACAtt	UGUCUGCUGAUUGCUGAGGtt	110
FES	NM_002005	AGUGGGUGCUGAACCAUGAtt	UCAUGGUUCAGCACCCACUtg	330
FGFR1	NM_023106	GAGGCUACAAGGUCCGUUAtt	UAACGGACCUUGUAGCCUCca	174
FGFR1	NM_023106	GCAUUGUGGAGAAUGAGUAtt	UACUCAUUCUCCACAAUGCag	91
FGFR1	NM_023106	ACACUGCGCUGGUUGAAAAtt	UUUUCAACCAGCGCAGUGUgg	203
FGFR2	NM_022970	GGAGUACUCCUAUGACAUUtt	AAUGUCAUAGGAGUACUCCat	173
FGFR2	NM_022970	GUAGGACUGUAGACAGUGAtt	UCACUGUCUACAGUCCUACtg	126
FGFR2	NM_022970	GAACAGUAUUCACCUAGUUtt	AACUAGGUGAAUACUGUUCga	251
FGFR3	NM_000142	CCGUAGCCGUGAAGAUGCUtt	AGCAUCUUCACGGCUACGGtg	274
FGFR3	NM_000142	CCUGCGUCGUGGAGAACAAtt	UUGUUCUCCACGACGCAGGtg	137
FGFR3	NM_000142	AGGUGUACAGUGACGCACAtt	UGUGCGUCACUGUACACCUtg	137
FGFR4	NM_002011	CAUUGACUACUAUAAGAAAtt	UUUCUUAUAGUAGUCAAUGtg	178
FGFR4	NM_002011	CCACCACAUUGACUACUAUtt	AUAGUAGUCAAUGUGGUGGac	301
FGFR4	NM_002011	ACACCUGCCUGGUAGAGAAtt	UUCUCUACCAGGCAGGUGUat	172
FGFRL1	NM_001004358	AGGACGAUGCGGGCAUGUAtt	UACAUGCCCGCAUCGUCCUgg	179
FGFRL1	NM_001004358	AAGAAGAAGUGGACACUGAtt	UCAGUGUCCACUUCUUCUUcc	183
FGFRL1	NM_001004358	CGACGGCUCCUACCUCAAUtt	AUUGAGGUAGGAGCCGUCGgg	74
FGR	NM_001042747	GCAUUACAAGAUCCGCAAAtt	UUUGCGGAUCUUGUAAUGCtt	191
FGR	NM_001042747	CCCUGUUCAUUGCCCUGUAtt	UACAGGGCAAUGAACAGGGtc	121
FGR	NM_001042747	GCAUCCUCAUUUGCCCACAtt	UGUGGGCAAAUGAGGAUGCag	226
FLJ10986	NM_018291	ACCUGGAUCUGAUUAAGAAtt	UUCUUAAUCAGAUCCAGGUga	129

FLJ10986	NM_018291	GAUUGAAACUGUCUCAGGAtt	UCCUGAGACAGUUUCAAUCcg	112
FLJ10986	NM_018291	GCAGGGCACUCAAUCAGUAtt	UACUGAUUGAGUGCCCUGCtg	239
FLJ25006	NM_144610	ACAGAUCAACCAUCCCUUUtt	AAAGGGAUGGUUGAUCUGUcg	68
FLJ25006	NM_144610	AGAAUAUUCUUCUAGAUGAtt	UCAUCUAGAAGAAUAUUCUcc	77
FLJ25006	NM_144610	CUACCAGAGUUUCCCAUUAtt	UAAUGGGAAACUCUGGUAGaa	243
FLT1	NM_002019	GGUGAGUAAGGAAAGCGAAtt	UUCGCUUUCCUUACUCACCat	165
FLT1	NM_002019	CCCUGAUGGAAAACGCAUAtt	UAUGCGUUUUCCAUCAGGGat	129
FLT1	NM_002019	CGCCGGAAGUUGUAUGGUUtt	AACCAUACAACUUCCGGCGag	315
FLT3	NM_004119	CAACUAUCUAAGAAGUAAAtt	UUUACUUCUUAGAUAGUUGag	154
FLT3	NM_004119	GGCUGUUCACAAUAGAUCUtt	AGAUCUAUUGUGAACAGCCtg	131
FLT3	NM_004119	GGACUAUUACAAAUCAAGAtt	UCUUGAUUUGUAAUAGUCCca	173
FLT4	NM_002020	CCAGCAUCCUGACCAUCCAtt	UGGAUGGUCAGGAUGCUGGag	119
FLT4	NM_002020	CGAGGUCAUUGUGCAUGAAtt	UUCAUGCACAAUGACCUCGgt	78
FLT4	NM_002020	GCUUCACCAUCGAAUCCAAtt	UUGGAUUCGAUGGUGAAGCcg	86
FN3K	NM_022158	CAGUGUUCGUCAAAGUCAAtt	UUGACUUUGACGAACACUGgg	194
FN3K	NM_022158	CAUCAAAACUUGGAGAGCAtt	UGCUCUCCAAGUUUUGAUGct	292
FN3K	NM_022158	CCGCCUUUGUGAUGGAGCAtt	UGCUCCAUCACAAAGGCGGcc	260
FN3KRP	NM_024619	ACAAGAAGCUUGGAGAGAUtt	AUCUCUCCAAGCUUCUUGUta	81
FN3KRP	NM_024619	CUACGACACGGAUCAAGGAtt	UCCUUGAUCCGUGUCGUAGct	146
FN3KRP	NM_024619	GAGUGUUCGUGAAAGUGAAtt	UUCACUUUCACGAACACUCgt	49
FRAP1	NM_004958	GCUCGUAGUUGGGAUAACAtt	UGUUAUCCCAACUACGAGCag	166
FRAP1	NM_004958	CAUUCGCAUUCAGUCCAUAtt	UAUGGACUGAAUGCGAAUGat	236
FRAP1	NM_004958	GGAGCCUUGUUGAUCCUUAtt	UAAGGAUCAACAAGGCUCCat	77
FRK	NM_002031	GCAACUACAAGGCUAUAUUtt	AAUAUAGCCUUGUAGUUGCtg	189
FRK	NM_002031	CCAUUUGAUUUGUCGUAUAtt	UAUACGACAAAUCAAAUGGag	98
FRK	NM_002031	GCAGACAAGUCAACCGUGAtt	UCACGGUUGACUUGUCUGCct	137
FUK	NM_145059	GACUUACACUCAACCCUCAtt	UGAGGGUUGAGUGUAAGUCta	77
FUK	NM_145059	GCACCUACCUAGGCUUGGAtt	UCCAAGCCUAGGUAGGUGCag	118
FUK	NM_145059	GGCCUACGCUCAGAAUCAUtt	AUGAUUCUGAGCGUAGGCCgg	50
FYN	NM_153048	GCUUGUACAACAUUACUCAtt	UGAGUAAUGUUGUACAAGCtg	136
FYN	NM_153048	GGGAUGAUAUGAAAGGAGAtt	UCUCCUUUCAUAUCAUCCCaa	112

FYN	NM_153048	GAUUGAUAGAAGACAAUGAtt	UCAUUGUCUUCUAUCAAUCgg	82
GAK	XM_001127411	GUCCGUCGCUAAUUAUGCAtt	UGCAUAAUUAGCGACGGACtg	297
GAK	XM_001127411	CACCAGAAAUCAUAGACUUtt	AAGUCUAUGAUUUCUGGUGtt	178
GAK	XM_001127411	CGAGGAAUACAACACCAAUtt	AUUGGUGUUGUAUUCCUCGtg	157
GALK1	NM_000154	GACCAGUUCAUCUCACUUAtt	UAAGUGAGAUGAACUGGUCca	125
GALK1	NM_000154	UGCUCAUCACCAACUCUAAtt	UUAGAGUUGGUGAUGAGCAcg	97
GALK1	NM_000154	UCAAGGGAGUGAUUCAGUAtt	UACUGAAUCACUCCCUUGAca	341
GALK2	NM_002044	GGAGAGUUGAUGAACCAGAtt	UCUGGUUCAUCAACUCUCCca	152
GALK2	NM_002044	GGAACACUUUGGUCUUAGUtt	ACUAAGACCAAAGUGUUCCtg	131
GALK2	NM_002044	CGAGUGCUCCAGUUUAAGAtt	UCUUAAACUGGAGCACUCGcg	208
GCK	NM_033507	GGCACGAAGACAUCGAUAAtt	UUAUCGAUGUCUUCGUGCCtc	183
GCK	NM_033507	GCAUGUGCGUCAAUACCGAtt	UCGGUAUUGACGCACAUGCgg	146
GCK	NM_033507	GGUCAGCAGCUGUAUGAGAtt	UCUCAUACAGCUGCUGACCgg	293
GCKR	NM_001486	CCCUGUUAUUAGCAGCCCAtt	UGGGCUGCUAAUAACAGGGtt	176
GCKR	NM_001486	GGCAUUGCAGCAUCUCAAAtt	UUUGAGAUGCUGCAAUGCCct	131
GCKR	NM_001486	GAACAUUGUUCGACUGCUAtt	UAGCAGUCGAACAAUGUUCtc	304
GK	NM_203391	GAGUCUUAGUAAAAGAAUUtt	AAUUCUUUUACUAAGACUCtc	167
GK	NM_203391	ACUUAGUCAUCAUCAAGUAtt	UACUUGAUGAUGACUAAGUag	148
GK	NM_203391	CAUCAUCAAGUAGAAAUAAtt	UUAUUUCUACUUGAUGAUGac	114
GK2	NM_033214	GGAAGAAAGCCGUAAUGAAtt	UUCAUUACGGCUUUCUUCCat	133
GK2	NM_033214	CAAUAAAUGUCAUAUUGCUtt	AGCAAUAUGACAUUUAUUGgt	136
GK2	NM_033214	GUAACUUCGUCAAGUCUAAtt	UUAGACUUGACGAAGUUACta	179
GK5	NM_001039547	CGAGCAAUAUUGGAGUCAAtt	UUGACUCCAAUAUUGCUCGta	124
GK5	NM_001039547	GGAAAUAGCCUUCAACAGAtt	UCUGUUGAAGGCUAUUUCCag	98
GK5	NM_001039547	GGGUAGAAAUUGAUCCUGAtt	UCAGGAUCAAUUUCUACCCag	224
GLYCTK	NM_145262	GGAUGACAGGUACCAAUGUtt	ACAUUGGUACCUGUCAUCCct	105
GLYCTK	NM_145262	GCCCACAAUGACUCACAUAtt	UAUGUGAGUCAUUGUGGGCta	137
GLYCTK	NM_145262	ACAUAGCCGUGUCCAGGUAtt	UACCUGGACACGGCUAUGUgg	98
GRIP2	XM_940982	GAACAUUGGUGACUAUAUUtt	AAUAUAGUCACCAAUGUUCag	104
GRIP2	XM_940982	GAGAGACCGUCACACUGAAtt	UUCAGUGUGACGGUCUCUCca	81
GRIP2	XM_940982	GCACCGACAAGGAUGGAAAtt	UUUCCAUCCUUGUCGGUGCca	525

GRK1	NM_002929	GCAAUGUCCGGAUCUCUGAtt	UCAGAGAUCCGGACAUUGCcg	280
GRK1	NM_002929	CCAAAACUGUCUACGCAAAtt	UUUGCGUAGACAGUUUUGGag	80
GRK1	NM_002929	CUAUGCGUUUGAAACCAAAtt	UUUGGUUUCAAACGCAUAGgc	98
GRK4	NM_182982	GGACUGUCAAUCUUAGAUAtt	UAUCUAAGAUUGACAGUCCac	123
GRK4	NM_182982	GAGUUGGAACAGUCGGCUAtt	UAGCCGACUGUUCCAACUCtt	137
GRK4	NM_182982	GAAUCAAGAAUGAUACCGAtt	UCGGUAUCAUUCUUGAUUCtt	327
GRK5	NM_005308	CGUCUACCGAGAUCUGAAAtt	UUUCAGAUCUCGGUAGACGgt	94
GRK5	NM_005308	GAAGGACCAUAGACAGAGAtt	UCUCUGUCUAUGGUCCUUCgg	34
GRK5	NM_005308	GGCAACCGGUGACCAAAAAtt	UUUUUGGUCACCGGUUGCCtt	67
GRK6	NM_001004106	GGACUUCUACCAGAAGUUUtt	AAACUUCUGGUAGAAGUCCtg	88
GRK6	NM_001004106	GAGAAAAAGCGGAUCAAGAtt	UCUUGAUCCGCUUUUUCUCta	138
GRK6	NM_001004106	GCAUCUACUUCAACCGUUUtt	AAACGGUUGAAGUAGAUGCtg	79
GRK7	NM_139209	GAUCCUAAUGGAAAAGGUAtt	UACCUUUUCCAUUAGGAUCtc	157
GRK7	NM_139209	GCAACUGCAGGUUAUCUGAtt	UCAGAUAACCUGCAGUUGCcg	158
GRK7	NM_139209	CGAACACCAUUCAAAGAUUtt	AAUCUUUGAAUGGUGUUCGtc	77
GSG2	NM_031965	GACAAGAUGCUGAAACAAAtt	UUUGUUUCAGCAUCUUGUCtg	95
GSG2	NM_031965	GUAAGCUAAAUGUAUCUUAtt	UAAGAUACAUUUAGCUUACtt	329
GSG2	NM_031965	CCAGGACCUGACUCCUUUAtt	UAAAGGAGUCAGGUCCUGGgt	37
GSK3A	NM_019884	GAAAGACGAGCUUUACCUAtt	UAGGUAAAGCUCGUCUUUCtt	106
GSK3A	NM_019884	ACACCAACCCGGGAACAAAtt	UUUGUUCCCGGGUUGGUGUtc	164
GSK3A	NM_019884	GGCUUACACGGACAUCAAAtt	UUUGAUGUCCGUGUAAGCCac	158
GSK3B	NM_002093	CUCAAGAACUGUCAAGUAAtt	UUACUUGACAGUUCUUGAGtg	180
GSK3B	NM_002093	CGAGAGCUCCAGAUCAUGAtt	UCAUGAUCUGGAGCUCUCGat	197
GSK3B	NM_002093	GCUAGAUCACUGUAACAUAtt	UAUGUUACAGUGAUCUAGCtt	160
GUK1	NM_000858	GGCUUCAGCGUGUCCCAUAtt	UAUGGGACACGCUGAAGCCaa	221
GUK1	NM_000858	GAGGCGCUCUCUGAGGAAAtt	UUUCCUCAGAGAGCGCCUCct	30
GUK1	NM_000858	GGCCAUGAACCGCAUCUGUtt	ACAGAUGCGGUUCAUGGCCtg	190
HCK	NM_002110	UGAUGGAGAUCGUCACCUAtt	UAGGUGACGAUCUCCAUCAgc	134
HCK	NM_002110	GAGAUACCGUGAAACAUUAtt	UAAUGUUUCACGGUAUCUCcc	237
HCK	NM_002110	GGGAUAGCGAGACCACUAAtt	UUAGUGGUCUCGCUAUCCCgg	204
HGS	NM_004712	CGUCUUUCCAGAAUUCAAAtt	UUUGAAUUCUGGAAAGACGtg	95

HGS	NM_004712	UGGAAUCUGUGGUAAAGAAtt	UUCUUUACCACAGAUUCCAtg	257
HGS	NM_004712	CACGGUAUCUCAACCGGAAtt	UUCCGGUUGAGAUACCGUGcg	491
HIPK1	NM_198269	CAUUGAUCUGUUAAAGAAAtt	UUUCUUUAACAGAUCAAUGta	94
HIPK1	NM_198269	GAUUUGCACUCAGACAGAUtt	AUCUGUCUGAGUGCAAAUCtg	66
HIPK1	NM_198269	GCUCAAUACAGUGCACAAUtt	AUUGUGCACUGUAUUGAGCtg	44
HIPK2	NM_022740	CCAUGACCUUUAACAACCAtt	UGGUUGUUAAAGGUCAUGGtc	191
HIPK2	NM_022740	GAGAAUCACUCCAAUCGAAtt	UUCGAUUGGAGUGAUUCUCtt	160
HIPK2	NM_022740	CGGACUCACCAUAUCCUUUtt	AAAGGAUAUGGUGAGUCCGtg	134
HIPK3	NM_001048200	CGUGUACCUCAAGACCUAUtt	AUAGGUCUUGAGGUACACGga	93
HIPK3	NM_001048200	CCGUGUACCUCAAGACCUAtt	UAGGUCUUGAGGUACACGGag	76
HIPK3	NM_001048200	CGAUGUGGAUUGAAGCGCAtt	UGCGCUUCAAUCCACAUCGct	125
HIPK4	NM_144685	CUCAAGUCGUUGGACCAGAtt	UCUGGUCCAACGACUUGAGca	162
HIPK4	NM_144685	AGUUCUACCUGGUCUUUGAtt	UCAAAGACCAGGUAGAACUtg	110
HIPK4	NM_144685	ACAUGACCAUGGAAGCUGAtt	UCAGCUUCCAUGGUCAUGUtg	92
HK1	NM_033497	CAUCCACACUUCUCCAGAAtt	UUCUGGAGAAGUGUGGAUGaa	104
HK1	NM_033497	CGAUGAAACUCUCAUAGAUtt	AUCUAUGAGAGUUUCAUCGga	167
HK1	NM_033497	GAUCGAGAGUGACCGAUUAtt	UAAUCGGUCACUCUCGAUCtg	211
HK2	NM_000189	GGAUGAAGGUAGAAAUGGAtt	UCCAUUUCUACCUUCAUCCtc	93
HK2	NM_000189	UGGGUGAGAUUGUCCGUAAtt	UUACGGACAAUCUCACCCAgg	111
HK2	NM_000189	CAGAGGUUCGAGAAAAUGAtt	UCAUUUUCUCGAACCUCUGct	142
HK3	NM_002115	ACCAUACCCUGGACCAUGAtt	UCAUGGUCCAGGGUAUGGUcg	115
HK3	NM_002115	AGAUGAUCGGAGGCCUGUAtt	UACAGGCCUCCGAUCAUCUtc	159
HK3	NM_002115	GGAAUGCGAUGUCUCCUUAtt	UAAGGAGACAUCGCAUUCCgg	152
HKDC1	NM_025130	GGAGCUCUUUGAUCACAUUtt	AAUGUGAUCAAAGAGCUCCtc	119
HKDC1	NM_025130	GUGCGAAUGUACAACAAGAtt	UCUUGUUGUACAUUCGCACtg	148
HKDC1	NM_025130	CCCUCACUUUUCUAGAAUAtt	UAUUCUAGAAAAGUGAGGGtg	259
HUNK	NM_014586	GCUCCUUGAUAUUUUAGAAtt	UUCUAAAAUAUCAAGGAGCtg	162
HUNK	NM_014586	GCCGCAAUAUUUUCCGCAAtt	UUGCGGAAAAUAUUGCGGCag	318
HUNK	NM_014586	GGCUCUACCAGAUAGAAAAtt	UUUUCUAUCUGGUAGAGCCgg	250
ICK	NM_014920	GGACUCGCAUUUAUUCACAtt	UGUGAAUAAAUGCGAGUCCtt	129
ICK	NM_014920	CAAUGUCAGUAAUCAGCAAtt	UUGCUGAUUACUGACAUUGtc	258

ICK	NM_014920	CCCAAUAACUUAAAGACCUtt	AGGUCUUUAAGUUAUUGGGta	108
IGF1R	NM_000875	GCAUGGUAGCCGAAGAUUUtt	AAAUCUUCGGCUACCAUGCaa	88
IGF1R	NM_000875	CCGAAGAUUUCACAGUCAAtt	UUGACUGUGAAAUCUUCGGct	84
IGF1R	NM_000875	GGAGUUCAAUUGUCACCAUtt	AUGGUGACAAUUGAACUCCtt	151
IGSF22	NM_173588	GAAGAGGGAUGACAAGUAUtt	AUACUUGUCAUCCCUCUUCag	165
IGSF22	NM_173588	CCUCUGUAUUCAUCGCAGAtt	UCUGCGAUGAAUACAGAGGct	128
IGSF22	NM_173588	CGCACACGCUUAAGAUUAAtt	UUAAUCUUAAGCGUGUGCGtc	130
IHPK1	NM_153273	GCAAUGCCCUCUAUCAAUAtt	UAUUGAUAGAGGGCAUUGCgg	95
IHPK1	NM_153273	GGCAUUACCUCUGCAGGAAtt	UUCCUGCAGAGGUAAUGCCct	191
IHPK1	NM_153273	AGACAGAGGCUACGUGUUUtt	AAACACGUAGCCUCUGUCUgg	425
IHPK2	NM_001005909	GAACAUCAGUUCUACGAGAtt	UCUCGUAGAACUGAUGUUCcc	217
IHPK2	NM_001005909	GCGUGCUCCGCUUCAAUGAtt	UCAUUGAAGCGGAGCACGCat	261
IHPK2	NM_001005909	GCUUCAAUGAGACAACCCUtt	AGGGUUGUCUCAUUGAAGCgg	171
IHPK3	NM_054111	UGACUUUGCUCAUACCACAtt	UGUGGUAUGAGCAAAGUCAat	66
IHPK3	NM_054111	CUCUAUCAGUUCCUACAUAtt	UAUGUAGGAACUGAUAGAGgg	83
IHPK3	NM_054111	GCAGGUUUAUCAAACAGAUtt	AUCUGUUUGAUAAACCUGCat	36
IKBKB	NM_001556	GGGCAGUCUUUGCACAUCAtt	UGAUGUGCAAAGACUGCCCtg	135
IKBKB	NM_001556	GGACAUUGUUGUUAGCGAAtt	UUCGCUAACAACAAUGUCCac	166
IKBKB	NM_001556	GACUUGAAUGGAACGGUGAtt	UCACCGUUCCAUUCAAGUCtt	111
IKBKE	NM_014002	CGACACCAGGAGUACCUCUtt	AGAGGUACUCCUGGUGUCGgg	66
IKBKE	NM_014002	CAGUGCUGUUUGGACAAGAtt	UCUUGUCCAAACAGCACUGaa	135
IKBKE	NM_014002	AGCUGGAUAAGGUGAAUUUtt	AAAUUCACCUUAUCCAGCUtg	536
IKBKG	NM_003639	AAACAGGAGGUGAUCGAUAtt	UAUCGAUCACCUCCUGUUUgg	65
IKBKG	NM_003639	GGAUCGAGGACAUGAGGAAtt	UUCCUCAUGUCCUCGAUCCtg	120
IKBKG	NM_003639	GAUUGUGAUGGAGACCGUUtt	AACGGUCUCCAUCACAAUCtt	133
ILK	NM_004517	GCCGUAGUGUAAUGAUUGAtt	UCAAUCAUUACACUACGGCta	210
ILK	NM_004517	CGACCCAAAUUUGACAUGAtt	UCAUGUCAAAUUUGGGUCGct	98
ILK	NM_004517	GAAUCACUCUGGAGAGCUAtt	UAGCUCUCCAGAGUGAUUCtc	84
INSR	NM_000208	GAAUGACCAGAGACAUCUAtt	UAGAUGUCUCUGGUCAUUCca	124
INSR	NM_000208	GAACGAUGUUGGACUCAUAtt	UAUGAGUCCAACAUCGUUCga	79
INSR	NM_000208	CUACGUGACAGACUAUUUAtt	UAAAUAGUCUGUCACGUAGaa	63

INSRR	NM_014215	GCUUCAUCGUGUACUACAAtt	UUGUAGUACACGAUGAAGCtg	213
INSRR	NM_014215	GGAUGACUCGGGACGUGUAtt	UACACGUCCCGAGUCAUCCcg	123
INSRR	NM_014215	GGAAGCUUCUGUCAUGAAAtt	UUUCAUGACAGAAGCUUCCtt	139
IPMK	NM_152230	CCAAACGAUUUAUACCUAAtt	UUAGGUAUAAAUCGUUUGGtg	102
IPMK	NM_152230	CAGCUUAAUUUUUACGCAAtt	UUGCGUAAAAAUUAAGCUGct	88
IPMK	NM_152230	GCAUUACGGAAGAAGCUUAtt	UAAGCUUCUUCCGUAAUGCtg	145
IPPK	NM_022755	GCAGAUCAGCAAAUACUGUtt	ACAGUAUUUGCUGAUCUGCtt	137
IPPK	NM_022755	GCAAGAUCGUCAACUAUUAtt	UAAUAGUUGACGAUCUUGCcg	85
IPPK	NM_022755	CCUUCCUAAUUUAACCAGAtt	UCUGGUUAAAUUAGGAAGGca	139
IQCH	NM_001031715	GAGUUUGAGCUGACGAAUAtt	UAUUCGUCAGCUCAAACUCtg	120
IQCH	NM_001031715	GGUUGGUUACUUUUCGAUAtt	UAUCGAAAAGUAACCAACCac	257
IQCH	NM_001031715	GAACUAUGCUAUACCAGAAtt	UUCUGGUAUAGCAUAGUUCct	170
IRAK1	NM_001025242	GGUUGUCCUUGAGUAAUAAtt	UUAUUACUCAAGGACAACCtg	70
IRAK1	NM_001025242	CAAGUCAGGUUUCAUGUAAtt	UUACAUGAAACCUGACUUGct	203
IRAK1	NM_001025242	GGAAAAGACAUGUAUCACAtt	UGUGAUACAUGUCUUUUCCct	137
IRAK2	NM_001570	CAGGAUCAAUCGAAAGAUUtt	AAUCUUUCGAUUGAUCCUGga	201
IRAK2	NM_001570	CCUCCUACGUGAUCACAGAtt	UCUGUGAUCACGUAGGAGGcg	101
IRAK2	NM_001570	AGUCCAGGAUCAAUCGAAAtt	UUUCGAUUGAUCCUGGACUtg	152
IRAK3	NM_007199	GAGAGAUUUUUGAGGUAUAtt	UAUACCUCAAAAAUCUCUCct	84
IRAK3	NM_007199	GUAGAGUAGUGUUAGAUGAtt	UCAUCUAACACUACUCUACat	286
IRAK3	NM_007199	GGAUGUUCGUCAUAUUGAAtt	UUCAAUAUGACGAACAUCCag	348
IRAK4	NM_016123	GGUUGACAUUACUACUGAAtt	UUCAGUAGUAAUGUCAACCat	112
IRAK4	NM_016123	GCCUAAUGGUUCAUUGCUAtt	UAGCAAUGAACCAUUAGGCat	242
IRAK4	NM_016123	GGUGUGGUUUUACUAGAAAtt	UUUCUAGUAAAACCACACCaa	117
ITGB1BP3	NM_014446	AUUCAGAACUCGCUGCUGAtt	UCAGCAGCGAGUUCUGAAUgt	194
ITGB1BP3	NM_014446	ACUGCUGCGUGAUCCAUCAtt	UGAUGGAUCACGCAGCAGUtg	163
ITGB1BP3	NM_014446	GCAUGUGAGCGUUUCCCUAtt	UAGGGAAACGCUCACAUGCtg	353
ITK	NM_005546	CCUAUGAGUGGUACAAUAAtt	UUAUUGUACCACUCAUAGGtt	193
ITK	NM_005546	GAGCCUUCAUGGUAAGGGAtt	UCCCUUACCAUGAAGGCUCct	134
ITK	NM_005546	GAGUGGUACAAUAAGAGUAtt	UACUCUUAUUGUACCACUCat	144
ITPK1	NM_014216	GAUUGAGGCCUACAUGGAAtt	UUCCAUGUAGGCCUCAAUCtt	119

ITPK1	NM_014216	GGAUGGAGGUUGUGCAGCUtt	AGCUGCACAACCUCCAUCCct	92
ITPK1	NM_014216	GUUGUGCAGCUGAACCUUAtt	UAAGGUUCAGCUGCACAACct	95
ITPKA	NM_002220	GAAGGACAUGUACAAGAAAtt	UUUCUUGUACAUGUCCUUCcg	188
ITPKA	NM_002220	GGACUUACCUAGAGGAGGAtt	UCCUCCUCUAGGUAAGUCCtg	94
ITPKA	NM_002220	CGAGGACGUGGGUCAGAAAtt	UUUCUGACCCACGUCCUCGcc	96
ITPKB	NM_002221	GGAUCGAGGGAAUCAAGAAtt	UUCUUGAUUCCCUCGAUCCtg	220
ITPKB	NM_002221	GAGAGUUCACUAAAGGAAAtt	UUUCCUUUAGUGAACUCUCtg	160
ITPKB	NM_002221	CAUCCGCUUUUGUAAGGAUtt	AUCCUUACAAAAGCGGAUGga	200
ITPKC	NM_025194	GGAUGGUCGGAUUCUGAAAtt	UUUCAGAAUCCGACCAUCCtc	116
ITPKC	NM_025194	GGACCUAUCUGGAAGAGGAtt	UCCUCUUCCAGAUAGGUCCtg	121
ITPKC	NM_025194	GGAAGGACAUGUAUGAGAAtt	UUCUCAUACAUGUCCUUCCgg	240
JAK1	NM_002227	CCAUCACCGUUGAUGACAAtt	UUGUCAUCAACGGUGAUGGtg	87
JAK1	NM_002227	GUAUUAAGCUCAUCAUGGAtt	UCCAUGAUGAGCUUAAUACca	89
JAK1	NM_002227	GGUGCUGUCUAGGCAAGAAtt	UUCUUGCCUAGACAGCACCgt	150
JAK2	NM_004972	CAAAGAUCCAAGACUAUCAtt	UGAUAGUCUUGGAUCUUUGct	121
JAK2	NM_004972	GGACUGUAUGUACUUCGAUtt	AUCGAAGUACAUACAGUCCag	160
JAK2	NM_004972	CCAGCGGAAUUUAUGCGUAtt	UACGCAUAAAUUCCGCUGGtg	139
JAK3	NM_000215	CUAAGAAACUCCAAUUUUAtt	UAAAAUUGGAGUUUCUUAGca	234
JAK3	NM_000215	GUAUCGUGGUGUCAGCUAUtt	AUAGCUGACACCACGAUACtt	146
JAK3	NM_000215	GGAGAUUCAGAUCCUCAAAtt	UUUGAGGAUCUGAAUCUCCcg	94
KDR	NM_002253	CAUGUUCUCUAAUAGCACAtt	UGUGCUAUUAGAGAACAUGgt	164
KDR	NM_002253	CCAUCGUCAUGGAUCCAGAtt	UCUGGAUCCAUGACGAUGGac	109
KDR	NM_002253	GACGGACAGUGGUAUGGUUtt	AACCAUACCACUGUCCGUCtg	164
KHK	NM_006488	GUAUGGUCGUGUGAGGAAAtt	UUUCCUCACACGACCAUACaa	119
KHK	NM_006488	GGUAAGGCCUUAUAAUGUAtt	UACAUUAUAAGGCCUUACCca	206
KHK	NM_006488	GCCUGAAAGUCUCACCCUUtt	AAGGGUGAGACUUUCAGGCtc	100
KIAA0999	NM_025164	GGAACAUUGUUCAUCGUGAtt	UCACGAUGAACAAUGUUCCga	100
KIAA0999	NM_025164	GCUUGAACGUGAAUCGGUUtt	AACCGAUUCACGUUCAAGCtg	99
KIAA0999	NM_025164	GAACAUUGUUCAUCGUGAUtt	AUCACGAUGAACAAUGUUCcg	177
KIAA1804	NM_032435	GUUUAAGAGAAGUCGUUUAtt	UAAACGACUUCUCUUAAACtt	105
KIAA1804	NM_032435	GAUUGUAGUGUAUCAAGAAtt	UUCUUGAUACACUACAAUCag	191

KIAA1804	NM_032435	GCGGACAUGUGGGAAUGUAtt	UACAUUCCCACAUGUCCGCtg	199
KIT	NM_000222	CAGUGGAUCUAUAUGAACAtt	UGUUCAUAUAGAUCCACUGct	138
KIT	NM_000222	GCAGCGAUAGUACUAAUGAtt	UCAUUAGUACUAUCGCUGCag	130
KIT	NM_000222	GAAUGCCGGUCGAUUCUAAtt	UUAGAAUCGACCGGCAUUCca	184
KSR1	NM_014238	GCCUACUUCAUUCAUCAUAtt	UAUGAUGAAUGAAGUAGGCag	110
KSR1	NM_014238	GGAAUGAAGCGUGUCCUGAtt	UCAGGACACGCUUCAUUCCtt	206
KSR1	NM_014238	GGAGUGAAGUGCAAGCAUUtt	AAUGCUUGCACUUCACUCCaa	268
KSR2	NM_173598	CGAAAACUGAUACACUUGAtt	UCAAGUGUAUCAGUUUUCGgg	204
KSR2	NM_173598	GCAUCCCACUACUACAAAUtt	AUUUGUAGUAGUGGGAUGCtg	118
KSR2	NM_173598	CAGGCAGAUUGCUCAAGAAtt	UUCUUGAGCAAUCUGCCUGgt	132
LATS1	NM_004690	GCCUUGCAGGAAAUUCGAAtt	UUCGAAUUUCCUGCAAGGCtt	94
LATS1	NM_004690	CCUCCAUACGAGUCAAUCAtt	UGAUUGACUCGUAUGGAGGaa	218
LATS1	NM_004690	GGAGUGAUGAUAACGAGGAtt	UCCUCGUUAUCAUCACUCCat	232
LATS2	NM_014572	GGAAGAUCCUCUACCAGAAtt	UUCUGGUAGAGGAUCUUCCgc	152
LATS2	NM_014572	GUUCGGACCUUAUCAGAAAtt	UUUCUGAUAAGGUCCGAACtt	136
LATS2	NM_014572	GCAUUUUACGAAUUCACCUtt	AGGUGAAUUCGUAAAAUGCgt	180
LCK	NM_001042771	CCCGAACCCUGGUUCUUCAtt	UGAAGAACCAGGGUUCGGGct	179
LCK	NM_001042771	GGAAUUAUAUUCAUCGUGAtt	UCACGAUGAAUAUAAUUCCgc	126
LCK	NM_001042771	GGCAUCAAGUUGACCAUCAtt	UGAUGGUCAACUUGAUGCCtg	183
LIMK1	NM_002314	GCAUGACCCUCACGAUACAtt	UGUAUCGUGAGGGUCAUGCtc	136
LIMK1	NM_002314	CCUCACGUGUGGGACCUUUtt	AAAGGUCCCACACGUGAGGca	90
LIMK1	NM_002314	GCAUGAGCCCAGAUGUGAAtt	UUCACAUCUGGGCUCAUGCag	116
LIMK2	NM_001031801	AGCUGUUGAUUGAACAUGAtt	UCAUGUUCAAUCAACAGCUga	134
LIMK2	NM_001031801	GCUGCAAGGUGAUCAUUGAtt	UCAAUGAUCACCUUGCAGCtc	195
LIMK2	NM_001031801	CGUUCUCUGUGAGAUCAUUtt	AAUGAUCUCACAGAGAACGat	153
LMTK2	NM_014916	GCGACUAUUAUAUCCAGUUtt	AACUGGAUAUAAUAGUCGCtt	53
LMTK2	NM_014916	GAGCAUCUUCUAUCCGGUUtt	AACCGGAUAGAAGAUGCUCgt	305
LMTK2	NM_014916	CAGUCUACCUGUUUGACCAtt	UGGUCAAACAGGUAGACUGtg	212
LMTK3	XM_936372	GGAAUUUGAGAACCCUGAAtt	UUCAGGGUUCUCAAAUUCCtt	112
LMTK3	XM_936372	GGACUACUGGUAUGACAUUtt	AAUGUCAUACCAGUAGUCCgc	117
LMTK3	XM_936372	AGUUCAUCUCGGAAGCACAtt	UGUGCUUCCGAGAUGAACUtg	97

LOC375133	NM_199345	GGAAGCAAGUCAACCCAAAtt	UUUGGGUUGACUUGCUUCCga	123
LOC375133	NM_199345	GCGGCUGCGUGAAGACAUAtt	UAUGUCUUCACGCAGCCGCtt	174
LOC375133	NM_199345	GGACCGCCAUGUUCUCAGAtt	UCUGAGAACAUGGCGGUCCaa	154
LOC390877	XM_372705	CCAGCGAAAUGCCUUGAGAtt	UCUCAAGGCAUUUCGCUGGta	134
LOC390877	XM_372705	GGUCUUGACCUACCAGCGAtt	UCGCUGGUAGGUCAAGACCgg	250
LOC390877	XM_372705	AGAAUAUGGCGACCAAGUAtt	UACUUGGUCGCCAUAUUCUtg	279
LRPPRC	NM_133259	GGGCUCUAAUGAGACUAGAtt	UCUAGUCUCAUUAGAGCCCaa	73
LRPPRC	NM_133259	CUAUAAGAGAUGUCCUAAAtt	UUUAGGACAUCUCUUAUAGgt	128
LRPPRC	NM_133259	GGAAGUUCCGUUUGACGUAtt	UACGUCAAACGGAACUUCCtg	335
LRRK1	NM_024652	GGCCUCGCAUUGUAUAUGAtt	UCAUAUACAAUGCGAGGCCtg	142
LRRK1	NM_024652	GCACAUCAACAUCAAGCUAtt	UAGCUUGAUGUUGAUGUGCtc	163
LRRK1	NM_024652	GACUCAGACAUGCUACAUAtt	UAUGUAGCAUGUCUGAGUCct	375
LRRK2	NM_198578	GCUGUGCCUUAUAACCGAAtt	UUCGGUUAUAAGGCACAGCct	80
LRRK2	NM_198578	GUGAUGACCUGGAUAGUGAtt	UCACUAUCCAGGUCAUCACtt	73
LRRK2	NM_198578	GGAAACGAUUAUUACAACUtt	AGUUGUAAUAAUCGUUUCCgg	85
LTK	NM_206961	GCAUGGAGCUAGCUGUGGAtt	UCCACAGCUAGCUCCAUGCct	266
LTK	NM_206961	AGAUCUUUGGAGUGCCUAAtt	UUAGGCACUCCAAAGAUCUgt	191
LTK	NM_206961	GGAGAGGUAGAGAUCCGAAtt	UUCGGAUCUCUACCUCUCCgt	225
LY6G5B	NM_021221	GCUCAUCCCUGUGCAUGGUtt	ACCAUGCACAGGGAUGAGCtg	188
LY6G5B	NM_021221	CCUUCUGUAGGAUGCAUUUtt	AAAUGCAUCCUACAGAAGGgt	264
LY6G5B	NM_021221	CCAGUGCUGUCAGUACGAUtt	AUCGUACUGACAGCACUGGgc	285
LYK5	NM_001003788	CCUAGAAGCUUGUUCCAAUtt	AUUGGAACAAGCUUCUAGGtt	116
LYK5	NM_001003788	GAAUCUCCAGGGUUAUGAUtt	AUCAUAACCCUGGAGAUUCtg	121
LYK5	NM_001003788	CCAACAGGAGAGUACGUGAtt	UCACGUACUCUCCUGUUGGtt	138
LYN	NM_002350	ACAUUGUGGUAGCCUUGUAtt	UACAAGGCUACCACAAUGUct	107
LYN	NM_002350	CUAGAGUAAUUGAAGAUAAtt	UUAUCUUCAAUUACUCUAGca	172
LYN	NM_002350	AGACUCAACCAGUACGUAAtt	UUACGUACUGGUUGAGUCUtc	100
MADD	NM_130470	CCACUACUAUAGUAAAGAAtt	UUCUUUACUAUAGUAGUGGgt	125
MADD	NM_130470	CAACAAGUUCUAUACUAAAtt	UUUAGUAUAGAACUUGUUGag	152
MADD	NM_130470	GACCCACUACUAUAGUAAAtt	UUUACUAUAGUAGUGGGUCtg	322
MAGI1	NM_004742	GAGCAUGUAUGAAAACCGAtt	UCGGUUUUCAUACAUGCUCct	177

MAGI1	NM_004742	GACUAGCAGGGUUCACGAAtt	UUCGUGAACCCUGCUAGUCca	240
MAGI1	NM_004742	GGCCCUAACUCACAACCAAtt	UUGGUUGUGAGUUAGGGCCtg	473
MAGI2	NM_012301	GAGGAAUUGUUGAUAAAGAtt	UCUUUAUCAACAAUUCCUCct	89
MAGI2	NM_012301	GGAAAACGGAAGAGGAAUAtt	UAUUCCUCUUCCGUUUUCCtt	161
MAGI2	NM_012301	CGACUUGCGAAAAAGGCUAtt	UAGCCUUUUUCGCAAGUCGtg	299
MAGI3	NM_152900	GGAUGUUUUUCUUCGAAAAtt	UUUUCGAAGAAAAACAUCCaa	122
MAGI3	NM_152900	GAACCUUGGUUGUUAUCCAtt	UGGAUAACAACCAAGGUUCtg	90
MAGI3	NM_152900	GAUGUUGUCUUGCAACGAAtt	UUCGUUGCAAGACAACAUCat	160
MAK	NM_005906	GGAGGACUAUGAUUUCGGAtt	UCCGAAAUCAUAGUCCUCCaa	154
MAK	NM_005906	GGACGUACUUAUAAUCCUAtt	UAGGAUUAUAAGUACGUCCtg	76
MAK	NM_005906	GGACAAGUGAGGUCGAUGAtt	UCAUCGACCUCACUUGUCCct	223
MAP2K1	NM_002755	GGAACCAGAUCAUAAGGGAtt	UCCCUUAUGAUCUGGUUCCgg	195
MAP2K1	NM_002755	UGUUCAGUCUGGAAUUUCAtt	UGAAAUUCCAGACUGAACAct	56
MAP2K1	NM_002755	AGGCCUUUCUUACCCAGAAtt	UUCUGGGUAAGAAAGGCCUca	353
MAP2K1IP1	NM_021970	CAAACUUGGACUUUCCAAAtt	UUUGGAAAGUCCAAGUUUGct	93
MAP2K1IP1	NM_021970	CAAUUUAAUCGUUUACCUUtt	AAGGUAAACGAUUAAAUUGaa	215
MAP2K1IP1	NM_021970	GGUUCAAUUUAAUCGUUUAtt	UAAACGAUUAAAUUGAACCac	412
MAP2K2	NM_030662	GAUCAGCAUUUGCAUGGAAtt	UUCCAUGCAAAUGCUGAUCtc	95
MAP2K2	NM_030662	GAACUUGACGAGCAGCAGAtt	UCUGCUGCUCGUCAAGUUCca	56
MAP2K2	NM_030662	CAAAGACGAUGACUUCGAAtt	UUCGAAGUCAUCGUCUUUGag	230
MAP2K3	NM_002756	GGUCGACUGUUUCUACACUtt	AGUGUAGAAACAGUCGACCgt	189
MAP2K3	NM_002756	ACAGAAACUUUGAGGUGGAtt	UCCACCUCAAAGUUUCUGUct	78
MAP2K3	NM_002756	CCCGGACCUUCAUCACCAUtt	AUGGUGAUGAAGGUCCGGGag	172
MAP2K4	NM_003010	GGUAAACGCAAAGCACUGAtt	UCAGUGCUUUGCGUUUACCct	218
MAP2K4	NM_003010	GCAUGCAGGGUAAACGCAAtt	UUGCGUUUACCCUGCAUGCtg	99
MAP2K4	NM_003010	GCAACUGUGAAAGCACUAAtt	UUAGUGCUUUCACAGUUGCta	272
MAP2K5	NM_145160	GCCCUCCAAUAUGCUAGUAtt	UACUAGCAUAUUGGAGGGCtt	98
MAP2K5	NM_145160	GUAAUGGAACAGCAAGUAAtt	UUACUUGCUGUUCCAUUACtg	187
MAP2K5	NM_145160	GGCCUUACUUAUUUGUGGAtt	UCCACAAAUAAGUAAGGCCtt	265
MAP2K6	NM_002758	GCAUCACGAUGAUUGAGUUtt	AACUCAAUCAUCGUGAUGCcc	142
MAP2K6	NM_002758	GGAUACAUCACUAGAUAAAtt	UUUAUCUAGUGAUGUAUCCat	100

MAP2K6	NM_002758	GGUGGACUCUGUUGCUAAAtt	UUUAGCAACAGAGUCCACCaa	205
MAP2K7	NM_145185	CGUCAUUGCCGUUAAGCAAtt	UUGCUUAACGGCAAUGACGtg	143
MAP2K7	NM_145185	GACAGUUUCCCUACAAGAAtt	UUCUUGUAGGGAAACUGUCct	160
MAP2K7	NM_145185	GCAUUGAGAUUGACCAGAAtt	UUCUGGUCAAUCUCAAUGCtc	118
MAP3K1	XM_042066	GCGUAGCUCAAGGAUCAAAtt	UUUGAUCCUUGAGCUACGCct	131
MAP3K1	XM_042066	GGCUCUUCGUUGUUUAGAAtt	UUCUAAACAACGAAGAGCCac	142
MAP3K1	XM_042066	GAAUCUCCAGGAGUAAGGAtt	UCCUUACUCCUGGAGAUUCtg	278
MAP3K10	NM_002446	GGCUUUGAGCAUAAGAUCAtt	UGAUCUUAUGCUCAAAGCCag	127
MAP3K10	NM_002446	GGCUUGAAGUCAUCGAACAtt	UGUUCGAUGACUUCAAGCCgc	168
MAP3K10	NM_002446	GGUGCUUUCCCAAGACUGUtt	ACAGUCUUGGGAAAGCACCtg	281
MAP3K11	NM_002419	CGUGAUCUCAAGUCCAACAtt	UGUUGGACUUGAGAUCACGgt	151
MAP3K11	NM_002419	GCGUAGCUGUUAACAAGCUtt	AGCUUGUUAACAGCUACGCca	124
MAP3K11	NM_002419	CAUGGUACCUGGAUUCAGAtt	UCUGAAUCCAGGUACCAUGtg	36
MAP3K12	NM_006301	GCAUCUCAUCGGAAGAGGAtt	UCCUCUUCCGAUGAGAUGCca	106
MAP3K12	NM_006301	CCAACAUGCUAAUCACCUAtt	UAGGUGAUUAGCAUGUUGGgt	169
MAP3K12	NM_006301	CCAAUUCUGAGGACUCAGAtt	UCUGAGUCCUCAGAAUUGGga	233
MAP3K13	NM_004721	CGUGAACACUAUGAGCGGAtt	UCCGCUCAUAGUGUUCACGaa	102
MAP3K13	NM_004721	GCUCAAUCCCAAUACCCUUtt	AAGGGUAUUGGGAUUGAGCtt	259
MAP3K13	NM_004721	GAGUAAACCUCGAAACCGAtt	UCGGUUUCGAGGUUUACUCtg	375
MAP3K14	NM_003954	GUCCAAAUACAGUCUCUUAtt	UAAGAGACUGUAUUUGGACtt	117
MAP3K14	NM_003954	GGAUUGACCUCACCCAGAAtt	UUCUGGGUGAGGUCAAUCCtg	258
MAP3K14	NM_003954	GGAUUAUGAGUACCGAGAAtt	UUCUCGGUACUCAUAAUCCac	532
MAP3K15	NM_001001671	GGAUGUUCGCGAUGGACAAtt	UUGUCCAUCGCGAACAUCCag	147
MAP3K15	NM_001001671	CAAUCAAGUUUUACACCAAtt	UUGGUGUAAAACUUGAUUGtc	103
MAP3K15	NM_001001671	GGUUAUACACUUUCGGAUAtt	UAUCCGAAAGUGUAUAACCct	127
MAP3K2	XM_001128799	GGUCAUUCACUAGUAUCAAtt	UUGAUACUAGUGAAUGACCca	131
MAP3K2	XM_001128799	CCUCAUGUCUCAGACUAUAtt	UAUAGUCUGAGACAUGAGGtg	64
MAP3K2	XM_001128799	GUAUGAUGAUAGUCGAAUAtt	UAUUCGACUAUCAUCAUACtc	320
MAP3K3	NM_203351	CGAGGGCUAUGGAAGGAAAtt	UUUCCUUCCAUAGCCCUCGcc	123
MAP3K3	NM_203351	GGAAUACUCAGAUCGGGAAtt	UUCCCGAUCUGAGUAUUCCtg	230
MAP3K3	NM_203351	GCCGGCCUGUGAAAUAUGAtt	UCAUAUUUCACAGGCCGGCtg	164

MAP3K4	NM_005922	CUAACGAACUGAUCUGGUUtt	AACCAGAUCAGUUCGUUAGat	120
MAP3K4	NM_005922	GCUUCGCCUUUGUUAGAGAtt	UCUCUAACAAAGGCGAAGCtc	190
MAP3K4	NM_005922	CCUCGACAGAUGAAACGCAtt	UGCGUUUCAUCUGUCGAGGtg	115
MAP3K5	NM_005923	GCGAGUAGAUAAUAUCGAAtt	UUCGAUAUUAUCUACUCGCtg	146
MAP3K5	NM_005923	GGACCGCUUUUACAAUGCAtt	UGCAUUGUAAAAGCGGUCCag	99
MAP3K5	NM_005923	GGUGAACACCAUUACCGAAtt	UUCGGUAAUGGUGUUCACCat	230
MAP3K6	NM_004672	GAGCAGCUGUAUAAGCUCAtt	UGAGCUUAUACAGCUGCUCtg	65
MAP3K6	NM_004672	GCAAGGCUUUUGACGUAGAtt	UCUACGUCAAAAGCCUUGCga	190
MAP3K6	NM_004672	GGAACUGAAUGUGGAUUCAtt	UGAAUCCACAUUCAGUUCCtg	171
MAP3K7	NM_145332	CGUGUGAACCAUCCUAAUAtt	UAUUAGGAUGGUUCACACGgg	145
MAP3K7	NM_145332	AGAUACCAAUGGAUCAGAUtt	AUCUGAUCCAUUGGUAUCUgt	100
MAP3K7	NM_145332	GACUCGUUGUUGGUCUAAAtt	UUUAGACCAACAACGAGUCat	295
MAP3K7IP2	NM_015093	GAAUAAGUGAAACACGGAAtt	UUCCGUGUUUCACUUAUUCta	87
MAP3K7IP2	NM_015093	GGUUUUACAUGACCUGCGAtt	UCGCAGGUCAUGUAAAACCtg	156
MAP3K7IP2	NM_015093	GUCGAGCAAUAGGCAAUAAtt	UUAUUGCCUAUUGCUCGACtt	116
MAP3K8	NM_005204	CCGCAGACCUACUAAAACAtt	UGUUUUAGUAGGUCUGCGGct	111
MAP3K8	NM_005204	CCCUCCUACCUGUACAUAAtt	UUAUGUACAGGUAGGAGGGat	228
MAP3K8	NM_005204	GGAACUUCCUGAGAACAUUtt	AAUGUUCUCAGGAAGUUCCag	237
MAP3K9	NM_033141	GCGAUGAAAUUGUCGUGUAtt	UACACGACAAUUUCAUCGCtg	172
MAP3K9	NM_033141	GACCAUCUUUCACGAAUAUtt	AUAUUCGUGAAAGAUGGUCgt	146
MAP3K9	NM_033141	CAAACGAGAUCCUAACCAAtt	UUGGUUAGGAUCUCGUUUGaa	60
MAP4K1	NM_001042600	GGGACAUCAAGGGAGCUAAtt	UUAGCUCCCUUGAUGUCCCtg	98
MAP4K1	NM_001042600	CCAAGAUGCUCAGUCAUCAtt	UGAUGACUGAGCAUCUUGGtg	206
MAP4K1	NM_001042600	GCUCAGUCAUCAACUGGUAtt	UACCAGUUGAUGACUGAGCat	185
MAP4K2	NM_004579	CCAAGAUUCCUGACACCAAtt	UUGGUGUCAGGAAUCUUGGtg	176
MAP4K2	NM_004579	CCUAUGACAUGUUUCCAGAtt	UCUGGAAACAUGUCAUAGGtc	165
MAP4K2	NM_004579	GCAGCUACCUCAGGAAUGAtt	UCAUUCCUGAGGUAGCUGCca	174
MAP4K3	NM_003618	GCACUUACCAAAAAUCCGAtt	UCGGAUUUUUGGUAAGUGCca	189
MAP4K3	NM_003618	CAAUCGAGCUGUUGGAUAAtt	UUAUCCAACAGCUCGAUUGcc	221
MAP4K3	NM_003618	GGCACGGAAUGUUAACACUtt	AGUGUUAACAUUCCGUGCCtt	157
MAP4K4	NM_145687	CCUAUGGACAAGUCUAUAAtt	UUAUAGACUUGUCCAUAGGtg	135

MAP4K4	NM_145687	CGGCUAGAAGAGCAACAAAtt	UUUGUUGCUCUUCUAGCCGtc	168
MAP4K4	NM_145687	CGAAGACGAUUUCAACAAAtt	UUUGUUGAAAUCGUCUUCGgt	191
MAP4K5	NM_006575	GAGUUAUCUUAGUCGGGAAtt	UUCCCGACUAAGAUAACUCcc	92
MAP4K5	NM_006575	CUACCAUUGCAAAACGAAAtt	UUUCGUUUUGCAAUGGUAGct	158
MAP4K5	NM_006575	GCAAAGGCACUGAAUCGAAtt	UUCGAUUCAGUGCCUUUGCta	225
MAPK1	NM_138957	CAGGGUUCCUGACAGAAUAtt	UAUUCUGUCAGGAACCCUGtg	271
MAPK1	NM_138957	CAACCAUCGAGCAAAUGAAtt	UUCAUUUGCUCGAUGGUUGgt	63
MAPK1	NM_138957	GCAGAAAUGCUUUCUAACAtt	UGUUAGAAAGCAUUUCUGCca	464
MAPK10	NM_138981	GGAAUAAGGUAAUUGAACAtt	UGUUCAAUUACCUUAUUCCac	127
MAPK10	NM_138981	CAAGUAACAUUGUAGUCAAtt	UUGACUACAAUGUUACUUGgt	73
MAPK10	NM_138981	CCAUAUGUGGUGACACGUUtt	AACGUGUCACCACAUAUGGag	108
MAPK11	NM_002751	GCGACUACAUUGACCAGCUtt	AGCUGGUCAAUGUAGUCGCtt	135
MAPK11	NM_002751	GAACACGCCCGGACAUAUAtt	UAUAUGUCCGGGCGUGUUCtg	205
MAPK11	NM_002751	GGAGCUCACUUACCAGGAAtt	UUCCUGGUAAGUGAGCUCCtt	208
MAPK12	NM_002969	CAGUUCCUCGUGUACCAGAtt	UCUGGUACACGAGGAACUGga	143
MAPK12	NM_002969	GAAGUAUGAUGACUCCUUUtt	AAAGGAGUCAUCAUACUUCtg	106
MAPK12	NM_002969	GCAAGCUCAUGAAACAUGAtt	UCAUGUUUCAUGAGCUUGCcc	119
MAPK13	NM_002754	AAUGAGGACUGUGAACUGAtt	UCAGUUCACAGUCCUCAUUca	93
MAPK13	NM_002754	GGUGUAUCAGAUGCUCAAAtt	UUUGAGCAUCUGAUACACCag	58
MAPK13	NM_002754	GCACAUCUACAAGGAGAUUtt	AAUCUCCUUGUAGAUGUGCtg	155
MAPK14	NM_001315	CCUAAAACCUAGUAAUCUAtt	UAGAUUACUAGGUUUUAGGtc	162
MAPK14	NM_001315	GAAGCUCUCCAGACCAUUUtt	AAAUGGUCUGGAGAGCUUCtt	211
MAPK14	NM_001315	CUGCGGUUACUUAAACAUAtt	UAUGUUUAAGUAACCGCAGtt	87
MAPK15	NM_139021	AGAACGACAGGGACAUUUAtt	UAAAUGUCCCUGUCGUUCUct	98
MAPK15	NM_139021	UGAACGCAGUCAUCCGGAAtt	UUCCGGAUGACUGCGUUCAgg	92
MAPK15	NM_139021	AGAACAUUCCGGGAAAUCAtt	UGAUUUCCCGGAAUGUUCUct	139
MAPK3	NM_001040056	GGAUCAGCUCAACCACAUUtt	AAUGUGGUUGAGCUGAUCCag	46
MAPK3	NM_001040056	GGACCGGAUGUUAACCUUUtt	AAAGGUUAACAUCCGGUCCag	71
MAPK3	NM_001040056	GACCUGAAUUGUAUCAUCAtt	UGAUGAUACAAUUCAGGUCct	160
MAPK4	NM_002747	GGAUCGUUGAUCAGCAUUAtt	UAAUGCUGAUCAACGAUCCtt	123
MAPK4	NM_002747	CAGAAGGGUUGGUAACAAAtt	UUUGUUACCAACCCUUCUGac	127

MAPK4	NM_002747	CCACAAGGGUUAUCUGUCAtt	UGACAGAUAACCCUUGUGGga	192
MAPK6	NM_002748	CCAUCCUUACAUGAGCAUAtt	UAUGCUCAUGUAAGGAUGGga	164
MAPK6	NM_002748	CUAGGUAUAUGGACUUAAAtt	UUUAAGUCCAUAUACCUAGaa	150
MAPK6	NM_002748	CUACUGAUGUUGUUGAUAAtt	UUAUCAACAACAUCAGUAGgc	176
MAPK7	NM_139034	GCUGAACAUCAGUACUUCAtt	UGAAGUACUGAUGUUCAGCgg	145
MAPK7	NM_139034	GAGGAAUUCUUAAACCAGUtt	ACUGGUUUAAGAAUUCCUCca	130
MAPK7	NM_139034	CCAUUGAUCUGACCCUGCAtt	UGCAGGGUCAGAUCAAUGGtg	94
MAPK8	NM_002750	GUUGCAAUCAAGAAGCUAAtt	UUAGCUUCUUGAUUGCAACat	118
MAPK8	NM_002750	CAAAGAUCCCUGACAAGCAtt	UGCUUGUCAGGGAUCUUUGgt	81
MAPK8	NM_002750	CCAGUAAUAUAGUAGUAAAtt	UUUACUACUAUAUUACUGGgc	141
MAPK9	NM_002752	GAAUCUGAGCGAGACAAAAtt	UUUUGUCUCGCUCAGAUUCtg	152
MAPK9	NM_002752	CUUACGUGGUGACACGGUAtt	UACCGUGUCACCACGUAAGgg	95
MAPK9	NM_002752	GCGGAUCUCUGUAGACGAAtt	UUCGUCUACAGAGAUCCGCtt	155
MAPKAPK2	NM_004759	GGAUCAUGCAAUCAACAAAtt	UUUGUUGAUUGCAUGAUCCaa	148
MAPKAPK2	NM_004759	CAGUAUCUGCAUUCAAUCAtt	UGAUUGAAUGCAGAUACUGga	314
MAPKAPK2	NM_004759	GGACGGUGGAGAACUCUUUtt	AAAGAGUUCUCCACCGUCCaa	90
MAPKAPK3	NM_004635	GGUGGUGAGUUGUUCAGCAtt	UGCUGAACAACUCACCACCtt	138
MAPKAPK3	NM_004635	UGUCAAGCCUGAAAACCUAtt	UAGGUUUUCAGGCUUGACAtc	130
MAPKAPK3	NM_004635	ACCCUGCUAUACUCCCUAUtt	AUAGGGAGUAUAGCAGGGUgt	248
MAPKAPK5	NM_003668	CCACGACCAUGAGAAUGGAtt	UCCAUUCUCAUGGUCGUGGat	64
MAPKAPK5	NM_003668	GAAUGGUCGUGGAUUCACAtt	UGUGAAUCCACGACCAUUCca	105
MAPKAPK5	NM_003668	CACCGGCACUUUACAGAGAtt	UCUCUGUAAAGUGCCGGUGct	368
MARK1	NM_018650	GGUGGUGAAUCGUUAUCCAtt	UGGAUAACGAUUCACCACCtt	89
MARK1	NM_018650	GCACAACAGUUGGAUCAAAtt	UUUGAUCCAACUGUUGUGCtg	128
MARK1	NM_018650	GAAUCAUCCUAAUAUAGGUtt	ACCUAUAUUAGGAUGAUUCag	276
MARK2	NM_001039468	GCCUAGGAGUUAUCCUCUAtt	UAGAGGAUAACUCCUAGGCtc	147
MARK2	NM_001039468	GAAUCAUCCCAACAUAGUUtt	AACUAUGUUGGGAUGAUUCaa	143
MARK2	NM_001039468	GACUCAGAGUAACAACGCAtt	UGCGUUGUUACUCUGAGUCtt	167
MARK3	NM_002376	CCAUUGUCCCAGACUCGAAtt	UUCGAGUCUGGGACAAUGGtg	151
MARK3	NM_002376	GCGGUAAACUCGACACGUUtt	AACGUGUCGAGUUUACCGCca	179
MARK3	NM_002376	CGGAUAUUCCUGAACGCAAtt	UUGCGUUCAGGAAUAUCCGcc	113

MARK4	NM_031417	GCAGAACUCUAACCGCUGUtt	ACAGCGGUUAGAGUUCUGCcg	188
MARK4	NM_031417	GCACUAUUGUCACCAGAAAtt	UUUCUGGUGACAAUAGUGCac	641
MARK4	NM_031417	CACCUACGUUUGCACAGAAtt	UUCUGUGCAAACGUAGGUGtt	79
MAST1	NM_014975	CCGCACCAGUAAUCGGAAAtt	UUUCCGAUUACUGGUGCGGca	193
MAST1	NM_014975	CCAUCAAGCUCAUAAGCAAtt	UUGCUUAUGAGCUUGAUGGta	492
MAST1	NM_014975	GUGAAGAAGUUGCUUAUUAtt	UAAUAAGCAACUUCUUCACca	394
MAST2	NM_015112	CGUCUAUACUUUGCGGAAAtt	UUUCCGCAAAGUAUAGACGca	243
MAST2	NM_015112	GAUACUAGCUAUUUUGACAtt	UGUCAAAAUAGCUAGUAUCat	249
MAST2	NM_015112	GUAUGAGGGUCAUAUUGAAtt	UUCAAUAUGACCCUCAUACaa	185
MAST3	XM_038150	GACCUUCCACAGUACAUCAtt	UGAUGUACUGUGGAAGGUCag	126
MAST3	XM_038150	GAAGAGCGGCAACAAGAUAtt	UAUCUUGUUGCCGCUCUUCag	220
MAST3	XM_038150	GGAGUACCUGCAUAACUAUtt	AUAGUUAUGCAGGUACUCCag	188
MASTL	NM_032844	GAGUAUAAGCAUAACGAAAtt	UUUCGUUAUGCUUAUACUCta	152
MASTL	NM_032844	GGACAAGUGUUAUCGCUUAtt	UAAGCGAUAACACUUGUCCtg	169
MASTL	NM_032844	GCCCUUAGAUUCAGAUAGAtt	UCUAUCUGAAUCUAAGGGCtg	192
MATK	NM_002378	CGAUAAAGACGGAUUCUAAtt	UUAGAAUCCGUCUUUAUCGgg	319
MATK	NM_002378	AGUGUGAUGUGACAGCCCAtt	UGGGCUGUCACAUCACACUtg	470
MATK	NM_002378	AGACGGCCGUCAUGACGAAtt	UUCGUCAUGACGGCCGUCUcg	105
MELK	NM_014791	GGUUGUCUUCCGUCAGAUAtt	UAUCUGACGGAAGACAACCcg	166
MELK	NM_014791	GAAACGGAUUUCUAUGAAAtt	UUUCAUAGAAAUCCGUUUCtt	185
MELK	NM_014791	CCUGGAUCAUGCAAGAUUAtt	UAAUCUUGCAUGAUCCAGGga	164
MERTK	NM_006343	GAACUUACCUUACAUAGCUtt	AGCUAUGUAAGGUAAGUUCaa	129
MERTK	NM_006343	CAGUAGCCGUGUUAACGAAtt	UUCGUUAACACGGCUACUGtt	196
MERTK	NM_006343	GGAUGAAGCCUCCGACUAAtt	UUAGUCGGAGGCUUCAUCCat	110
MET	NM_000245	GCACUAGCAAAGUCCGAGAtt	UCUCGGACUUUGCUAGUGCct	160
MET	NM_000245	CACCUUAUCCUGACGUAAAtt	UUUACGUCAGGAUAAGGUGgg	175
MET	NM_000245	GCUACUUAUGUGAACGUAAtt	UUACGUUCACAUAAGUAGCgt	216
MFHAS1	NM_004225	GCAGAUUGAAUAUAGCUUUtt	AAAGCUAUAUUCAAUCUGCaa	148
MFHAS1	NM_004225	GCCUCAUGCUAGACAACAAtt	UUGUUGUCUAGCAUGAGGCtc	166
MFHAS1	NM_004225	CAGGUCGGAUGGUAAAUUUtt	AAAUUUACCAUCCGACCUGtg	354
MGC42105	NM_153361	GAAUGAUGAAUGGAUGCAAtt	UUGCAUCCAUUCAUCAUUCat	82

MGC42105	NM_153361	CACAUGCAUGAAAACCAAAtt	UUUGGUUUUCAUGCAUGUGct	104
MGC42105	NM_153361	GCAUAUUCGAAAUAACCAAtt	UUGGUUAUUUCGAAUAUGCtc	68
MINK1	NM_153827	CGUACGGGCGCAUCAUUAAtt	UUAAUGAUGCGCCCGUACGtg	102
MINK1	NM_153827	GGAGGACUGUAUCGCCUAUtt	AUAGGCGAUACAGUCCUCCtt	136
MINK1	NM_153827	GAAUCCCGCUGACAAACCAtt	UGGUUUGUCAGCGGGAUUCat	134
MKNK1	NM_003684	GCCCUUGACUUCCUGCAUAtt	UAUGCAGGAAGUCAAGGGCag	134
MKNK1	NM_003684	CCGUCAAAAUCAUCGAGAAtt	UUCUCGAUGAUUUUGACGGca	245
MKNK1	NM_003684	GGAGUAGGGUGUUUCGAGAtt	UCUCGAAACACCCUACUCCga	384
MKNK2	NM_199054	GGAAUUUUGUAUUCUGUUUtt	AAACAGAAUACAAAAUUCCgg	151
MKNK2	NM_199054	GCAGCGGCAUCAAACUCAAtt	UUGAGUUUGAUGCCGCUGCcc	85
MKNK2	NM_199054	GCCUUGGACUUUCUGCAUAtt	UAUGCAGAAAGUCCAAGGCgc	225
MLCK	NM_182493	UGAACAUGCUGAACGAGAAtt	UUCUCGUUCAGCAUGUUCAgc	88
MLCK	NM_182493	GGAUCUCCAUCCACAUACAtt	UGUAUGUGGAUGGAGAUCCtt	63
MLCK	NM_182493	GACCAUGAAUUUCAUUGUAtt	UACAAUGAAAUUCAUGGUCtc	136
MLKL	NM_152649	GCUAAGAAGAGAUAAUGAAtt	UUCAUUAUCUCUUCUUAGCat	118
MLKL	NM_152649	CGAUUAGAAAUCAACAUGAtt	UCAUGUUGAUUUCUAAUCGtc	124
MLKL	NM_152649	CGUGGAUUCUGCUAAGGGAtt	UCCCUUAGCAGAAUCCACGgg	185
MORN1	NM_024848	CACGGGAAGUUGUUAUUUAtt	UAAAUAACAACUUCCCGUGac	145
MORN1	NM_024848	CACCUUCUCUGGACAGUUUtt	AAACUGUCCAGAGAAGGUGtc	157
MORN1	NM_024848	CGAUAUGAAGGAGAAUGGAtt	UCCAUUCUCCUUCAUAUCGaa	131
MOS	NM_005372	GGACAGUUAAGUUUGGGAAtt	UUCCCAAACUUAACUGUCCtc	145
MOS	NM_005372	GCCUAAAGCCGACAUUUAUtt	AUAAAUGUCGGCUUUAGGCgt	231
MOS	NM_005372	CGGUGUACAAGGCGACUUAtt	UAAGUCGCCUUGUACACCGag	229
MPP1	NM_002436	GGAGUACCACUUUAUCUCAtt	UGAGAUAAAGUGGUACUCCtt	157
MPP1	NM_002436	GCUCGAUUUUUGAUCAGUUtt	AACUGAUCAAAAAUCGAGCtg	166
MPP1	NM_002436	CAGUGCACCAGAUCCAUAAtt	UUAUGGAUCUGGUGCACUGtt	188
MPP2	NM_005374	CGGCCGAGUUUGUCCCUUAtt	UAAGGGACAAACUCGGCCGtt	131
MPP2	NM_005374	AGGUCAGGGUUACAGCUUUtt	AAAGCUGUAACCCUGACCUtc	188
MPP2	NM_005374	UGAUGUAUUUGACCACCAAtt	UUGGUGGUCAAAUACAUCAtt	177
MPP3	NM_001932	GAAGAUCGCUUAAAGGAGAtt	UCUCCUUUAAGCGAUCUUCct	135
MPP3	NM_001932	CAAUAUCGAUGAGGAUUUUtt	AAAAUCCUCAUCGAUAUUGtc	83

MPP3	NM_001932	GAAGCUUCGCUAUUAUGAAtt	UUCAUAAUAGCGAAGCUUCtc	104
MPP4	NM_033066	CCCUAUCAAUUUCUAUGGAtt	UCCAUAGAAAUUGAUAGGGtt	196
MPP4	NM_033066	GGUGUUAUCCUAUGAGGUAtt	UACCUCAUAGGAUAACACCtg	115
MPP4	NM_033066	GCUAUAUGCUGGAGACAAAtt	UUUGUCUCCAGCAUAUAGCaa	128
MPP5	NM_022474	GAAUCAGACAGCGAAGUAAtt	UUACUUCGCUGUCUGAUUCct	219
MPP5	NM_022474	CAUAGAUUCUGUACGGCAAtt	UUGCCGUACAGAAUCUAUGct	138
MPP5	NM_022474	CGAUCUUGAUAAAGCCUAUtt	AUAGGCUUUAUCAAGAUCGga	125
MPP6	NM_016447	CCUAAUACCUUGCAAAGAAtt	UUCUUUGCAAGGUAUUAGGtt	102
MPP6	NM_016447	CAUUACUCCUCAACAGGUAtt	UACCUGUUGAGGAGUAAUGgt	84
MPP6	NM_016447	GGACAAUUCAGGACCUUUUtt	AAAAGGUCCUGAAUUGUCCca	104
MPP7	NM_173496	CCGUAUCGGCGACAAACUAtt	UAGUUUGUCGCCGAUACGGtg	78
MPP7	NM_173496	GGCAAUUCCAUGUAAGGAAtt	UUCCUUACAUGGAAUUGCCtt	130
MPP7	NM_173496	GGAUACCAGUGGAGGAUAAtt	UUAUCCUCCACUGGUAUCCcg	83
MRC2	NM_006039	CCUGCCCAAUGUGACCUUUtt	AAAGGUCACAUUGGGCAGGct	125
MRC2	NM_006039	GUAUGAGUGUGACCGGGAAtt	UUCCCGGUCACACUCAUACat	249
MRC2	NM_006039	GAUCUUCGGUGAAUCAGAAtt	UUCUGAUUCACCGAAGAUCtt	95
MST1R	NM_002447	GGCCCAGAAUCGAAUCCAAtt	UUGGAUUCGAUUCUGGGCCtg	119
MST1R	NM_002447	GGACAGCUCAAAACUCAGAtt	UCUGAGUUUUGAGCUGUCCtt	68
MST1R	NM_002447	GCGUAGAUGGUGAAUGUCAtt	UGACAUUCACCAUCUACGCag	166
MUSK	NM_005592	GUAAGUUUGUUCACCGAGAtt	UCUCGGUGAACAAACUUACgt	153
MUSK	NM_005592	GCAUAGCAGAAUGGAGUAAtt	UUACUCCAUUCUGCUAUGCtg	182
MUSK	NM_005592	GCUUACCUCUCAGAACGUAtt	UACGUUCUGAGAGGUAAGCca	212
MVK	NM_000431	GGGAAGAUUUCAUCCUUAAtt	UUAAGGAUGAAAUCUUCCCtt	162
MVK	NM_000431	GCAAGGUGAUGUCACAACAtt	UGUUGUGACAUCACCUUGCtc	155
MVK	NM_000431	GGAGCAAGGUGAUGUCACAtt	UGUGACAUCACCUUGCUCCag	160
MYLK	NM_053028	GCCUCAUGUAAAACCCUAUtt	AUAGGGUUUUACAUGAGGCtt	104
MYLK	NM_053028	GGACGGGAACUGCUCUUUAtt	UAAAGAGCAGUUCCCGUCCtc	168
MYLK	NM_053028	GACCAUUCGCGAUUUAGAAtt	UUCUAAAUCGCGAAUGGUCtt	303
MYLK2	NM_033118	GGGCAUUUGGUGAAGAUCAtt	UGAUCUUCACCAAAUGCCCgg	104
MYLK2	NM_033118	GGUGGUGAAUUAUGACCAAtt	UUGGUCAUAAUUCACCACCtc	150
MYLK2	NM_033118	CCAAAUCUCCGAUAAGACAtt	UGUCUUAUCGGAGAUUUGGtc	144

NADK	NM_023018	AGGAGAACAUGAUCGUGUAtt	UACACGAUCAUGUUCUCCUcc	160
NADK	NM_023018	CGUGUAUGUGGAAAAGAAAtt	UUUCUUUUCCACAUACACGat	162
NADK	NM_023018	GUACCUUUCGAGAAGAUUAtt	UAAUCUUCUCGAAAGGUACag	100
NAGK	NM_017567	GGCUAGGGAUACUCACUCAtt	UGAGUGAGUAUCCCUAGCCga	181
NAGK	NM_017567	GCUACUUAAUCACCACCGAtt	UCGGUGGUGAUUAAGUAGCtt	212
NAGK	NM_017567	GAUCGGCUAGGGAUACUCAtt	UGAGUAUCCCUAGCCGAUCtg	223
NEK1	NM_012224	GAUUGAAGGCGGUGUCUAAtt	UUAGACACCGCCUUCAAUCtt	254
NEK1	NM_012224	CGUAGGAGAUGUUCGUCAAtt	UUGACGAACAUCUCCUACGgt	180
NEK1	NM_012224	CCGUAGGAGAUGUUCGUCAtt	UGACGAACAUCUCCUACGGtg	267
NEK10	NM_001031741	GAAUUGGACAUUUCGGAUAtt	UAUCCGAAAUGUCCAAUUCat	113
NEK10	NM_001031741	UCAGUUCGAUGAUAUCAGAtt	UCUGAUAUCAUCGAACUGAct	103
NEK10	NM_001031741	GCUAGAACGGGAACGAAGAtt	UCUUCGUUCCCGUUCUAGCtt	134
NEK11	NM_024800	GCAUGCAUUUUGUAUGAGAtt	UCUCAUACAAAAUGCAUGCca	210
NEK11	NM_024800	CCACGAACUUGAAUCAAUUtt	AAUUGAUUCAAGUUCGUGGag	142
NEK11	NM_024800	GAUACGUGCUUCAACAAAAtt	UUUUGUUGAAGCACGUAUCtt	119
NEK2	NM_002497	GGCGAAUUCCAUACCGUUAtt	UAACGGUAUGGAAUUCGCCtg	273
NEK2	NM_002497	CAUCGUUCGUUACUAUGAUtt	AUCAUAGUAACGAACGAUGtt	198
NEK2	NM_002497	GCUUGCUAAAGGAACGGAAtt	UUCCGUUCCUUUAGCAAGCtg	196
NEK3	NM_002498	CUUAAUUGGUUUACCCAAAtt	UUUGGGUAAACCAAUUAAGta	199
NEK3	NM_002498	GGCAUUUGCUUGUACCUAUtt	AUAGGUACAAGCAAAUGCCat	99
NEK3	NM_002498	CAUCCAUUUCAGGCAAAUAtt	UAUUUGCCUGAAAUGGAUGct	146
NEK4	NM_003157	GCGUAAAUAUUGACAUCUUtt	AAGAUGUCAAUAUUUACGCta	85
NEK4	NM_003157	GAAUCCACCCAGAUUUACAtt	UGUAAAUCUGGGUGGAUUCtg	122
NEK4	NM_003157	CCAACAUUGUCACCUACAAtt	UUGUAGGUGACAAUGUUGGga	155
NEK5	NM_199289	CCUACAACAAUAAAACGGAtt	UCCGUUUUAUUGUUGUAGGgt	96
NEK5	NM_199289	CUACAACAAUAAAACGGAUtt	AUCCGUUUUAUUGUUGUAGgg	79
NEK5	NM_199289	CGAGACCGACCAUCCAUAAtt	UUAUGGAUGGUCGGUCUCGag	138
NEK6	NM_014397	GCACUACUCCGAGAAGUUAtt	UAACUUCUCGGAGUAGUGCtc	69
NEK6	NM_014397	CAACUGAACCACCCAAAUAtt	UAUUUGGGUGGUUCAGUUGct	120
NEK6	NM_014397	GGCUGUCUGCUGUACGAGAtt	UCUCGUACAGCAGACAGCCca	328
NEK7	NM_133494	GACCGGAUAUGGGCUAUAAtt	UUAUAGCCCAUAUCCGGUCgt	186

NEK7	NM_133494	CCAGAAUGAUCAAGCAUUUtt	AAAUGCUUGAUCAUUCUGGat	72
NEK7	NM_133494	GCCGGUUUUUCAGCUCAAAtt	UUUGAGCUGAAAAACCGGCca	99
NEK8	NM_178170	GGGUGAUUGCUACACUUUUtt	AAAAGUGUAGCAAUCACCCga	132
NEK8	NM_178170	CCAAGAUCCUUAGCAGCAAtt	UUGCUGCUAAGGAUCUUGGag	158
NEK8	NM_178170	CCUUUGCACCUAUCUCUGAtt	UCAGAGAUAGGUGCAAAGGtg	252
NEK9	NM_033116	GGACUCAAUGAAUUCAAUAtt	UAUUGAAUUCAUUGAGUCCac	103
NEK9	NM_033116	GCUUAUCCAUUGGAACUGUtt	ACAGUUCCAAUGGAUAAGCca	79
NEK9	NM_033116	GCUGGAAUAUUGUAAUGGAtt	UCCAUUACAAUAUUCCAGCtc	90
NLK	NM_016231	CCAGAAAUCCUGAUGGGCAtt	UGCCCAUCAGGAUUUCUGGag	238
NLK	NM_016231	GGUGUUGUCUGGUCAGUAAtt	UUACUGACCAGACAACACCaa	289
NLK	NM_016231	CCAAAAGAAUAUCCGCUAAtt	UUAGCGGAUAUUCUUUUGGat	537
NME1	NM_000269	GCUUGUGGUUUCACCCUGAtt	UCAGGGUGAAACCACAAGCcg	67
NME1	NM_000269	GCAUAGGAUUCAUUGAGUUtt	AACUCAAUGAAUCCUAUGCtg	191
NME1	NM_000269	CUGAGGAACUGGUAGAUUAtt	UAAUCUACCAGUUCCUCAGgg	204
NME2	NM_001018138	GCACUACAUUGACCUGAAAtt	UUUCAGGUCAAUGUAGUGCtg	128
NME2	NM_001018138	GCCUAUGGUUUAAGCCUGAtt	UCAGGCUUAAACCAUAGGCtg	187
NME2	NM_001018138	GAAAUCAGCCUAUGGUUUAtt	UAAACCAUAGGCUGAUUUCtt	169
NME3	NM_002513	GCAUCGAGGUUGGCAAGAAtt	UUCUUGCCAACCUCGAUGCag	90
NME3	NM_002513	GCGCUUGGAGCAUCCCUUUtt	AAAGGGAUGCUCCAAGCGCtg	63
NME3	NM_002513	CCAACGUGGUCCAACGUUUtt	AAACGUUGGACCACGUUGGgc	167
NME4	NM_005009	AGCACAAGAUUGGACCAAUtt	AUUGGUCCAAUCUUGUGCUgg	121
NME4	NM_005009	AGAUUGGACCAAUCCUUUUtt	AAAAGGAUUGGUCCAAUCUtg	173
NME4	NM_005009	GCAAGAACCCAAGCCCACAtt	UGUGGGCUUGGGUUCUUGCtg	188
NME5	NM_003551	CAGAUAUUGUUGACAAAGAtt	UCUUUGUCAACAAUAUCUGgt	186
NME5	NM_003551	GAACUUUUGGGACCAAAUAtt	UAUUUGGUCCCAAAAGUUCta	126
NME5	NM_003551	GCUAAGGACUAUUUAAAUUtt	AAUUUAAAUAGUCCUUAGCag	145
NME6	NM_005793	GGAGUUUCGGCCUCACUGAtt	UCAGUGAGGCCGAAACUCCca	142
NME6	NM_005793	GUUUUACCGAGAGCAUGAAtt	UUCAUGCUCUCGGUAAAACct	118
NME6	NM_005793	AGUUCCUGAUUGUACGAAUtt	AUUCGUACAAUCAGGAACUtg	387
NME7	NM_013330	CUGGAUUUACUAUAACCAAtt	UUGGUUAUAGUAAAUCCAGct	137
NME7	NM_013330	CAAUGGAGAUUCAACAGAAtt	UUCUGUUGAAUCUCCAUUGct	112

NME7	NM_013330	GGGUUAAUGUUGAGGAAUUtt	AAUUCCUCAACAUUAACCCga	178
NRBP1	NM_013392	CCAUUACAGAGGGAGUUCAtt	UGAACUCCCUCUGUAAUGGgt	73
NRBP1	NM_013392	GACGAUGAAUGAAAAGGCAtt	UGCCUUUUCAUUCAUCGUCtt	182
NRBP1	NM_013392	CCAGCUCUGGAAUUAGAUAtt	UAUCUAAUUCCAGAGCUGGtg	183
NRBP2	NM_178564	GGAAUGGAAUCUACCCACUtt	AGUGGGUAGAUUCCAUUCCtg	114
NRBP2	NM_178564	GGCUCAGUCUUAGAGAUUUtt	AAAUCUCUAAGACUGAGCCag	83
NRBP2	NM_178564	AGAGAUUUCUAUGCCCUCAtt	UGAGGGCAUAGAAAUCUCUaa	36
NRGN	NM_006176	CACACUCACUUAAAGAAAAtt	UUUUCUUUAAGUGAGUGUGct	143
NRGN	NM_006176	GCCGGACGACGACAUUCUAtt	UAGAAUGUCGUCGUCCGGCtt	239
NRGN	NM_006176	CCCAAGCACACUCACUUAAtt	UUAAGUGAGUGUGCUUGGGtg	206
NRK	NM_198465	CUUAUACGCUGGAUUCGUAtt	UACGAAUCCAGCGUAUAAGat	141
NRK	NM_198465	GGAGUUCGCAAAAUCGUCAtt	UGACGAUUUUGCGAACUCCtt	126
NRK	NM_198465	CUGUAUACUUGACAAACGAtt	UCGUUUGUCAAGUAUACAGgg	135
NTRK1	NM_001007792	GAGAGCAUCCUGUACCGUAtt	UACGGUACAGGAUGCUCUCgg	132
NTRK1	NM_001007792	GCUCCUUGUGCUCAACAAAtt	UUUGUUGAGCACAAGGAGCag	168
NTRK1	NM_001007792	CAACAAAUGUGGACGGAGAtt	UCUCCGUCCACAUUUGUUGag	276
NTRK2	NM_001018065	GCUCCUUAAGGAUAACUAAtt	UUAGUUAUCCUUAAGGAGCcc	111
NTRK2	NM_001018065	GAAUUGACGAUGGUGCAAAtt	UUUGCACCAUCGUCAAUUCca	74
NTRK2	NM_001018065	GGUUAGAAAUCAUCAACGAtt	UCGUUGAUGAUUUCUAACCtt	146
NTRK3	NM_002530	GGAAUAUCACUUCCAUACAtt	UGUAUGGAAGUGAUAUUCCtt	134
NTRK3	NM_002530	CGGAUAACUUUAUCUUGUUtt	AACAAGAUAAAGUUAUCCGtg	58
NTRK3	NM_002530	GAUGUGGACUGGAUAGUCAtt	UGACUAUCCAGUCCACAUCag	135
NUAK1	NM_014840	GCCCUUCGAUGGUUUCGAUtt	AUCGAAACCAUCGAAGGGCat	321
NUAK1	NM_014840	CCAGUGUCAUCAGCGAUGAtt	UCAUCGCUGAUGACACUGGaa	285
NUAK1	NM_014840	CACUCUUGUUUAUGGAACAtt	UGUUCCAUAAACAAGAGUGta	202
NUAK2	NM_030952	CAACCACCCUCACAUCAUUtt	AAUGAUGUGAGGGUGGUUGag	117
NUAK2	NM_030952	GCAUGACCAUAAGAUCCUAtt	UAGGAUCUUAUGGUCAUGCcc	71
NUAK2	NM_030952	GCAAGAUCUGAUGCACAUAtt	UAUGUGCAUCAGAUCUUGCtc	158
OXSR1	NM_005109	CGAGUGAAAGAAUCAAUAUtt	AUAUUGAUUCUUUCACUCGgg	102
OXSR1	NM_005109	GAACCUCAGUCAAAUCGAUtt	AUCGAUUUGACUGAGGUUCtt	341
OXSR1	NM_005109	CCAAGAUCCCAAUCAGUCUtt	AGACUGAUUGGGAUCUUGGtt	177

PACSIN1	NM_020804	CACCUGAGCAGCAAAAGAAtt	UUCUUUUGCUGCUCAGGUGtg	111
PACSIN1	NM_020804	AGCAAUCGGUCACACCUGAtt	UCAGGUGUGACCGAUUGCUcc	333
PACSIN1	NM_020804	CGUCUAUGCAACGACCUGAtt	UCAGGUCGUUGCAUAGACGgt	83
PACSIN2	NM_007229	ACCCAUCCUUCAACCCUGAtt	UCAGGGUUGAAGGAUGGGUct	197
PACSIN2	NM_007229	GGAGGUUCAGAAGCACCUAtt	UAGGUGCUUCUGAACCUCCag	182
PACSIN2	NM_007229	AGAUGUUCUUAAGACCAAAtt	UUUGGUCUUAAGAACAUCUtg	342
PACSIN3	NM_016223	GGAUGAGGAGUGGUCAGAUtt	AUCUGACCACUCCUCAUCCtg	51
PACSIN3	NM_016223	CAAGAAAAGCUACCACGCAtt	UGCGUGGUAGCUUUUCUUGga	109
PACSIN3	NM_016223	CAAUCAGCCGGAAAGAGAAtt	UUCUCUUUCCGGCUGAUUGtc	111
PAK1	NM_002576	GCUCUGUCAAGCUAACUGAtt	UCAGUUAGCUUGACAGAGCca	226
PAK1	NM_002576	CAGAGGUUGUGACACGAAAtt	UUUCGUGUCACAACCUCUGgt	222
PAK1	NM_002576	GAAGGACCGAUUUUACCGAtt	UCGGUAAAAUCGGUCCUUCtt	163
PAK2	XM_001126110	GAACUGAUCAUUAACGAGAtt	UCUCGUUAAUGAUCAGUUCct	120
PAK2	XM_001126110	GGUGAUGAAAGAAUUGAAAtt	UUUCAAUUCUUUCAUCACCag	121
PAK2	XM_001126110	CAGAGGUGGUUACACGGAAtt	UUCCGUGUAACCACCUCUGgt	92
PAK3	NM_002578	CGAUUACUCCAAACUUCCAtt	UGGAAGUUUGGAGUAAUCGtg	105
PAK3	NM_002578	CAACCCAAGAAGGAAUUAAtt	UUAAUUCCUUCUUGGGUUGct	175
PAK3	NM_002578	GCUACUUGGUGGGUGAUGAtt	UCAUCACCCACCAAGUAGCta	105
PAK4	NM_001014834	GGGUGAAGCUGUCAGACUUtt	AAGUCUGACAGCUUCACCCtg	36
PAK4	NM_001014834	ACACCAGGAUGAACGAGGAtt	UCCUCGUUCAUCCUGGUGUgg	110
PAK4	NM_001014834	ACUAAGAGGUGAACAUGUAtt	UACAUGUUCACCUCUUAGUgt	126
PAK6	NM_020168	AUCCAGAAGUUGUCAGUCAtt	UGACUGACAACUUCUGGAUgt	169
PAK6	NM_020168	GGCUAUUCCGAAGCAUGUUtt	AACAUGCUUCGGAAUAGCCtg	136
PAK6	NM_020168	GGACAGCUACGUGAAGAUUtt	AAUCUUCACGUAGCUGUCCag	102
PAK7	NM_020341	GGUGGAUUACGAUCGAGCAtt	UGCUCGAUCGUAAUCCACCtt	175
PAK7	NM_020341	GUAUUCCAGCGAAUCCGAUtt	AUCGGAUUCGCUGGAAUACtg	273
PAK7	NM_020341	GGAUUACCACCAUGACAAUtt	AUUGUCAUGGUGGUAAUCCcg	76
PANK1	NM_148977	GCAACAUGAUGAGUAAAGAtt	UCUUUACUCAUCAUGUUGCca	169
PANK1	NM_148977	CGCGUUGAAUGAGAACAUAtt	UAUGUUCUCAUUCAACGCGca	371
PANK1	NM_148977	GUACUGCCUUGAUAACCCAtt	UGGGUUAUCAAGGCAGUACgg	76
PANK2	NM_024960	GAGCGACUUUGAUCACCAUtt	AUGGUGAUCAAAGUCGCUCtg	175

PANK2	NM_024960	GCAUAUGCUUUGGAUUAUUtt	AAUAAUCCAAAGCAUAUGCca	292
PANK2	NM_024960	GGACGGUCACAGUGCUAUUtt	AAUAGCACUGUGACCGUCCat	151
PANK3	NM_024594	GCGAGAAUCUGUUAGUAAAtt	UUUACUAACAGAUUCUCGCtt	89
PANK3	NM_024594	CUCUCGUACUUUGAACCUAtt	UAGGUUCAAAGUACGAGAGct	65
PANK3	NM_024594	GAUUUUCGCACAAUUGGAAtt	UUCCAAUUGUGCGAAAAUCtt	66
PANK4	NM_018216	CCUUAAUUUUCGCAGAUAAtt	UUAUCUGCGAAAAUUAAGGca	225
PANK4	NM_018216	GGCCUACUAUUCAACGGUAtt	UACCGUUGAAUAGUAGGCCag	166
PANK4	NM_018216	GUGCCUUAAUUUUCGCAGAtt	UCUGCGAAAAUUAAGGCACat	182
PAPSS1	NM_005443	GGAUCGAUUCUGAAUAUGAtt	UCAUAUUCAGAAUCGAUCCca	151
PAPSS1	NM_005443	GCAUCGCAGAAGUUGCUAAtt	UUAGCAACUUCUGCGAUGCgt	209
PAPSS1	NM_005443	GGCUUAGUGUGCAUCACAAtt	UUGUGAUGCACACUAAGCCag	221
PAPSS2	NM_001015880	GGGUAGCUAUCUUACGAGAtt	UCUCGUAAGAUAGCUACCCtc	154
PAPSS2	NM_001015880	CAUUCGCAAAGGAUCGUGAtt	UCACGAUCCUUUGCGAAUGga	209
PAPSS2	NM_001015880	CCACCAAUGUAGUCUAUCAtt	UGAUAGACUACAUUGGUGGat	477
PASK	NM_015148	GAAUCUUGCUGACUAUACAtt	UGUAUAGUCAGCAAGAUUCac	92
PASK	NM_015148	CCUGGUUGCUAACGACAAAtt	UUUGUCGUUAGCAACCAGGat	110
PASK	NM_015148	GCCUAGACCUCUUCGCUUUtt	AAAGCGAAGAGGUCUAGGCcg	287
PBK	NM_018492	GACUAAUGGAUGAAGCUAAtt	UUAGCUUCAUCCAUUAGUCtc	125
PBK	NM_018492	CCCUGAGGCUUGUUACAUUtt	AAUGUAACAAGCCUCAGGGtc	348
PBK	NM_018492	GCACUAAUGAAGACCCUAAtt	UUAGGGUCUUCAUUAGUGCat	156
PCM1	NM_006197	CCGUGAUUCACUUAGAUCAtt	UGAUCUAAGUGAAUCACGGtg	135
PCM1	NM_006197	CAAAGACUCCACAUACGUUtt	AACGUAUGUGGAGUCUUUGtt	130
PCM1	NM_006197	CAGUAUGCCUGAUCCAGUAtt	UACUGGAUCAGGCAUACUGct	136
PCTK1	NM_033018	ACAUCGUUACGCUACAUGAtt	UCAUGUAGCGUAACGAUGUtg	126
PCTK1	NM_033018	CUUCAUCUUCCGUAUCUUAtt	UAAGAUACGGAAGAUGAAGtg	296
PCTK1	NM_033018	CCAUAUUUGCACUAAAGGAtt	UCCUUUAGUGCAAAUAUGGaa	207
PCTK2	NM_002595	CCAUUUGACCAACCAAUGAtt	UCAUUGGUUGGUCAAAUGGtg	97
PCTK2	NM_002595	GGGUACAUAUGCAACAGUAtt	UACUGUUGCAUAUGUACCCtc	148
PCTK2	NM_002595	GGAUCUCAAUGGAGGAUUUtt	AAAUCCUCCAUUGAGAUCCgt	203
PCTK3	NM_002596	GCACUGAGACCAUUGAAGAtt	UCUUCAAUGGUCUCAGUGCgg	122
PCTK3	NM_002596	GCAGCAAACUGACGGAGAAtt	UUCUCCGUCAGUUUGCUGCgc	141

PCTK3	NM_002596	CCCUCACCCUGGUGUUUGAtt	UCAAACACCAGGGUGAGGGac	171
PDGFRA	NM_006206	CUCUAGGAAUGACGGAUUAtt	UAAUCCGUCAUUCCUAGAGgt	87
PDGFRA	NM_006206	GGCCUUACUUUAUUGGAUUtt	AAUCCAAUAAAGUAAGGCCtt	104
PDGFRA	NM_006206	CAUCAGAGCUGGAUCUAGAtt	UCUAGAUCCAGCUCUGAUGtt	53
PDGFRB	NM_002609	GAGCAACUUUGAUCAACGAtt	UCGUUGAUCAAAGUUGCUCgg	128
PDGFRB	NM_002609	GGCUAGACACGGGAGAAUAtt	UAUUCUCCCGUGUCUAGCCca	111
PDGFRB	NM_002609	GGAACGUGCUCAUCUGUGAtt	UCACAGAUGAGCACGUUCCta	107
PDGFRL	NM_006207	GAGCUUCGAUGUAAAGGGAtt	UCCCUUUACAUCGAAGCUCta	256
PDGFRL	NM_006207	GAACGGACAUUGUUUAUGAtt	UCAUAAACAAUGUCCGUUCca	228
PDGFRL	NM_006207	CGAUGUAAAGGGAGUAGAAtt	UUCUACUCCCUUUACAUCGaa	127
PDIK1L	NM_152835	GAAUGAAACAACUGAUUAAtt	UUAAUCAGUUGUUUCAUUCgc	137
PDIK1L	NM_152835	GGGCGAAUGAAACAACUGAtt	UCAGUUGUUUCAUUCGCCCat	128
PDIK1L	NM_152835	GAGUAUCUGUUGUCCAGGAtt	UCCUGGACAACAGAUACUCat	104
PDK1	NM_002610	CAGAUACUGUGAUACGGAUtt	AUCCGUAUCACAGUAUCUGta	152
PDK1	NM_002610	GAGUCGCAUUUCAAUUAGAtt	UCUAAUUGAAAUGCGACUCat	192
PDK1	NM_002610	CAAACUGCAAUGUACUUGAtt	UCAAGUACAUUGCAGUUUGga	319
PDK2	NM_002611	AGAUCAACCUGCUUCCCGAtt	UCGGGAAGCAGGUUGAUCUct	219
PDK2	NM_002611	CAACCCAGCCCAUCCCAAAtt	UUUGGGAUGGGCUGGGUUGgt	186
PDK2	NM_002611	CAGCAAUGCCUGUGAGAAAtt	UUUCUCACAGGCAUUGCUGga	162
PDK3	NM_005391	GACUUAUCCAUUAAGAUCAtt	UGAUCUUAAUGGAUAAGUCtt	118
PDK3	NM_005391	CCCUCGUUACUUUGGGUAAtt	UUACCCAAAGUAACGAGGGtt	150
PDK3	NM_005391	CAUAUAUGUUUCUACGAAAtt	UUUCGUAGAAACAUAUAUGaa	163
PDK4	NM_002612	GGAUGCUCUGUGAUCAGUAtt	UACUGAUCACAGAGCAUCCtt	118
PDK4	NM_002612	GGGCAACAGUUGAACACCAtt	UGGUGUUCAACUGUUGCCCgc	150
PDK4	NM_002612	CCGCCUCUUUAGUUAUACAtt	UGUAUAACUAAAGAGGCGGtc	242
PDPK1	NM_002613	CGGUUAGGCUGUGAGGAAAtt	UUUCCUCACAGCCUAACCGct	97
PDPK1	NM_002613	GGAACAGCGCAGUACGUUUtt	AAACGUACUGCGCUGUUCCca	134
PDPK1	NM_002613	GGGUUUAUUUGCAAGACGAtt	UCGUCUUGCAAAUAAACCCtt	139
PDXK	NM_003681	CCGUGGUGAUGGAACGCAUtt	AUGCGUUCCAUCACCACGGag	46
PDXK	NM_003681	GGAUGGACAUUCGCAAAGUtt	ACUUUGCGAAUGUCCAUCCgg	143
PDXK	NM_003681	GGUUAUACGAGGGACAAGUtt	ACUUGUCCCUCGUAUAACCtg	294

PFKL	NM_002626	GCACAAUACCGCAUCAGUAtt	UACUGAUGCGGUAUUGUGCca	156
PFKL	NM_002626	CGUCACUGAGCUCAAGAAAtt	UUUCUUGAGCUCAGUGACGgg	165
PFKL	NM_002626	CAGUAUACGUGGUGCACGAtt	UCGUGCACCACGUAUACUGtg	188
PFKM	NM_000289	GGACUUUCGGGAACGAGAAtt	UUCUCGUUCCCGAAAGUCCtt	164
PFKM	NM_000289	GCUCUAAACUUGGGACUAAtt	UUAGUCCCAAGUUUAGAGCca	137
PFKM	NM_000289	GGAAGAAUGUGCUUGGUCAtt	UGACCAAGCACAUUCUUCCtg	114
PFKP	NM_002627	GAGCAAUUGAUACCCAAAAtt	UUUUGGGUAUCAAUUGCUCct	107
PFKP	NM_002627	CCGCAUACAUUUUCGAAGAtt	UCUUCGAAAAUGUAUGCGGca	190
PFKP	NM_002627	GGAUCACUGCAAAACUCAAtt	UUGAGUUUUGCAGUGAUCCac	139
PFTK1	NM_012395	CCAACGAAGUGGUUACCUUtt	AAGGUAACCACUUCGUUGGag	114
PFTK1	NM_012395	GGAAUGGACUCAGUGAUCAtt	UGAUCACUGAGUCCAUUCCct	92
PFTK1	NM_012395	GGAUCAACUUGAACGAAUAtt	UAUUCGUUCAAGUUGAUCCtg	162
PGK1	NM_000291	GGAACAAGGUUAAAGCCGAtt	UCGGCUUUAACCUUGUUCCca	221
PGK1	NM_000291	GCCUGACAAGUACUCCUUAtt	UAAGGAGUACUUGUCAGGCat	82
PGK1	NM_000291	GAACGGAUCAGAUGUCUAUtt	AUAGACAUCUGAUCCGUUCct	316
PGK2	NM_138733	GGAACUAGAUUACUUUGCUtt	AGCAAAGUAAUCUAGUUCCtt	244
PGK2	NM_138733	GCAUCCGGAUUCUUGAUGAtt	UCAUCAAGAAUCCGGAUGCtt	128
PGK2	NM_138733	GCCUUCCGAGCAUCACUUUtt	AAAGUGAUGCUCGGAAGGCtt	69
PHKG1	NM_006213	GGAUGAUCAUGAGCGGCAAtt	UUGCCGCUCAUGAUCAUCCtc	152
PHKG1	NM_006213	GGACUUCUAUGAGAAUUAUtt	AUAAUUCUCAUAGAAGUCCtg	160
PHKG1	NM_006213	GCACAGGACUUCUAUGAGAtt	UCUCAUAGAAGUCCUGUGCag	227
PHKG2	NM_000294	AAGGAAACCAGGUCCAUCAtt	UGAUGGACCUGGUUUCCUUtt	209
PHKG2	NM_000294	AGAUCCUGAUGUUACGCAUtt	AUGCGUAACAUCAGGAUCUgc	147
PHKG2	NM_000294	GCACUGUCAAAGACCUGAUtt	AUCAGGUCUUUGACAGUGCtg	97
PI4K2B	NM_018323	GGUUAGUCAGAUACGAAAAtt	UUUUCGUAUCUGACUAACCaa	155
PI4K2B	NM_018323	CCUAUCUUGUGGACAACAAtt	UUGUUGUCCACAAGAUAGGca	152
PI4K2B	NM_018323	GAUUGACCGUGCAAAAUCAtt	UGAUUUUGCACGGUCAAUCgc	127
PI4KII	NM_018425	CCAAAGAUAUCGGACCCUAtt	UAGGGUCCGAUAUCUUUGGaa	156
PI4KII	NM_018425	GAAGCUACUUCGUCAAGGAtt	UCCUUGACGAAGUAGCUUCcg	97
PI4KII	NM_018425	GCAUCGGGCUACCACCAAAtt	UUUGGUGGUAGCCCGAUGCgg	154
PICK1	NM_012407	GGUGAGCACCGGCAACUAUtt	AUAGUUGCCGGUGCUCACCcg	95

PICK1	NM_012407	GAUCUGAACACGUACCUCAtt	UGAGGUACGUGUUCAGAUCcg	171
PICK1	NM_012407	GCAGGUCAAUCAAAGGGAAtt	UUCCCUUUGAUUGACCUGCca	268
PIK3AP1	NM_152309	GCAUUUUGCUGCGAAGUAUtt	AUACUUCGCAGCAAAAUGCaa	122
PIK3AP1	NM_152309	CAGAGACCCUUGAUAAACUtt	AGUUUAUCAAGGGUCUCUGtg	54
PIK3AP1	NM_152309	GGAAAAUCUUAAACGGCUAtt	UAGCCGUUUAAGAUUUUCCtg	131
PIK3C2A	NM_002645	GCCUACAACUUGAUAAGAAtt	UUCUUAUCAAGUUGUAGGCct	204
PIK3C2A	NM_002645	GGAUCUUUUUAAACCUAUUtt	AAUAGGUUUAAAAAGAUCCtt	90
PIK3C2A	NM_002645	GGCUUUGAGUUGUCAAGCAtt	UGCUUGACAACUCAAAGCCtc	206
PIK3C2G	NM_004570	CCAUCUACCAGCUAAUCAAtt	UUGAUUAGCUGGUAGAUGGat	144
PIK3C2G	NM_004570	GUAGCAUUCCUCCAACAAAtt	UUUGUUGGAGGAAUGCUACtt	149
PIK3C2G	NM_004570	GCUACUGGGUGGGAGUAUAtt	UAUACUCCCACCCAGUAGCtg	419
PIK3C3	NM_002647	GAGAUGUACUUGAACGUAAtt	UUACGUUCAAGUACAUCUCat	143
PIK3C3	NM_002647	GCAUGGAGAUGAUUUACGUtt	ACGUAAAUCAUCUCCAUGCtt	121
PIK3C3	NM_002647	GCUUAGACCUGUCGGAUGAtt	UCAUCCGACAGGUCUAAGCgg	75
PIK3CA	NM_006218	GACUAGCUAGAGACAAUGAtt	UCAUUGUCUCUAGCUAGUCtg	118
PIK3CA	NM_006218	GUAAUUACCCAGAUCCUAUtt	AUAGGAUCUGGGUAAUUACag	107
PIK3CA	NM_006218	GGUGCACUGCAGUUCAACAtt	UGUUGAACUGCAGUGCACCtt	101
PIK3CB	NM_006219	GUAAAUGAAUUUCGAAGAAtt	UUCUUCGAAAUUCAUUUACtt	106
PIK3CB	NM_006219	CUCCAAAUGUUGCGCUUGAtt	UCAAGCGCAACAUUUGGAGtg	272
PIK3CB	NM_006219	GGGAAAGCUGGACUACUAAtt	UUAGUAGUCCAGCUUUCCCtg	392
PIK3CD	NM_005026	GUGAGAAAUUUGAACGGUUtt	AACCGUUCAAAUUUCUCACta	80
PIK3CD	NM_005026	GACUAAUAAUAGUGAGAAAtt	UUUCUCACUAUUAUUAGUCtt	115
PIK3CD	NM_005026	GCACUUCCGAGUGAAGUUUtt	AAACUUCACUCGGAAGUGCtt	116
PIK3CG	NM_002649	GCUUUAGAGUUCCAUAUGAtt	UCAUAUGGAACUCUAAAGCtt	110
PIK3CG	NM_002649	GCUGCACGACUUUACCCAAtt	UUGGGUAAAGUCGUGCAGCat	117
PIK3CG	NM_002649	GUAAUCGAGAUGUUACAAAtt	UUUGUAACAUCUCGAUUACtt	135
PIK3R3	NM_003629	GGAUAUCAAUCGAGUACAAtt	UUGUACUCGAUUGAUAUCCtc	112
PIK3R3	NM_003629	GAUCCGAGAUCAACACCUUtt	AAGGUGUUGAUCUCGGAUCtt	159
PIK3R3	NM_003629	GGAUGCUGCUGAGAACUAUtt	AUAGUUCUCAGCAGCAUCCtc	113
PIK3R4	NM_014602	CCACCUAUCAGAUUCGAAUtt	AUUCGAAUCUGAUAGGUGGat	260
PIK3R4	NM_014602	GGAUCGUAUUACUCCAUAUtt	AUAUGGAGUAAUACGAUCCaa	302

PIK3R4	NM_014602	CCACUACCAGAUCUAUUCUtt	AGAAUAGAUCUGGUAGUGGtg	199
PIK4CA	NM_002650	GACUAACAUUUAUCUAGAUtt	AUCUAGAUAAAUGUUAGUCtt	222
PIK4CA	NM_002650	CAGUUCAUCUGGAACAUGAtt	UCAUGUUCCAGAUGAACUGgt	293
PIK4CA	NM_002650	CUACCUGUCUUAUUUCAUAtt	UAUGAAAUAAGACAGGUAGgg	144
PIK4CB	NM_002651	CGAGAGUAUUGAUAAUUCAtt	UGAAUUAUCAAUACUCUCGgt	138
PIK4CB	NM_002651	GCACCGAGAGUAUUGAUAAtt	UUAUCAAUACUCUCGGUGCtg	138
PIK4CB	NM_002651	GCACUGUGCCCAACUAUGAtt	UCAUAGUUGGGCACAGUGCtg	41
PIM1	NM_002648	ACAUCCUUAUCGACCUCAAtt	UUGAGGUCGAUAAGGAUGUtt	153
PIM1	NM_002648	CCGUCUACACGGACUUCGAtt	UCGAAGUCCGUGUAGACGGtg	140
PIM1	NM_002648	CCUUCGAAGAAAUCCAGAAtt	UUCUGGAUUUCUUCGAAGGtt	177
PIM2	NM_006875	CUGCUUGACUGGUUUGAGAtt	UCUCAAACCAGUCAAGCAGgc	138
PIM2	NM_006875	ACCUUCUUCCCGACCCUCAtt	UGAGGGUCGGGAAGAAGGUtt	175
PIM2	NM_006875	AGAACAUCCUGAUAGACCUtt	AGGUCUAUCAGGAUGUUCUca	286
PIM3	XM_938171	AUUUAUUUGUUGAGGUUAUtt	AUAACCUCAACAAAUAAAUta	109
PIM3	XM_938171	GCCCUGGGUGGAUACUUGAtt	UCAAGUAUCCACCCAGGGCag	104
PIM3	XM_938171	GUCUGGAGAUCAUAUUUUUtt	AAAAAUAUGAUCUCCAGACat	177
PINK1	NM_032409	CCUCGUUAUGAAGAACUAUtt	AUAGUUCUUCAUAACGAGGaa	151
PINK1	NM_032409	ACAGAGACCUGAAAUCCGAtt	UCGGAUUUCAGGUCUCUGUgc	131
PINK1	NM_032409	GGAGCAGUCACUUACAGAAtt	UUCUGUAAGUGACUGCUCCat	207
PIP5K1A	NM_003557	CUAGACUUCUUACAAGACAtt	UGUCUUGUAAGAAGUCUAGgt	114
PIP5K1A	NM_003557	GCAACUCCUGCAUUACUUAtt	UAAGUAAUGCAGGAGUUGCca	128
PIP5K1A	NM_003557	GGACUUUGCUGCCUAAAUUtt	AAUUUAGGCAGCAAAGUCCga	55
PIP5K1B	NM_003558	CAGUGAACCUCUAAUAGAAtt	UUCUAUUAGAGGUUCACUGca	149
PIP5K1B	NM_003558	GGCUCAACGUAUAAGCGAAtt	UUCGCUUAUACGUUGAGCCtt	120
PIP5K1B	NM_003558	GAUCAUGGAUUAUAGCCUUtt	AAGGCUAUAAUCCAUGAUCtt	202
PIP5K1C	NM_012398	AGACCGUCAUGCACAAGGAtt	UCCUUGUGCAUGACGGUCUtg	49
PIP5K1C	NM_012398	CAGUCCUACAGGUUCAUCAtt	UGAUGAACCUGUAGGACUGca	181
PIP5K1C	NM_012398	GCGUCGUGGUCAUGAACAAtt	UUGUUCAUGACCACGACGCgg	122
PIP5K2A	NM_005028	CCGUGAACCCAGAACAGUAtt	UACUGUUCUGGGUUCACGGtg	111
PIP5K2A	NM_005028	CACACUUCCUACGACAAAAtt	UUUUGUCGUAGGAAGUGUGaa	102
PIP5K2A	NM_005028	CCGGCUUAAUGUUGAUGGAtt	UCCAUCAACAUUAAGCCGGta	267

PIP5K2B	NM_003559	GCUUUAAGUUUAAGGAGUAtt	UACUCCUUAAACUUAAAGCgg	295
PIP5K2B	NM_003559	GGAGAUGCACAACAUCUUAtt	UAAGAUGUUGUGCAUCUCCgc	124
PIP5K2B	NM_003559	GCCCAGCCGCUUUAAGUUUtt	AAACUUAAAGCGGCUGGGCag	183
PIP5K2C	NM_024779	GAAAGUAUAUAUUGGUGAAtt	UUCACCAAUAUAUACUUUCtg	171
PIP5K2C	NM_024779	GAACCUCCGUGAUCGAUUUtt	AAAUCGAUCACGGAGGUUCct	203
PIP5K2C	NM_024779	GGAGCAGUAUGCUAAGCGAtt	UCGCUUAGCAUACUGCUCCgg	211
PIP5KL1	NM_173492	CAAGAUCAUUCAGCAAUAAtt	UUAUUGCUGAAUGAUCUUGat	92
PIP5KL1	NM_173492	GGAUUACAGCCUCCUGAUAtt	UAUCAGGAGGCUGUAAUCCag	106
PIP5KL1	NM_173492	AGCUCAACGUGCUGGAUUAtt	UAAUCCAGCACGUUGAGCUcc	126
PKLR	NM_181871	GCUUCUGUCUCGGUACCGAtt	UCGGUACCGAGACAGAAGCtg	166
PKLR	NM_181871	GCUUUACCGUGAACCUCCAtt	UGGAGGUUCACGGUAAAGCaa	41
PKLR	NM_181871	GCAAAAUUGAGAACCACGAtt	UCGUGGUUCUCAAUUUUGCtg	237
PKM2	NM_182470	GAUUAAGUCUGGAAUGAAUtt	AUUCAUUCCAGACUUAAUCat	97
PKM2	NM_182470	CAUCAAGAUUAUCAGCAAAtt	UUUGCUGAUAAUCUUGAUGtt	125
PKM2	NM_182470	GGAAAGAACAUCAAGAUUAtt	UAAUCUUGAUGUUCUUUCCct	187
PKMYT1	NM_004203	CAGCGGAUGUGUUCAGUCUtt	AGACUGAACACAUCCGCUGct	83
PKMYT1	NM_004203	GGACAGCAGCGGAUGUGUUtt	AACACAUCCGCUGCUGUCCca	154
PKMYT1	NM_004203	GCGGUAAAGCGUUCCAUGUtt	ACAUGGAACGCUUUACCGCat	288
PKN1	NM_213560	GGACAGUAAGACCAAGAUUtt	AAUCUUGGUCUUACUGUCCtg	149
PKN1	NM_213560	GCACUGUGCUUAAGCUGGAtt	UCCAGCUUAAGCACAGUGCtg	182
PKN1	NM_213560	ACAGCGACGUGUUCUCUGAtt	UCAGAGAACACGUCGCUGUgg	260
PKN2	NM_006256	GACGAGAAGAUGUUAGUAAtt	UUACUAACAUCUUCUCGUCct	146
PKN2	NM_006256	GCACCCAUUUUUCCGGCUAtt	UAGCCGGAAAAAUGGGUGCtt	176
PKN2	NM_006256	GGAUCUUCAAAGGAUCGGAtt	UCCGAUCCUUUGAAGAUCCat	152
PKN3	NM_013355	GGCUUGAGUUCAUUCAGAAtt	UUCUGAAUGAACUCAAGCCct	96
PKN3	NM_013355	GGAACGCAUCUUCUCUAAAtt	UUUAGAGAAGAUGCGUUCCtg	229
PKN3	NM_013355	GGGCUUGAGUUCAUUCAGAtt	UCUGAAUGAACUCAAGCCCtt	248
PLK1	NM_005030	CCAUUAACGAGCUGCUUAAtt	UUAAGCAGCUCGUUAAUGGtt	70
PLK1	NM_005030	CAACCAAAGUCGAAUAUGAtt	UCAUAUUCGACUUUGGUUGcc	50
PLK1	NM_005030	GCAAUUACAUGAGCGAGCAtt	UGCUCGCUCAUGUAAUUGCgg	79
PLK2	NM_006622	GCUAGUAUGUUGUCCAAAAtt	UUUUGGACAACAUACUAGCaa	236

PLK2	NM_006622	GGUUGAUUACUCUAACAAAtt	UUUGUUAGAGUAAUCAACCca	350
PLK2	NM_006622	CUACUUCGAGGACAAAGAAtt	UUCUUUGUCCUCGAAGUAGtg	153
PLK3	NM_004073	GCAUCAAGCAGGUUCACUAtt	UAGUGAACCUGCUUGAUGCag	289
PLK3	NM_004073	GGCUUUGGGUAUCAACUGUtt	ACAGUUGAUACCCAAAGCCga	200
PLK3	NM_004073	GUUCGGCUUUGGGUAUCAAtt	UUGAUACCCAAAGCCGAACtt	140
PLK4	NM_014264	GGACCUUAUUCACCAGUUAtt	UAACUGGUGAAUAAGGUCCtt	77
PLK4	NM_014264	GGACUUGGUCUUACAACUAtt	UAGUUGUAAGACCAAGUCCtt	143
PLK4	NM_014264	GGAACGAUGUCACUCAGCAtt	UGCUGAGUGACAUCGUUCCat	178
PLXNA1	NM_032242	AGCGGGUGGUGAAACUCUAtt	UAGAGUUUCACCACCCGCUgg	108
PLXNA1	NM_032242	CCAAAGGAGUCAGCACUGUtt	ACAGUGCUGACUCCUUUGGtg	232
PLXNA1	NM_032242	CCCUGAGAAUGAGAAUGCAtt	UGCAUUCUCAUUCUCAGGGtt	237
PLXNA2	NM_025179	GGCACUAUGGUGACCAUUAtt	UAAUGGUCACCAUAGUGCCtc	126
PLXNA2	NM_025179	GGAUCGCCCAUCAUUCUGAtt	UCAGAAUGAUGGGCGAUCCtg	379
PLXNA2	NM_025179	GGGAAGAUAUUUGUCAGCAtt	UGCUGACAAAUAUCUUCCCtg	112
PLXNA3	NM_017514	CAGUGAACCGAGUCUUUAAtt	UUAAAGACUCGGUUCACUGcg	187
PLXNA3	NM_017514	GCAGUGAACCGAGUCUUUAtt	UAAAGACUCGGUUCACUGCgc	243
PLXNA3	NM_017514	CAUCAUCAAUGGAAGCACUtt	AGUGCUUCCAUUGAUGAUGct	134
PLXNA4B	NM_181775	UCGCAUAUGUCUACAAGAAtt	UUCUUGUAGACAUAUGCGAtg	113
PLXNA4B	NM_181775	GCAGUGCGCUCUUAACCAUtt	AUGGUUAAGAGCGCACUGCtg	79
PLXNA4B	NM_181775	GGCCUUUGUGGGCACCAAAtt	UUUGGUGCCCACAAAGGCCag	96
PLXNB1	NM_002673	CGCGGACAGUUCAAGUAUAtt	UAUACUUGAACUGUCCGCGtt	135
PLXNB1	NM_002673	CAACUGCAUUCACUCCCAAtt	UUGGGAGUGAAUGCAGUUGgt	82
PLXNB1	NM_002673	CACCUUUGAUGGGACCUUUtt	AAAGGUCCCAUCAAAGGUGag	87
PLXNB2	XM_371474	GAACGAGAGUAUGAGAAGAtt	UCUUCUCAUACUCUCGUUCgg	69
PLXNB2	XM_371474	CAACCACACUGUUGCUUUUtt	AAAAGCAACAGUGUGGUUGtt	120
PLXNB2	XM_371474	ACAUCUUCCUCACGUCCUAtt	UAGGACGUGAGGAAGAUGUtg	157
PLXNB3	NM_005393	GGAAGAGACUCAACACCUUtt	AAGGUGUUGAGUCUCUUCCag	107
PLXNB3	NM_005393	CAGGUACUAUGAUCAGAUUtt	AAUCUGAUCAUAGUACCUGtg	148
PLXNB3	NM_005393	CUCUUUAUGUGAUCCUGUAtt	UACAGGAUCACAUAAAGAGca	409
PLXNC1	NM_005761	CCACUAUAAAAGUCUUUAAtt	UUAAAGACUUUUAUAGUGGat	109
PLXNC1	NM_005761	GGGCAUCGAACAUCACAAUtt	AUUGUGAUGUUCGAUGCCCgg	199

PLXNC1	NM_005761	GGAGAAUUCGUGUUGCAAAtt	UUUGCAACACGAAUUCUCCtc	269
PLXND1	NM_015103	CGAGUACCGGGUCAAGAUAtt	UAUCUUGACCCGGUACUCGtg	104
PLXND1	NM_015103	GCCCAUCACAAUCCAGGUAtt	UACCUGGAUUGUGAUGGGCag	163
PLXND1	NM_015103	GCCUUUCAGUCAUCGCGCAtt	UGCGCGAUGACUGAAAGGCag	372
PMVK	NM_006556	CAGUGUGGAUACUAAUAAAtt	UUUAUUAGUAUCCACACUGgg	205
PMVK	NM_006556	CGAGAACCAUGGAGUUGAAtt	UUCAACUCCAUGGUUCUCGat	233
PMVK	NM_006556	CCAGGCUUCUUUUGCAGGAtt	UCCUGCAAAAGAAGCCUGGgt	307
PNCK	NM_001039582	GGAUGACAUCUCAGAAUCAtt	UGAUUCUGAGAUGUCAUCCca	87
PNCK	NM_001039582	GCAGUGCUCCGUAGGAUCAtt	UGAUCCUACGGAGCACUGCga	157
PNCK	NM_001039582	CGGAAGAACUUUGCUCGGAtt	UCCGAGCAAAGUUCUUCCGga	141
PNKP	NM_007254	GGAGGAUCUUGUACCCAGAtt	UCUGGGUACAAGAUCCUCCag	202
PNKP	NM_007254	GGAAGUCCACCUUUCUCAAtt	UUGAGAAAGGUGGACUUCCcg	116
PNKP	NM_007254	CGAAGAAGCGUAUGCGGAAtt	UUCCGCAUACGCUUCUUCGgc	377
PRKAA1	NM_006251	GGAUCCAUCAUAUAGUUCAtt	UGAACUAUAUGAUGGAUCCtc	421
PRKAA1	NM_006251	GAGUCUACAGUUAUACCAAtt	UUGGUAUAACUGUAGACUCat	298
PRKAA1	NM_006251	CGGGAUCAGUUAGCAACUAtt	UAGUUGCUAACUGAUCCCGat	151
PRKAA2	NM_006252	GAUUUCGGAUUAUCUAAUAtt	UAUUAGAUAAUCCGAAAUCgg	181
PRKAA2	NM_006252	GCAUAUGGUUGUUCAUCGAtt	UCGAUGAACAACCAUAUGCct	177
PRKAA2	NM_006252	GAUUCGCAGUUUAGAUGUUtt	AACAUCUAAACUGCGAAUCtt	170
PRKACA	NM_002730	CCUGCAAGCUGUCAACUUUtt	AAAGUUGACAGCUUGCAGGat	132
PRKACA	NM_002730	CAAGGACAACUCAAACUUAtt	UAAGUUUGAGUUGUCCUUGaa	66
PRKACA	NM_002730	AGAUCGUCCUGACCUUUGAtt	UCAAAGGUCAGGACGAUCUgg	71
PRKACB	NM_207578	GCUAUAUCCAGGUCACAGAtt	UCUGUGACCUGGAUAUAGCct	78
PRKACB	NM_207578	GGAGUGCUAAUCUAUGAAAtt	UUUCAUAGAUUAGCACUCCta	61
PRKACB	NM_207578	CGAGUACCUCCAUUCACUAtt	UAGUGAAUGGAGGUACUCGaa	187
PRKACG	NM_002732	GAAGCAGGUCGAGCACAUAtt	UAUGUGCUCGACCUGCUUCat	140
PRKACG	NM_002732	CGGUUUCCCUCCAAACUCAtt	UGAGUUUGGAGGGAAACCGca	196
PRKACG	NM_002732	AGCACAUACUGAACGAGAAtt	UUCUCGUUCAGUAUGUGCUcg	132
PRKCA	NM_002737	CAACGUACCCAUUCCGGAAtt	UUCCGGAAUGGGUACGUUGta	143
PRKCA	NM_002737	GCUCCACACUAAAUCCGCAtt	UGCGGAUUUAGUGUGGAGCgg	98
PRKCA	NM_002737	GGCUGUACUUCGUCAUGGAtt	UCCAUGACGAAGUACAGCCga	137

PRKCB1	NM_212535	GGUCUGUUCUUCUUACAGAtt	UCUGUAAGAAGAACAGACCga	174
PRKCB1	NM_212535	GGAUGAAACUGACCGAUUUtt	AAAUCGGUCAGUUUCAUCCgg	113
PRKCB1	NM_212535	GAAUCGGACAAAGACAGAAtt	UUCUGUCUUUGUCCGAUUCtt	115
PRKCD	NM_212539	GGAUUAAAGUGUGAAGACUtt	AGUCUUCACACUUUAAUCCct	76
PRKCD	NM_212539	GGGACACUAUAUUCCAGAAtt	UUCUGGAAUAUAGUGUCCCgg	132
PRKCD	NM_212539	GGAGUGACCGGAAACAUCAtt	UGAUGUUUCCGGUCACUCCca	116
PRKCDBP	NM_145040	CGAAUCCUACAUCCACCAAtt	UUGGUGGAUGUAGGAUUCGct	119
PRKCDBP	NM_145040	GCUUGUGCCUUGUCCCAAAtt	UUUGGGACAAGGCACAAGCac	102
PRKCDBP	NM_145040	GCUCCAAAUGGAGAGUGUAtt	UACACUCUCCAUUUGGAGCag	120
PRKCE	NM_005400	GAGUGUAUGUGAUCAUCGAtt	UCGAUGAUCACAUACACUCtt	156
PRKCE	NM_005400	GGAAAGCAGGGAUACCAGUtt	ACUGGUAUCCCUGCUUUCCta	206
PRKCE	NM_005400	ACCACGCAUUAAAACCAAAtt	UUUGGUUUUAAUGCGUGGUtt	114
PRKCG	NM_002739	CCCGUAACCUAAUUCCUAUtt	AUAGGAAUUAGGUUACGGGcc	124
PRKCG	NM_002739	GGAACCUGACGAAACAGAAtt	UUCUGUUUCGUCAGGUUCCga	85
PRKCG	NM_002739	ACAAGUUACUGAACCAGGAtt	UCCUGGUUCAGUAACUUGUac	76
PRKCH	NM_006255	GGAUGAGUUUAGAAACUUUtt	AAAGUUUCUAAACUCAUCCtg	94
PRKCH	NM_006255	GUCUCGUCGUUUUGAUGAAtt	UUCAUCAAAACGACGAGACtt	70
PRKCH	NM_006255	CAAAGUGGAUUCAAAGAUUtt	AAUCUUUGAAUCCACUUUGtt	90
PRKCI	NM_002740	GAGACCUAAUGUUUCAUAUtt	AUAUGAAACAUUAGGUCUCct	136
PRKCI	NM_002740	GGAUAUGAUGGAGCAAAAAtt	UUUUUGCUCCAUCAUAUCCca	107
PRKCI	NM_002740	GUAAUUCCAUAUAAUCCUUtt	AAGGAUUAUAUGGAAUUACtg	87
PRKCQ	NM_006257	GCGAGGCUGUUAACCCUUAtt	UAAGGGUUAACAGCCUCGCcc	73
PRKCQ	NM_006257	CGUUGGAUGAGGUGGAUAAtt	UUAUCCACCUCAUCCAACGga	92
PRKCQ	NM_006257	GCUGCUUAAGAGAUACUGAtt	UCAGUAUCUCUUAAGCAGCga	84
PRKCSH	NM_002743	GGAACAGACGAGUACAACAtt	UGUUGUACUCGUCUGUUCCat	246
PRKCSH	NM_002743	CCACCAACGAAUACGUCUAtt	UAGACGUAUUCGUUGGUGGtg	114
PRKCSH	NM_002743	UGACUAUUGCGACUGCAAAtt	UUUGCAGUCGCAAUAGUCAtc	161
PRKCZ	NM_001033582	CGUUCGACAUCAUCACCGAtt	UCGGUGAUGAUGUCGAACGgg	190
PRKCZ	NM_001033582	GGACUUUGACCUAAUCAGAtt	UCUGAUUAGGUCAAAGUCCtg	142
PRKCZ	NM_001033582	CGAGGAUAUUGACUGGGUAtt	UACCCAGUCAAUAUCCUCGtc	195
PRKD1	NM_002742	CGAAGUUUUUAAUUACUCAtt	UGAGUAAUUAAAAACUUCGtt	147

PRKD1	NM_002742	CCUCAUUGUUUCGAAAUCAtt	UGAUUUCGAAACAAUGAGGat	92
PRKD1	NM_002742	CAUCAUCUAUGUAAGCCUAtt	UAGGCUUACAUAGAUGAUGac	86
PRKD2	NM_001079880	CAGUGGGCGUGAUCAUGUAtt	UACAUGAUCACGCCCACUGac	146
PRKD2	NM_001079880	GAACAACACGACCAACAGAtt	UCUGUUGGUCGUGUUGUUCtg	171
PRKD2	NM_001079880	AGAUGAUCCUGUCCAGUGAtt	UCACUGGACAGGAUCAUCUcc	89
PRKD3	NM_005813	GAACGAGUCUUUGUAGUAAtt	UUACUACAAAGACUCGUUCtg	178
PRKD3	NM_005813	GCCCGACUCUCUAAUGGAAtt	UUCCAUUAGAGAGUCGGGCag	384
PRKD3	NM_005813	CCACGAGAUUUCACAAACAtt	UGUUUGUGAAAUCUCGUGGtg	171
PRKDC	NM_001081640	GCGUUGGAGUGCUACAACAtt	UGUUGUAGCACUCCAACGCgg	76
PRKDC	NM_001081640	GCGCUUUUCUGGGUGAACUtt	AGUUCACCCAGAAAAGCGCgg	149
PRKDC	NM_001081640	CAAGCGACUUUAUAGCCUUtt	AAGGCUAUAAAGUCGCUUGaa	157
PRKG1	NM_006258	GGAUAGAGGUUCGUUUGAAtt	UUCAAACGAACCUCUAUCCct	140
PRKG1	NM_006258	CGGUAAAUGUCACUCGUGAtt	UCACGAGUGACAUUUACCGtt	50
PRKG1	NM_006258	GAUCCUAUGAAAACCUAUAtt	UAUAGGUUUUCAUAGGAUCtg	77
PRKG2	NM_006259	GCUAUGAAGUGUAUAAGGAtt	UCCUUAUACACUUCAUAGCaa	171
PRKG2	NM_006259	GCCUGGUUAUAGAUCGAGAtt	UCUCGAUCUAUAACCAGGCat	184
PRKG2	NM_006259	GAGAUUACAUCAUUAGAGAtt	UCUCUAAUGAUGUAAUCUCct	248
PRKX	NM_005044	GGCGAUUAGGAAACAUGAAtt	UUCAUGUUUCCUAAUCGCCtt	82
PRKX	NM_005044	GAACAAGGCGAUUAGGAAAtt	UUUCCUAAUCGCCUUGUUCtg	162
PRKX	NM_005044	GCUCGUGGUUGACAGAACAtt	UGUUCUGUCAACCACGAGCag	57
PRKY	NM_002760	GAACUGGACUUAUUUGAUUtt	AAUCAAAUAAGUCCAGUUCaa	215
PRKY	NM_002760	UCACAGAGCCUAUUAUUUUtt	AAAAUAAUAGGCUCUGUGAag	120
PRKY	NM_002760	CAAUGCAUUGUAUUCAGAAtt	UUCUGAAUACAAUGCAUUGct	94
PRPF4B	NM_003913	CAGUUGAUUUAAGAGGUAAtt	UUACCUCUUAAAUCAACUGgg	89
PRPF4B	NM_003913	GGAAAUAGGUCUAGUACUAtt	UAGUACUAGACCUAUUUCCat	184
PRPF4B	NM_003913	CCACGUGAUAAAUCAAGAAtt	UUCUUGAUUUAUCACGUGGtt	399
PRPS1	NM_002764	GCACUAUUGUCUCACCUGAtt	UCAGGUGAGACAAUAGUGCag	148
PRPS1	NM_002764	GAACUGCACUAUUGUCUCAtt	UGAGACAAUAGUGCAGUUCct	87
PRPS1	NM_002764	GACUUUGCCUUGAUUCACAtt	UGUGAAUCAAGGCAAAGUCca	84
PRPS1L1	NM_175886	GCAUUGCUCCAAAAUACGAtt	UCGUAUUUUGGAGCAAUGCtt	212
PRPS1L1	NM_175886	GACUUUGCUUUGAUUCAUAtt	UAUGAAUCAAAGCAAAGUCca	197

PRPS1L1	NM_175886	GGACCUACAUGCUUCUCAAtt	UUGAGAAGCAUGUAGGUCCat	115
PRPS2	NM_001039091	CCAAGAUUCAGGUCAUUGAtt	UCAAUGACCUGAAUCUUGGtg	164
PRPS2	NM_001039091	GGGUUACAUCAAUUGCAGAtt	UCUGCAAUUGAUGUAACCCtt	188
PRPS2	NM_001039091	AGGCUGUUGUCGUCACAAAtt	UUUGUGACGACAACAGCCUca	148
PRPSAP1	NM_002766	CAAUGUCCCAAGAUAAAGAtt	UCUUUAUCUUGGGACAUUGca	109
PRPSAP1	NM_002766	CGAUAACUGUAGUUGGAGAtt	UCUCCAACUACAGUUAUCGgt	134
PRPSAP1	NM_002766	GCAUCGCAAUCAUCGUGGAtt	UCCACGAUGAUUGCGAUGCgg	204
PRPSAP2	NM_002767	CAAGAGUACAAAUUCAAGAtt	UCUUGAAUUUGUACUCUUGtt	176
PRPSAP2	NM_002767	CAAUCACGGUUGUGGGUGAtt	UCACCCACAACCGUGAUUGgg	144
PRPSAP2	NM_002767	GUAUGGAGCUAUCAAAGAAtt	UUCUUUGAUAGCUCCAUACat	93
PSKH1	NM_006742	ACCGAGACCUCAAACCUGAtt	UCAGGUUUGAGGUCUCGGUgt	250
PSKH1	NM_006742	GCACUAAGAGUGACGUGUAtt	UACACGUCACUCUUAGUGCca	234
PSKH1	NM_006742	GGUGAUGACUGCUUGAUGAtt	UCAUCAAGCAGUCAUCACCct	132
PSKH2	NM_033126	GGACUUUAUAGACAAACUAtt	UAGUUUGUCUAUAAAGUCCtt	65
PSKH2	NM_033126	CCAGGUGAAGAGUCGAAAAtt	UUUUCGACUCUUCACCUGGat	87
PSKH2	NM_033126	GGAUCUGCACAGUCUUCUAtt	UAGAAGACUGUGCAGAUCCag	84
PTCD2	NM_024754	GAUACCUACUUACAGAUAAtt	UUAUCUGUAAGUAGGUAUCtt	166
PTCD2	NM_024754	CAGUCAAAUAUGUUGGAAAtt	UUUCCAACAUAUUUGACUGga	139
PTCD2	NM_024754	GACUCCACAUCAUUCAAUAtt	UAUUGAAUGAUGUGGAGUCtg	193
PTK2	NM_153831	CGAUAUAUGGAAGAUAGUAtt	UACUAUCUUCCAUAUAUCGgg	121
PTK2	NM_153831	GAUGUUGGUUUAAAGCGAUtt	AUCGCUUUAAACCAACAUCtt	113
PTK2	NM_153831	GGUCGAAUGAUAAGGUGUAtt	UACACCUUAUCAUUCGACCgg	210
PTK2B	NM_173176	GUGAAGAUGUGGUCCUGAAtt	UUCAGGACCACAUCUUCACgg	248
PTK2B	NM_173176	GAUGUGGUCCUGAAUCGUAtt	UACGAUUCAGGACCACAUCtt	102
PTK2B	NM_173176	GGAUCAUCAUGGAAUUGUAtt	UACAAUUCCAUGAUGAUCCag	126
PTK6	NM_005975	CAUCCAUGGUUAAGUCAUAtt	UAUGACUUAACCAUGGAUGaa	140
PTK6	NM_005975	GGUUUUGACUCACCUGAAAtt	UUUCAGGUGAGUCAAAACCaa	88
PTK6	NM_005975	CCGCGACUCUGAUGAGAAAtt	UUUCUCAUCAGAGUCGCGGag	187
PTK7	NM_002821	GCACAAGUGAUAAGAUGCAtt	UGCAUCUUAUCACUUGUGCtg	112
PTK7	NM_002821	GGAUGAAAAAUUGAAGUCAtt	UGACUUCAAUUUUUCAUCCtt	103
PTK7	NM_002821	GGAUUUCCAAGAGCAAGGAtt	UCCUUGCUCUUGGAAAUCCtc	74

PXK	NM_017771	CGGAAUAUAUUAUUCGAGUtt	ACUCGAAUAAUAUAUUCCGtg	230
PXK	NM_017771	GGAUCUGAUCUACAAGGCAtt	UGCCUUGUAGAUCAGAUCCtt	141
PXK	NM_017771	GACAUAGGUUGGAGAAUAAtt	UUAUUCUCCAACCUAUGUCtt	163
RAF1	NM_002880	GGAACUGUUUAUAAGGGUAtt	UACCCUUAUAAACAGUUCCaa	181
RAF1	NM_002880	GGAUUUCGAUGUCAGACUUtt	AAGUCUGACAUCGAAAUCCat	48
RAF1	NM_002880	CGUGUUUUCUUGCCGAACAtt	UGUUCGGCAAGAAAACACGga	342
RAGE	NM_014226	GGAAUACCUCUACUAACAAtt	UUGUUAGUAGAGGUAUUCCtg	471
RAGE	NM_014226	CACUAAUAUGUGAACUUAUtt	AUAAGUUCACAUAUUAGUGca	104
RAGE	NM_014226	CCUGGAUCAUAUUCACAGAtt	UCUGUGAAUAUGAUCCAGGga	167
RBKS	NM_022128	GAAUUUACAUAUCAGACUAtt	UAGUCUGAUAUGUAAAUUCtg	123
RBKS	NM_022128	GGUGGUAAUCAUUACCUUAtt	UAAGGUAAUGAUUACCACCtg	297
RBKS	NM_022128	GAUUGCUAUUAGUCCCAAAtt	UUUGGGACUAAUAGCAAUCaa	291
RET	NM_020630	GCUUGUCCCGAGAUGUUUAtt	UAAACAUCUCGGGACAAGCcg	202
RET	NM_020630	GGAUUGAAAACAAACUCUAtt	UAGAGUUUGUUUUCAAUCCat	101
RET	NM_020630	CCACUGCUACCACAAGUUUtt	AAACUUGUGGUAGCAGUGGat	189
RFK	NM_018339	GAGUUACCAGAACAUUUGAtt	UCAAAUGUUCUGGUAACUCta	160
RFK	NM_018339	CCAUAUUACAAGAAUACGAtt	UCGUAUUCUUGUAAUAUGGgt	212
RFK	NM_018339	GGAUGGAACCCAUAUUACAtt	UGUAAUAUGGGUUCCAUCCta	310
RIOK1	NM_031480	GCGAGCAUAUAUUCCUAGAtt	UCUAGGAAUAUAUGCUCGCtt	182
RIOK1	NM_031480	GGUGGAGGCGUGUAUAUCAtt	UGAUAUACACGCCUCCACCgt	281
RIOK1	NM_031480	GGUCAUUCAGUACAUGAGAtt	UCUCAUGUACUGAAUGACCtg	220
RIOK2	NM_018343	GAACCUCGUUUCGAAAUUUtt	AAAUUUCGAAACGAGGUUCtt	263
RIOK2	NM_018343	GGAAGAACCUCGUUUCGAAtt	UUCGAAACGAGGUUCUUCCta	176
RIOK2	NM_018343	GGUUGACAAAUGCAGGAUAtt	UAUCCUGCAUUUGUCAACCga	117
RIOK3	NM_003831	GGUUGAUCGAUGUCAGUCAtt	UGACUGACAUCGAUCAACCag	184
RIOK3	NM_003831	CAACAGUUGUACUACUGAAtt	UUCAGUAGUACAACUGUUGga	216
RIOK3	NM_003831	GAUCGCUUCAGUAAACUAAtt	UUAGUUUACUGAAGCGAUCtt	199
RIPK1	NM_003804	CCACUAGUCUGACGGAUAAtt	UUAUCCGUCAGACUAGUGGta	86
RIPK1	NM_003804	GUGUAGAAGAGGACGUGAAtt	UUCACGUCCUCUUCUACACtt	240
RIPK1	NM_003804	GCAAAGACCUUACGAGAAUtt	AUUCUCGUAAGGUCUUUGCtg	205
RIPK2	NM_003821	GCACGAUAUAUAUAGCUAUtt	AUAGCUAUAUAUAUCGUGCtt	457

RIPK2	NM_003821	GUAUGAUCUCUCUAAUAGAtt	UCUAUUAGAGAGAUCAUACgt	353
RIPK2	NM_003821	GCACGUAUGAUCUCUCUAAtt	UUAGAGAGAUCAUACGUGCtc	173
RIPK3	NM_006871	GGCAAGUCUGGAUAACGAAtt	UUCGUUAUCCAGACUUGCCat	138
RIPK3	NM_006871	GAACUGUUUGUUAACGUAAtt	UUACGUUAACAAACAGUUCtg	230
RIPK3	NM_006871	GGAGAACCAUAGAAAACCAtt	UGGUUUUCUAUGGUUCUCCta	290
RIPK4	NM_020639	UGGGAUCUCCGGUUCCGAAtt	UUCGGAACCGGAGAUCCCAtg	138
RIPK4	NM_020639	CCGAUGUCAUUGACCUGUUtt	AACAGGUCAAUGACAUCGGcg	86
RIPK4	NM_020639	GCACGAUGUAUACAGCUUUtt	AAAGCUGUAUACAUCGUGCtt	402
RIPK5	NM_015375	GGGUCGUCAGUUACAAGGAtt	UCCUUGUAACUGACGACCCtg	96
RIPK5	NM_015375	GUCUGGAAUAUACUCGAAAtt	UUUCGAGUAUAUUCCAGACgt	302
RIPK5	NM_015375	GCUUUGGAAUUUCACUAUAtt	UAUAGUGAAAUUCCAAAGCca	232
RNASEL	NM_021133	GGUAAGGUCAAAGCCCUAAtt	UUAGGGCUUUGACCUUACCat	152
RNASEL	NM_021133	CGACAUCAAAACACGAAAAtt	UUUUCGUGUUUUGAUGUCGga	257
RNASEL	NM_021133	GGAAGUCUCUUGUCUGCAAtt	UUGCAGACAAGAGACUUCCcg	153
ROCK1	NM_005406	GGUUAGAACAAGAGGUAAAtt	UUUACCUCUUGUUCUAACCgt	131
ROCK1	NM_005406	CGGUUAGAACAAGAGGUAAtt	UUACCUCUUGUUCUAACCGtt	102
ROCK1	NM_005406	GCUUGUAGGUGAUACACCUtt	AGGUGUAUCACCUACAAGCat	165
ROCK2	NM_004850	GGAGAUUACCUUACGGAAAtt	UUUCCGUAAGGUAAUCUCCtc	126
ROCK2	NM_004850	GAGAUUACCUUACGGAAAAtt	UUUUCCGUAAGGUAAUCUCct	192
ROCK2	NM_004850	GGAUCGAACCCAUGGAUCAtt	UGAUCCAUGGGUUCGAUCCct	349
ROR1	NM_001083592	GUACUGCGAUGAAACUUCAtt	UGAAGUUUCAUCGCAGUACgg	102
ROR1	NM_001083592	GGAUGAAAACUUUAAGUCUtt	AGACUUAAAGUUUUCAUCCaa	105
ROR1	NM_001083592	CCGUCUAUAUGGAGUCUUUtt	AAAGACUCCAUAUAGACGGtg	158
ROR2	NM_004560	GACAGAAUAUGGUUCACGAtt	UCGUGAACCAUAUUCUGUCtt	204
ROR2	NM_004560	GGAUUACAGAGGAACGGCAtt	UGCCGUUCCUCUGUAAUCCat	78
ROR2	NM_004560	UGGAGGUUCUGGAUCCGAAtt	UUCGGAUCCAGAACCUCCAct	101
ROS1	NM_002944	CUUCUUACUUAUUUGCGUAtt	UACGCAAAUAAGUAAGAAGgt	143
ROS1	NM_002944	GGUUGGAUGCUAUAUACCAtt	UGGUAUAUAGCAUCCAACCga	264
ROS1	NM_002944	CUACUACAGUGACACGAAAtt	UUUCGUGUCACUGUAGUAGag	154
RP11-145H9.1	NM_001012418	GCGAAUAAGUGCAAGCGAAtt	UUCGCUUGCACUUAUUCGCca	151
RP11-145H9.1	NM_001012418	GCAUCAUCGAUGAGAGCUAtt	UAGCUCUCAUCGAUGAUGCgg	124

RP11-145H9.1	NM_001012418	GAAUCGGGAUGCUAAGCAAtt	UUGCUUAGCAUCCCGAUUCac	152
RP2	NM_006915	GCGAGAAGGUUGAUCCAAAtt	UUUGGAUCAACCUUCUCGCgc	164
RP2	NM_006915	CCAUUGAUGACUGUACUAAtt	UUAGUACAGUCAUCAAUGGta	112
RP2	NM_006915	GCAGCGUGUUUUUCCGGAAtt	UUCCGGAAAAACACGCUGCct	176
RP6-213H19.1	NM_001042453	GAUAGAUCGUUUUAAGAGAtt	UCUCUUAAAACGAUCUAUCag	114
RP6-213H19.1	NM_001042453	GAAGAACUCGAGAAAAGUAtt	UACUUUUCUCGAGUUCUUCaa	115
RP6-213H19.1	NM_001042453	GCUUUACCACCGUACGAAAtt	UUUCGUACGGUGGUAAAGCtc	212
RPS6KA1	NM_001006665	CACUGAUUCUGAAGGCGAAtt	UUCGCCUUCAGAAUCAGUGtc	158
RPS6KA1	NM_001006665	ACGGCUACGUGGUAAAGGAtt	UCCUUUACCACGUAGCCGUca	184
RPS6KA1	NM_001006665	CAUUGACUGGAAUAAGCUAtt	UAGCUUAUUCCAGUCAAUGgt	315
RPS6KA2	NM_001006932	CGAUAUCUGACGCAGCUAAtt	UUAGCUGCGUCAGAUAUCGag	88
RPS6KA2	NM_001006932	CGAGCUCUCUUCAAACGGAtt	UCCGUUUGAAGAGAGCUCGca	90
RPS6KA2	NM_001006932	GAGUAUGCCGUGAAGAUCAtt	UGAUCUUCACGGCAUACUCgg	77
RPS6KA3	NM_004586	GAGAUUUGUUUACACGCUUtt	AAGCGUGUAAACAAAUCUCct	283
RPS6KA3	NM_004586	GCGCAGAGUCUUUUACGAAtt	UUCGUAAAAGACUCUGCGCtt	112
RPS6KA3	NM_004586	GGAUGCACCACAUCUAGUAtt	UACUAGAUGUGGUGCAUCCtg	103
RPS6KA4	NM_003942	CCUCCAUUCUCUUUGACCAtt	UGGUCAAAGAGAAUGGAGGgt	67
RPS6KA4	NM_003942	GCACUUCAGCGAGUCGGAAtt	UUCCGACUCGCUGAAGUGCcg	261
RPS6KA4	NM_003942	UCAUUUACCGAGACCUGAAtt	UUCAGGUCUCGGUAAAUGAtg	174
RPS6KA5	NM_182398	GCUUUAGCGAAAGACCUAAtt	UUAGGUCUUUCGCUAAAGCac	96
RPS6KA5	NM_182398	GCUGAGAUAUCUAGGAGAAtt	UUCUCCUAGAUAUCUCAGCtt	195
RPS6KA5	NM_182398	GGAAAUAACAGCUCUGAAAtt	UUUCAGAGCUGUUAUUUCCtt	335
RPS6KA6	NM_014496	GGAGAGUUACUUGACCGUAtt	UACGGUCAAGUAACUCUCCtc	78
RPS6KA6	NM_014496	GAGAGUUACUUGACCGUAUtt	AUACGGUCAAGUAACUCUCct	82
RPS6KA6	NM_014496	GGCUAGUGAUAUACUAUAUtt	AUAUAGUAUAUCACUAGCCtc	71
RPS6KB1	NM_003161	CAUGGAACAUUGUGAGAAAtt	UUUCUCACAAUGUUCCAUGcc	172
RPS6KB1	NM_003161	GGUUUUUCAAGUACGAAAAtt	UUUUCGUACUUGAAAAACCtt	89
RPS6KB1	NM_003161	GGACUAUGCAAAGAAUCUAtt	UAGAUUCUUUGCAUAGUCCaa	109
RPS6KB2	NM_003952	ACAUCAAACUGACCGACUUtt	AAGUCGGUCAGUUUGAUGUgg	208
RPS6KB2	NM_003952	CCCUUUUUCCGGCACAUGAtt	UCAUGUGCCGGAAAAAGGGat	73
RPS6KB2	NM_003952	GCAAGGAGUCUAUCCAUGAtt	UCAUGGAUAGACUCCUUGCag	267

RPS6KC1	NM_012424	GGAAUGGUGUUGAUACAAAtt	UUUGUAUCAACACCAUUCCct	74
RPS6KC1	NM_012424	GGCAAACUGUGGUCAUAUAtt	UAUAUGACCACAGUUUGCCac	141
RPS6KC1	NM_012424	CAAAUCCUAUAGUAUAACAtt	UGUUAUACUAUAGGAUUUGgg	265
RPS6KL1	NM_031464	ACUCAUCUCAGGACACUGAtt	UCAGUGUCCUGAGAUGAGUct	146
RPS6KL1	NM_031464	CGAUGUUAGUGAGGACUAUtt	AUAGUCCUCACUAACAUCGcg	94
RPS6KL1	NM_031464	GGUACUUUGUGAGCGAGGAtt	UCCUCGCUCACAAAGUACCtg	153
RYK	NM_001005861	GGUGAAGGAUAUAGCAAUAtt	UAUUGCUAUAUCCUUCACCtt	43
RYK	NM_001005861	GAAAGAUGGUUACCGAAUAtt	UAUUCGGUAACCAUCUUUCag	143
RYK	NM_001005861	GAAAUGACCUUAUUAGUCAtt	UGACUAAUAAGGUCAUUUCtc	122
SBK1	XM_937568	GGUCUUUGACGUGGUCUUUtt	AAAGACCACGUCAAAGACCtt	70
SBK1	XM_937568	CAAGGGCACAGGCACAAAAtt	UUUUGUGCCUGUGCCCUUGta	66
SBK1	XM_937568	GCUACGUCUUUGCCCAGGAtt	UCCUGGGCAAAGACGUAGCag	196
SCGB2A1	NM_002407	CCGACAUAUCUAUACCUGAtt	UCAGGUAUAGAUAUGUCGGaa	173
SCGB2A1	NM_002407	ACAUAUCUAUACCUGAAUAtt	UAUUCAGGUAUAGAUAUGUcg	84
SCGB2A1	NM_002407	AAGAGUUCAUAGACAGUGAtt	UCACUGUCUAUGAACUCUUga	187
SCYL1	NM_001048218	CGACCACAAAUCCUCCAAAtt	UUUGGAGGAUUUGUGGUCGga	193
SCYL1	NM_001048218	GAGUAUCAGCAGAAGAUCAtt	UGAUCUUCUGCUGAUACUCct	256
SCYL1	NM_001048218	GUCUCACUGUAGAUCCUGAtt	UCAGGAUCUACAGUGAGACcg	235
SCYL2	NM_017988	GCAUGAACCAGUUAUCACAtt	UGUGAUAACUGGUUCAUGCta	159
SCYL2	NM_017988	GGAUCAGUUGAGUCGUUUAtt	UAAACGACUCAACUGAUCCaa	258
SCYL2	NM_017988	GUCUGAACAUAAGACUAAAtt	UUUAGUCUUAUGUUCAGACtc	97
SCYL3	NM_181093	GAAUUCACCAUUCAAGUAAtt	UUACUUGAAUGGUGAAUUCct	191
SCYL3	NM_181093	GCACCUUACUAUCUCAUGAtt	UCAUGAGAUAGUAAGGUGCag	166
SCYL3	NM_181093	CAACAGACCUUGCACUCAAtt	UUGAGUGCAAGGUCUGUUGaa	50
SGK	NM_005627	CAAUCCUCAUGCUAAACCAtt	UGGUUUAGCAUGAGGAUUGga	160
SGK	NM_005627	CAGCUGAAAUGUACGACAAtt	UUGUCGUACAUUUCAGCUGtg	159
SGK	NM_005627	GGCUACCUGCAUUCACUGAtt	UCAGUGAAUGCAGGUAGCCca	239
SGK2	NM_170693	CUCCACCCUUCAACCCAAAtt	UUUGGGUUGAAGGGUGGAGtt	69
SGK2	NM_170693	AGAGCCUUAUGAUCGAGCAtt	UGCUCGAUCAUAAGGCUCUtt	159
SGK2	NM_170693	GCAGUGAAGGUACUACAGAtt	UCUGUAGUACCUUCACUGCat	313
SGK269	XM_370878	CAUUGUCUCCUGUUCGAUUtt	AAUCGAACAGGAGACAAUGtg	234

SGK269	XM_370878	GUCAAGAUCUGUAAGAGCAtt	UGCUCUUACAGAUCUUGACtg	75
SGK269	XM_370878	GCAUGAUAGGUGGGAUAAAtt	UUUAUCCCACCUAUCAUGCaa	103
SGK3	NM_001033578	GAGCAUUCCUUCAAAUGGAtt	UCCAUUUGAAGGAAUGCUCtg	141
SGK3	NM_001033578	GAGCAGGACUAAACGAAUUtt	AAUUCGUUUAGUCCUGCUCgt	324
SGK3	NM_001033578	CAUUAUUCCUUCCAAACAAtt	UUGUUUGGAAGGAAUAAUGca	80
SH3BP4	NM_014521	GCGGUAUGAUUGAUAAUCUtt	AGAUUAUCAAUCAUACCGCtg	141
SH3BP4	NM_014521	GAGACGAGCUUUAAUGACAtt	UGUCAUUAAAGCUCGUCUCtg	66
SH3BP4	NM_014521	GCGUAUGACUUCUUACUCAtt	UGAGUAAGAAGUCAUACGCag	195
SH3BP5	NM_001018009	GCAAAAGGCGAGUACAAGAtt	UCUUGUACUCGCCUUUUGCca	115
SH3BP5	NM_001018009	GGAGCGAGCUGGUGCAUAAtt	UUAUGCACCAGCUCGCUCCtg	176
SH3BP5	NM_001018009	UGACAGCUGUAGCAACUUUtt	AAAGUUGCUACAGCUGUCAtc	256
SH3BP5L	NM_030645	GAGUCGGCGAGGAAACUGAtt	UCAGUUUCCUCGCCGACUCct	102
SH3BP5L	NM_030645	GGUGCUAGUGGGUCCCUAAtt	UUAGGGACCCACUAGCACCtt	117
SH3BP5L	NM_030645	AGAACUGGAUCCUAGAAUAtt	UAUUCUAGGAUCCAGUUCUtc	162
SKAP1	NM_001075099	GCAAGGAGUAUAACAUGUAtt	UACAUGUUAUACUCCUUGCtc	87
SKAP1	NM_001075099	GGGUGGAUCAAAUAAGUUUtt	AAACUUAUUUGAUCCACCCag	184
SKAP1	NM_001075099	CACCCUUUUUGUCAGAUUAtt	UAAUCUGACAAAAAGGGUGca	296
SKP2	NM_032637	GAAUCUUAGCGGCUACAGAtt	UCUGUAGCCGCUAAGAUUCag	145
SKP2	NM_032637	GGCCUAAGCUAAAUCGAGAtt	UCUCGAUUUAGCUUAGGCCtg	149
SKP2	NM_032637	GACCUAUCGAACUCAGUUAtt	UAACUGAGUUCGAUAGGUCca	267
SLAMF6	NM_052931	GGAAAGCGACUGAACUUCAtt	UGAAGUUCAGUCGCUUUCCct	117
SLAMF6	NM_052931	GGAACAUACAAGUUACCAAtt	UUGGUAACUUGUAUGUUCCtc	94
SLAMF6	NM_052931	CCACGUGACUAAUCCGAAAtt	UUUCGGAUUAGUCACGUGGat	159
SLK	NM_014720	GGAAAUUGAGAAUCUAGAAtt	UUCUAGAUUCUCAAUUUCCtg	109
SLK	NM_014720	GAUCGAUAUCUUUACAAGAtt	UCUUGUAAAGAUAUCGAUCca	232
SLK	NM_014720	GCAGAAACAGACUAUCGAAtt	UUCGAUAGUCUGUUUCUGCtg	569
SMG1	NM_015092	GUGGGUACCAUAUCGCUUAtt	UAAGCGAUAUGGUACCCACta	218
SMG1	NM_015092	GCAACUGCUUUAAACCCGAtt	UCGGGUUUAAAGCAGUUGCca	290
SMG1	NM_015092	CCAUAUCGCUUAGUAGUGAtt	UCACUACUAAGCGAUAUGGta	585
SNF1LK	NM_173354	ACACGAUUAGAUUCAAGCAtt	UGCUUGAAUCUAAUCGUGUtt	105
SNF1LK	NM_173354	GCUUUACCAGGUUAUGGAAtt	UUCCAUAACCUGGUAAAGCtt	115

SNF1LK	NM_173354	CACUGAAUUUGCUAAAAAUtt	AUUUUUAGCAAAUUCAGUGac	112
SNF1LK2	NM_015191	CCAUAGCCCAAAUCAAGGAtt	UCCUUGAUUUGGGCUAUGGtt	156
SNF1LK2	NM_015191	GGAAGAUUGUGCACCGUGAtt	UCACGGUGCACAAUCUUCCga	272
SNF1LK2	NM_015191	GAAGGAUGUUGGUCCUAGAtt	UCUAGGACCAACAUCCUUCgg	300
SNRK	NM_017719	CCCGCAAGCUUAAAACCCAtt	UGGGUUUUAAGCUUGCGGGtg	117
SNRK	NM_017719	CAAUGAUCAUGGAUUGCAAtt	UUGCAAUCCAUGAUCAUUGtc	234
SNRK	NM_017719	CACUGACAAUGAUCAUGGAtt	UCCAUGAUCAUUGUCAGUGtt	204
SNX16	NM_022133	GGGUCCAUUUGAUAGCCUAtt	UAGGCUAUCAAAUGGACCCgg	228
SNX16	NM_022133	GAUAGACCAUCUACACCUAtt	UAGGUGUAGAUGGUCUAUCtt	109
SNX16	NM_022133	CACUUGAGGUUGAUCAAGAtt	UCUUGAUCAACCUCAAGUGca	233
SPHK1	NM_182965	AACUACUUCUGGAUGGUCAtt	UGACCAUCCAGAAGUAGUUtg	72
SPHK1	NM_182965	GGAAGAGUGGGUUCCAAGAtt	UCUUGGAACCCACUCUUCCta	238
SPHK1	NM_182965	UCACGCUGAUGCUCACUGAtt	UCAGUGAGCAUCAGCGUGAag	503
SPHK2	NM_020126	CCCUCACCCUUACAUCGCAtt	UGCGAUGUAAGGGUGAGGGca	133
SPHK2	NM_020126	CGGCCUACUUCUGCAUCUAtt	UAGAUGCAGAAGUAGGCCGct	100
SPHK2	NM_020126	CAAGGCAGCUCUACACUCAtt	UGAGUGUAGAGCUGCCUUGgg	116
SRC	NM_198291	CCAUUUACAUCGUCACGGAtt	UCCGUGACGAUGUAAAUGGgc	267
SRC	NM_198291	GCACAGGACAGACAGGCUAtt	UAGCCUGUCUGUCCUGUGCtg	132
SRC	NM_198291	GCCUCUCAGUGUCUGACUUtt	AAGUCAGACACUGAGAGGCag	201
SRMS	NM_080823	GCACGAGGUUUUCACCUAUtt	AUAGGUGAAAACCUCGUGCag	112
SRMS	NM_080823	GACCAACCCUGGUACUUUAtt	UAAAGUACCAGGGUUGGUCtg	128
SRMS	NM_080823	AGACCAACCCUGGUACUUUtt	AAAGUACCAGGGUUGGUCUga	139
SRPK1	NM_003137	GGUUAUCAUGGGAUAUUCAtt	UGAAUAUCCCAUGAUAACCat	148
SRPK1	NM_003137	GAUACCAUGUGAUCCGAAAtt	UUUCGGAUCACAUGGUAUCtc	116
SRPK1	NM_003137	GGAGAUCUAUUCAAUGGGAtt	UCCCAUUGAAUAGAUCUCCaa	230
SRPK2	NM_182691	GCAUAAACACUUCACGGAAtt	UUCCGUGAAGUGUUUAUGCac	130
SRPK2	NM_182691	GCAGAUAAAAUUAGAGUAAtt	UUACUCUAAUUUUAUCUGCat	88
SRPK2	NM_182691	GGGAGAUUAUUUGUUUGAAtt	UUCAAACAAAUAAUCUCCCgt	131
SRPK3	NM_014370	GAGAGACCAUUGUCCAGCUtt	AGCUGGACAAUGGUCUCUCtt	81
SRPK3	NM_014370	GCAGAUAAGAUCAAGAUCAtt	UGAUCUUGAUCUUAUCUGCat	117
SRPK3	NM_014370	GGCGCAAACGGAAACAGCAtt	UGCUGUUUCCGUUUGCGCCtc	116

STAP2	NM_017720	CCUUCACACUACUAUGAGAtt	UCUCAUAGUAGUGUGAAGGca	158
STAP2	NM_017720	AACUAUUUCGUGUCGCAUAtt	UAUGCGACACGAAAUAGUUga	114
STAP2	NM_017720	GGAGAUCAAGUUCAAGGUAtt	UACCUUGAACUUGAUCUCCtg	79
STK10	NM_005990	AGAUAGCCCUGGAUAAGAAtt	UUCUUAUCCAGGGCUAUCUtc	160
STK10	NM_005990	GAAGAGCAUCGGAACCAGAtt	UCUGGUUCCGAUGCUCUUCtt	86
STK10	NM_005990	GGACUACACCAGGUUCCAAtt	UUGGAACCUGGUGUAGUCCcg	386
STK11	NM_000455	GGCUCUUACGGCAAGGUGAtt	UCACCUUGCCGUAAGAGCCtt	134
STK11	NM_000455	ACAUCACCACGGGUCUGUAtt	UACAGACCCGUGGUGAUGUtg	103
STK11	NM_000455	AGGAGGUUACGGCACAAAAtt	UUUUGUGCCGUAACCUCCUca	173
STK11IP	NM_052902	CUGCGUUUCUUGAACCUAAtt	UUAGGUUCAAGAAACGCAGag	131
STK11IP	NM_052902	GGCAAGGUCUUGUCACUGAtt	UCAGUGACAAGACCUUGCCat	103
STK11IP	NM_052902	CCCUGUUCCUGUUAGAUGAtt	UCAUCUAACAGGAACAGGGtg	150
STK16	NM_001008910	AUAUAUUGCUUGGAGAUGAtt	UCAUCUCCAAGCAAUAUAUtg	121
STK16	NM_001008910	CCAACUCAGCAUCCCACAAtt	UUGUGGGAUGCUGAGUUGGtt	206
STK16	NM_001008910	GGUACGCUGUGGAAUGAGAtt	UCUCAUUCCACAGCGUACCtc	356
STK17A	NM_004760	GGUCAUUAAUUUACAUGAAtt	UUCAUGUAAAUUAAUGACCca	93
STK17A	NM_004760	GGAUACCGACAAAUCAGAAtt	UUCUGAUUUGUCGGUAUCCga	160
STK17A	NM_004760	GGACACUUUUAGUUAAGAAtt	UUCUUAACUAAAAGUGUCCtg	597
STK17B	NM_004226	CAGAAAUCCUGAACUAUGAtt	UCAUAGUUCAGGAUUUCUGga	60
STK17B	NM_004226	CGAUGACUCAUUACCCAAUtt	AUUGGGUAAUGAGUCAUCGaa	198
STK17B	NM_004226	CAUUUGUGGGAGAAGAUAAtt	UUAUCUUCUCCCACAAAUGgt	250
STK19	NM_032454	GCAUGGUCCGGAAGGCAAAtt	UUUGCCUUCCGGACCAUGCta	104
STK19	NM_032454	UCCUUAGCAUGGUCCGGAAtt	UUCCGGACCAUGCUAAGGAca	235
STK19	NM_032454	CCUGAUCCCGGAGACCUUUtt	AAAGGUCUCCGGGAUCAGGtg	227
STK24	NM_001032296	CCAGAUCGCUACUAUAUUAtt	UAAUAUAGUAGCGAUCUGGgt	196
STK24	NM_003576	GCUUUAGACCCACUGCUAAtt	UUAGCAGUGGGUCUAAAGCtc	168
STK24	NM_001032296	GCUCCGCACUAGAUCUAUUtt	AAUAGAUCUAGUGCGGAGCct	161
STK25	NM_006374	UCCUGUUCCUGAUUCCCAAtt	UUGGGAAUCAGGAACAGGAcg	148
STK25	NM_006374	ACAAGUUCAUCACACGCUAtt	UAGCGUGUGAUGAACUUGUgc	195
STK25	NM_006374	GGAUUAUCUGCACUCCGAAtt	UUCGGAGUGCAGAUAAUCCag	272
STK3	NM_006281	GAGAUACACUGCGAAAAGAtt	UCUUUUCGCAGUGUAUCUCtg	137

STK3	NM_006281	GACAUAAUUAGAUUACGAAtt	UUCGUAAUCUAAUUAUGUCtg	107
STK3	NM_006281	GCCCAUAUGUUGUAAAGUAtt	UACUUUACAACAUAUGGGCtg	185
STK32A	NM_145001	CGUUAUCACCUGCAACAGAtt	UCUGUUGCAGGUGAUAACGca	152
STK32A	NM_145001	UGAAGUACAUGAAUAAACAtt	UGUUUAUUCAUGUACUUCAtt	127
STK32A	NM_145001	AGGUCUGCAUUGUACAGAAtt	UUCUGUACAAUGCAGACCUtc	136
STK32B	NM_018401	AGAUGAUUCUAGAAUCCAAtt	UUGGAUUCUAGAAUCAUCUct	97
STK32B	NM_018401	ACAUAGCGACGGUAGUGAAtt	UUCACUACCGUCGCUAUGUtg	182
STK32B	NM_018401	CAUUACAGACUUCAACAUAtt	UAUGUUGAAGUCUGUAAUGtg	99
STK32C	NM_173575	GGAUGAGAGAGGACAUGCAtt	UGCAUGUCCUCUCUCAUCCag	170
STK32C	NM_173575	CGAGAAUGACUAUCUUCAAtt	UUGAAGAUAGUCAUUCUCGga	120
STK32C	NM_173575	GGCCGGUGUUUGACGACAAtt	UUGUCGUCAAACACCGGCCtc	144
STK33	NM_030906	GCUUAGCGGUGAAGAAGCAtt	UGCUUCUUCACCGCUAAGCca	160
STK33	NM_030906	GCUAAGGAACUACUAGAUAtt	UAUCUAGUAGUUCCUUAGCtg	169
STK33	NM_030906	GCAAUUUCCUGCAACCAGUtt	ACUGGUUGCAGGAAAUUGCtt	136
STK35	NM_080836	AGAUCGUCCCUGUUGGUGAtt	UCACCAACAGGGACGAUCUca	147
STK35	NM_080836	CAACAAAAGUUUCAUGCUAtt	UAGCAUGAAACUUUUGUUGgt	283
STK35	NM_080836	GGGCAAUGAUAGAAAGAAUtt	AUUCUUUCUAUCAUUGCCCag	131
STK36	NM_015690	GGAUCUUAGCCUCAGAAUUtt	AAUUCUGAGGCUAAGAUCCcg	257
STK36	NM_015690	CUACUGAAGUGACACUCUAtt	UAGAGUGUCACUUCAGUAGac	175
STK36	NM_015690	GCAUAUGCUUGACAGCUUUtt	AAAGCUGUCAAGCAUAUGCac	387
STK38	NM_007271	CCUUAUCGCUCAACAUGAAtt	UUCAUGUUGAGCGAUAAGGtt	66
STK38	NM_007271	GAGACUGACUACAAGAACAtt	UGUUCUUGUAGUCAGUCUCag	128
STK38	NM_007271	GCAAUGAAAAUACUCCGUAtt	UACGGAGUAUUUUCAUUGCat	97
STK38L	NM_015000	GGUUUGAAGGGUUGACUCAtt	UGAGUCAACCCUUCAAACCtt	30
STK38L	NM_015000	GGAGUGAUUAUGUAUGAAAtt	UUUCAUACAUAAUCACUCCca	144
STK38L	NM_015000	GGACUUAAUUCUCAGAUUUtt	AAAUCUGAGAAUUAAGUCCtt	258
STK39	NM_013233	GUGUAGCAAUAAAACGGAUtt	AUCCGUUUUAUUGCUACACgt	168
STK39	NM_013233	GGUUCAAUGUUGGAUAUCAtt	UGAUAUCCAACAUUGAACCtc	164
STK39	NM_013233	CCAACGUAGUGACCUAUUAtt	UAAUAGGUCACUACGUUGGga	114
STK4	NM_006282	GGAUGGAGACUACGAGUUUtt	AAACUCGUAGUCUCCAUCCtg	173
STK4	NM_006282	CAGUGAUAGGAACACCAUUtt	AAUGGUGUUCCUAUCACUGta	140

STK4	NM_006282	GGAAAUUGGAUACAACUGUtt	ACAGUUGUAUCCAAUUUCCtg	223
STK40	NM_032017	GCCGGAUGGUUAAGAAGAUtt	AUCUUCUUAACCAUCCGGCtg	101
STK40	NM_032017	GGCUGCCGAGUAUACCAUUtt	AAUGGUAUACUCGGCAGCCtt	301
STK40	NM_032017	GAAUCCAGCCGGAUGGUUAtt	UAACCAUCCGGCUGGAUUCtg	153
STYK1	NM_018423	GGCGGGAUGUGAUGACUAUtt	AUAGUCAUCACAUCCCGCCga	148
STYK1	NM_018423	CAAGAUUUCUUAGGGCGAAtt	UUCGCCCUAAGAAAUCUUGta	242
STYK1	NM_018423	CCAACUUUGUUGGUUACUAtt	UAGUAACCAACAAAGUUGGga	129
TAF1	NM_138923	GGAACUACAGCGAAUGCUAtt	UAGCAUUCGCUGUAGUUCCtt	217
TAF1	NM_138923	GGACCAGGAUUCUAUUACUtt	AGUAAUAGAAUCCUGGUCCtt	260
TAF1	NM_138923	CACUGUUCACUGUGACUAUtt	AUAGUCACAGUGAACAGUGgt	306
TAF1L	NM_153809	CACUGUUCAUUGUGACUAUtt	AUAGUCACAAUGAACAGUGgt	162
TAF1L	NM_153809	CCGUGAAAAUGUGCGUAAAtt	UUUACGCACAUUUUCACGGag	89
TAF1L	NM_153809	CAGUGUAUCUUCAUAAGAUtt	AUCUUAUGAAGAUACACUGga	143
TAOK1	NM_020791	GGAAUAUAAUAAGCGAAGAtt	UCUUCGCUUAUUAUAUUCCag	109
TAOK1	NM_020791	GAACGAGAACUAAGACGAAtt	UUCGUCUUAGUUCUCGUUCtc	89
TAOK1	NM_020791	CAGUGUAUUUUGCACGAGAtt	UCUCGUGCAAAAUACACUGct	164
TAOK2	NM_016151	CCACCGCUCUUUAACAUGAtt	UCAUGUUAAAGAGCGGUGGtt	89
TAOK2	NM_016151	GGGAGGACCUGAACAAGAAtt	UUCUUGUUCAGGUCCUCCCgc	209
TAOK2	NM_016151	CGGAAACCACCGCUCUUUAtt	UAAAGAGCGGUGGUUUCCGtt	206
TAOK3	NM_016281	GGAGUACAAUAAGAGGCGAtt	UCGCCUCUUAUUGUACUCCag	161
TAOK3	NM_016281	GCAUGGAUCUGAUCCGUUUtt	AAACGGAUCAGAUCCAUGCgt	306
TAOK3	NM_016281	GCAUGACUUUGUUCGACGAtt	UCGUCGAACAAAGUCAUGCct	144
TBK1	NM_013254	GAACGUAGAUUAGCUUAUAtt	UAUAAGCUAAUCUACGUUCtg	154
TBK1	NM_013254	GGAACCUCUGAAUACCAUAtt	UAUGGUAUUCAGAGGUUCCcg	237
TBK1	NM_013254	GGCACAACAUUUCCCUAAAtt	UUUAGGGAAAUGUUGUGCCag	127
tcag7.875	XM_372002	GAACUAUCUCUACCAAUAUtt	AUAUUGGUAGAGAUAGUUCaa	114
tcag7.875	XM_372002	CUCAGAUGCUGUUAACAGAtt	UCUGUUAACAGCAUCUGAGtt	303
tcag7.875	XM_372002	GGAUUACAACAUGUGAAUUtt	AAUUCACAUGUUGUAAUCCca	202
TEC	NM_003215	CUGAGAUUAUUGAAUAUCAtt	UGAUAUUCAAUAAUCUCAGga	108
TEC	NM_003215	CAAUAUGAAUGGUAUUGCAtt	UGCAAUACCAUUCAUAUUGat	74
TEC	NM_003215	CAGUCUCCCUUUAUACCAAtt	UUGGUAUAAAGGGAGACUGtg	48

TEK	NM_000459	CAAACCCGUUAAUCACUAUtt	AUAGUGAUUAACGGGUUUGta	116
TEK	NM_000459	CUUCUAUACAAACCCGUUAtt	UAACGGGUUUGUAUAGAAGct	107
TEK	NM_000459	GCUUCUAUACAAACCCGUUtt	AACGGGUUUGUAUAGAAGCtt	141
TESK1	NM_006285	GGCAAGUCAUGGUGCUGAAtt	UUCAGCACCAUGACUUGCCct	133
TESK1	NM_006285	CGACAGUCAGGGCAAGUCAtt	UGACUUGCCCUGACUGUCGgt	104
TESK1	NM_006285	GGACAGCUGCACGCUCUUAtt	UAAGAGCGUGCAGCUGUCCct	113
TESK2	NM_007170	GAAGUACAGCUCAUGAAUAtt	UAUUCAUGAGCUGUACUUCtt	97
TESK2	NM_007170	GAACAGUUGCUAGACAGUAtt	UACUGUCUAGCAACUGUUCca	163
TESK2	NM_007170	GCACUUACAGAGUAUAUCAtt	UGAUAUACUCUGUAAGUGCat	341
TEX14	NM_031272	GACCUAACCAGGUAGAUGAtt	UCAUCUACCUGGUUAGGUCcg	104
TEX14	NM_031272	CGAGUUAUUGAGUAUCAUAtt	UAUGAUACUCAAUAACUCGct	207
TEX14	NM_031272	GCAGUUUCGAAAUCAACGAtt	UCGUUGAUUUCGAAACUGCag	110
TGFBR1	NM_004612	GCAAUGGGCUUAGUAUUCUtt	AGAAUACUAAGCCCAUUGCat	150
TGFBR1	NM_004612	GCCUUAUUAUGAUCUUGUAtt	UACAAGAUCAUAAUAAGGCag	90
TGFBR1	NM_004612	GGUACUACGUUGAAAGACUtt	AGUCUUUCAACGUAGUACCct	67
TGFBR2	NM_003242	CCAGCAAUCCUGACUUGUUtt	AACAAGUCAGGAUUGCUGGtg	149
TGFBR2	NM_003242	GGAGAAAGAAUGACGAGAAtt	UUCUCGUCAUUCUUUCUCCat	115
TGFBR2	NM_003242	GCUUUGCUGAGGUCUAUAAtt	UUAUAGACCUCAGCAAAGCga	242
TIE1	NM_005424	GAGUUCAACUUAGAGACGAtt	UCGUCUCUAAGUUGAACUCca	147
TIE1	NM_005424	GAACCGAGGUUACUUGUAUtt	AUACAAGUAACCUCGGUUCtt	77
TIE1	NM_005424	GGUUACUUGUAUAUCGCUAtt	UAGCGAUAUACAAGUAACCtc	115
TK1	NM_003258	GGGAAGCCGCCUAUACCAAtt	UUGGUAUAGGCGGCUUCCCgg	121
TK1	NM_003258	GGGCCGAUGUUCUCAGGAAtt	UUCCUGAGAACAUCGGCCCga	145
TK1	NM_003258	GUCGCUUCCAGAUUGCUCAtt	UGAGCAAUCUGGAAGCGACgg	170
TK2	NM_004614	CUGUUGAUUUGAUAGUUUAtt	UAAACUAUCAAAUCAACAGac	260
TK2	NM_004614	GAACAAAAUCGGGAUCGAAtt	UUCGAUCCCGAUUUUGUUCaa	444
TK2	NM_004614	GCUCUGUGAUACCCAAUAAtt	UUAUUGGGUAUCACAGAGCaa	179
TLK1	NM_012290	CCUCAUCUGUUCGACCGAAtt	UUCGGUCGAACAGAUGAGGaa	112
TLK1	NM_012290	CUUGGAUACAGAUACGUUUtt	AAACGUAUCUGUAUCCAAGga	219
TLK1	NM_012290	GCGACUAUUUUGAAUACCAtt	UGGUAUUCAAAAUAGUCGCta	408
TLK2	NM_006852	GACUCGUUUUGUACAGUAUtt	AUACUGUACAAAACGAGUCag	88

TLK2	NM_006852	GCAUGUAGGGAAUACCGGAtt	UCCGGUAUUCCCUACAUGCat	284
TLK2	NM_006852	CAAGCAUGCAUGUAGGGAAtt	UUCCCUACAUGCAUGCUUGtg	326
TNIK	NM_015028	CGACAUACCCAGACUGAUAtt	UAUCAGUCUGGGUAUGUCGct	134
TNIK	NM_015028	GCUACGAGUUUACUAUCUUtt	AAGAUAGUAAACUCGUAGCtt	256
TNIK	NM_015028	GCUAGUUGAUCUCACGGUAtt	UACCGUGAGAUCAACUAGCag	184
TNK1	NM_003985	UCAACACCCAGGAAGCAUAtt	UAUGCUUCCUGGGUGUUGAtc	119
TNK1	NM_003985	CGCCCACCUUUAUCCUCUAtt	UAGAGGAUAAAGGUGGGCGtg	172
TNK1	NM_003985	AGGUAUCGGUCAUGAUGAAtt	UUCAUCAUGACCGAUACCUct	97
TNK2	NM_001010938	GGUGUUCAGUGGAAAGCGAtt	UCGCUUUCCACUGAACACCtt	94
TNK2	NM_001010938	AGGACUUUGAGGAACCGGAtt	UCCGGUUCCUCAAAGUCCUga	192
TNK2	NM_001010938	CGCAAGUCGUGGAUGAGUAtt	UACUCAUCCACGACUUGCGtt	150
TNNI3K	NM_015978	CCAUCUCGACUGACAAGAAtt	UUCUUGUCAGUCGAGAUGGgc	105
TNNI3K	NM_015978	GCAUUGACCUAGUCAAAUUtt	AAUUUGACUAGGUCAAUGCtc	222
TNNI3K	NM_015978	CAUUGCAUAUUGCAGCGUAtt	UACGCUGCAAUAUGCAAUGga	325
TPD52L3	NM_001001875	GGCUGAAAUUGUAACCCUAtt	UAGGGUUACAAUUUCAGCCtc	81
TPD52L3	NM_001001874	UCAGGUCUCCAACACCUAUtt	AUAGGUGUUGGAGACCUGAac	80
TPD52L3	NM_001001874	ACUCGACUUCUGAACUGGAtt	UCCAGUUCAGAAGUCGAGUgg	31
TPK1	NM_001042482	GCCUUUGGACAACUAUUUUtt	AAAAUAGUUGUCCAAAGGCtg	85
TPK1	NM_001042482	GGAGACUUUGAUUCUAUUAtt	UAAUAGAAUCAAAGUCUCCat	183
TPK1	NM_001042482	GCUUAUAUGAUAUCACCGAtt	UCGGUGAUAUCAUAUAAGCgg	316
TPR	NM_003292	GAGUCUGCGUUAUCGACAAtt	UUGUCGAUAACGCAGACUCtc	114
TPR	NM_003292	GAAGUUCAUACUAAGCGUAtt	UACGCUUAGUAUGAACUUCct	103
TPR	NM_003292	CAAUAUGAAGGUCGAAUUAtt	UAAUUCGACCUUCAUAUUGgg	137
TRAF3IP3	NM_025228	GACCAACAAUUAAGAACGAtt	UCGUUCUUAAUUGUUGGUCct	126
TRAF3IP3	NM_025228	GAACAGGAGAAACUCUUAAtt	UUAAGAGUUUCUCCUGUUCtt	93
TRAF3IP3	NM_025228	GGACCUACAAGAUCAACUAtt	UAGUUGAUCUUGUAGGUCCtg	90
TRIB1	NM_025195	CCCUUCUGGUUGGACGAUAtt	UAUCGUCCAACCAGAAGGGtg	116
TRIB1	NM_025195	GAUGAUGCUUUGUCAGACAtt	UGUCUGACAAAGCAUCAUCtt	140
TRIB1	NM_025195	GCAAGGUGUUUCCCAUUAAtt	UUAAUGGGAAACACCUUGCag	218
TRIB2	NM_021643	CCAGAGAUCUUGAACACCAtt	UGGUGUUCAAGAUCUCUGGgc	147
TRIB2	NM_021643	GAACUUGUCGCAUUGCGUUtt	AACGCAAUGCGACAAGUUCgg	202

TRIB2	NM_021643	GAAGAGUUGUCGUCUAUAAtt	UUAUAGACGACAACUCUUCga	473
TRIB3	NM_021158	CCAGCUCCUCUACGCCUUUtt	AAAGGCGUAGAGGAGCUGGgt	139
TRIB3	NM_021158	CGGUUGGAGUUGGAUGACAtt	UGUCAUCCAACUCCAACCGct	46
TRIB3	NM_021158	GGAAGAAGCGGUUGGAGUUtt	AACUCCAACCGCUUCUUCCtg	331
TRRAP	NM_003496	CUACGAUUCUGGUGGAAUAtt	UAUUCCACCAGAAUCGUAGca	212
TRRAP	NM_003496	GGAAUUACGGAGAUAUAGAtt	UCUAUAUCUCCGUAAUUCCtt	570
TRRAP	NM_003496	GAAUUACGGAGAUAUAGAAtt	UUCUAUAUCUCCGUAAUUCct	603
TSKS	NM_021733	GGAAGAUGCUGAAAUCAAAtt	UUUGAUUUCAGCAUCUUCCac	118
TSKS	NM_021733	GCACCAACGUGUCACUGCUtt	AGCAGUGACACGUUGGUGCag	361
TSKS	NM_021733	GCUGUUCACCGGCAUCGAAtt	UUCGAUGCCGGUGAACAGCtt	291
TSSK1B	NM_032028	CGUCGACUAUGGAGACAGAtt	UCUGUCUCCAUAGUCGACGgc	246
TSSK1B	NM_032028	CUCCUCGAGUUAAUCAAAAtt	UUUUGAUUAACUCGAGGAGgt	109
TSSK1B	NM_032028	CAAGAUCCAUAAACUUUUAtt	UAAAAGUUUAUGGAUCUUGcc	152
TSSK2	NM_053006	GCUCCAUCAUCAAGACUUAtt	UAAGUCUUGAUGAUGGAGCcg	232
TSSK2	NM_053006	GGUUCCUACGCAAAAGUCAtt	UGACUUUUGCGUAGGAACCct	197
TSSK2	NM_053006	GGACGGAUCUACAUCAUCAtt	UGAUGAUGUAGAUCCGUCCgt	186
TSSK3	NM_052841	GGCCUUCAAUUGAAGAAGUtt	ACUUCUUCAAUUGAAGGCCgg	143
TSSK3	NM_052841	GGUGUGGUCCUGUAUGUCAtt	UGACAUACAGGACCACACCca	295
TSSK3	NM_052841	GGGACCUACUCAAAAGUCAtt	UGACUUUUGAGUAGGUCCCtt	87
TSSK4	NM_174944	GCUCCACAACACCACUAAAtt	UUUAGUGGUGUUGUGGAGCtg	147
TSSK4	NM_174944	UGAUGACACCAAUCUCAAAtt	UUUGAGAUUGGUGUCAUCAaa	124
TSSK4	NM_174944	GAAGGCCUCUGAUGACUAUtt	AUAGUCAUCAGAGGCCUUCtt	152
TSSK6	NM_032037	CGGACUUCGUCAACAAGUUtt	AACUUGUUGACGAAGUCCGgg	123
TSSK6	NM_032037	GGGAAACUGUACAUCGUGAtt	UCACGAUGUACAGUUUCCCgt	206
TSSK6	NM_032037	CAGUUGCCCUUGUUCGGAAtt	UUCCGAACAAGGGCAACUGcg	199
TTBK1	NM_032538	GAACGAGAAGUUUAACUAUtt	AUAGUUAAACUUCUCGUUCct	143
TTBK1	NM_032538	CAAAGUUGAGAGGACCUUUtt	AAAGGUCCUCUCAACUUUGgt	127
TTBK1	NM_032538	GACUACCAGUUGAUCAUGUtt	ACAUGAUCAACUGGUAGUCgg	153
TTBK2	NM_173500	GCAUCUUUCUAGACCAUAUtt	AUAUGGUCUAGAAAGAUGCtg	93
TTBK2	NM_173500	GUCAUGACAUGUUACCCAAtt	UUGGGUAACAUGUCAUGACca	124
TTBK2	NM_173500	GAAUGAUCGAUUCAACUAUtt	AUAGUUGAAUCGAUCAUUCct	209

TTC33	NM_012382	CACUUUUCACCAAAGUCAAtt	UUGACUUUGGUGAAAAGUGtg	178
TTC33	NM_012382	GUAGCACAGAGGAUUAAAAtt	UUUUAAUCCUCUGUGCUACct	138
TTC33	NM_012382	GAUGCUACCCUAUACGAGAtt	UCUCGUAUAGGGUAGCAUCat	378
TTK	NM_003318	GGUUGUGCCUGGAUCUAAAtt	UUUAGAUCCAGGCACAACCaa	93
TTK	NM_003318	GAUAGUUACCGGAACGAAAtt	UUUCGUUCCGGUAACUAUCaa	187
TTK	NM_003318	CUCUUGAUAGUUACCGGAAtt	UUCCGGUAACUAUCAAGAGtt	127
TWF1	NM_002822	GGAAUUCGAAGACUAAUUAtt	UAAUUAGUCUUCGAAUUCCtc	117
TWF1	NM_002822	GAAAGAUCGAGAUAGACAAtt	UUGUCUAUCUCGAUCUUUCta	67
TWF1	NM_002822	GGAUUCAGCUCGUUACCAUtt	AUGGUAACGAGCUGAAUCCtt	34
TWF2	NM_007284	AGUCUGUAGUGUUCAUCUAtt	UAGAUGAACACUACAGACUca	106
TWF2	NM_007284	AGAUGAAGCUGGACCUAGAtt	UCUAGGUCCAGCUUCAUCUgg	200
TWF2	NM_007284	AGCAGAAAAUGGUCAACUAtt	UAGUUGACCAUUUUCUGCUtg	219
TXK	NM_003328	GGAAUGACAAGGUACGUUUtt	AAACGUACCUUGUCAUUCCaa	127
TXK	NM_003328	CCCUGUAAUUUAGCCUUAAtt	UUAAGGCUAAAUUACAGGGtt	93
TXK	NM_003328	GUGCAGAAGCGACAAAUGAtt	UCAUUUGUCGCUUCUGCACtg	181
TXNDC3	NM_016616	GACAAGUAGUAUUAUCGGAtt	UCCGAUAAUACUACUUGUCtt	193
TXNDC3	NM_016616	CGAACGAUCUGAGGAUCAAtt	UUGAUCCUCAGAUCGUUCGtt	150
TXNDC3	NM_016616	CAACUUCUAUAGUCGAAUAtt	UAUUCGACUAUAGAAGUUGac	165
TXNDC6	NM_178130	GAAAGACUGAUGAGAUUAUtt	AUAAUCUCAUCAGUCUUUCca	65
TXNDC6	NM_178130	GGUUUGAAAUUCUAACAAAtt	UUUGUUAGAAUUUCAAACCca	95
TXNDC6	NM_178130	AGAUGAGGCUCUUUCUGAUtt	AUCAGAAAGAGCCUCAUCUtt	40
TYK2	NM_003331	GAUGCUAUAUUUCCGCAUAtt	UAUGCGGAAAUAUAGCAUCag	178
TYK2	NM_003331	CAUCCACAUUGCACAUAAAtt	UUUAUGUGCAAUGUGGAUGca	120
TYK2	NM_003331	GGAGUAUAAGUUCUACUAUtt	AUAGUAGAACUUAUACUCCtt	238
TYRO3	NM_006293	GAGCUUUACUUGUCUGCGAtt	UCGCAGACAAGUAAAGCUCgg	140
TYRO3	NM_006293	CAGUGACUGUCGGUACAUAtt	UAUGUACCGACAGUCACUGgg	91
TYRO3	NM_006293	CAAGCGACAUUGAAGAGUUtt	AACUCUUCAAUGUCGCUUGag	36
UCK1	NM_031432	CACGAGGAGUGGACAAUAUtt	AUAUUGUCCACUCCUCGUGgg	121
UCK1	NM_031432	AGCAGAUUCUGACGCAGUAtt	UACUGCGUCAGAAUCUGCUcc	244
UCK1	NM_031432	CUGCGGACGUGGUUCUGUUtt	AACAGAACCACGUCCGCAGgg	133
UCK2	NM_012474	AGCUUCUACCGUGUCCUUAtt	UAAGGACACGGUAGAAGCUat	65

UCK2	NM_012474	GAUUUUAUCUCAGUACAUUtt	AAUGUACUGAGAUAAAAUCtg	151
UCK2	NM_012474	GUACGAGACCUGUUCCAGAtt	UCUGGAACAGGUCUCGUACct	362
UCKL1	NM_017859	GCAUUUCCGCGAGUGAGAAtt	UUCUCACUCGCGGAAAUGCat	174
UCKL1	NM_017859	CAAGCAGUACAACAAGUUUtt	AAACUUGUUGUACUGCUUGat	241
UCKL1	NM_017859	CAAUGAACACGGCACGCAAtt	UUGCGUGCCGUGUUCAUUGta	308
UHMK1	NM_175866	GGAUGUCAGUGUUUCGGAAtt	UUCCGAAACACUGACAUCCag	171
UHMK1	NM_175866	CCACGUAACAUAUUGUGGAtt	UCCACAAUAUGUUACGUGGtt	111
UHMK1	NM_175866	CGAGUAUGGUUUCCGCAAAtt	UUUGCGGAAACCAUACUCGgc	123
ULK1	XM_001133335	GCAUCGGCACCAUCGUCUAtt	UAGACGAUGGUGCCGAUGCtc	222
ULK1	XM_001133335	GCAUGGACUUCGAUGAGUUtt	AACUCAUCGAAGUCCAUGCgg	90
ULK1	XM_001133335	CGCCUGUUCUACGAGAAGAtt	UCUUCUCGUAGAACAGGCGca	137
ULK2	NM_014683	GCAGAUUAUUUGCAAGCGAtt	UCGCUUGCAAAUAAUCUGCga	93
ULK2	NM_014683	GCUCGUUACCUACAUAGUAtt	UACUAUGUAGGUAACGAGCaa	159
ULK2	NM_014683	GAAUCUGAACGAACGAUAUtt	AUAUCGUUCGUUCAGAUUCtt	228
ULK3	XM_001134013	CUGUCUCGCUUCAUCCAUAtt	UAUGGAUGAAGCGAGACAGgt	161
ULK3	XM_001134013	GCAUGAACGGAAUAUCUCUtt	AGAGAUAUUCCGUUCAUGCag	358
ULK3	XM_001134013	CAGCGGAAGGAGGCAAUUAtt	UAAUUGCCUCCUUCCGCUGgg	259
ULK4	XM_929989	CCACAUCUCUACUCGUGAAtt	UUCACGAGUAGAGAUGUGGtt	133
ULK4	XM_929989	CCUCGUAACUUCACAGGUAtt	UACCUGUGAAGUUACGAGGtg	99
ULK4	XM_929989	GCUCCACGGUUGUUGACUAtt	UAGUCAACAACCGUGGAGCga	219
VRK1	NM_003384	GCAGUUGGAGAGAUAAUAAtt	UUAUUAUCUCUCCAACUGCaa	76
VRK1	NM_003384	GAGAUUCCAAAAUUAGAUAtt	UAUCUAAUUUUGGAAUCUCta	172
VRK1	NM_003384	GAUACGGAAUGGUCAAACAtt	UGUUUGACCAUUCCGUAUCtt	169
VRK2	NM_006296	GGAUUUGGAUUGAUAUAUUtt	AAUAUAUCAAUCCAAAUCCtc	74
VRK2	NM_006296	GGAGGAUUUGGAUUGAUAUtt	AUAUCAAUCCAAAUCCUCCag	148
VRK2	NM_006296	GACUAGGAAUAGAUUUACAtt	UGUAAAUCUAUUCCUAGUCtt	419
VRK3	NM_001025778	GGAACAACCUAGAAGCUUUtt	AAAGCUUCUAGGUUGUUCCtc	96
VRK3	NM_001025778	GAAUGAGUAUGUUCAUGGAtt	UCCAUGAACAUACUCAUUCtc	203
VRK3	NM_001025778	CCACCUCACUUGAAGCUUUtt	AAAGCUUCAAGUGAGGUGGtc	82
WEE1	NM_003390	CAUCUCGACUUAUUGGAAAtt	UUUCCAAUAAGUCGAGAUGtt	87
WEE1	NM_003390	CAAUUACGAAUAGAAUUGAtt	UCAAUUCUAUUCGUAAUUGtt	216

WEE1	NM_003390	GUGUCGUCGUAGAAAGAGAtt	UCUCUUUCUACGACGACACtg	107
WNK1	NM_018979	CAUCAUCCCUUAGUCUACAtt	UGUAGACUAAGGGAUGAUGca	107
WNK1	NM_018979	CCAGCGUAGUUUCAAGUAUtt	AUACUUGAAACUACGCUGGaa	161
WNK1	NM_018979	CAAUGAGUCAGAUAUCGAAtt	UUCGAUAUCUGACUCAUUGtc	137
WNK2	NM_006648	CGUAUAUGGUGGAGCAUGAtt	UCAUGCUCCACCAUAUACGtg	168
WNK2	NM_006648	CUAUAAGUCUAGUAGCAAAtt	UUUGCUACUAGACUUAUAGgt	165
WNK2	NM_006648	GGAGUAUGCUAGGCUAUGAtt	UCAUAGCCUAGCAUACUCCtg	153
WNK3	NM_001002838	GCCUCACGUUUGUCAGUAUtt	AUACUGACAAACGUGAGGCat	194
WNK3	NM_001002838	GAAGAUUGGUGAUCUAGGAtt	UCCUAGAUCACCAAUCUUCac	230
WNK3	NM_001002838	GGAGUUUGCAGACAACCGAtt	UCGGUUGUCUGCAAACUCCag	236
WNK4	NM_032387	GGCUUUUGCCCUAUCCAUUtt	AAUGGAUAGGGCAAAAGCCga	159
WNK4	NM_032387	AGAUGUACGAGGAAAAGUAtt	UACUUUUCCUCGUACAUCUcg	69
WNK4	NM_032387	ACAUCGUCCGCUUCUAUGAtt	UCAUAGAAGCGGACGAUGUtg	133
XYLB	NM_005108	GGAGGAGCAUCUCACAAUAtt	UAUUGUGAGAUGCUCCUCCtg	143
XYLB	NM_005108	GCAUCUCACAAUAGAGAAAtt	UUUCUCUAUUGUGAGAUGCtc	187
XYLB	NM_005108	GUGUGCAUUUUGACAGAGAtt	UCUCUGUCAAAAUGCACACtt	165
YES1	NM_005433	CAGGUAUGGUGAACCGUGAtt	UCACGGUUCACCAUACCUGga	97
YES1	NM_005433	CCACGAAAGUAGCAAUCAAtt	UUGAUUGCUACUUUCGUGGtt	178
YES1	NM_005433	GUAUGGUCGGUUUACAAUAtt	UAUUGUAAACCGACCAUACag	263
YSK4	NM_001018046	CCACACUGGUUAAUGAAGAtt	UCUUCAUUAACCAGUGUGGaa	113
YSK4	NM_001018046	GGUACAUCGCGAUAUCAAAtt	UUUGAUAUCGCGAUGUACCac	211
YSK4	NM_001018046	GAAAUAAUGUUAUGCUCAUtt	AUGAGCAUAACAUUAUUUCct	147
ZAK	NM_133646	GGGAGAUGCUAACAAGGGAtt	UCCCUUGUUAGCAUCUCCCag	114
ZAK	NM_133646	GGAUAUCACAGGACAAGGAtt	UCCUUGUCCUGUGAUAUCCat	68
ZAK	NM_133646	GGAUCACUCUAUGAUUACAtt	UGUAAUCAUAGAGUGAUCCca	152
ZAP70	NM_001079	AGAACUUUGUGCACCGUGAtt	UCACGGUGCACAAAGUUCUtc	80
ZAP70	NM_001079	GAACUGUACGCACUCAUGAtt	UCAUGAGUGCGUACAGUUCgg	125
ZAP70	NM_001079	GCGCGAUAACCUCCUCAUAtt	UAUGAGGAGGUUAUCGCGCtt	212
ZC3HC1	NM_016478	GAACAACCUUCAUUGGAAUtt	AUUCCAAUGAAGGUUGUUCcg	190
ZC3HC1	NM_016478	CAAGGAAAGUAUUCCGAAUtt	AUUCGGAAUACUUUCCUUGat	260
ZC3HC1	NM_016478	CACUCGUCUGUGCAAAAUAtt	UAUUUUGCACAGACGAGUGga	93

ZMYND8	NM_012408	CUAUCACGACGAAAACGGAtt	UCCGUUUUCGUCGUGAUAGga	157
ZMYND8	NM_012408	CGAUAGUGAGUAUAUCAGUtt	ACUGAUAUACUCACUAUCGct	249
ZMYND8	NM_012408	GCUCCUAUCACGACGAAAAtt	UUUUCGUCGUGAUAGGAGCtg	219
