# Supplementary material for: Automated High-Throughput RNAi Screening in Human Cells Combined with Reporter mRNA Transfection to Identify Novel Regulators of Translation
Source: PLoS One. 2012 Sep 27;7(9):e45943. doi: 10.1371/journal.pone.0045943 (PMC3459937; doi:10.1371/journal.pone.0045943)
Supplement: Table S2 — Phosphatase library. Gene symbol, RefSeq, sense- and antisense siRNA sequences and normalized luciferase values for the screened 298 phosphatases. (PDF) [file pone.0045943.s004.pdf]

Supplementary table 2

Gene Symbol	RefSeq Accession Number	Sense siRNA Sequence 5'-3'	Antisense siRNA Sequence 5'-3'	Normalized luciferase values
ACP1	NM_004300	GGGUAACAUUUGUCGAUCAtt	UGAUCGACAAAUGUUACCCag	302
ACP1	NM_004300	GGAAACUUGUAACCGAUCAtt	UGAUCGGUUACAAGUUUCCtg	241
ACP1	NM_001040649	AACUUGUAACCGAUCAAAAtt	UUUUGAUCGGUUACAAGUUtc	173
ACP2	NM_001610	GUAUCAGAAUGAGAGUUCUtt	AGAACUCUCAUUCUGAUACtc	125
ACP2	NM_001610	GGACUCUCAUGAGUGCUGAtt	UCAGCACUCAUGAGAGUCCgg	246
ACP2	NM_001610	CGGAGUCUGCGCUUCGUUAtt	UAACGAAGCGCAGACUCCGgg	224
ACP5	NM_001611	AGAUCCCACAGACCAAUGUtt	ACAUUGGUCUGUGGGAUCUtg	282
ACP5	NM_001611	CCCAGAUUGCAUACUCUAAtt	UUAGAGUAUGCAAUCUGGGca	321
ACP5	NM_001611	UGACCGCUCCCUUCGCAAAtt	UUUGCGAAGGGAGCGGUCAga	175
ACP6	NM_016361	GCACGGAUGAUCGAACAGAtt	UCUGUUCGAUCAUCCGUGCaa	81
ACP6	NM_016361	CACUAACAUUUUUCGGAAUtt	AUUCCGAAAAAUGUUAGUGga	268
ACP6	NM_016361	CUGGUGGUCCGAAACCAUAtt	UAUGGUUUCGGACCACCAGct	230
ACPL2	NM_001037172	CUCCUACGUUUGAAAAACAtt	UGUUUUUCAAACGUAGGAGgt	108
ACPL2	NM_001037172	CACUGUAUGUCAUUCCCAAtt	UUGGGAAUGACAUACAGUGgg	172
ACPL2	NM_001037172	GCUAUUGCCCGGUAAGAAAtt	UUUCUUACCGGGCAAUAGCag	211
ACPP	NM_001099	GCUGCACCCUUAUAAGGAUtt	AUCCUUAUAAGGGUGCAGCct	370
ACPP	NM_001099	GGACUUUGAUGAGUGCUAUtt	AUAGCACUCAUCAAAGUCCgg	198
ACPP	NM_001099	GCUAGAUGUUUACAACGGAtt	UCCGUUGUAAACAUCUAGCgc	227
ACPT	NM_033068	AGAUCUCGGCUUUGGAUAUtt	AUAUCCAAAGCCGAGAUCUgg	154
ACPT	NM_033068	GACUGUCGCUGGUUGGAGAtt	UCUCCAACCAGCGACAGUCcc	276
ACPT	NM_033068	AGCCCACGGUCUUCCACUAtt	UAGUGGAAGACCGUGGGCUtg	113
ACYP1	NM_203488	GUGUUUUUCCGUAAGCAUAtt	UAUGCUUACGGAAAAACACcc	125
ACYP1	NM_203488	GGUGCGUCAUAUGCAGGAAtt	UUCCUGCAUAUGACGCACCtt	377
ACYP1	NM_203488	GAGGAAGUCCUAAAUCACAtt	UGUGAUUUAGGACUUCCUCtt	53
ACYP2	NM_138448	CAUUGACCGCACAAACUUUtt	AAAGUUUGUGCGGUCAAUGcg	77
ACYP2	NM_138448	GGUGAAGAAUACCAGCAAAtt	UUUGCUGGUAUUCUUCACCca	251
ACYP2	NM_138448	CUUCAGAAUGUAUACAGAAtt	UUCUGUAUACAUUCUGAAGca	186
ALPI	NM_001631	GACAUACAAUGUGGACAGAtt	UCUGUCCACAUUGUAUGUCtt	89
ALPI	NM_001631	CGUCCAUCCUGUACGGCAAtt	UUGCCGUACAGGAUGGACGtg	173
ALPI	NM_001631	GGGCUACGUGUUCAACUCAtt	UGAGUUGAACACGUAGCCCgg	178
ALPL	NM_000478	CUGCCUUACUAACUCCUUAtt	UAAGGAGUUAGUAAGGCAGgt	76
ALPL	NM_000478	GUAUGAGAGUGACGAGAAAtt	UUUCUCGUCACUCUCAUACtc	167

ALPL	NM_000478	CAAGCGCAAGAGACACUGAtt	UCAGUGUCUCUUGCGCUUGgt	93
ALPP	NM_001632	GGACACUGGGCAUAGAUUUtt	AAAUCUAUGCCCAGUGUCCag	123
ALPP	NM_001632	GGAAGUGUUUGUAAUCCCAtt	UGGGAUUACAAACACUUCCtt	150
ALPP	NM_001632	GCACCCAACUGAGAUCCGAtt	UCGGAUCUCAGUUGGGUGCtg	60
ALPPL2	NM_031313	GGACAUUGAUGUGAUCCUAtt	UAGGAUCACAUCAAUGUCCat	78
ALPPL2	NM_031313	AGACAUACAGUGUAGACAAtt	UUGUCUACACUGUAUGUCUtg	451
ALPPL2	NM_031313	CCACUGGGCUAAUUCUACAtt	UGUAGAAUUAGCCCAGUGGag	179
ANP32A	NM_006305	UCACAUAUCUCGACGGCUAtt	UAGCCGUCGAGAUAUGUGAgt	88
ANP32A	NM_006305	UGAACUAAGCGAUAACAGAtt	UCUGUUAUCGCUUAGUUCAag	211
ANP32A	NM_006305	CAAUCGCAAACUUACCAAAtt	UUUGGUAAGUUUGCGAUUGag	133
ANP32B	NM_006401	CCUUGGAACCUUUGAAAAAtt	UUUUUCAAAGGUUCCAAGGtg	129
ANP32B	NM_006401	GUCUGGACAUGUUAGCUGAtt	UCAGCUAACAUGUCCAGACct	392
ANP32B	NM_006401	AGAGUUCCUCAGUUUAAUAtt	UAUUAAACUGAGGAACUCUaa	150
ANP32C	NM_012403	GGACAACAGUCGGUCGAAUtt	AUUCGACCGACUGUUGUCCag	95
ANP32C	NM_012403	CUGGAAUUCUUAAGUAAAAtt	UUUUACUUAAGAAUUCCAGtt	101
ANP32C	NM_012403	GAACUGGAAUUCUUAAGUAtt	UACUUAAGAAUUCCAGUUCtt	104
ANP32D	NM_012404	CCUCAUACAUCUAAAUUUAtt	UAAAUUUAGAUGUAUGAGGtt	122
ANP32D	NM_012404	GGACAACAGUCAGUCAAAUtt	AUUUGACUGACUGUUGUCCag	115
ANP32D	NM_012404	GCCUCACCUCAAUUGCAAAtt	UUUGCAAUUGAGGUGAGGCct	172
ANP32E	NM_030920	GAUUUGAUCAGGAGGAUAAtt	UUAUCCUCCUGAUCAAAUCca	126
ANP32E	NM_030920	CUGUUUAACUGUGAGAUCAtt	UGAUCUCACAGUUAAACAGgt	158
ANP32E	NM_030920	GAGCUUAGUGAUAAUAUAAtt	UUAUAUUAUCACUAAGCUCca	143
ASNA1	NM_004317	GCCCUUUCAUCUCACAGAUtt	AUCUGUGAGAUGAAAGGGCtg	153
ASNA1	NM_004317	GGCUUAUGCAGAUCAAGAAtt	UUCUUGAUCUGCAUAAGCCgg	271
ASNA1	NM_004317	CAACAUCUCAGAUGCUUUUtt	AAAAGCAUCUGAGAUGUUGtg	520
ATP6V0E1	NM_003945	UGCCUUGGUUCAUCCCUAAtt	UUAGGGAUGAACCAAGGCAcc	95
ATP6V0E1	NM_003945	CCCUCUCUUUGGACCGCAAtt	UUGCGGUCCAAAGAGAGGGtt	170
ATP6V0E1	NM_003945	GACCGCAAUUGAAAAAUGAtt	UCAUUUUUCAAUUGCGGUCca	160
ATP6V0E2	NM_145230	GCCGUCUGCUGUUACCUCUtt	AGAGGUAACAGCAGACGGCgg	148
ATP6V0E2	NM_145230	GCGGAGUGAUCAUCACCAUtt	AUGGUGAUGAUCACUCCGCgg	244
ATP6V0E2	NM_145230	ACGUCUAGAUGUGAAAUUUtt	AAAUUUCACAUCUAGACGUgt	93
BPNT1	NM_006085	GAGGAAUUAUGACUACUAUtt	AUAGUAGUCAUAAUUCCUCag	284
BPNT1	NM_006085	CAUAUUCUAUUGCUCAAAAtt	UUUUGAGCAAUAGAAUAUGcg	128
BPNT1	NM_006085	GAAUGAUAGUCAGACGUGUtt	ACACGUCUGACUAUCAUUCct	145
C21orf6	NM_016940	GGAGAUGUUUGUAUACUGAtt	UCAGUAUACAAACAUCUCCat	136
C21orf6	NM_016940	CUCUAUCAGUUCUUAAACAtt	UGUUUAAGAACUGAUAGAGct	120

C21orf6	NM_016940	GGAUGUAUCUGACGAAAAAtt	UUUUUCGUCAGAUACAUCCag	134
C3orf48	NM_144714	GGAGUGUCAUAUAUUCUCAtt	UGAGAAUAUAUGACACUCCtg	168
C3orf48	NM_144714	CAAAUACCAGCAUACUAUAtt	UAUAGUAUGCUGGUAUUUGtt	91
C3orf48	NM_144714	CACAUUACCCUGUUCCAUAtt	UAUGGAACAGGGUAAUGUGgt	251
CABIN1	NM_012295	GGAUUGAUUUGUCGGACUAtt	UAGUCCGACAAAUCAAUCCtt	169
CABIN1	NM_012295	GGAUUGCCGGUACAGCAAAtt	UUUGCUGUACCGGCAAUCCtt	287
CABIN1	NM_012295	GCGAUUCUAUGUGCGAGUAtt	UACUCGCACAUAGAAUCGCag	199
CANT1	NM_138793	UGAUUGUUUUCAAUGGGAAtt	UUCCCAUUGAAAACAAUCAgg	164
CANT1	NM_138793	ACCUGGUUCAGUUACCUGAtt	UCAGGUAACUGAACCAGGUgt	299
CANT1	NM_138793	GAAUCGCAGUUAUCGCAGAtt	UCUGCGAUAACUGCGAUUCga	191
CCDC104	NM_080667	GAAGAGAUGAAAAUCCUGAtt	UCAGGAUUUUCAUCUCUUCgt	138
CCDC104	NM_080667	GCAUUUUGCUAAUCAGUCAtt	UGACUGAUUAGCAAAAUGCat	246
CCDC104	NM_080667	GCAAAUUGACCUAUACAGAtt	UCUGUAUAGGUCAAUUUGCtt	192
CDC14A	NM_033313	CCUAUGCAGUAAUCUAUUUtt	AAAUAGAUUACUGCAUAGGca	173
CDC14A	NM_033313	GUUCAUGAAAGAUCGGUUAtt	UAACCGAUCUUUCAUGAACtc	305
CDC14A	NM_033313	CUACACCUGUUUUGACCAAtt	UUGGUCAAAACAGGUGUAGtg	189
CDC14B	NM_003671	AGAAUUCCUAGAUAUCUGUtt	ACAGAUAUCUAGGAAUUCUtt	138
CDC14B	NM_003671	AGAUCAGCAAGAACCCGAAtt	UUCGGGUUCUUGCUGAUCUtg	192
CDC14B	NM_003671	AGACAUCCUAUAUUCCUUUtt	AAAGGAAUAUAGGAUGUCUct	224
CDC14C	XM_001125780	AACAUUCUCCCGAGACUUAtt	UAAGUCUCGGGAGAAUGUUgg	201
CDC14C	XM_001125780	GAAAAGGCGAAGACAAUCAtt	UGAUUGUCUUCGCCUUUUCag	311
CDC14C	XM_001125780	AGGCGAAGACAAUCAAAAAtt	UUUUUGAUUGUCUUCGCCUtt	114
CDC25A	NM_201567	GGGUUAUCUCUUUCAUACAtt	UGUAUGAAAGAGAUAACCCtt	152
CDC25A	NM_201567	CAAGGGACCUUAUAGGAGAtt	UCUCCUAUAAGGUCCCUUGgg	337
CDC25A	NM_201567	CAUCGACUGUCGAUACCCAtt	UGGGUAUCGACAGUCGAUGat	130
CDC25B	NM_021873	GGAUCAUUCGAAACGAGCAtt	UGCUCGUUUCGAAUGAUCCgg	101
CDC25B	NM_021873	GAGAGCUGAUUGGAGAUUAtt	UAAUCUCCAAUCAGCUCUCgg	126
CDC25B	NM_021873	GCAACAUCGUGGAUAAGUUtt	AACUUAUCCACGAUGUUGCtg	51
CDC25C	NM_001790	CAGGGAGCCUUAAACUUAUtt	AUAAGUUUAAGGCUCCCUGga	62
CDC25C	NM_001790	CCUUUACCGUCUGUCCAGAtt	UCUGGACAGACGGUAAAGGaa	167
CDC25C	NM_001790	GAACUCCAGUGGGCAAAUUtt	AAUUUGCCCACUGGAGUUCta	68
CDKN3	NM_005192	CCUUAAAAAUUACCGAAAAtt	UUUUCGGUAAUUUUUAAGGca	202
CDKN3	NM_005192	GACUCCAAUUCAUAUAUCAtt	UGAUAUAUGAAUUGGAGUCtg	110
CDKN3	NM_005192	CCAUCAAGCAAUACAAUUAtt	UAAUUGUAUUGCUUGAUGGtc	82
CHP	NM_007236	GAGUAAAUAUCUCAGAUGAtt	UCAUCUGAGAUAUUUACUCcg	111
CHP	NM_007236	AGAUGUGAAUGGACCCGAAtt	UUCGGGUCCAUUCACAUCUtt	319

CHP	NM_007236	GCAUGAUGGUCGGAGUAAAtt	UUUACUCCGACCAUCAUGCgt	136
CHTF18	NM_022092	UGGACAUUCUUGCACCCAAtt	UUGGGUGCAAGAAUGUCCAgg	129
CHTF18	NM_022092	CAGCACCCGUGAAAAGCAAtt	UUGCUUUUCACGGGUGCUGta	111
CHTF18	NM_022092	AGGGCUUGUUUGACAACUUtt	AAGUUGUCAAACAAGCCCUgg	69
CIB2	NM_006383	GACUGCACCUUCUUCAAUAtt	UAUUGAAGAAGGUGCAGUCct	143
CIB2	NM_006383	GGAAAAGCAGAUAACCCAAtt	UUGGGUUAUCUGCUUUUCCct	167
CIB2	NM_006383	GCUGCAUUCGCGAUUCUAUtt	AUAGAAUCGCGAAUGCAGCtt	217
CIB3	NM_054113	GGCUUACUAUGCUUUUAAAtt	UUUAAAAGCAUAGUAAGCCtt	127
CIB3	NM_054113	UGUUUUCCGUGAUGAGUGAtt	UCACUCAUCACGGAAAACAtg	112
CIB3	NM_054113	GGAGCAGACGGUGACCAAAtt	UUUGGUCACCGUCUGCUCCag	131
CTDP1	NM_048368	GACUCAAUGGUUUGCAUUAtt	UAAUGCAAACCAUUGAGUCtc	207
CTDP1	NM_048368	CAAACUUCCCGAUAGAGAAtt	UUCUCUAUCGGGAAGUUUGtc	277
CTDP1	NM_048368	GGGCACGGGUGAUAUGAAUtt	AUUCAUAUCACCCGUGCCCtg	130
CTDSP1	NM_182642	GCCCAGGACUCAGACAAGAtt	UCUUGUCUGAGUCCUGGGCct	109
CTDSP1	NM_182642	CGGACUUCAUCAUCCCUGUtt	ACAGGGAUGAUGAAGUCCGcg	109
CTDSP1	NM_182642	GCCUCGUGGUUUGACAACAtt	UGUUGUCAAACCACGAGGCca	126
CTDSP2	NM_005730	CGUAUAAGGAGGAAGCAAAtt	UUUGCUUCCUCCUUAUACGca	156
CTDSP2	NM_005730	ACAUCUUCAAGGCCCUUUUtt	AAAAGGGCCUUGAAGAUGUta	101
CTDSP2	NM_005730	UGACCUCGAUGAAACCCUUtt	AAGGGUUUCAUCGAGGUCAat	111
CTDSPL	NM_005808	GCCUAUUAGUAAUGCUGAUtt	AUCAGCAUUACUAAUAGGCtt	148
CTDSPL	NM_005808	GGUCAUUGAUUUAGAUGAAtt	UUCAUCUAAAUCAAUGACCac	88
CTDSPL	NM_005808	CACAGUUCGUUUAAGCCUAtt	UAGGCUUAAACGAACUGUGca	130
DOLPP1	NM_020438	CAGUUUAUGUGGUUCUUCUtt	AGAAGAACCACAUAAACUGgg	333
DOLPP1	NM_020438	AGUUCUUCCUAAUCCGAGAtt	UCUCGGAUUAGGAAGAACUcg	380
DOLPP1	NM_020438	AGAAUGCACCAAACAAACAtt	UGUUUGUUUGGUGCAUUCUta	165
DUPD1	NM_001003892	AGCUCUACAUUGGCGAUGAtt	UCAUCGCCAAUGUAGAGCUtg	175
DUPD1	NM_001003892	GCCUACUCAUCUGCCAAGAtt	UCUUGGCAGAUGAGUAGGCat	218
DUPD1	NM_001003892	UCCAGCAAGUGGCCAAGAAtt	UUCUUGGCCACUUGCUGGAtg	120
DUSP1	NM_004417	CCACCACCGUGUUCAACUUtt	AAGUUGAACACGGUGGUGGtg	166
DUSP1	NM_004417	GGACUAAUCGAGUCAAGCUtt	AGCUUGACUCGAUUAGUCCtc	318
DUSP1	NM_004417	AGGCCUUUGAGUUUGUGAAtt	UUCACAAACUCAAAGGCCUcg	392
DUSP10	NM_007207	AGAAAGGCCUGUUCAACUAtt	UAGUUGAACAGGCCUUUCUca	120
DUSP10	NM_007207	GACCAUGACUGAUGCUUAUtt	AUAAGCAUCAGUCAUGGUCat	217
DUSP10	NM_007207	AGUUCGAGGAAGACCUAAAtt	UUUAGGUCUUCCUCGAACUct	123
DUSP11	NM_003584	GGACUCGUUUCAUUGCUUUtt	AAAGCAAUGAAACGAGUCCca	174
DUSP11	NM_003584	GACCCAAAGUUUGCAACAAtt	UUGUUGCAAACUUUGGGUCtg	133

DUSP11	NM_003584	GAUAUUUGAUUGAUGUAGAtt	UCUACAUCAAUCAAAUAUCtg	218
DUSP12	NM_007240	GGAUAACACCUGCUUUUCAtt	UGAAAAGCAGGUGUUAUCCac	127
DUSP12	NM_007240	AGGCGAUCAUUAUUUCGAAtt	UUCGAAAUAAUGAUCGCCUgc	299
DUSP12	NM_007240	GAAGUGGACCUAUAGCCUUtt	AAGGCUAUAGGUCCACUUCct	286
DUSP13	NM_016364	CAAUAUCUGCCCUAACUCAtt	UGAGUUAGGGCAGAUAUUGcg	146
DUSP13	NM_016364	GAAUGUCCCUGGAGUACUAtt	UAGUACUCCAGGGACAUUCca	171
DUSP13	NM_016364	CACUGAACCAUAUCGAUGAtt	UCAUCGAUAUGGUUCAGUGtg	275
DUSP14	NM_007026	CAUUGUUAAUGCUACCAUUtt	AAUGGUAGCAUUAACAAUGca	138
DUSP14	NM_007026	GGAAGUCGACAGUUAAAAUtt	AUUUUAACUGUCGACUUCCca	226
DUSP14	NM_007026	GGAGGCAACUGAUAGACUAtt	UAGUCUAUCAGUUGCCUCCag	215
DUSP16	NM_030640	GGUUGUAGUUUACGAUCAAtt	UUGAUCGUAAACUACAACCtt	167
DUSP16	NM_030640	GCUGGAAAGUGGAACGGAAtt	UUCCGUUCCACUUUCCAGCag	175
DUSP16	NM_030640	GGACAAAGUGUUAAUUACAtt	UGUAAUUAACACUUUGUCCtg	375
DUSP18	NM_152511	AGCUCAUGCUGUCUAGCAAtt	UUGCUAGACAGCAUGAGCUtg	85
DUSP18	NM_152511	GCCUCUCGCAGAUAACCAAtt	UUGGUUAUCUGCGAGAGGCcg	66
DUSP18	NM_152511	AGUACAUGCAGGUACCUGUtt	ACAGGUACCUGCAUGUACUgg	113
DUSP19	NM_080876	GACUUUACAUAUAAGAGCAtt	UGCUCUUAUAUGUAAAGUCac	74
DUSP19	NM_080876	GACCUUCCAUAUGUCCAAAtt	UUUGGACAUAUGGAAGGUCtt	170
DUSP19	NM_080876	GGAGCAGCUUCGUACAUAUtt	AUAUGUACGAAGCUGCUCCat	248
DUSP2	NM_004418	GUCCCGAUCUGUGCUCUGAtt	UCAGAGCACAGAUCGGGACag	239
DUSP2	NM_004418	ACUUAGCACUUUAUAUUUAtt	UAAAUAUAAAGUGCUAAGUtt	190
DUSP2	NM_004418	GGCCUUUUCCGCUACAAGAtt	UCUUGUAGCGGAAAAGGCCct	213
DUSP21	NM_022076	GGUGGAAGUGGUCAACGUAtt	UACGUUGACCACUUCCACCga	144
DUSP21	NM_022076	CAACAACGGCUUUUGGGAAtt	UUCCCAAAAGCCGUUGUUGgg	167
DUSP21	NM_022076	GGGAACAGCUCAUCAAUUAtt	UAAUUGAUGAGCUGUUCCCaa	138
DUSP22	XM_001132925	AGCUCUAUGUUUUCGUUAAtt	UUAACGAAAACAUAGAGCUtt	110
DUSP22	XM_001132925	GCAUACAUCAUGACCGUCAtt	UGACGGUCAUGAUGUAUGCga	197
DUSP22	XM_001132925	UGACACUGGUGAUCGCAUAtt	UAUGCGAUCACCAGUGUCAcg	149
DUSP23	NM_017823	GAAGUGGACUAAAGUAUUAtt	UAAUACUUUAGUCCACUUCcc	135
DUSP23	NM_017823	GGAAGUGGACUAAAGUAUUtt	AAUACUUUAGUCCACUUCCct	232
DUSP23	NM_017823	GCCCACUACCAGUUCCUGUtt	ACAGGAACUGGUAGUGGGCgg	356
DUSP26	NM_024025	CUCCCUCACCAAAACAAAAtt	UUUUGUUUUGGUGAGGGAGag	69
DUSP26	NM_024025	GGGAUACGGCAAGCAUGAAtt	UUCAUGCUUGCCGUAUCCCtg	88
DUSP26	NM_024025	UCCGCUACCUGGGUGUUGAtt	UCAACACCCAGGUAGCGGAtg	143
DUSP28	NM_001033575	CGGAUUAGAUGUUGCUAUAtt	UAUAGCAACAUCUAAUCCGga	306
DUSP28	NM_001033575	GCAAGAGGGUUGUAUAUUUtt	AAAUAUACAACCCUCUUGCct	195

DUSP28	NM_001033575	GGUUGUAUAUUUUCCCGUUtt	AACGGGAAAAUAUACAACCct	197
DUSP3	NM_004090	GAACCCUCCUAAAUCCAUAtt	UAUGGAUUUAGGAGGGUUCtc	157
DUSP3	NM_004090	AGGACUCCGGCAUCACAUAtt	UAUGUGAUGCCGGAGUCCUtg	185
DUSP3	NM_004090	GGUCCUUCAUGCACGUCAAtt	UUGACGUGCAUGAAGGACCtg	145
DUSP4	NM_001394	GAAGGACACUAUCAGUACAtt	UGUACUGAUAGUGUCCUUCaa	119
DUSP4	NM_001394	GCAGUUCGUCUUCAGCUUUtt	AAAGCUGAAGACGAACUGCga	213
DUSP4	NM_001394	CCACUUUGAAGGACACUAUtt	AUAGUGUCCUUCAAAGUGGtt	61
DUSP5	NM_004419	GAGUGUUGCGUGGAUGUAAtt	UUACAUCCACGCAACACUCag	84
DUSP5	NM_004419	UGCAGACACUGAGCCCUGAtt	UCAGGGCUCAGUGUCUGCAaa	70
DUSP5	NM_004419	CGGCUGACAUUAGCUCCCAtt	UGGGAGCUAAUGUCAGCCGtg	204
DUSP6	NM_001946	CGGACACUAUUAUCACUAAtt	UUAGUGAUAAUAGUGUCCGta	272
DUSP6	NM_001946	GAACUGUGGUGUCUUGGUAtt	UACCAAGACACCACAGUUCtt	130
DUSP6	NM_001946	AGCUCAAUCUGUCGAUGAAtt	UUCAUCGACAGAUUGAGCUtc	85
DUSP7	NM_001947	AGAUGAACCUGUCACUCAAtt	UUGAGUGACAGGUUCAUCUtc	63
DUSP7	NM_001947	ACAAGUUUCAAACAGAGUAtt	UACUCUGUUUGAAACUUGUtg	229
DUSP7	NM_001947	AGUCCAACAUCUCGCCCAAtt	UUGGGCGAGAUGUUGGACUtt	274
DUSP8	NM_004420	CAACGACAACUACUGUGAAtt	UUCACAGUAGUUGUCGUUGat	105
DUSP8	NM_004420	CCAUCGAGUUCAUCGAUAAtt	UUAUCGAUGAACUCGAUGGac	276
DUSP8	NM_004420	CGACAACUACUGUGAAAAAtt	UUUUUCACAGUAGUUGUCGtt	131
DUSP9	NM_001395	GACUUUCACUACAAGCAGAtt	UCUGCUUGUAGUGAAAGUCac	153
DUSP9	NM_001395	CAAACUUCUUCGAGAAGAAtt	UUCUUCUCGAAGAAGUUUGgg	115
DUSP9	NM_001395	CUCUCUCAACGAUGCCUAUtt	AUAGGCAUCGUUGAGAGAGag	157
DUT	NM_001948	GCUCAUUUGCGAACGGAUUtt	AAUCCGUUCGCAAAUGAGCtg	102
DUT	NM_001948	CCUUCUGGGUGUUAUGGAAtt	UUCCAUAACACCCAGAAGGga	272
DUT	NM_001948	AGAUUAUAGAGGAAAUGUUtt	AACAUUUCCUCUAUAAUCUtc	73
ENOPH1	NM_021204	GCAACGCAGGAUUAACAGAtt	UCUGUUAAUCCUGCGUUGCct	137
ENOPH1	NM_021204	GGAAUGAAGGUGUACAUCUtt	AGAUGUACACCUUCAUUCCgg	161
ENOPH1	NM_021204	GAGAGUGAAAGUUACCGAAtt	UUCGGUAACUUUCACUCUCta	233
ENTPD1	NM_001776	GGCAUUUACCUGACUGAUUtt	AAUCAGUCAGGUAAAUGCCta	144
ENTPD1	NM_001776	GGUUCAGCAUAGUCCCAUAtt	UAUGGGACUAUGCUGAACCac	230
ENTPD1	NM_001776	GCUGGAUUACUAUCAACUAtt	UAGUUGAUAGUAAUCCAGCca	183
ENTPD2	NM_203468	GGAUGUGCCCAAAGAGAGAtt	UCUCUCUUUGGGCACAUCCtg	142
ENTPD2	NM_203468	CGUCCAUGUUUAUCUACAAtt	UUGUAGAUAAACAUGGACGtg	378
ENTPD2	NM_203468	GCUUCAGGAUGUGCCCAAAtt	UUUGGGCACAUCCUGAAGCgc	170
ENTPD3	NM_001248	CCCUCGCUUUCUUCACAGUtt	ACUGUGAAGAAAGCGAGGGtg	173
ENTPD3	NM_001248	GCAGAAUUCUCCUACCAAAtt	UUUGGUAGGAGAAUUCUGCag	177

ENTPD3	NM_001248	CCAGGACUGAAGUAUGGUAtt	UACCAUACUUCAGUCCUGGag	186
ENTPD4	NM_004901	GUAUGGGCGACUAACCAGAtt	UCUGGUUAGUCGCCCAUACtt	140
ENTPD4	NM_004901	GACCCGCUUUCUACCGUUAtt	UAACGGUAGAAAGCGGGUCct	231
ENTPD4	NM_004901	GAAGCUACAGACACCAAUAtt	UAUUGGUGUCUGUAGCUUCaa	137
ENTPD5	NM_001249	GCAUCAUGGAUGGAUCCGAtt	UCGGAUCCAUCCAUGAUGCta	156
ENTPD5	NM_001249	CACCUUUCCUGGUACCAAAtt	UUUGGUACCAGGAAAGGUGac	196
ENTPD5	NM_001249	GUCUUACAGCUCACAAAGAtt	UCUUUGUGAGCUGUAAGACtg	160
ENTPD6	NM_001247	AGAGGUCCUUCAAAACAGAtt	UCUGUUUUGAAGGACCUCUga	114
ENTPD6	NM_001247	GGAAAAUUGACAAUGUUGAtt	UCAACAUUGUCAAUUUUCCga	207
ENTPD6	NM_001247	CUGCGGAUGUUUAACAGGAtt	UCCUGUUAAACAUCCGCAGtg	94
ENTPD7	NM_020354	GGCAUAUAUCAAUCGCCUAtt	UAGGCGAUUGAUAUAUGCCat	140
ENTPD7	NM_020354	CGAGAUAGGCAAUACGAAAtt	UUUCGUAUUGCCUAUCUCGtg	126
ENTPD7	NM_020354	GGACAGUAGGGAUACUGGAtt	UCCAGUAUCCCUACUGUCCtt	184
ENTPD8	NM_198585	CGGGAACUUUUCAACUUCUtt	AGAAGUUGAAAAGUUCCCGga	104
ENTPD8	NM_198585	CCAGCCUCGAGUUCCGAAAtt	UUUCGGAACUCGAGGCUGGgc	429
ENTPD8	NM_198585	CUUGGAGGGUGAGACUGCAtt	UGCAGUCUCACCCUCCAAGtg	114
EPM2A	NM_001018041	GACACUAUGAUUAAACUAUtt	AUAGUUUAAUCAUAGUGUCtg	123
EPM2A	NM_001018041	CAUGUAACCAUCAAACUGAtt	UCAGUUUGAUGGUUACAUGtt	175
EPM2A	NM_001018041	GACCGUUGCUGUACUUACAtt	UGUAAGUACAGCAACGGUCat	135
FBP1	NM_000507	CCAACGACCUGGUUAUGAAtt	UUCAUAACCAGGUCGUUGGag	199
FBP1	NM_000507	GAAAGAAAUCAACUGAUGAtt	UCAUCAGUUGAUUUCUUUCta	198
FBP1	NM_000507	CUACCAACGUGACAGGUGAtt	UCACCUGUCACGUUGGUAGaa	146
FBP2	NM_003837	ACAUGGUCCAAUCCUCCUAtt	UAGGAGGAUUGGACCAUGUtg	104
FBP2	NM_003837	GCUCCUGUAUGAAUGCAAUtt	AUUGCAUUCAUACAGGAGCcg	247
FBP2	NM_003837	CCACUGAAUAUGUGCAGAAtt	UUCUGCACAUAUUCAGUGGtg	147
FHIT	NM_002012	GCCGAUUUGUUUCAGACGAtt	UCGUCUGAAACAAAUCGGCca	116
FHIT	NM_002012	CGCUCUUGUGAAUAGGAAAtt	UUUCCUAUUCACAAGAGCGaa	405
FHIT	NM_002012	UAGUGUUUCUCAAAACAGAtt	UCUGUUUUGAGAAACACUAca	179
FLJ16165	NM_001004318	GAGAGAUGCCCUACACUAAtt	UUAGUGUAGGGCAUCUCUCgg	93
FLJ16165	NM_001004318	GGCCGUGGAUCAUCACUAUtt	AUAGUGAUGAUCCACGGCCgg	156
FLJ16165	NM_001004318	GGCGGAAGCUCUACAUACAtt	UGUAUGUAGAGCUUCCGCCgg	302
FLJ32658	NM_144688	GGAUGGAGUGAAAAAGAGAtt	UCUCUUUUUCACUCCAUCCcg	114
FLJ32658	NM_144688	GGAACAGAAUCGCAGCCUUtt	AAGGCUGCGAUUCUGUUCCtc	75
FLJ32658	NM_144688	GCAGCUGAGUCAGACCUAUtt	AUAGGUCUGACUCAGCUGCtc	91
FLJ40125	XM_001132018	CAUCCAGACCAAAAAGAAAtt	UUUCUUUUUGGUCUGGAUGtt	209
FLJ40125	XM_001132018	CCAACAUCCAGACCAAAAAtt	UUUUUGGUCUGGAUGUUGGtg	319

FLJ40125	XM_001132018	GUAGAGUUUUGAUUAUUAAtt	UUAAUAAUCAAAACUCUACtt	159
G6PC	NM_000151	GCAGGUGUAUACUACGUGAtt	UCACGUAGUAUACACCUGCtg	267
G6PC	NM_000151	GAAUCUACCUUGCUGCUCAtt	UGAGCAGCAAGGUAGAUUCgt	221
G6PC	NM_000151	GGUGGGUUUUGGAUACUGAtt	UCAGUAUCCAAAACCCACCag	92
G6PC2	NM_021176	GAUUUACCCAAAUCACUCAtt	UGAGUGAUUUGGGUAAAUCtg	91
G6PC2	NM_021176	GCUUACUACACUUUUCUAAtt	UUAGAAAAGUGUAGUAAGCtc	162
G6PC2	NM_021176	UGUUUUCAAUUUAAUCAGAtt	UCUGAUUAAAUUGAAAACAaa	216
G6PC3	NM_138387	GGCUUGUCGCGAAUCUUCAtt	UGAAGAUUCGCGACAAGCCaa	181
G6PC3	NM_138387	GGAUCAGCCUCAUCACCGAtt	UCGGUGAUGAGGCUGAUCCag	170
G6PC3	NM_138387	CCAAGAUCCUCUUUCUGUUtt	AACAGAAAGAGGAUCUUGGga	188
HDDC3	NM_198527	CACUGACAUUGUGGUGUUAtt	UAACACCACAAUGUCAGUGat	101
HDDC3	NM_198527	AACACGUUCUGGAAAAUAAtt	UUAUUUUCCAGAACGUGUUag	182
HDDC3	NM_198527	CCAUCCUUCCCAGAUAUUAtt	UAAUAUCUGGGAAGGAUGGag	472
HDHD1A	NM_012080	AGAAAUAUGUAAUCGCUAUtt	AUAGCGAUUACAUAUUUCUtg	149
HDHD1A	NM_012080	CGCUAUGACAAGAAAUACAtt	UGUAUUUCUUGUCAUAGCGat	500
HDHD1A	NM_012080	GCCGAGAUCUGACAACAAAtt	UUUGUUGUCAGAUCUCGGCtc	136
HDHD2	NM_032124	GAAUCAAGCAUUCCGGUUAtt	UAACCGGAAUGCUUGAUUCag	214
HDHD2	NM_032124	GGAGCACCUCUGAUAGCAAtt	UUGCUAUCAGAGGUGCUCCat	226
HDHD2	NM_032124	GCUAGUUGAUGAUCGGGCAtt	UGCCCGAUCAUCAACUAGCag	207
HINT1	NM_005340	CUGCUGAUCUGGGCCUGAAtt	UUCAGGCCCAGAUCAGCAGca	186
HINT1	NM_005340	GGUGGUGAAUGAAGGUUCAtt	UGAACCUUCAUUCACCACCat	319
HINT1	NM_005340	GUGAUACCCAAGAAACAUAtt	UAUGUUUCUUGGGUAUCACca	168
HINT2	NM_032593	GCAGCUUCUAGGACACCUAtt	UAGGUGUCCUAGAAGCUGCtg	298
HINT2	NM_032593	ACCGACUUGUGAUCAACGAtt	UCGUUGAUCACAAGUCGGUat	190
HINT2	NM_032593	UCAUCUGCACAUUCAUGUAtt	UACAUGAAUGUGCAGAUGAta	230
ILKAP	NM_030768	GCAUCAAAACUUAAUCAGAtt	UCUGAUUAAGUUUUGAUGCaa	138
ILKAP	NM_030768	CAUGCAGCCUUAAGCCUCAtt	UGAGGCUUAAGGCUGCAUGtt	129
ILKAP	NM_030768	GAAGAAGCCGUGAACUUCAtt	UGAAGUUCACGGCUUCUUCtg	142
IMPA1	NM_005536	GCAGAUGCAUAUUAUGAAAtt	UUUCAUAAUAUGCAUCUGCtc	206
IMPA1	NM_005536	GGUUCUUUCUAAUAUGGAAtt	UUCCAUAUUAGAAAGAACCat	295
IMPA1	NM_005536	GGAUAGCUAAAGAAAUUCAtt	UGAAUUUCUUUAGCUAUCCtt	90
IMPA2	NM_014214	GAUUUGCUGUUCGACAAGAtt	UCUUGUCGAACAGCAAAUCca	126
IMPA2	NM_014214	CUGCAGAUCUUGUGACAGAtt	UCUGUCACAAGAUCUGCAGct	237
IMPA2	NM_014214	CGACGGCACCUGCAAUUUUtt	AAAAUUGCAGGUGCCGUCGat	257
IMPAD1	NM_017813	GAAAGUACGUCACUACUAUtt	AUAGUAGUGACGUACUUUCga	311
IMPAD1	NM_017813	GGAUGUGCCUGAUAAGAGUtt	ACUCUUAUCAGGCACAUCCaa	160

IMPAD1	NM_017813	CAGAGGAUCUUCGAAAGUAtt	UACUUUCGAAGAUCCUCUGta	107
INPP1	NM_002194	GAUUCAACUUAUCAGUAUAtt	UAUACUGAUAAGUUGAAUCta	230
INPP1	NM_002194	GAAUCCAAUGAGUUUACUAtt	UAGUAAACUCAUUGGAUUCtt	143
INPP1	NM_002194	CAGCAGAGCUUCUUAGCAAtt	UUGCUAAGAAGCUCUGCUGtt	220
INPP5A	XM_001133189	GAUCUACUCGGAUACCUUAtt	UAAGGUAUCCGAGUAGAUCtc	245
INPP5A	XM_001133189	GCCUUUGACUUGGUGAAUAtt	UAUUCACCAAGUCAAAGGCac	193
INPP5A	XM_001133189	CAGCGAUUCGAGAAGGUUUtt	AAACCUUCUCGAAUCGCUGat	192
INPP5B	NM_005540	GCGGGAACAUACAAUGUAAtt	UUACAUUGUAUGUUCCCGCaa	220
INPP5B	NM_005540	GUUGAUUACCUGUACCGAAtt	UUCGGUACAGGUAAUCAACca	336
INPP5B	NM_005540	GAGUCUUACUGUAAGCAGUtt	ACUGCUUACAGUAAGACUCtt	111
INPP5D	NM_001017915	GCGACAUCAUGACGAGUGAtt	UCACUCGUCAUGAUGUCGCtg	152
INPP5D	NM_001017915	GGAGCGUGAUGAAUCCAGUtt	ACUGGAUUCAUCACGCUCCgt	167
INPP5D	NM_001017915	GACUGACUCGGGACAAAUAtt	UAUUUGUCCCGAGUCAGUCtc	184
INPP5E	NM_019892	GGACUACACCAGGACUGUAtt	UACAGUCCUGGUGUAGUCCag	101
INPP5E	NM_019892	GGAUUUCGAAGGAGAUUCAtt	UGAAUCUCCUUCGAAAUCCgt	81
INPP5E	NM_019892	GACCGCGUCUUGUACAGAAtt	UUCUGUACAAGACGCGGUCcg	141
INPP5F	NM_014937	GGAUCGGAUUUACCCAUAAtt	UUAUGGGUAAAUCCGAUCCca	80
INPP5F	NM_014937	GAAGGUUGAUGACCGAUUUtt	AAAUCGGUCAUCAACCUUCtg	293
INPP5F	NM_014937	GGAAUGCGCUAUAAACGAAtt	UUCGUUUAUAGCGCAUUCCtg	114
INPPL1	NM_001567	UCACAGAAGUUCACGCUGAtt	UCAGCGUGAACUUCUGUGAct	261
INPPL1	NM_001567	CAAUCACUGUGGAAUAUCAtt	UGAUAUUCCACAGUGAUUGca	358
INPPL1	NM_001567	GAAGAGCUUUGAGAAUGAUtt	AUCAUUCUCAAAGCUCUUCtt	164
ITPA	NM_033453	CGUACGCAGAGAUGCCUAAtt	UUAGGCAUCUCUGCGUACGtc	152
ITPA	NM_033453	GCUUUCAGCCUGAUGGAUAtt	UAUCCAUCAGGCUGAAAGCag	348
ITPA	NM_033453	GCCGGAUGAGAUUUCCAUAtt	UAUGGAAAUCUCAUCCGGCtc	120
KIAA0274	NM_014845	GCACGUGACUUUAUGCCUAtt	UAGGCAUAAAGUCACGUGCtg	81
KIAA0274	NM_014845	GGUUCUUAGAAGGCUAUUAtt	UAAUAGCCUUCUAAGAACCtg	186
KIAA0274	NM_014845	GGACGACCAGUGUAUGUCAtt	UGACAUACACUGGUCGUCCat	244
KIAA1274	NM_014431	UGAUUCUCUUCAACGCGUAtt	UACGCGUUGAAGAGAAUCAgg	286
KIAA1274	NM_014431	GAAGUUAGAAGGUAUCCGAtt	UCGGAUACCUUCUAACUUCtt	265
KIAA1274	NM_014431	GAGCGAUACUUCUACCUGAtt	UCAGGUAGAAGUAUCGCUCca	160
LHPP	NM_022126	GGAAAAACCUGUGCUCAUAtt	UAUGAGCACAGGUUUUUCCag	156
LHPP	NM_022126	CCAGCGGUGUGGAAUGAGAtt	UCUCAUUCCACACCGCUGGgc	222
LHPP	NM_022126	CCUUCUCCUGAGUUUUUCAtt	UGAAAAACUCAGGAGAAGGct	270
LOC283871	NM_001042371	GAAAGUGACUGCGUGUCUAtt	UAGACACGCAGUCACUUUCct	236
LOC283871	NM_001042371	CUGCGUGUCUAAGAAGAAAtt	UUUCUUCUUAGACACGCAGtc	65

LOC283871	NM_001042371	GUGACUGCGUGUCUAAGAAtt	UUCUUAGACACGCAGUCACtt	171
LOC389217	XM_371701	GGACCGUCCAUAUACUUGAtt	UCAAGUAUAUGGACGGUCCtg	141
LOC389217	XM_371701	AAUCCAUUCUUUAGAUCUUtt	AAGAUCUAAAGAAUGGAUUtg	142
LOC389217	XM_371701	GGUUGUGACAAGCAAGAAAtt	UUUCUUGCUUGUCACAACCca	112
LOC441511	XM_937130	GGAAAACUAUAGGCUGGAAtt	UUCCAGCCUAUAGUUUUCCaa	163
LOC441511	XM_937130	GAACUUCUAUGGGCAUCGAtt	UCGAUGCCCAUAGAAGUUCta	164
LOC441511	XM_937130	CUGUUUCAUUAACUAUGUAtt	UACAUAGUUAAUGAAACAGat	133
LOC441868	XM_497647	UCACUACAAGAACAUCCUUtt	AAGGAUGUUCUUGUAGUGAtt	101
LOC441868	XM_497647	CAGCUGAAGUGCACCACCAtt	UGGUGGUGCACUUCAGCUGcg	190
LOC441868	XM_497647	CAAGCCCAAGAAUCACUACtt	GUAGUGAUUCUUGGGCUUGtt	132
LOC63928	NM_022097	CAAACUUCACUAUGCAUUUtt	AAAUGCAUAGUGAAGUUUGtt	139
LOC63928	NM_022097	GGCUCAUUUUCGCCCUGUAtt	UACAGGGCGAAAAUGAGCCaa	147
LOC63928	NM_022097	GGAGUUCACCAAGUCCUUAtt	UAAGGACUUGGUGAACUCCac	188
LPPR2	NM_022737	CUGCGUUGUGCAUAACUUUtt	AAAGUUAUGCACAACGCAGgt	125
LPPR2	NM_022737	CCAGGGAUUCUUCUGCUAUtt	AUAGCAGAAGAAUCCCUGGgt	760
LPPR2	NM_022737	GGAUUCUUCUGCUAUGACAtt	UGUCAUAGCAGAAGAAUCCct	111
LPPR4	NM_014839	GGCUAACACGGAUAACUCAtt	UGAGUUAUCCGUGUUAGCCcg	174
LPPR4	NM_014839	GACCUGCAAUUACGAUUAUtt	AUAAUCGUAAUUGCAGGUCca	282
LPPR4	NM_014839	GAAGCAACAUUGAUAGCAAtt	UUGCUAUCAAUGUUGCUUCtc	140
LRGUK	NM_144648	CACCAGUGGUCUACACUAUtt	AUAGUGUAGACCACUGGUGag	57
LRGUK	NM_144648	GAAUAUUGGUUCUUCGUAAtt	UUACGAAGAACCAAUAUUCag	219
LRGUK	NM_144648	GGAUCUUUCAGCGAAUAAAtt	UUUAUUCGCUGAAAGAUCCaa	243
MFN1	NM_033540	GAAAUUUGAUCUCAGUUAUtt	AUAACUGAGAUCAAAUUUCtt	143
MFN1	NM_033540	CGCAAACUCUGAAUCAACAtt	UGUUGAUUCAGAGUUUGCGac	74
MFN1	NM_033540	GGCGAUUACUGCAAUCUUUtt	AAAGAUUGCAGUAAUCGCCtt	106
MFN2	NM_014874	CAGCUGGAUUGACAAGUUUtt	AAACUUGUCAAUCCAGCUGtc	107
MFN2	NM_014874	GUAUUGAUGUCACCACAGAtt	UCUGUGGUGACAUCAAUACca	129
MFN2	NM_014874	GACUAUAAGCUGCGAAUUAtt	UAAUUCGCAGCUUAUAGUCtt	282
MINPP1	NM_004897	CAUAGAUGAUGCAAAGGUAtt	UACCUUUGCAUCAUCUAUGtc	126
MINPP1	NM_004897	GUUCCGAAGUGGUCUCAUUtt	AAUGAGACCACUUCGGAACtt	188
MINPP1	NM_004897	GGAUAUGGGUAUACUAUUAtt	UAAUAGUAUACCCAUAUCCtc	96
MTM1	NM_000252	CAUUGAAGGGUUCGAAAUAtt	UAUUUCGAACCCUUCAAUGct	156
MTM1	NM_000252	GGUACUUUCUUAUUCAACUtt	AGUUGAAUAAGAAAGUACCaa	70
MTM1	NM_000252	CUGUGAAUCUGCUCGAGAAtt	UUCUCGAGCAGAUUCACAGtt	204
MTMR1	NM_003828	GUUACUACAGGACCAUUAAtt	UUAAUGGUCCUGUAGUAACtg	172
MTMR1	NM_003828	GCUUAUAGCUGCUACAAUUtt	AAUUGUAGCAGCUAUAAGCtg	472

MTMR1	NM_003828	GGAUCACCUUUAUAGCUGUtt	ACAGCUAUAAAGGUGAUCCaa	264
MTMR10	NM_017762	CGAUAAGAUCAAUUCGGAAtt	UUCCGAAUUGAUCUUAUCGtc	219
MTMR10	NM_017762	GAAUUGUCAGAUUUCGCUUtt	AAGCGAAAUCUGACAAUUCtg	332
MTMR10	NM_017762	GGAUCCCUAUUUUAGGACAtt	UGUCCUAAAAUAGGGAUCCag	187
MTMR11	NM_181873	GGGACUGGGAUUUACGUUAtt	UAACGUAAAUCCCAGUCCCag	143
MTMR11	NM_181873	CUUCAGGAGUUAUUACGGAtt	UCCGUAAUAACUCCUGAAGat	284
MTMR11	NM_181873	GCAAUGCACAGAUACUACAtt	UGUAGUAUCUGUGCAUUGCta	126
MTMR12	NM_001040446	CCUAAACUGCUUAAACGAUtt	AUCGUUUAAGCAGUUUAGGag	163
MTMR12	NM_001040446	CAGCUAUUUCUGAUAGAUAtt	UAUCUAUCAGAAAUAGCUGtt	214
MTMR12	NM_001040446	CCCAAGAACCAUACCGUAAtt	UUACGGUAUGGUUCUUGGGat	141
MTMR14	NM_001077525	GUUUCAGCGUGAUUCCAAAtt	UUUGGAAUCACGCUGAAACag	115
MTMR14	NM_001077525	GGAAUAUAAAGAUCGGGAUtt	AUCCCGAUCUUUAUAUUCCtt	292
MTMR14	NM_001077525	GCUGGUACACUGUAUCUCAtt	UGAGAUACAGUGUACCAGCag	267
MTMR2	NM_016156	GGACAUCGAUUUCAACUAAtt	UUAGUUGAAAUCGAUGUCCaa	176
MTMR2	NM_016156	CUCUGACUGUCACGAAUUAtt	UAAUUCGUGACAGUCAGAGtt	264
MTMR2	NM_016156	GAGAGAAUCAUUACGAAAAtt	UUUUCGUAAUGAUUCUCUCat	188
MTMR3	NM_153050	GCUGGACCCUUAUUACCGAtt	UCGGUAAUAAGGGUCCAGCaa	159
MTMR3	NM_153050	GGAACAUUCCUGUGCAACAtt	UGUUGCACAGGAAUGUUCCaa	200
MTMR3	NM_153050	GGAUGGGUUUUGAUAUGAAtt	UUCAUAUCAAAACCCAUCCtc	133
MTMR4	NM_004687	CGUACAUCCAGUGACCCUAtt	UAGGGUCACUGGAUGUACGag	121
MTMR4	NM_004687	GGAUUUUGGGCACAAGUUUtt	AAACUUGUGCCCAAAAUCCag	151
MTMR4	NM_004687	GCAACACCUCUGAUCCUGAtt	UCAGGAUCAGAGGUGUUGCtc	109
MTMR6	NM_004685	GACCAAAAGAAGUACUUAAtt	UUAAGUACUUCUUUUGGUCtt	99
MTMR6	NM_004685	GACAACUAUUCCAAUAUUAtt	UAAUAUUGGAAUAGUUGUCtt	222
MTMR6	NM_004685	GGAUAUUACACCACCAUAUtt	AUAUGGUGGUGUAAUAUCCag	101
MTMR7	NM_004686	GGCAGUUAAUGGAACAAUUtt	AAUUGUUCCAUUAACUGCCaa	129
MTMR7	NM_004686	GUCAGUUACAGAUUACCUAtt	UAGGUAAUCUGUAACUGACtg	158
MTMR7	NM_004686	GCACUAAGGUGAAGAGUAAtt	UUACUCUUCACCUUAGUGCaa	40
MTMR8	NM_017677	GGCAAUUAAUGGAACAGUUtt	AACUGUUCCAUUAAUUGCCag	112
MTMR8	NM_017677	CCAUAACUGUUGUGAGAUUtt	AAUCUCACAACAGUUAUGGtg	150
MTMR8	NM_017677	CCCAGUUCCUAGACUGUAUtt	AUACAGUCUAGGAACUGGGtg	155
MTMR9	NM_015458	GGCUAAGCUAUGUCAAUAAtt	UUAUUGACAUAGCUUAGCCtc	88
MTMR9	NM_015458	GCGAAUUCAUAAGUCCAUUtt	AAUGGACUUAUGAAUUCGCct	404
MTMR9	NM_015458	GAAUAGAUCCUCUAAGUAUtt	AUACUUAGAGGAUCUAUUCca	201
NAP1L1	NM_004537	CUUUUUACGUGAGCGUAUAtt	UAUACGCUCACGUAAAAAGtg	162
NAP1L1	NM_004537	GGAACACGAUGAACCUAUUtt	AAUAGGUUCAUCGUGUUCCtg	128

NAP1L1	NM_004537	GCCUCUAUUUGAUAAGCGAtt	UCGCUUAUCAAAUAGAGGCtg	262
NAP1L2	NM_021963	CACUCACUCCUUUGAUUAAtt	UUAAUCAAAGGAGUGAGUGta	156
NAP1L2	NM_021963	CAAUCCGAACUGUAACUGAtt	UCAGUUACAGUUCGGAUUGtt	242
NAP1L2	NM_021963	CAAUUUACGUACUUACAUAtt	UAUGUAAGUACGUAAAUUGtg	180
NAP1L3	NM_004538	GGAACAAAUUUCGUGGAUAtt	UAUCCACGAAAUUUGUUCCca	67
NAP1L3	NM_004538	GGCAGAUUUUACAUGAUAAtt	UUAUCAUGUAAAAUCUGCCca	163
NAP1L3	NM_004538	GUAGAUACCCUGUUCUUAAtt	UUAAGAACAGGGUAUCUACct	321
NAP1L4	NM_005969	CAACAGAUGCGGAAUCGGAtt	UCCGAUUCCGCAUCUGUUGgt	139
NAP1L4	NM_005969	GGUGUACUAUUGACUGGAAtt	UUCCAGUCAAUAGUACACCcg	148
NAP1L4	NM_005969	CAUCGAAACUUUACCUAAAtt	UUUAGGUAAAGUUUCGAUGta	176
NAP1L5	NM_153757	GAAUAUCGAUGGACUUAAAtt	UUUAAGUCCAUCGAUAUUCtg	156
NAP1L5	NM_153757	AAAGUAUAAUGACAUCUAUtt	AUAGAUGUCAUUAUACUUUtt	210
NAP1L5	NM_153757	CUGCAGAAGCGAUGCGAUAtt	UAUCGCAUCGCUUCUGCAGct	310
NT5C2	NM_012229	GUAUCGUCGAGAAGCCUAUtt	AUAGGCUUCUCGACGAUACtt	77
NT5C2	NM_012229	GCGUCAGGUGGAUACUAAAtt	UUUAGUAUCCACCUGACGCag	128
NT5C2	NM_012229	GCGUUAUGCUGACCUCUAUtt	AUAGAGGUCAGCAUAACGCat	404
NT5E	NM_002526	GUAUCCAUGUGCAUUUUAAtt	UUAAAAUGCACAUGGAUACgt	278
NT5E	NM_002526	GAGACAGAGUAGUCAAAUUtt	AAUUUGACUACUCUGUCUCca	233
NT5E	NM_002526	CGAGUUUGAUGAAAGAGGAtt	UCCUCUUUCAUCAAACUCGat	266
NUDT10	NM_153183	CAAAAAUUCUUACUCCUAUtt	AUAGGAGUAAGAAUUUUUGct	137
NUDT10	NM_153183	GCAUAUAAGAUAUUGCUUAtt	UAAGCAAUAUCUUAUAUGCta	265
NUDT10	NM_153183	GCCCAAACAUUGAACCUAAtt	UUAGGUUCAAUGUUUGGGCaa	154
NUDT11	NM_018159	CAACUCUAGUGUUUUUGUAtt	UACAAAAACACUAGAGUUGtt	181
NUDT11	NM_018159	UGAUGUCUUUGAGAGCAAAtt	UUUGCUCUCAAAGACAUCAta	113
NUDT11	NM_018159	CUUCUUACAUGUCAUCUUAtt	UAAGAUGACAUGUAAGAAGtg	159
NUDT14	NM_177533	GCGUGACCGUUCUCUUAUUtt	AAUAAGAGAACGGUCACGCtg	246
NUDT14	NM_177533	GCAUGACAGCGUGACCGUUtt	AACGGUCACGCUGUCAUGCgt	70
NUDT14	NM_177533	UGACCGUUCUCUUAUUCAAtt	UUGAAUAAGAGAACGGUCAcg	204
NUDT3	NM_006703	GGAAUGGUUUAAAAUAGAAtt	UUCUAUUUUAAACCAUUCCct	106
NUDT3	NM_006703	GGAAGAUUCAGUUAACAUUtt	AAUGUUAACUGAAUCUUCCca	174
NUDT3	NM_006703	CAGGCAUCAUAUUUUGAAAtt	UUUCAAAAUAUGAUGCCUGca	211
NUDT4	NM_199040	CCAAGACCGAAAGCACAGAtt	UCUGUGCUUUCGGUCUUGGtt	133
NUDT4	NM_199040	CCAGUGUCAUAAACCUGUAtt	UACAGGUUUAUGACACUGGag	127
NUDT4	NM_199040	CAGUCACUGAAAUAUUAGAtt	UCUAAUAUUUCAGUGACUGtt	298
NUDT5	NM_014142	GUCAUUUCUUUACCCAAGAtt	UCUUGGGUAAAGAAAUGACtt	155
NUDT5	NM_014142	GGUCUAUUCCUACGCUCUAtt	UAGAGCGUAGGAAUAGACCct	199

NUDT5	NM_014142	GGAUCCUACUGGUAAAACUtt	AGUUUUACCAGUAGGAUCCat	104
NUDT6	NM_007083	GAAGAUAUUGGAGACACAGtt	CUGUGUCUCCAAUAUCUUCtt	128
NUDT6	NM_007083	GAAAAAUACUGGUUGUACAtt	UGUACAACCAGUAUUUUUCta	171
NUDT6	NM_007083	AGAUCGAAAUAAAUUGAAAtt	UUUCAAUUUAUUUCGAUCUtg	134
NUDT8	NM_181843	GCUCCACUCCAGGACUGAAtt	UUCAGUCCUGGAGUGGAGCtg	96
NUDT8	NM_181843	GCCUGUGUAUGAUCCGCAAtt	UUGCGGAUCAUACACAGGCcg	166
NUDT8	NM_181843	CCACUUCCGCUACACACUAtt	UAGUGUGUAGCGGAAGUGGcc	102
OCRL	NM_001587	GAUUACUUCUUGACUAUCAtt	UGAUAGUCAAGAAGUAAUCtt	131
OCRL	NM_001587	GAAAGGAUCAGUGUCGAUAtt	UAUCGACACUGAUCCUUUCtg	274
OCRL	NM_001587	CUCCCGCAGUUGAACAUCAtt	UGAUGUUCAACUGCGGGAGgg	155
PAP2D	NM_001010861	GAGUAGCAGAAUAUCGAAAtt	UUUCGAUAUUCUGCUACUCtg	211
PAP2D	NM_001010861	GGACUGUUUGCUACAGAUAtt	UAUCUGUAGCAAACAGUCCaa	266
PAP2D	NM_001010861	GUGUCUAUGCAGCUAUGUAtt	UACAUAGCUGCAUAGACACtg	109
PDP2	NM_020786	GAUUUUCACUUGCAACUCAtt	UGAGUUGCAAGUGAAAAUCat	55
PDP2	NM_020786	UCACUGUGGUGUAUUUUAAtt	UUAAAAUACACCACAGUGAca	286
PDP2	NM_020786	CAAGAUUCUUGACCUUGAAtt	UUCAAGGUCAAGAAUCUUGtg	142
PDXP	NM_020315	CUCUAUCCCUGAGUACUUAtt	UAAGUACUCAGGGAUAGAGgg	111
PDXP	NM_020315	CAGUUUAGGUUCCUAAGUAtt	UACUUAGGAACCUAAACUGgt	100
PDXP	NM_020315	GCACGCUUAUGGUGGGUGAtt	UCACCCACCAUAAGCGUGCgt	332
PFKFB1	NM_002625	CCAUCUACCUUUGCCGACAtt	UGUCGGCAAAGGUAGAUGGag	373
PFKFB1	NM_002625	GCUCGAGGCAAGACCUAUAtt	UAUAGGUCUUGCCUCGAGCtg	70
PFKFB1	NM_002625	GGAUAGGAACACCAACUAAtt	UUAGUUGGUGUUCCUAUCCag	129
PFKFB2	NM_001018053	GCCUCGCACCAUUUACCUUtt	AAGGUAAAUGGUGCGAGGCtg	163
PFKFB2	NM_001018053	CGACUCUGAUCGUUAUGAUtt	AUCAUAACGAUCAGAGUCGgg	181
PFKFB2	NM_001018053	CCAAGAAACUAACACGCUAtt	UAGCGUGUUAGUUUCUUGGac	381
PFKFB3	NM_004566	CCAUCUACCUGAACGUGGAtt	UCCACGUUCAGGUAGAUGGat	184
PFKFB3	NM_004566	GAGGAUCAGUUGCUAUGAAtt	UUCAUAGCAACUGAUCCUCtt	161
PFKFB3	NM_004566	CCAAUAUCAUGGAAGUUAAtt	UUAACUUCCAUGAUAUUGGag	70
PFKFB4	NM_004567	UGACCUACGAGGAAAUUCAtt	UGAAUUUCCUCGUAGGUCAtt	30
PFKFB4	NM_004567	GUAUAUUACCUCAUGAACAtt	UGUUCAUGAGGUAAUAUACga	79
PFKFB4	NM_004567	GGAGAGCGACCAUCUUUAAtt	UUAAAGAUGGUCGCUCUCCgt	175
PHACTR1	NM_030948	GAAAGAUCCUCAUCCGCUUtt	AAGCGGAUGAGGAUCUUUCtt	173
PHACTR1	NM_030948	GCUGCAGAAUGAUAGACGAtt	UCGUCUAUCAUUCUGCAGCcc	293
PHACTR1	NM_030948	CCAUCCGCAGGAGAAGUAAtt	UUACUUCUCCUGCGGAUGGgt	178
PHACTR2	NM_014721	GGCCUAUCUUGUACACCGAtt	UCGGUGUACAAGAUAGGCCcg	193
PHACTR2	NM_014721	GAAGGAUCCUGCGAUUUAAtt	UUAAAUCGCAGGAUCCUUCga	119

PHACTR2	NM_014721	GGAGGGAUACUCUUGCUAUtt	AUAGCAAGAGUAUCCCUCCgg	286
PHACTR3	NM_183246	CAUAAAUUCUGAACUCAAAtt	UUUGAGUUCAGAAUUUAUGat	88
PHACTR3	NM_183246	GGAAUGAUCAGACAGAGCAtt	UGCUCUGUCUGAUCAUUCCtt	112
PHACTR3	NM_183246	GGCAGCAAUUCGUAAAGAAtt	UUCUUUACGAAUUGCUGCCtt	167
PHACTR4	NM_001048183	CAGAAGUUUUAGAACGGAAtt	UUCCGUUCUAAAACUUCUGaa	166
PHACTR4	NM_001048183	GGUCUCUUCCCAUCACUAUtt	AUAGUGAUGGGAAGAGACCtg	114
PHACTR4	NM_001048183	CGCAAUAUAUUGCAACCUAtt	UAGGUUGCAAUAUAUUGCGtt	129
PIB5PA	NM_001002837	CUUCCGCAUUGAGAGCUAUtt	AUAGCUCUCAAUGCGGAAGtt	96
PIB5PA	NM_001002837	GGUACCAACAAAUACGAUAtt	UAUCGUAUUUGUUGGUACCca	183
PIB5PA	NM_001002837	AGUUUGAUGUGGGUACCAAtt	UUGGUACCCACAUCAAACUtg	87
PNKP	NM_007254	GGAGGAUCUUGUACCCAGAtt	UCUGGGUACAAGAUCCUCCag	144
PNKP	NM_007254	GGAAGUCCACCUUUCUCAAtt	UUGAGAAAGGUGGACUUCCcg	137
PNKP	NM_007254	CGAAGAAGCGUAUGCGGAAtt	UUCCGCAUACGCUUCUUCGgc	189
PPA1	NM_021129	GUUUAGAAGGUAUAAGGUUtt	AACCUUAUACCUUCUAAACca	97
PPA1	NM_021129	GGAAUCAGUUGCAUGAAUAtt	UAUUCAUGCAACUGAUUCCtt	142
PPA1	NM_021129	CCUGCACAGUACCAACAGAtt	UCUGUUGGUACUGUGCAGGca	111
PPA2	NM_176867	GCAGAUAUCUGAUAGCCCUtt	AGGGCUAUCAGAUAUCUGCac	152
PPA2	NM_176867	GAUCAUUAGUUGAAUCGGUtt	ACCGAUUCAACUAAUGAUCtt	183
PPA2	NM_176867	GCAAGAUCAUUAGUUGAAUtt	AUUCAACUAAUGAUCUUGCtt	388
PPAP2A	NM_176895	CCAGAUUGGUCAAAAAUCAtt	UGAUUUUUGACCAAUCUGGat	124
PPAP2A	NM_176895	GAUCCAGAUUGGUCAAAAAtt	UUUUUGACCAAUCUGGAUCac	138
PPAP2A	NM_176895	CUGCAGCGAUGGUUACAUUtt	AAUGUAACCAUCGCUGCAGtt	235
PPAP2B	NM_177414	ACUGAAAACUGGUGAGACAtt	UGUCUCACCAGUUUUCAGUgg	114
PPAP2B	NM_177414	GGCUACAUUCAGAACUACAtt	UGUAGUUCUGAAUGUAGCCtt	177
PPAP2B	NM_177414	CACCGAGGGUUUUACUGCAtt	UGCAGUAAAACCCUCGGUGgt	107
PPAP2C	NM_177526	ACACCCGCGUGUCUGAUUAtt	UAAUCAGACACGCGGGUGUag	167
PPAP2C	NM_177526	GCUCGGACUUCAACAACUAtt	UAGUUGUUGAAGUCCGAGCga	149
PPAP2C	NM_177526	CCGCGUGUCUGAUUACAAAtt	UUUGUAAUCAGACACGCGGgt	142
PPAPDC1A	NM_001030059	GGUGCAAUCAGAUAACAUAtt	UAUGUUAUCUGAUUGCACCaa	173
PPAPDC1A	NM_001030059	CCUUUGGUGCAAUCAGAUAtt	UAUCUGAUUGCACCAAAGGat	163
PPAPDC1A	NM_001030059	GCACAAACACUAUUAAAUUtt	AAUUUAAUAGUGUUUGUGCag	214
PPAPDC1B	NM_032483	AGCUUAUUAUACUUACUUAtt	UAAGUAAGUAUAAUAAGCUtt	193
PPAPDC1B	NM_032483	CCAAGCCGAUGUUUGUUAUtt	AUAACAAACAUCGGCUUGGtg	424
PPAPDC1B	NM_032483	CAGUGGACAUUCUUCCUUUtt	AAAGGAAGAAUGUCCACUGgg	109
PPAPDC2	NM_203453	GCCCUGAUGUCGAGGUUCAtt	UGAACCUCGACAUCAGGGCgg	200
PPAPDC2	NM_203453	AGACAUAGGUUUUUCUAGUtt	ACUAGAAAAACCUAUGUCUtg	196

PPAPDC2	NM_203453	GAAAGAAACAUGAUGUUCAtt	UGAACAUCAUGUUUCUUUCtc	175
PPAPDC3	NM_032728	GCUUCUUGCUAAACCUUCUtt	AGAAGGUUUAGCAAGAAGCtt	100
PPAPDC3	NM_032728	UGACAAGCCCUAUAGAAAAtt	UUUUCUAUAGGGCUUGUCAga	254
PPAPDC3	NM_032728	GGAUGUGCAUUUCAUGUGAtt	UCACAUGAAAUGCACAUCCaa	122
PPEF1	NM_152226	CCUCAGUUCGAAUCUGGUAtt	UACCAGAUUCGAACUGAGGga	125
PPEF1	NM_152226	CGUGUAGAGAGGAACAAGAtt	UCUUGUUCCUCUCUACACGgt	223
PPEF1	NM_152226	GUACAAUCGUUGACAAUGAtt	UCAUUGUCAACGAUUGUACcg	106
PPEF2	NM_006239	CGCAAGGUAUUAACAAUCUtt	AGAUUGUUAAUACCUUGCGgt	105
PPEF2	NM_006239	GAAUAUCGACAUUACAGAUtt	AUCUGUAAUGUCGAUAUUCat	181
PPEF2	NM_006239	CUGGAAACAUUGUAUCGAAtt	UUCGAUACAAUGUUUCCAGca	254
PPM1A	NM_177951	GACUUGAAGUCACUGAUGAtt	UCAUCAGUGACUUCAAGUCtg	170
PPM1A	NM_177951	GGAUGUUAUGGGAAAUGAAtt	UUCAUUUCCCAUAACAUCCca	109
PPM1A	NM_177951	CCAAUAACCAGGAUUUUAAtt	UUAAAAUCCUGGUUAUUGGtg	119
PPM1B	NM_177969	ACAUGAGUAUUGUACUAGUtt	ACUAGUACAAUACUCAUGUta	210
PPM1B	NM_177969	AGAUAACAUGAGUAUUGUAtt	UACAAUACUCAUGUUAUCUcg	118
PPM1B	NM_001033556	GAGAUAACAUGAGUAUUGUtt	ACAAUACUCAUGUUAUCUCga	196
PPM1D	NM_003620	GGGUCUUCCUAGCACAUCAtt	UGAUGUGCUAGGAAGACCCgt	166
PPM1D	NM_003620	GGAAAGAGAACGAAUCGAAtt	UUCGAUUCGUUCUCUUUCCtt	197
PPM1D	NM_003620	GGACAUUAGAAGAGUCCAAtt	UUGGACUCUUCUAAUGUCCtt	218
PPM1E	NM_014906	GAAUCAUGGAGAGUGCAAAtt	UUUGCACUCUCCAUGAUUCtc	94
PPM1E	NM_014906	GAGUAGAUGCUGCUAUUUAtt	UAAAUAGCAGCAUCUACUCcc	188
PPM1E	NM_014906	GAGGCAAAAUAGUUGGAAAtt	UUUCCAACUAUUUUGCCUCca	205
PPM1F	NM_014634	GACCUUUCCGAAUUCAGGAtt	UCCUGAAUUCGGAAAGGUCtg	135
PPM1F	NM_014634	GGAUGAGAAGGCGCGCAUUtt	AAUGCGCGCCUUCUCAUCCtg	316
PPM1F	NM_014634	UGUGAACCGCGCCUACUUUtt	AAAGUAGGCGCGGUUCACAgg	90
PPM1G	NM_002707	CCAUGACUAUUGAAGAGCUtt	AGCUCUUCAAUAGUCAUGGta	80
PPM1G	NM_002707	AGGCUACCAUGACUAUUGAtt	UCAAUAGUCAUGGUAGCCUct	122
PPM1G	NM_002707	CUCUGACAGUGGUACAACAtt	UGUUGUACCACUGUCAGAGcc	157
PPM1H	XM_350880	CAGGGACUUUAAUAUGACAtt	UGUCAUAUUAAAGUCCCUGta	133
PPM1H	XM_350880	GAGACGACAUUUCUGUAUAtt	UAUACAGAAAUGUCGUCUCct	315
PPM1H	XM_350880	GCGGAUAUCUAAUGACCGAtt	UCGGUCAUUAGAUAUCCGCca	104
PPM1J	NM_005167	CCAAUGUCCCGGGAGUUUAtt	UAAACUCCCGGGACAUUGGaa	167
PPM1J	NM_005167	UGCUAGUCCUGGGAACAGAtt	UCUGUUCCCAGGACUAGCAca	139
PPM1J	NM_005167	GGAUGUCACUACUGACUGUtt	ACAGUCAGUAGUGACAUCCca	253
PPM1K	NM_152542	CAUUGACCAUACUCCAGAAtt	UUCUGGAGUAUGGUCAAUGgt	69
PPM1K	NM_152542	GAAUUAACUUCAUGGUGAAtt	UUCACCAUGAAGUUAAUUCca	163

PPM1K	NM_152542	GCCCUAUUGCGAGAUGGUAtt	UACCAUCUCGCAAUAGGGCta	126
PPM1L	NM_139245	ACCGAGAAAUGCUAGAAAAtt	UUUUCUAGCAUUUCUCGGUca	161
PPM1L	NM_139245	GGACUACGAGAAAGACAAAtt	UUUGUCUUUCUCGUAGUCCtg	80
PPM1L	NM_139245	GAGCAUAGUUUUACAGUCAtt	UGACUGUAAAACUAUGCUCtt	194
PPM1M	NM_144641	GGGAUGUACUGUCCAACGAtt	UCGUUGGACAGUACAUCCCag	167
PPM1M	NM_144641	AGAAGGUUUUGUUCAGGGAtt	UCCCUGAACAAAACCUUCUgt	268
PPM1M	NM_144641	GGUGCGGAGAGAUGAGAUAtt	UAUCUCAUCUCUCCGCACCaa	197
PPM2C	NM_018444	CAACGAGUUUGGGACUGUUtt	AACAGUCCCAAACUCGUUGtt	81
PPM2C	NM_018444	CAGUCACGCUGUCUAAUGAtt	UCAUUAGACAGCGUGACUGct	209
PPM2C	NM_018444	CGCAAGUUGGUGAUCCUAAtt	UUAGGAUCACCAACUUGCGcc	118
PPP1CA	NM_001008709	CAUCUAUGGUUUCUACGAUtt	AUCGUAGAAACCAUAGAUGcg	113
PPP1CA	NM_001008709	CGAGAGCAACUACCUCUUUtt	AAAGAGGUAGUUGCUCUCGgg	172
PPP1CA	NM_001008709	CGACCUUCUGCGACUAUUUtt	AAAUAGUCGCAGAAGGUCGta	80
PPP1CB	NM_002709	GAUCUUCUGUUGUCAUGGAtt	UCCAUGACAACAGAAGAUCtt	171
PPP1CB	NM_002709	GCUAUGGUCUGAUCCAGAUtt	AUCUGGAUCAGACCAUAGCaa	142
PPP1CB	NM_002709	GAGCUGAUGUAGUCAGUAAtt	UUACUGACUACAUCAGCUCca	168
PPP1CC	NM_002710	CACCAGAUCUUCAAUCUAUtt	AUAGAUUGAAGAUCUGGUGat	131
PPP1CC	NM_002710	CACAGACUGUUUUAACUGUtt	ACAGUUAAAACAGUCUGUGaa	240
PPP1CC	NM_002710	CAGCUUCAGGAGAAUGAAAtt	UUUCAUUCUCCUGAAGCUGga	57
PPP1R11	NM_021959	AGGUAGAAUGGACAAGUGAtt	UCACUUGUCCAUUCUACCUtt	116
PPP1R11	NM_021959	GAGCCUUACCAUCAAACUUtt	AAGUUUGAUGGUAAGGCUCcg	243
PPP1R11	NM_021959	CUGCUGUAUUUAUGAGAAAtt	UUUCUCAUAAAUACAGCAGca	151
PPP1R12A	NM_002480	CAUCAGCUGGUGAUCGAUAtt	UAUCGAUCACCAGCUGAUGta	128
PPP1R12A	NM_002480	CACCUACAUCACCUAUUAAtt	UUAAUAGGUGAUGUAGGUGtt	367
PPP1R12A	NM_002480	GCAGUACCUCAAAUCGUUUtt	AAACGAUUUGAGGUACUGCtt	252
PPP1R12B	NM_032104	GACCAGCCGUGUAGAAGAAtt	UUCUUCUACACGGCUGGUCag	61
PPP1R12B	NM_032104	GCAUCAAUUUCUGGACAAAtt	UUUGUCCAGAAAUUGAUGCct	198
PPP1R12B	NM_032104	GGGAGGUAGUAAUCCUACAtt	UGUAGGAUUACUACCUCCCga	115
PPP1R12C	NM_017607	GAGCAGUCGCGAGAAGAUUtt	AAUCUUCUCGCGACUGCUCag	62
PPP1R12C	NM_017607	ACAUUGAGGUGAUGAGGUUtt	AACCUCAUCACCUCAAUGUag	83
PPP1R12C	NM_017607	GGAGGACCUUCGGAACCAAtt	UUGGUUCCGAAGGUCCUCCtg	81
PPP1R14A	NM_033256	GUGGGAAACCUGUCGAGGAtt	UCCUCGACAGGUUUCCCACat	122
PPP1R14A	NM_033256	GCCCGAUGAGAUCAACAUUtt	AAUGUUGAUCUCAUCGGGCat	98
PPP1R14A	NM_033256	CCAGCUUGCUUGUGUAUAAtt	UUAUACACAAGCAAGCUGGgc	156
PPP1R14B	NM_138689	CUUUAAUAAAGCUAGGAUAtt	UAUCCUAGCUUUAUUAAAGgg	104
PPP1R14B	NM_138689	AGGACCACGCGUCUACUUUtt	AAAGUAGACGCGUGGUCCUgg	153

PPP1R14B	NM_138689	UGAUGGGACUUUUUGUGUUtt	AACACAAAAAGUCCCAUCAgt	110
PPP1R14C	NM_030949	GGAUAAGAGGCAUGAGGAAtt	UUCCUCAUGCCUCUUAUCCga	79
PPP1R14C	NM_030949	AGAGAGAGCUUCAAAAUUAtt	UAAUUUUGAAGCUCUCUCUtc	149
PPP1R14C	NM_030949	CAGGGAAAAGUGACAGUGAtt	UCACUGUCACUUUUCCCUGct	193
PPP1R14D	NM_017726	GGACAUUUGCAUACUCCUAtt	UAGGAGUAUGCAAAUGUCCct	70
PPP1R14D	NM_017726	CCCGGACUCCUCCAAGAUAtt	UAUCUUGGAGGAGUCCGGGtg	143
PPP1R14D	NM_017726	GGAGGGACAUUUGCAUACUtt	AGUAUGCAAAUGUCCCUCCtt	84
PPP1R16A	NM_032902	GCAUUGAUGAUUUCCGAGAtt	UCUCGGAAAUCAUCAAUGCag	166
PPP1R16A	NM_032902	GGCUGAAGCAUGCCCAGAAtt	UUCUGGGCAUGCUUCAGCCgc	463
PPP1R16A	NM_032902	GCAUCUAUACUCCAAGCGAtt	UCGCUUGGAGUAUAGAUGCcg	152
PPP1R16B	NM_015568	GGACCAACCUGUAUAGGAAtt	UUCCUAUACAGGUUGGUCCtg	185
PPP1R16B	NM_015568	GCCCUGAUUUGUGCAAUGAtt	UCAUUGCACAAAUCAGGGCtg	109
PPP1R16B	NM_015568	GGACAGACCAAGAGAAUAAtt	UUAUUCUCUUGGUCUGUCCtg	187
PPP1R1A	NM_006741	AGAUGAUGGUUGAACAUCAtt	UGAUGUUCAACCAUCAUCUgg	137
PPP1R1A	NM_006741	CCACAUCUCAAGUCCACUUtt	AAGUGGACUUGAGAUGUGGgt	278
PPP1R1A	NM_006741	CGGCAACGGAAGAAGAUGAtt	UCAUCUUCUUCCGUUGCCGtg	135
PPP1R1B	NM_181505	GCUGAAGUCCUGAAGGUCAtt	UGACCUUCAGGACUUCAGCct	84
PPP1R1B	NM_181505	UCAGCAAUUUGAAUGAGAAtt	UUCUCAUUCAAAUUGCUGAta	189
PPP1R1B	NM_181505	GGAGCUGGGUUAUCCAAGAtt	UCUUGGAUAACCCAGCUCCcg	199
PPP1R1C	XM_933439	GCCGUGCCUGUAUUCCAGAtt	UCUGGAAUACAGGCACGGCaa	154
PPP1R1C	XM_933439	GGUUUGCAUUCUAACAACAtt	UGUUGUUAGAAUGCAAACCtt	150
PPP1R1C	XM_933439	AGAGUCAGAUUGCACCUGAtt	UCAGGUGCAAUCUGACUCUgg	102
PPP1R2	NM_006241	CCAAGCACUCCUUACCAUAtt	UAUGGUAAGGAGUGCUUGGtt	131
PPP1R2	NM_006241	GGAAAUUAGCUGCAGCUGAtt	UCAGCUGCAGCUAAUUUCCtg	52
PPP1R2	NM_006241	CGAAGUUCAUAGACGAGAUtt	AUCUCGUCUAUGAACUUCGta	211
PPP1R3A	NM_002711	CAAGGGUAUUAUUCGAGUUtt	AACUCGAAUAAUACCCUUGat	220
PPP1R3A	NM_002711	GGAUGAUAAUGCUAAUCCAtt	UGGAUUAGCAUUAUCAUCCag	152
PPP1R3A	NM_002711	GGCUUGACAUUCUACGUUUtt	AAACGUAGAAUGUCAAGCCaa	164
PPP1R3B	NM_024607	GCAUUUGAGAAGACCGUGAtt	UCACGGUCUUCUCAAAUGCga	125
PPP1R3B	NM_024607	GGAAUUCGAUGACCCGCUAtt	UAGCGGGUCAUCGAAUUCCga	256
PPP1R3B	NM_024607	GCACUGCGAAGGUUCAGAAtt	UUCUGAACCUUCGCAGUGCct	170
PPP1R3C	NM_005398	CCACGUCCUUUGACAAGUUtt	AACUUGUCAAAGGACGUGGat	79
PPP1R3C	NM_005398	GAAAUCAUGUCUCAAUAUAtt	UAUAUUGAGACAUGAUUUCag	190
PPP1R3C	NM_005398	CACUUUCGAUUCUUGGAAAtt	UUUCCAAGAAUCGAAAGUGat	194
PPP1R3D	NM_006242	GGACAACAACGACCACCGAtt	UCGGUGGUCGUUGUUGUCCca	267
PPP1R3D	NM_006242	GUACAUGUUUGAUCAUUAUtt	AUAAUGAUCAAACAUGUACag	132

PPP1R3D	NM_006242	GAAUAUUCUCGAUUUAAUAtt	UAUUAAAUCGAGAAUAUUCct	194
PPP1R3F	NM_033215	GCUCAUCAAGGACACCGAAtt	UUCGGUGUCCUUGAUGAGCtc	206
PPP1R3F	NM_033215	CCAGCUCUUUGCACAUGAAtt	UUCAUGUGCAAAGAGCUGGtg	67
PPP1R3F	NM_033215	AGAUAGAGGUCACCAGUGAtt	UCACUGGUGACCUCUAUCUgg	76
PPP1R7	NM_002712	GGAUUUGAGGUACUGAAGAtt	UCUUCAGUACCUCAAAUCCtt	96
PPP1R7	NM_002712	GAUUGAAGGAUUUGAGGUAtt	UACCUCAAAUCCUUCAAUCtt	139
PPP1R7	NM_002712	GCAACUUACAUCAACUACAtt	UGUAGUUGAUGUAAGUUGCtt	110
PPP1R8	NM_002713	GAGACAAACUAAUUGAGAAtt	UUCUCAAUUAGUUUGUCUCct	165
PPP1R8	NM_002713	GAGAAGAAGUAUUACUUAUtt	AUAAGUAAUACUUCUUCUCat	152
PPP1R8	NM_002713	CAAGCGGAUUUCUACCCUUtt	AAGGGUAGAAAUCCGCUUGtt	126
PPP2CA	NM_002715	CCAAACUAUUGUUAUCGUUtt	AACGAUAACAAUAGUUUGGag	195
PPP2CA	NM_002715	GCUUGUAGCUCUUAAGGUUtt	AACCUUAAGAGCUACAAGCag	128
PPP2CA	NM_002715	GAACUUGACGAUACUCUAAtt	UUAGAGUAUCGUCAAGUUCca	115
PPP2CB	NM_001009552	GGAUAUUAUUCAGUGGAGAtt	UCUCCACUGAAUAAUAUCCtc	196
PPP2CB	NM_001009552	CAAUUACUGUUAUCGUUGUtt	ACAACGAUAACAGUAAUUGgg	206
PPP2CB	NM_001009552	CAAUAUUGAGAGGAAAUCAtt	UGAUUUCCUCUCAAUAUUGta	299
PPP2R1A	NM_014225	GAACAGCUGGGAACCUUCAtt	UGAAGGUUCCCAGCUGUUCtg	64
PPP2R1A	NM_014225	CUUCGACAGUACUUCCGGAtt	UCCGGAAGUACUGUCGAAGtt	152
PPP2R1A	NM_014225	GGAGUUCUUUGAUGAGAAAtt	UUUCUCAUCAAAGAACUCCac	164
PPP2R1B	NM_181699	CGAUCGCGGUUUUAAUCGAtt	UCGAUUAAAACCGCGAUCGgg	251
PPP2R1B	NM_181699	CCUAAUUACUUGCAUAGAAtt	UUCUAUGCAAGUAAUUAGGat	206
PPP2R1B	NM_181699	GACCAAUUCUAGAUACCAAtt	UUGGUAUCUAGAAUUGGUCca	111
PPP2R2A	NM_002717	GAGCUAACAGAGGUGAUUAtt	UAAUCACCUCUGUUAGCUCtt	83
PPP2R2A	NM_002717	GCAUCGCGGGAAAACAAUAtt	UAUUGUUUUCCCGCGAUGCtt	73
PPP2R2A	NM_002717	GCCUAGACUUCAAUAAGAAtt	UUCUUAUUGAAGUCUAGGCtg	99
PPP2R2B	NM_181674	CAAGCGUGAUGUGACCCUUtt	AAGGGUCACAUCACGCUUGgt	53
PPP2R2B	NM_181674	CAUCGAGACUUACCAGGUUtt	AACCUGGUAAGUCUCGAUGgg	100
PPP2R2B	NM_181674	GGGACUACUUGACCGUCAAtt	UUGACGGUCAAGUAGUCCCtg	149
PPP2R2C	NM_020416	GGAAGAUUACCGAACGAGAtt	UCUCGUUCGGUAAUCUUCCat	157
PPP2R2C	NM_020416	GGGACUACCUUACAGUCAAtt	UUGACUGUAAGGUAGUCCCgg	216
PPP2R2C	NM_020416	GAUACAACCUGAAGGAUGAtt	UCAUCCUUCAGGUUGUAUCct	109
PPP2R2D	NM_001003656	AGUGCAACGUGUUCGUCUAtt	UAGACGAACACGUUGCACUgg	220
PPP2R2D	NM_001003656	CCAUAUCCGAUGUAAAAUUtt	AAUUUUACAUCGGAUAUGGat	127
PPP2R2D	NM_001003656	CGUCUACAGCAGUAGCAAAtt	UUUGCUACUGCUGUAGACGaa	122
PPP2R3A	NM_181897	GAAAGUUGCUGAAUAACCAtt	UGGUUAUUCAGCAACUUUCtc	35
PPP2R3A	NM_181897	GAAGGAUGUUGAGAACGAUtt	AUCGUUCUCAACAUCCUUCtg	60

PPP2R3A	NM_181897	CGAUCUGUCUCGAUACAAUtt	AUUGUAUCGAGACAGAUCGgc	81
PPP2R3B	NM_013239	CCUCGACCACGAGCAGAAAtt	UUUCUGCUCGUGGUCGAGGta	78
PPP2R3B	NM_013239	GGUCAAGCCGAGGACUGAAtt	UUCAGUCCUCGGCUUGACCag	48
PPP2R3B	NM_013239	CGUUUGUACGGAAUGAUAAtt	UUAUCAUUCCGUACAAACGca	206
PPP2R3C	NM_017917	CGAUGAUCAAUUACGAAAAtt	UUUUCGUAAUUGAUCAUCGct	127
PPP2R3C	NM_017917	CCACCAUUCUAAUCGAUUUtt	AAAUCGAUUAGAAUGGUGGtt	206
PPP2R3C	NM_017917	GGUUUUUGCUGGACAAACAtt	UGUUUGUCCAGCAAAAACCat	101
PPP2R4	NM_178001	GGAUUCAUCCUUACCCUCAtt	UGAGGGUAAGGAUGAAUCCga	64
PPP2R4	NM_178001	AGAACUUCAUCAUUCCAAAtt	UUUGGAAUGAUGAAGUUCUga	227
PPP2R4	NM_178001	GAGUGUAUCCUGUUUAUUAtt	UAAUAAACAGGAUACACUCca	136
PPP2R5A	NM_006243	CGAUGACCUUACUAGCUCAtt	UGAGCUAGUAAGGUCAUCGaa	195
PPP2R5A	NM_006243	GUACUUCUGGAAUAACGAAtt	UUCGUUAUUCCAGAAGUACaa	427
PPP2R5A	NM_006243	GCCUAGCAUUGCAAAACGAtt	UCGUUUUGCAAUGCUAGGCtg	77
PPP2R5B	NM_006244	CAAACCAUCGUAUCACUGAtt	UCAGUGAUACGAUGGUUUGgt	187
PPP2R5B	NM_006244	GAACAAUGAGUAUAUCCUAtt	UAGGAUAUACUCAUUGUUCca	175
PPP2R5B	NM_006244	CGCAAACAGUGCAACCACAtt	UGUGGUUGCACUGUUUGCGga	85
PPP2R5C	NM_178588	GGCUUGAGAGCUUACAUCAtt	UGAUGUAAGCUCUCAAGCCta	52
PPP2R5C	NM_178588	GUAAUGUUCUUAAACGAAUtt	AUUCGUUUAAGAACAUUACtt	137
PPP2R5C	NM_178588	GGAUUUGCCUUACCACUAAtt	UUAGUGGUAAGGCAAAUCCat	85
PPP2R5D	NM_006245	UCCAUGGACUGAUCUAUAAtt	UUAUAGAUCAGUCCAUGGAtt	212
PPP2R5D	NM_006245	GCCGUGAUGUUGUCACUGAtt	UCAGUGACAACAUCACGGCta	224
PPP2R5D	NM_006245	UCAUGAGCCUGAUAAGUGAtt	UCACUUAUCAGGCUCAUGAtg	170
PPP2R5E	NM_006246	CACGCUAUCUGAUCUUAAAtt	UUUAAGAUCAGAUAGCGUGtc	197
PPP2R5E	NM_006246	GGAGCUAUUUGACAGCGAAtt	UUCGCUGUCAAAUAGCUCCag	436
PPP2R5E	NM_006246	CAGUUUAGGUCUCAAGGCAtt	UGCCUUGAGACCUAAACUGtg	135
PPP3CA	NM_000944	CAUUGAGAAUAAUAACAGAtt	UCUGUUAUUAUUCUCAAUGca	154
PPP3CA	NM_000944	CAUCAAUUCUUCGACAGGAtt	UCCUGUCGAAGAAUUGAUGca	288
PPP3CA	NM_000944	CACUUUAGAUGAUAUCAGAtt	UCUGAUAUCAUCUAAAGUGtt	155
PPP3CB	NM_021132	GGGUUUGGAUAGGAUCAAUtt	AUUGAUCCUAUCCAAACCCtt	132
PPP3CB	NM_021132	GAGAUUAGAUAGAUUCAAAtt	UUUGAAUCUAUCUAAUCUCct	107
PPP3CB	NM_021132	GGUGAAAGAAGGUCGAGUAtt	UACUCGACCUUCUUUCACCaa	162
PPP3CC	NM_005605	GGACAAUUCUUUGACCUAAtt	UUAGGUCAAAGAAUUGUCCat	50
PPP3CC	NM_005605	GCACUACAGUUCGUAAGGAtt	UCCUUACGAACUGUAGUGCtt	305
PPP3CC	NM_005605	CUAGAUGUCUAUAACAAUAtt	UAUUGUUAUAGACAUCUAGgt	136
PPP3R1	NM_000945	GUACAGCGAGUAAUAGAUAtt	UAUCUAUUACUCGCUGUACta	200
PPP3R1	NM_000945	GGCUAGGAAAGAGAUUUAAtt	UUAAAUCUCUUUCCUAGCCtt	170

PPP3R1	NM_000945	CAGCAAAUUGUAGACAAAAtt	UUUUGUCUACAAUUUGCUGta	106
PPP3R2	NM_147180	GGUUUGCGUUCAGCAUUUAtt	UAAAUGCUGAACGCAAACCtc	65
PPP3R2	NM_147180	CACCGACGGUGAUGGAGAAtt	UUCUCCAUCACCGUCGGUGtc	130
PPP3R2	NM_147180	GCAUUUACGACAUGGAUAAtt	UUAUCCAUGUCGUAAAUGCtg	184
PPP4C	NM_002720	UCAAGGCCCUGUGCGCUAAtt	UUAGCGCACAGGGCCUUGAct	184
PPP4C	NM_002720	GGCACUUAAGGUUCGCUAUtt	AUAGCGAACCUUAAGUGCCag	436
PPP4C	NM_002720	GACAAUCGACCGAAAGCAAtt	UUGCUUUCGGUCGAUUGUCcg	121
PPP4R1	NM_001042388	GCCGCAUCUGCUAACCUUAtt	UAAGGUUAGCAGAUGCGGCat	256
PPP4R1	NM_001042388	GGAUAGGUGUUCUUAAACAtt	UGUUUAAGAACACCUAUCCtg	97
PPP4R1	NM_001042388	GGAGCUCAUUGAACGAUUUtt	AAAUCGUUCAAUGAGCUCCtg	161
PPP5C	NM_006247	AGAACAACCUGGACUAUAUtt	AUAUAGUCCAGGUUGUUCUct	68
PPP5C	NM_006247	CAGAUGUACGAGCUCUUUAtt	UAAAGAGCUCGUACAUCUGgg	179
PPP5C	NM_006247	ACAAGAUCGUGAAGCAGAAtt	UUCUGCUUCACGAUCUUGUtg	70
PPP6C	NM_002721	CCUAAUUACUGCUAUCGUUtt	AACGAUAGCAGUAAUUAGGag	131
PPP6C	NM_002721	CCAUCGAACGGAAUCAGGAtt	UCCUGAUUCCGUUCGAUGGtt	221
PPP6C	NM_002721	GCCUGAUCGUAUUACACUUtt	AAGUGUAAUACGAUCAGGCca	87
PPTC7	NM_139283	GGACGGUUCGUACCUAGUAtt	UACUAGGUACGAACCGUCCtt	215
PPTC7	NM_139283	GAAGGACGGUUCGUACCUAtt	UAGGUACGAACCGUCCUUCtt	139
PPTC7	NM_139283	GUUUGCAUGUGACAAUGGAtt	UCCAUUGUCACAUGCAAACtg	209
PRG2	XM_001129992	UCAACUCGGUCAUCUCGGAtt	UCCGAGAUGACCGAGUUGAag	221
PRG2	NM_024888	GGCUCAUGAUGGCCGAGAAtt	UUCUCGGCCAUCAUGAGCCac	146
PRG2	NM_024888	ACGACUCGGUUUAUCAGCAtt	UGCUGAUAAACCGAGUCGUgg	109
PSPH	NM_004577	GGAGCGAAAUGUUCAGGUUtt	AACCUGAACAUUUCGCUCCtg	106
PSPH	NM_004577	CUUUAACGGUGAAUAUGCAtt	UGCAUAUUCACCGUUAAAGta	132
PSPH	NM_004577	GGCAACAAGUCAAGGAUAAtt	UUAUCCUUGACUUGUUGCCtg	133
PTEN	NM_000314	GCAUACGAUUUUAAGCGGAtt	UCCGCUUAAAAUCGUAUGCag	137
PTEN	NM_000314	CACCGCAUAUUAAAACGUAtt	UACGUUUUAAUAUGCGGUGcc	162
PTEN	NM_000314	GGUUUUCGAGUCCUAAUUAtt	UAAUUAGGACUCGAAAACCtt	135
PTP4A1	NM_003463	GAUUGUUGAUGACUGGUUAtt	UAACCAGUCAUCAACAAUCtg	116
PTP4A1	NM_003463	CAACCAAUGCGACCUUAAAtt	UUUAAGGUCGCAUUGGUUGga	182
PTP4A1	NM_003463	GUAUGGAGUUACCACAAUAtt	UAUUGUGGUAACUCCAUACtt	137
PTP4A2	NM_080391	GAACAUGCGUUUUCUGAUAtt	UAUCAGAAAACGCAUGUUCtc	191
PTP4A2	NM_080391	CGAUUACGCUUCAGAGAUAtt	UAUCUCUGAAGCGUAAUCGca	136
PTP4A2	NM_080391	UGCGUUUUCUGAUAACUCAtt	UGAGUUAUCAGAAAACGCAtg	236
PTP4A3	NM_007079	AGAGGCUGCGGUUCAAAGAtt	UCUUUGAACCGCAGCCUCUgt	117
PTP4A3	NM_007079	GAAGUGACCUAUGACAAAAtt	UUUUGUCAUAGGUCACUUCac	147

PTP4A3	NM_007079	CCUUCAUUGAGGACCUGAAtt	UUCAGGUCCUCAAUGAAGGtg	109
PTPDC1	NM_152422	GCAUUACAGUCUGAAUUGAtt	UCAAUUCAGACUGUAAUGCag	68
PTPDC1	NM_152422	CAACGAGAAUGACUGCUGAtt	UCAGCAGUCAUUCUCGUUGca	290
PTPDC1	NM_152422	CACCUUACCUCAAUAUCUAtt	UAGAUAUUGAGGUAAGGUGac	244
PTPN1	NM_002827	GUCAGUCCCUUUGACCAUAtt	UAUGGUCAAAGGGACUGACgt	135
PTPN1	NM_002827	GGAGAAAGGUUCGUUAAAAtt	UUUUAACGAACCUUUCUCCat	147
PTPN1	NM_002827	GGAUUAAACUACAUCAAGAtt	UCUUGAUGUAGUUUAAUCCga	218
PTPN11	NM_002834	CGCUCAUGACUAUACGCUAtt	UAGCGUAUAGUCAUGAGCGgc	59
PTPN11	NM_002834	CAAUGACGGCAAGUCUAAAtt	UUUAGACUUGCCGUCAUUGct	350
PTPN11	NM_002834	CCAUGUUAUGAUUCGCUGUtt	ACAGCGAAUCAUAACAUGGgt	262
PTPN12	NM_002835	GGCAAUUCCUCAGAUAUCAtt	UGAUAUCUGAGGAAUUGCCtt	274
PTPN12	NM_002835	GCAAUUCCUCAGAUAUCAAtt	UUGAUAUCUGAGGAAUUGCct	183
PTPN12	NM_002835	CGAGUUAAAUUGACAUUAAtt	UUAAUGUCAAUUUAACUCGgc	162
PTPN13	NM_080684	CUAGUUCGAUGGAUAAGUAtt	UACUUAUCCAUCGAACUAGag	90
PTPN13	NM_080684	GGAUGUUAUUUACGCUCGAtt	UCGAGCGUAAAUAACAUCCtc	355
PTPN13	NM_080684	GUAUGGAGAUUAUCAACCAtt	UGGUUGAUAAUCUCCAUACtc	176
PTPN14	NM_005401	CGAGUAGAGCUGAUACCAAtt	UUGGUAUCAGCUCUACUCGat	134
PTPN14	NM_005401	CCACGAAGUUUCGAACGGAtt	UCCGUUCGAAACUUCGUGGtg	98
PTPN14	NM_005401	GAAGGGCGAUUACGAUGUAtt	UACAUCGUAAUCGCCCUUCaa	213
PTPN18	NM_014369	GGAAGAACCGCUACAAAGAtt	UCUUUGUAGCGGUUCUUCCtc	129
PTPN18	NM_014369	CAAUGACUGUAGCAUUCAAtt	UUGAAUGCUACAGUCAUUGgg	387
PTPN18	NM_014369	ACAAAGACGUGCUGCCUUAtt	UAAGGCAGCACGUCUUUGUag	71
PTPN2	NM_080423	GGAGAUUCUAGUAUACAGAtt	UCUGUAUACUAGAAUCUCCct	141
PTPN2	NM_080423	GUACAGGACUUUCCUCUAAtt	UUAGAGGAAAGUCCUGUACat	125
PTPN2	NM_080423	CCUUUGAUCAUUCACCAAAtt	UUUGGUGAAUGAUCAAAGGca	175
PTPN20A	NM_001042390	GGAUUACAGAGCCCAAUAAtt	UUAUUGGGCUCUGUAAUCCct	202
PTPN20A	NM_001042390	CCCUUUAGCUAACUAAUAAtt	UUAUUAGUUAGCUAAAGGGca	236
PTPN20A	NM_001042390	GAUGUUACAUAAAACGAUUtt	AAUCGUUUUAUGUAACAUCtg	165
PTPN20B	NM_001042365	CACCCUAACACUUAACAUAtt	UAUGUUAAGUGUUAGGGUGgg	200
PTPN20B	NM_001042365	GCAUGGUUCAAACGAAGGAtt	UCCUUCGUUUGAACCAUGCca	177
PTPN20B	NM_001042365	AGAUGUUACAUAAAACGAUtt	AUCGUUUUAUGUAACAUCUgt	136
PTPN21	NM_007039	GCAGUUGCACUAUAAUGGAtt	UCCAUUAUAGUGCAACUGCgg	179
PTPN21	NM_007039	CAACGAUUAGAACAAGGAAtt	UUCCUUGUUCUAAUCGUUGtt	78
PTPN21	NM_007039	GGAUAAAUAUGCAUUGGAAtt	UUCCAAUGCAUAUUUAUCCag	194
PTPN22	NM_012411	GGAUGUACGUUGUUACCAAtt	UUGGUAACAACGUACAUCCca	79
PTPN22	NM_012411	GUUCAAUAGUGAAACUCGAtt	UCGAGUUUCACUAUUGAACtt	251

PTPN22	NM_012411	CUAUGAUUAUAGCCGGGUAtt	UACCCGGCUAUAAUCAUAGgg	184
PTPN23	NM_015466	AGUUUGUCCUGAAGAAUUAtt	UAAUUCUUCAGGACAAACUtc	134
PTPN23	NM_015466	GACCAAAAGUGGAACUCCAtt	UGGAGUUCCACUUUUGGUCca	154
PTPN23	NM_015466	GGAAGAAACUUGUGCAGAUtt	AUCUGCACAAGUUUCUUCCag	133
PTPN3	NM_002829	CCAGGCUAUCUUUCCGAUAtt	UAUCGGAAAGAUAGCCUGGat	127
PTPN3	NM_002829	CCUACAUCACGGAAACGGAtt	UCCGUUUCCGUGAUGUAGGtc	163
PTPN3	NM_002829	CGAUGACUCCUCCGACUUUtt	AAAGUCGGAGGAGUCAUCGgg	128
PTPN5	NM_006906	GAACCGGUACAAAACCAUAtt	UAUGGUUUUGUACCGGUUCtt	164
PTPN5	NM_006906	UGACCUCACCAGACCCUGAtt	UCAGGGUCUGGUGAGGUCAga	62
PTPN5	NM_006906	CGUCAUGAGCCUCUACGAAtt	UUCGUAGAGGCUCAUGACGtg	126
PTPN6	NM_002831	CCCAUAUUCGGAUCCAGAAtt	UUCUGGAUCCGAAUAUGGGtc	197
PTPN6	NM_002831	GAACCGCUACAAGAACAUUtt	AAUGUUCUUGUAGCGGUUCtt	571
PTPN6	NM_002831	ACCUCUCCCUGACCCUGUAtt	UACAGGGUCAGGGAGAGGUgg	271
PTPN7	NM_080588	GGACGGAGAUUACAUCAAUtt	AUUGAUGUAAUCUCCGUCCtc	199
PTPN7	NM_080588	GAACCCAUCUGCUCUGUGAtt	UCACAGAGCAGAUGGGUUCta	205
PTPN7	NM_080588	GGCUAUGACGGGAAGGAGAtt	UCUCCUUCCCGUCAUAGCCtc	139
PTPN9	NM_002833	GACUGGUGUUUAUCUAUGAtt	UCAUAGAUAAACACCAGUCca	147
PTPN9	NM_002833	GGAGAAUACCUAUCGUGAUtt	AUCACGAUAGGUAUUCUCCaa	382
PTPN9	NM_002833	GAAUCUAUGAGGAAUAUGAtt	UCAUAUUCCUCAUAGAUUCct	166
PTPRA	NM_080841	CUAUCGUGAUGCUAACAGAtt	UCUGUUAGCAUCACGAUAGag	114
PTPRA	NM_080841	GCACCAAUUCUAUAGGCAUtt	AUGCCUAUAGAAUUGGUGCtt	310
PTPRA	NM_080841	CCAUCGUCAUGGUUACCAAtt	UUGGUAACCAUGACGAUGGtg	73
PTPRC	NM_002838	GUAUUUGUGACAGGGCAAAtt	UUUGCCCUGUCACAAAUACtt	79
PTPRC	NM_002838	AAGUAUUUGUGACAGGGCAtt	UGCCCUGUCACAAAUACUUct	241
PTPRC	NM_002838	CAGAAGUAUUUGUGACAGGtt	CCUGUCACAAAUACUUCUGtg	167
PTPRD	NM_001040712	GAAUAGAUACGCGAAUGUAtt	UACAUUCGCGUAUCUAUUCtt	209
PTPRD	NM_001040712	GCCUAUCCGUGGAGUAGAAtt	UUCUACUCCACGGAUAGGCtg	239
PTPRD	NM_001040712	GCACGAAUGUUGAGUUCGAtt	UCGAACUCAACAUUCGUGCct	329
PTPRE	NM_130435	GAGUGAUCCUUUCCAUGAAtt	UUCAUGGAAAGGAUCACUCgg	109
PTPRE	NM_130435	GCGUGGUUUUGGUCGACUAtt	UAGUCGACCAAAACCACGCag	297
PTPRE	NM_130435	CAUCCUUCAUAGACGGCUAtt	UAGCCGUCUAUGAAGGAUGcg	170
PTPRF	NM_130440	CCGUUACUGUUGCUGCCUAtt	UAGGCAGCAACAGUAACGGag	127
PTPRF	NM_130440	GACACCCGCUUUACCCUUAtt	UAAGGGUAAAGCGGGUGUCtg	125
PTPRF	NM_130440	GGAGUUCCUGAGUACCCAAtt	UUGGGUACUCAGGAACUCCat	373
PTPRG	NM_002841	GCACCUAUAUUGUAAUAGAtt	UCUAUUACAAUAUAGGUGCct	129
PTPRG	NM_002841	CCGUCACCCUUAUCAGCAAtt	UUGCUGAUAAGGGUGACGGta	217

PTPRG	NM_002841	GCCUUUACCUGUUCCGAGUtt	ACUCGGAACAGGUAAAGGCta	205
PTPRH	NM_002842	GGACUACACCUACUGGGUAtt	UACCCAGUAGGUGUAGUCCtg	147
PTPRH	NM_002842	CACUCUCAGUUGUACGUAUtt	AUACGUACAACUGAGAGUGgg	165
PTPRH	NM_002842	GACUGAGGCUCAGUACGUAtt	UACGUACUGAGCCUCAGUCtg	103
PTPRJ	NM_002843	CAAGGACCUUUACCGAACAtt	UGUUCGGUAAAGGUCCUUGtg	211
PTPRJ	NM_002843	CACUGAGUAUAGAACGGAAtt	UUCCGUUCUAUACUCAGUGcc	336
PTPRJ	NM_002843	GCACGUAUGACAAAGCGAUtt	AUCGCUUUGUCAUACGUGCtt	149
PTPRK	NM_002844	CAGCUAUAGCAGUAUAAGAtt	UCUUAUACUGCUAUAGCUGat	152
PTPRK	NM_002844	CCAGUAGCCCAGACUAAGAtt	UCUUAGUCUGGGCUACUGGta	100
PTPRK	NM_002844	GCCUUAAGAUCUCGGCGUAtt	UACGCCGAGAUCUUAAGGCtt	225
PTPRM	NM_002845	GCAUCGAUUUUCACUAUUUtt	AAAUAGUGAAAAUCGAUGCag	98
PTPRM	NM_002845	GCCUAAUUCCUAUUACCCAtt	UGGGUAAUAGGAAUUAGGCac	237
PTPRM	NM_002845	GGAAUAUCAUUGCAUACGAtt	UCGUAUGCAAUGAUAUUCCcg	111
PTPRN	NM_002846	UCAUCGACAUGGUCCUGAAtt	UUCAGGACCAUGUCGAUGAgg	117
PTPRN	NM_002846	GCAUAAAACUGAAGGUGGAtt	UCCACCUUCAGUUUUAUGCgg	355
PTPRN	NM_002846	ACAUCGUCACUGAUCAGAAtt	UUCUGAUCAGUGACGAUGUag	75
PTPRN2	NM_130843	GACUUCCGCAGAAAAGUAAtt	UUACUUUUCUGCGGAAGUCca	133
PTPRN2	NM_130843	GGUCGAAAAGCAAACUCAAtt	UUGAGUUUGCUUUUCGACCcg	162
PTPRN2	NM_130843	GCAGUGACCUUCAAAGUGAtt	UCACUUUGAAGGUCACUGCtg	62
PTPRO	NM_002848	GGAAGAACCUAUAGCCUAUtt	AUAGGCUAUAGGUUCUUCCgt	129
PTPRO	NM_002848	GAAUCGAUGUAAAAACCGUtt	ACGGUUUUUACAUCGAUUCag	280
PTPRO	NM_002848	CAUUCGGGAUCAUGAGUUUtt	AAACUCAUGAUCCCGAAUGtg	318
PTPRR	NM_130846	CACCUACAUUAAUGCUAAUtt	AUUAGCAUUAAUGUAGGUGct	99
PTPRR	NM_130846	CAUUCGAAACCUUGUCUUAtt	UAAGACAAGGUUUCGAAUGgt	275
PTPRR	NM_130846	GGGUCCAACGUAUCUCUUAtt	UAAGAGAUACGUUGGACCCtc	158
PTPRS	NM_130853	GCAAUGGACGCAUCAAACAtt	UGUUUGAUGCGUCCAUUGCtg	94
PTPRS	NM_130853	GGAUCAAGUGUGAUCAGUAtt	UACUGAUCACACUUGAUCCgt	125
PTPRS	NM_130853	CCUAUUACGUCAUCGAAUAtt	UAUUCGAUGACGUAAUAGGac	208
PTPRT	NM_007050	GCCUCGAUUCUCUACACUAtt	UAGUGUAGAGAAUCGAGGCtg	124
PTPRT	NM_007050	GCUUAUUCCUACUCCUAUUtt	AAUAGGAGUAGGAAUAAGCat	89
PTPRT	NM_007050	CCGCAAUAAGAAUCGAUAUtt	AUAUCGAUUCUUAUUGCGGtt	235
PTPRU	NM_005704	GCAUUGAUCCUCAGAGUAAtt	UUACUCUGAGGAUCAAUGCgg	91
PTPRU	NM_005704	GCAUCGUCAUGAUCACCAAtt	UUGGUGAUCAUGACGAUGCtg	222
PTPRU	NM_005704	GGCUCCUACUUGAUGGUCAtt	UGACCAUCAAGUAGGAGCCgt	63
PTPRZ1	NM_002851	GGAUGAUAUGGGACCAUAAtt	UUAUGGUCCCAUAUCAUCCtc	104
PTPRZ1	NM_002851	GCUUAUACAUUCCGAUGAAtt	UUCAUCGGAAUGUAUAAGCtt	126

PTPRZ1	NM_002851	GCGACCAACUGAUUGUCGAtt	UCGACAAUCAGUUGGUCGCtg	232
RNGTT	NM_003800	GCAGUGUAUAGAACGAGAAtt	UUCUCGUUCUAUACACUGCag	131
RNGTT	NM_003800	GAUUGAUAGAGACAAUUCAtt	UGAAUUGUCUCUAUCAAUCat	250
RNGTT	NM_003800	GGAACUUUUUCGUCGGUAUtt	AUACCGACGAAAAAGUUCCtt	207
RP11-35N6.1	NM_207299	CAAGCACAAUCAAGACGAAtt	UUCGUCUUGAUUGUGCUUGta	94
RP11-35N6.1	NM_207299	GGGUCUCUGAGUAUCGGAAtt	UUCCGAUACUCAGAGACCCgg	313
RP11-35N6.1	NM_207299	GGAUCUCCUUCCAAACCCAtt	UGGGUUUGGAAGGAGAUCCtt	96
RWDD2	NM_033411	GGAAUGACUAUCACAUGAAtt	UUCAUGUGAUAGUCAUUCCtt	62
RWDD2	NM_033411	GAUCUACAGUCACCAUAUAtt	UAUAUGGUGACUGUAGAUCca	146
RWDD2	NM_033411	GGCUCAUGGUGACUAUGGAtt	UCCAUAGUCACCAUGAGCCtc	181
SACM1L	NM_014016	GGAACUCAAUGAUACGAUAtt	UAUCGUAUCAUUGAGUUCCag	175
SACM1L	NM_014016	GAUCAGCGGUUUGUAUGGAtt	UCCAUACAAACCGCUGAUCtg	282
SACM1L	NM_014016	GCAAGAUGAAUUAAGUUAUtt	AUAACUUAAUUCAUCUUGCat	103
SAPS1	NM_014931	GCAAGGUCGUCAACCGCAAtt	UUGCGGUUGACGACCUUGCac	107
SAPS1	NM_014931	GAGCAGCUCUUAAGCAACAtt	UGUUGCUUAAGAGCUGCUCaa	96
SAPS1	NM_014931	GCAUCCUCAUCAACCGCAAtt	UUGCGGUUGAUGAGGAUGCcc	145
SAPS2	NM_014678	CAAUCUCAUUGCAAGAAAAtt	UUUUCUUGCAAUGAGAUUGcc	168
SAPS2	NM_014678	GACUUGUUCUUUAAGUACAtt	UGUACUUAAAGAACAAGUCca	354
SAPS2	NM_014678	ACAUCUUGCAGGAGUGUAAtt	UUACACUCCUGCAAGAUGUca	232
SAPS3	NM_018312	GGUUACAUGGGACACCUAAtt	UUAGGUGUCCCAUGUAACCat	135
SAPS3	NM_018312	GAUGAAUCCUUGCUAAUGAtt	UCAUUAGCAAGGAUUCAUCtt	196
SAPS3	NM_018312	GAUUAUCAGAUGCAACAAAtt	UUUGUUGCAUCUGAUAAUCag	86
SBF1	NM_002972	CUAAGACUGUGGACGAGAAtt	UUCUCGUCCACAGUCUUAGgg	136
SBF1	NM_002972	ACACGGAGGUGUUCAGGAAtt	UUCCUGAACACCUCCGUGUgg	499
SBF1	NM_002972	GCACUGCUGUUUCCUCUCAtt	UGAGAGGAAACAGCAGUGCca	177
SBF2	NM_030962	GAUGAUGAAUUGUACUCUAtt	UAGAGUACAAUUCAUCAUCct	117
SBF2	NM_030962	GCACUAUUAAAAUUCCCGAtt	UCGGGAAUUUUAAUAGUGCct	436
SBF2	NM_030962	GGACUGGGAUGAUACACCUtt	AGGUGUAUCAUCCCAGUCCtt	284
SET	NM_003011	GAGUCAAACGCAGAAUAAAtt	UUUAUUCUGCGUUUGACUCga	74
SET	NM_003011	UGGAAAGGAUUUGACGAAAtt	UUUCGUCAAAUCCUUUCCAga	194
SET	NM_003011	CGAAGUCCACCGAAAUCAAtt	UUGAUUUCGGUGGACUUCGaa	152
SGPP1	NM_030791	GUGUGAUGAUAUUCGAAAAtt	UUUUCGAAUAUCAUCACACgg	184
SGPP1	NM_030791	GGAUGGUAUUUGUACUAAUtt	AUUAGUACAAAUACCAUCCct	199
SGPP1	NM_030791	GCUGGAUUCCUAUAUACCAtt	UGGUAUAUAGGAAUCCAGCaa	217
SGPP2	NM_152386	GAAUAUUGACCCUUAUUUAtt	UAAAUAAGGGUCAAUAUUCca	145
SGPP2	NM_152386	CGCUGAAUCUCUCCCUGUUtt	AACAGGGAGAGAUUCAGCGgg	272

SGPP2	NM_152386	GUAUUAUACUCAUGGUUCAtt	UGAACCAUGAGUAUAAUACtt	150
SKIP	NM_130766	CCUCAAUCUUGACAUAUAUtt	AUAUAUGUCAAGAUUGAGGtt	123
SKIP	NM_130766	CAAUAUCCCUACCACUGAAtt	UUCAGUGGUAGGGAUAUUGct	170
SKIP	NM_130766	GAACCUCAAUCUUGACAUAtt	UAUGUCAAGAUUGAGGUUCcg	123
SSH1	NM_018984	CCUCCACAGUCAUAGCCUAtt	UAGGCUAUGACUGUGGAGGcc	192
SSH1	NM_018984	GGCUUAUUUGCAUAUCAUAtt	UAUGAUAUGCAAAUAAGCCag	276
SSH1	NM_018984	GCAUGGAGGAUGAUGCUAUtt	AUAGCAUCAUCCUCCAUGCcg	168
SSH2	NM_033389	GGACUUGAAUUGACUAGUUtt	AACUAGUCAAUUCAAGUCCtg	49
SSH2	NM_033389	GUAUCAUAACAUUCGGGUAtt	UACCCGAAUGUUAUGAUACtc	320
SSH2	NM_033389	GGAGCGACACGCUAAUUCAtt	UGAAUUAGCGUGUCGCUCCag	178
SSH3	NM_017857	AGACUGAACUCCGAACAGAtt	UCUGUUCGGAGUUCAGUCUct	72
SSH3	NM_017857	CCACGAGUCUUCACAUGAAtt	UUCAUGUGAAGACUCGUGGga	50
SSH3	NM_017857	GCACCUCAGAGACCAGUGAtt	UCACUGGUCUCUGAGGUGCtc	145
STYXL1	NM_016086	CCCUUCGAGUGAAGAAGAAtt	UUCUUCUUCACUCGAAGGGca	87
STYXL1	NM_016086	GUCACUUCAUUGAAAUUCAtt	UGAAUUUCAAUGAAGUGACac	107
STYXL1	NM_016086	ACAAAACUCUCCAGAUUAAtt	UUAAUCUGGAGAGUUUUGUgg	130
SYNJ1	NM_203446	GACUGGUUAUUACGUCUUAtt	UAAGACGUAAUAACCAGUCat	97
SYNJ1	NM_203446	GCCUAAGAAAAUUCGAGUAtt	UACUCGAAUUUUCUUAGGCtt	197
SYNJ1	NM_203446	GAGUGGUACAGUUCGAACAtt	UGUUCGAACUGUACCACUCtg	171
SYNJ2	NM_003898	CAGUGAAGAUUAGACCGAAtt	UUCGGUCUAAUCUUCACUGct	113
SYNJ2	NM_003898	CCUUCAAUCACCGACCUUAtt	UAAGGUCGGUGAUUGAAGGtt	170
SYNJ2	NM_003898	CGUACAAAGAUGACGCGGAtt	UCCGCGUCAUCUUUGUACGtt	275
TNS1	NM_022648	GUUCCGCUCUCAAUCCUUUtt	AAAGGAUUGAGAGCGGAACat	110
TNS1	NM_022648	CCAGGACACUUCUAAGUAUtt	AUACUUAGAAGUGUCCUGGac	333
TNS1	NM_022648	CCAUCAUGCAGCAGAAUAAtt	UUAUUCUGCUGCAUGAUGGtt	171
TNS3	NM_022748	GCUCAUUCAUUGUUCGAGAtt	UCUCGAACAAUGAAUGAGCcc	173
TNS3	NM_022748	CUUACGAAGCUUAACCCAAtt	UUGGGUUAAGCUUCGUAAGgt	179
TNS3	NM_022748	GGAUCUGCAUCGUCAUCGAtt	UCGAUGACGAUGCAGAUCCtg	286
TPTE	NM_199260	CGUUAUGUACGUGAUCUAAtt	UUAGAUCACGUACAUAACGag	103
TPTE	NM_199260	GAAAAUGUUCGGUACUUGAtt	UCAAGUACCGAACAUUUUCct	357
TPTE	NM_199260	AGAAGAGAUAUGUUGCAUAtt	UAUGCAACAUAUCUCUUCUga	183
TPTE2	NM_130785	CGAUAUUGCAUGACAUUGAtt	UCAAUGUCAUGCAAUAUCGaa	82
TPTE2	NM_130785	GAAUAGAUAUGUUGGAUAUtt	AUAUCCAACAUAUCUAUUCtg	165
TPTE2	NM_130785	GAGGAAAGCCUAUAUUAUUtt	AAUAAUAUAGGCUUUCCUCgg	193
