# Supplementary material for: Automated High-Throughput RNAi Screening in Human Cells Combined with Reporter mRNA Transfection to Identify Novel Regulators of Translation
Source: PLoS One. 2012 Sep 27;7(9):e45943. doi: 10.1371/journal.pone.0045943 (PMC3459937; doi:10.1371/journal.pone.0045943)
Supplement: Table S3 — Confirmed IRES specific hits. Gene symbol, RefSeq, sense- and antisense siRNA sequences for the 3 IRES specific hits. (PDF) [file pone.0045943.s005.pdf]

Supplementary Table 3

Gene Symbol	RefSeq	Sense siRNA Sequence 5'-3'	Antisense siRNA Sequence 5'-3'
ALPK2	NM_052947	CAUCGGCCUGAGAACAAUAtt	UAUUGUUCUCAGGCCGAUGga
		CCAUGACCUUCAUUGAUCAtt	UGAUCAAUGAAGGUCAUGGaa
		GGGCUGUACCUGAUAGUCUtt	AGACUAUCAGGUACAGCCCag
DCLK3	XM_940612	GAAUGACCGUGUGAGGAAAtt	UUUCCUCACACGGUCAUUCtt
		CCACUGACACUGAAGAGCAtt	UGCUCUUCAGUGUCAGUGGtt
		GACGAGCUCUUUAACAUCAtt	UGAUGUUAAAGAGCUCGUCct
MAPK3	NM_001040056	GGAUCAGCUCAACCACAUUtt	AAUGUGGUUGAGCUGAUCCag
		GGACCGGAUGUUAACCUUUtt	AAAGGUUAACAUCCGGUCCag
		GACCUGAAUUGUAUCAUCAtt	UGAUGAUACAAUUCAGGUCct
