# Supplementary material for: Automated High-Throughput RNAi Screening in Human Cells Combined with Reporter mRNA Transfection to Identify Novel Regulators of Translation
Source: PLoS One. 2012 Sep 27;7(9):e45943. doi: 10.1371/journal.pone.0045943 (PMC3459937; doi:10.1371/journal.pone.0045943)
Supplement: Table S4 — siRNA control sequences. (PDF) [file pone.0045943.s006.pdf]

Supplementary Table 4

Control	Sense siRNA Sequence 5'-3'	Antisense siRNA Sequence 5'-3'
Scrambled	UUCUCCGAACGUGUCACGUTT	ACGUGACACGUUCGGAGAATT
Firefly	CGGAUUACCAGGGAUUUCATT	UGAAAUCCCUGGUAAUCCGTT
PLK1	CAACCAAAGUCGAAUAUGATT	UCAUAUUCGACUUUGGUUGCC
