# Supplementary material for: Automated High-Throughput RNAi Screening in Human Cells Combined with Reporter mRNA Transfection to Identify Novel Regulators of Translation
Source: PLoS One. 2012 Sep 27;7(9):e45943. doi: 10.1371/journal.pone.0045943 (PMC3459937; doi:10.1371/journal.pone.0045943)
Supplement: Table S5 — Primer sequences used for qRT-PCR experiments. (PDF) [file pone.0045943.s007.pdf]

Supplementary Table 5

Control	Forward primer	Reverse primer
VEGF	CGAAACCATGAACTTTCTGC	CCTCAGTGGGCACACACTCC
MAPK3	GCAGGACCTGATGGAGACTGAC	CCAGAATGCAGCCCACAGAC
ACTIN β	TTCCGCTGCCCTGAGGCACTCT	TCTGCTGGAAGGTGGACAGCGA
